# AgPd, AuPd, and AuPt Nanoalloys with Ag- or Au-Rich Compositions: Modeling Chemical Ordering and Optical Properties

**DOI:** 10.1021/acs.jpcc.1c04222

**Published:** 2021-07-30

**Authors:** Nicola Danielis, Lorena Vega, Giovanna Fronzoni, Mauro Stener, Albert Bruix, Konstantin M. Neyman

**Affiliations:** † Dipartimento di Scienze Chimiche e Farmaceutiche, Università di Trieste, via L. Giorgieri 1, I-34127 Trieste, Italy; ‡ Departament de Ciència del Materials i Química Física & Institut de Química Teòrica i Computacional, Universitat de Barcelona, 08028 Barcelona, Spain; § ICREA (Institució Catalana de Recerca i Estudis Avançats), 08010 Barcelona, Spain

## Abstract

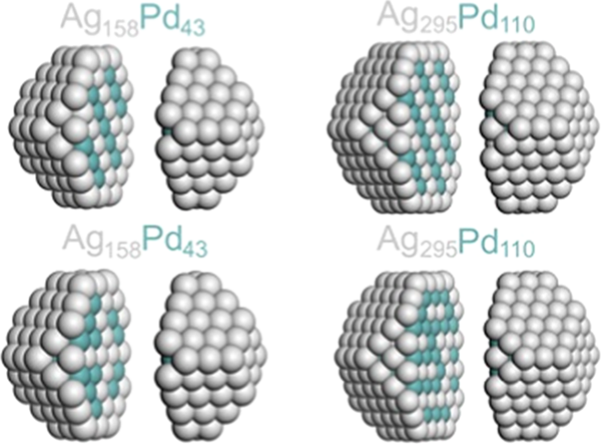

Bimetallic nanoparticles have a myriad of technological applications, but investigations of their chemical and physical properties are precluded due to their structural complexity. Here, the chemical ordering and optical properties of AgPd, AuPd, and AuPt nanoparticles have been studied computationally. One of the main aims was to clarify whether layered ordered phases similar to L1_1_ one observed in the core of AgPt nanoparticles [PirartJ.; Nat. Commun.2019, 10, 19823104027210.1038/s41467-019-09841-3PMC6491558] are also stabilized in other nanoalloys of coinage metals with platinum-group metals, or the remarkable ordering is a peculiarity only of AgPt nanoparticles. Furthermore, the effects of different chemical orderings and compositions of the nanoalloys on their optical properties have been explored. Particles with a truncated octahedral geometry containing 201 and 405 atoms have been modeled. For each particle, the studied stoichiometries of the Ag- or Au-rich compositions, ca. 4:1 for 201-atomic particles and ca. 3:1 for 405-atomic particles, corresponded to the layered structures L1_1_ and L1_0_ inside the monatomic coinage-metal skins. Density functional theory (DFT) calculations combined with a recently developed topological (TOP) approach [KozlovS. M.; Chem. Sci.2015, 6, 3868−38802921815810.1039/c4sc03321cPMC5707449] have been performed to study the chemical ordering of the particles, whose optical properties have been investigated using the time-dependent DFT method. The obtained results revealed that the remarkable ordering L1_1_ of inner atoms can be noticeably favored only in small AgPt particles and much less in AgPd ones, whereas this L1_1_ ordering in analogous Au-containing nanoalloys is significantly less stable compared to other calculated lowest-energy orderings. Optical properties were found to be more dependent on the composition (concentration of two metals) than on the chemical ordering. Both Pt and Pd elements promote the quenching of the plasmon.

## 1 Introduction

Metal nanoparticles (NPs) are very appealing systems since their properties are size-dependent. This opens new frontiers for basic science to prompt competitive technological applications. Structures of metal NPs may differ with respect to their bulk counterpart due to the effect of surface tension. For nanoalloys, two new degrees of freedom—relative content of the constituting metals and chemical ordering—dramatically increase the structural complexity, but also widen the flexibility to tune their properties.^1,2^ In this context, computational tools not only are fundamental for the understanding of NPs, but their usage is also a promising way for the knowledge-driven design of new materials with tailored properties.^3^

Computational modeling studies of the optical properties of nanoalloys pose two problems. First, the structure, composition, and chemical ordering need to be assessed. Then, the photoabsorption spectrum has to be calculated for particles of such a large size that it is still challenging for standard methods of quantum chemistry. Since metal nanoparticles typically form fcc-type structures with well-known shapes, determining the equilibrium chemical ordering in heterometallic NPs is the most tricky structural aspect. Indeed, the combinatorial number of homotops (isomers) for a fixed particle shape is so high that a sophisticated approach must be adopted to make this problem tractable. In this work, the chemical orderings in different bimetallic NPs have been assessed within the recently developed topological (TOP) method relying on the topological energy expressions parameterized using a small number of density functional theory (DFT) calculations.^4,5^ The TOP method is a powerful computational tool that is broadly applicable to binary nanoalloys^6−10^ and enables globally exploring configurational space of two metal elements in different crystalline positions of a bimetallic NP with given stoichiometry and morphology by means of Monte-Carlo (MC) minimization and subsequent DFT geometry relaxation. The minimum-energy homotop identified in the configurational space represents the most stable distribution of the two elements over the lattice formed by the atomic positions of the NP, i.e., its equilibrium chemical ordering. Energies of other higher-energy arbitrary chemical orderings, also taking temperature effects into account, can also be evaluated within the TOP approach.^4,5^ Often, atoms of one of the two elements comprising bimetallic NPs exhibit a clear preference to occupy inner or surface sites.^1,11^ Another important aspect of chemical ordering is whether different metal atoms prefer to mix, forming heteroatomic bonds in either seemingly disordered or layered structures. These details are of special importance because experimental structural data detailing atomic positions of all different elements in bare bimetallic particles are scarce.^12^ In addition, one cannot exclude that the interactions of the particles with the support and adsorbed molecules notably affect the chemical ordering.

After the chemical ordering issue has been tackled, it is possible to calculate the optical spectra, employing the geometry of the most stable homotop for each NP. It is worth noting that NPs comprising several hundred transition metal atoms are challenging for the standard methods of quantum chemistry. Currently, time-dependent density functional theory (TDDFT) is the only formalism capable of dealing with systems of such size and represents the best compromise between accuracy and computational effort.

In photoabsorption experiments, it has been found that while pure Ag NPs display strong surface plasmon resonance (SPR), such feature is quenched in bimetallic AgPt NPs, unless a very large size (around 10 nm) is reached.^13^ A similar role of Pt as a “poison” for the SPR in AgPt clusters has also been considered by Barcaro et al.^14^ where it has been found that although pure silver particles exhibit very intense and sharp plasmon resonance, it is enough to distribute a few Pt atoms on the particle surface to suppress the plasmon.

The size increase of a nanoscale system is usually accompanied by increasing the stability of its ordered structures.^15^ However, an intriguing counterexample to this trend has been recently demonstrated by the formation of a peculiar ordered layered phase L1_1_ inside AgPt NPs smaller than 2.5 nm, whereas in larger NPs the ordering disappeared.^16^ The L1_1_- phase stabilization in small NPs was associated with the formation of a monolayer-thick Ag outermost shell preventing Pt from occupying surface sites. This Ag shell has been calculated to exert increasing stress on the L1_1_ layered domains with increasing particle size making the alternating −Ag–Ag– and −Pt–Pt– layers energetically unfavorable, when the NP exceeds the critical size of ca. 2.5 nm. A computational modeling study by some of the authors of the present study dealt with the chemical ordering in AgPt NPs of increasing size at different compositions and revealed the favorable formation of the Ag shell.^17^ There, systems with L1_1_- and L1_0_-ordered AgPt cores were modeled by 116-, 140- and 201-atom truncated octahedral crystallites with fcc structures.

The present work explores, by means of the computational methods described above, if layered structures similar to the structure experimentally observed in the inner part of AgPt NPs are also characteristic at certain sizes of bimetallic AxPx NPs formed by combining the coinage metals (Ax = Ag, Au) with the platinum-group atoms (Px = Pd, Pt). More specifically, it is studied whether the stress effect of the skins of the coinage metal atoms can stabilize L1_1_ (or L1_0_) orderings in bimetallic cores of AgPd, AuPd, and AuPt NPs. For that, truncated octahedral NPs composed of 201 and 405 atoms are considered with the compositions enabling perfect layered L1_1_ and L1_0_ structures of the inner parts of the NPs.

Most of the studies carried out on AgPd alloys are related to their applications as catalysts for hydrogenation,^18−21^ fuel cell processes,^22^ and other reactions^23−25^ as well as sensors.^26^ Research on bulk AgPd alloys showed complete miscibility at all compositions based on the thermodynamic modeling of experimentally determined liquid points and thermochemical properties in the solid phase.^27^ Theoretical modeling studies of AgPd NPs revealed Ag-shell/Pd-core chemical ordering,^4,28^ in line with experimental data.^20,21^

The interest in AuPd nanoalloys is due to their applications in heterogeneous catalysis, which include processes of H_2_O_2_ synthesis,^29^ CH_4_ conversion to methanol,^30^ and oxygen reduction.^31^ Previous studies on bulk AuPd systems showed the presence of a continuous solid solution based on experimentally determined thermodynamic functions.^32^ Ordered phases were observed for two stoichiometric compositions, Au_3_Pd and AuPd_3_.^32^ The miscibility of the two components was evidenced by calorimetric measures of the enthalpy of mixing, which assumes negative values for all compositions. Theoretical studies predicted the Au-shell/Pd-core structure as the most thermodynamically stable in NPs of various sizes and compositions.^4,11,33,34^ Very recently, critical compositions of AuPd NPs have been established, at which single-surface Pd atoms are stabilized inside the monatomic Au skin.^35^ Interestingly, a variety of different structures have been observed experimentally.^36−38^

Bimetallic Pt-based catalysts have been proposed as promising alternatives to the exceedingly expensive pure Pt ones. In particular, AuPt NPs have been studied intensively because of their interesting optical properties,^39^ their potential as selective oxidation, and dehydrogenation catalysts^40−42^ and electrocatalysts.^43−45^ Many studies have been focused on the determination of the phase diagram of bulk AuPt alloys. These studies showed the presence of a large solid miscibility gap when the concentration of Pt exceeds ∼10%.^46^ This phase segregation was also supported by the enthalpy of formation displaying increasing positive values as the Pt concentration increases. Experimental studies carried out on AuPt NPs showed the formation of two different core–shell arrangements: Pt-shell/Au-core forms as the kinetic product since Au is more easily reduced, whereas Au-shell/Pt-core forms as the thermodynamic product since Au has lower surface energy than Pt^47,48^ (and Au atoms are larger than Pt ones).

On the other hand, a more recent experimental work has found complete alloying for Au–Pt nanoparticles supported on carbon,^49^ so at the moment it seems impossible to reliably and experimentally define the equilibrium chemical ordering of such bimetallic nanoparticles structurally unaffected by their environment.

## 2 Theory

### 2.1 Structures and Homotops: the TOP Method

DFT calculations of bimetallic NPs with more than a hundred atoms are feasible for two decades.^50^ However, the presence of more than one type of atom in nanoalloys severely hinders their comprehensive computational studies. The topological energy (TOP) method employed in the present study defines the energy of each homotop of a chosen AxPx NP (with a fixed shape and stoichiometry) as follows^4^
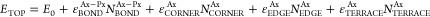
1where *E*_0_ is a constant offset between the TOP and DFT energy scales, *N*_BOND_^Ax–Px^ is the number of heterometallic bonds (nearest-neighbor contacts Ax–Px) in the homotop under scrutiny, and *N*_CORNER_^Ax^, *N*_EDGE_^Ax^, and *N*_TERRACE_^Ax^ are the numbers of Ax atoms in the corner (vertex), edge, and terrace positions, respectively. The terms ε represent the energy contributions to the total energy of the homotop *E*_TOP_ of either one Ax–Px bond or one Ax atom located in the corresponding surface position of the NP. Note that the expressions with the structure of eq 1 accurately describe chemical ordering at the surface of bimetallic NPs relevant for catalysis, whose reactivity is mainly determined by the atoms exposed on the surface. The chemical ordering of the inner atoms of the NPs is just partially accounted for in eq 1 via the number of the heterometallic bonds and the associated energy term ε. This is in some cases insufficient for finding the true equilibrium arrangement of the NP core.^4^ Given that the as-formulated TOP method does not discern between homotops differing in the ordering of the core, we have also calculated energies of structures with the layered cores L1_0_ and L1_1_. Homotops with lower DFT energies than that of the lowest-energy homotops obtained with the TOP method (referred to as low-energy homotop (LEH) in the following) indeed emerge (vide infra) for some compositions as a result of the core order.

Equation 1 is parameterized by fitting the energy terms ε_*i*_ to DFT energies of several dozen distinct homotops with various orderings of Px and Ax atoms represented via the topological numbers *N*_*i*_. The MC-minimization using eq 1 for estimating TOP energies is carried out, and ≤50 low-lying homotops above the lowest-energy homotop found are selected for reparametrization of eq 1. Each MC run includes several million minimization steps, and subsequent parametrizations and MC runs are carried out until the resulting low-energy homotops displayed the same topology, characterized by the same set of *N*_*i*_ numbers. NP structures are locally relaxed to obtain DFT energies of different homotops used for parametrizing eq 1. To evaluate the quality of the resulting fit, the precision δ and accuracy Δ*E* are calculated. Precision δ is defined as twice the residual standard deviation between the *E*_DFT_ and *E*_TOP_ energies. Accuracy Δ*E* is defined as the difference between *E*_TOP_ energies of the lowest-energy homotops obtained from the TOP and DFT optimizations of the chemical ordering.

The periodic plane-wave code VASP^51^ is used to perform the DFT calculations, employing the Perdew–Burke–Ernzerhof (PBE)^52^ exchange–correlation functional, quite reliably describing transition metals.^53−55^ Interactions between the valence and core electrons are described within the projected augmented wave (PAW) scheme.^56,57^ As in our previous studies,^4,5^ the energy cutoff of the plane-wave basis sets corresponded to the default PAW values (∼251 eV for AgPd and AuPd NPs and ∼230 eV for AuPt NPs). The 201-atom NPs are located in the unit cells 2.5 × 2.5 × 2.5 nm^3^ large and the 405-atom NPs occupied the unit cells 2.8 × 2.8 × 2.8 nm^3^. These sizes of the unit cells provide sufficient vacuum space between adjacent periodically repeated NPs for making interactions among them negligible.^58,59^ The one-electron states were smeared by 0.1 eV employing the first-order method of Methfessel and Paxton;^51^ the converged energies were extrapolated to zero smearings. Only Γ-point calculations are performed. All atoms are allowed to locally relax during the geometry optimization.

### 2.2 Optical Properties: the TDDFT Complex Polarizability

In the present work, the absorption spectrum is extracted from the imaginary part of the complex dynamical polarizability, solving the TDDFT equations over the space of the density instead of the occupied virtual pairs of the density matrix. The induced time-dependent density is represented employing an auxiliary basis set of Slater-type orbitals (STOs).^60−62^ Such method (referred to as complex polarizability TDDFT algorithm) has proven to be efficient to describe metal particles with many hundreds of atoms.^63^

The ADF program (distributed version 2018) has been used to calculate the optical properties at the time-dependent DFT (TDDFT) level. The PBE exchange-correlation (xc-) functional^52^ is chosen to solve the Kohn-Sham equations, while the adiabatic local density approximation^64^ (ALDA) is used in the TDDFT part for the exchange-correlation kernel. The basis set employed is included in the ADF code and consists of STOs of triple-ζ-polarized (TZP) quality with frozen core (FC) up to 4p shell for Ag and Pd atoms, and 4f shell for Au and Pt atoms. Relativistic effects (which are important for heavy elements such as platinum) have been treated at the zero-order regular approximation (ZORA) level.^65^

The individual component map of the oscillator strength (ICM-OS)^66^ tool has been employed to analyze details of the electronic transitions. This tool allows describing a specific absorption peak in terms of the energy of occupied (*x*-axis) and virtual (*y*-axis) orbitals involved to associate a specific nature to a particular electronic transition. Briefly, the diagonal line formed by the most intense features in the ICM-OS plots corresponds to the energy of the exciting photon (indicated above each plot), which results from the energy difference between the virtual and occupied molecular orbitals. Therefore, spots on the diagonal visualize single excitations, whereas the presence of off-diagonal features indicates a collective behavior typical of plasmons. Such features are characterized by occupied and virtual orbitals with smaller energy differences than the analyzed excitation energy, indicating that the excitation involves more than a single particle. In addition to the weight of each pair of orbitals in the oscillator strength, ICM-OS also accounts for dipole contributions, which might result in constructive or destructive interferences among excited configurations.

## 3 Results and Discussion

### 3.1 Chemical Ordering

The energy terms ε_*i*_ in eq 1 calculated for AgPd, AuPd, and AuPt nanoalloys are listed in Table 1 together with their confidence intervals and the resulting precision and accuracy values. Before analyzing the chemical ordering, the following comments are due: (i) confidence intervals of the terms ε_*i*_ depend not only on the number of homotops *N*_FIT_ in the fitting procedure but also on the diversity of each of the *N*_*i*_ values in the set of homotops chosen for fitting and on the overall capacity of the topological parameters to represent different structures. Therefore, confidence intervals of the terms ε_*i*_ for lower-coordinated surface sites (showing a strong propensity to be occupied solely by Ax atoms) are wider than for terrace surface sites with higher coordination. (ii) Precision δ and accuracy Δ*E* of eq 1 (defined in the Theory section) are generally better for 201-atom particles than for 405-atom ones, reflecting larger numbers of nonequivalent homotops with identical topologies for larger particles. In all cases, δ and Δ*E* values remain reasonably small, below 2 meV/atom.

**Table 1:** Topological Energy Terms ε (Equation 1)^a^ for Bimetallic Nanoparticles AxPx Containing Atoms of a Coinage Metal Ax (Ag or Au), and a Platinum-Group Metal Px (Pd or Pt)

nanoalloy	particle	ε_BOND_^Ax–Px^ (meV)	ε_CORNER_^Ax^ (meV)	ε_EDGE_^Ax^ (meV)	ε_TERRACE_^Ax^ (meV)	δ^b^ (meV)	Δ*E*^c^ (meV)	*N*_FIT_^d^
AgPd	Ag_161_Pd_40_	–12_–1_^+1^	–456_–189_^+135^	–249_–109_^+85^	–88_–64_^+52^	151	124	33
	Ag_300_Pd_105_	–20_–7_^+11^	–727_–925_^+242^	–351_–223_^+331^	–124_–45_^+29^	627	318	42
	Ag_158_Pd_43_	–12_–1_^+1^	–389_–80_^+193^	–403_–43_^+43^	–97_–12_^+15^	241	0	42
	Ag_295_Pd_110_	–19_–5_^+13^	–822_–1240_^+375^	–333_–135_^+624^	–133_–24_^+23^	404	385	49
AuPd	Au_161_Pd_40_	–24_–9_^+10^	–568_–221_^+97^	–273_–104_^+127^	–228_–34_^+39^	271	0	28
	Au_158_Pd_43_	–28_–15_^+10^	–403_–119_^+234^	–419_–111_^+68^	–210_–32_^+43^	174	0	27
AuPt	Au_161_Pt_40_	11_–4_^+3^	–953_–699_^+372^	–393_–127_^+135^	–342_–89_^+43^	250	0	29
	Au_300_Pt_105_	21_–1_^+0^	–789_–372_^+192^	–604_–221_^+75^	–248_–28_^+40^	368	86	49
	Au_158_Pt_43_	14_–5_^+4^	–574_–70_^+651^	–533_–106_^+100^	–280_–87_^+59^	161	0	30
	Au_295_Pt_110_	17_–1_^+1^	–850_–209_^+181^	–373_–46_^+47^	–370_–13_^+16^	296	169	48
AgPt^e^	Ag_151_Pt_50_	10_–3_^+2^	–601_–168_^+181^	–307_–94_^+106^	–210_–21_^+19^	239	40	62

a 95% confidence interval of ε given as _–*l*_^+*k*^; e.g., −20_–7_^+11^ means the ε range from −27 to −9 meV.

b Precision of the TOP energy expression δ is defined as twice the residual standard deviation between the *E*_DFT_ and *E*_TOP_ energies.

c Accuracy of the TOP energy expression Δ*E* is defined as the difference between *E*_TOP_ energies of the lowest-energy homotops obtained from the TOP and DFT optimizations of the chemical ordering.

d Number of homotops used in the fitting procedure to calculate the topological energy terms.

e Values taken from ref (17).

The first observation from Table 1 is that all energy terms ε_*i*_ corresponding to numbers of Ax atoms occupying surface sites (i.e., corners, edges, and terraces) are negative for all compositions. This reveals a strong preference of the coinage atoms Ag and Au to occupy the outer sites with respect to Pd and Pt atoms, forming a complete skin (or shell) of Ax atoms. Such monolayer skins in all low-energy structures described in Table 2 contain 24 corner, 36/60 edge, and 62/120 terrace Ax atoms in the 201-/405-atom AxPx NPs. The second observation is related to different trends in the (small in magnitudes) heterometallic bond terms ε_BOND_^Ax–Px^, which are negative for AgPd and AuPd NPs and positive for AuPt and AgPt^17^ NPs. This indicates that mixing of the coinage metals Au and Ag is slightly more favored with Pd than with Pt. Further inspection of the structures with the lowest topological energy (*E*_TOP_) reveals the following:

- in AgPd and AuPd NPs, the structures with the lowest energies *E*_TOP_ show an outer skin completely composed of the coinage metal, inside which the constituting metals are mixed;
- in AuPt NPs, the structures with the lowest energies *E*_TOP_ also contain an outer skin completely formed of the coinage metal atoms, but inside it, the two metals do not show a tendency to mix, forming a core composed solely of Pt and an incomplete two-layer skin.

**Table 2:** Calculated DFT Relative Energies *E*_rel_, Exchange Energies *E*_ex_ (see Equation 2) and Number of Heterometallic Bonds *N*_BOND_^Ax–Px^ of the Layered Homotops (L1_0_, L1_1_) and the Lowest *E*_TOP_ Homotops (LEH, Resulted from the TOP Search) for 201- and 405-Atom AxPx Nanoparticles^a^

nanoalloy	particle	homotop	*E*_rel_ (meV)	*E*_ex_ (meV)	*N*_BOND_^Ax–Px^
AgPd	Ag_161_Pd_40_	L1_0_	–412	–71	368
		LEH	0	–69	382
	Ag_300_Pd_105_	L1_0_	1684	–80	932
		LEH	0	–84	932
	Ag_158_Pd_43_	L1_1_	(−1762) −334	–75	336
		qL1_1_^b^	–457	–76	338
		LEH	0	–74	396
	Ag_295_Pd_110_	L1_1_	–204	–87	804
		qL1_1_^c^	–220	–87	804
		LEH	0	–86	942
AuPd	Au_161_Pd_40_	L1_0_	486	–84	368
		LEH	0	–86	384
	Au_158_Pd_43_	L1_1_	1880	–81	336
		LEH	0	–91	396
AgPt^d^	Ag_158_Pt_43_	L1_1_	(3619) −1651	–59	336
		qL1_1_	–1420	–58	
		LEH	0	–51	202
	Ag_161_Pt_40_	L1_0_	(7868) 1679	–40	368
		LEH	0	–48	196
AuPt	Au_161_Pt_40_	L1_0_	3941	–13	368
		LEH	0	–32	176
	Au_300_Pt_105_	L1_0_	12966	–15	932
		LEH	0	–47	334
	Au_158_Pt_43_	L1_1_	2464	–22	336
		LEH	0	–35	192
	Au_295_Pt_110_	L1_1_	5082	–33	804
		LEH	0	–46	360

a Energies of the relaxed layered nanoparticles without the Ag_122_ skins (skin-less ones) with respect to the skin-less LEH particles are given in parentheses.

b Quasi-perfect ordering, obtained from the perfect layered L1_1_ homotop by swapping two pairs of atoms Ax–Px.

c Quasi-perfect ordering, obtained from the perfect layered L1_1_ homotop by swapping one pair of atoms Ax–Px.

d Values taken from ref (17).

We note that a perfect layered ordering of core atoms is only possible for specific stoichiometries of given particle size, i.e., Ax_161_Px_40_ for L1_0_ and Ax_158_Px_43_ for L1_1_ orderings of 201-atom particles. The obtained DFT energies for the layered L1_1_ and L1_0_ homotops and for the corresponding lowest-energy homotops (LEH) provided by the TOP method are given in Table 2. The difference in stability is also assessed by the excess energy *E*_ex_,^11,67^ an indicator (sometimes referred to as mixing energy) representing the energy gain/loss due to mixing of metal components and calculated as
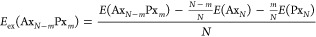
2where *N* is the total number of atoms in the AxPx NP containing *m* atoms Px.

#### 3.1.1 AgPd

**Figure 1:**
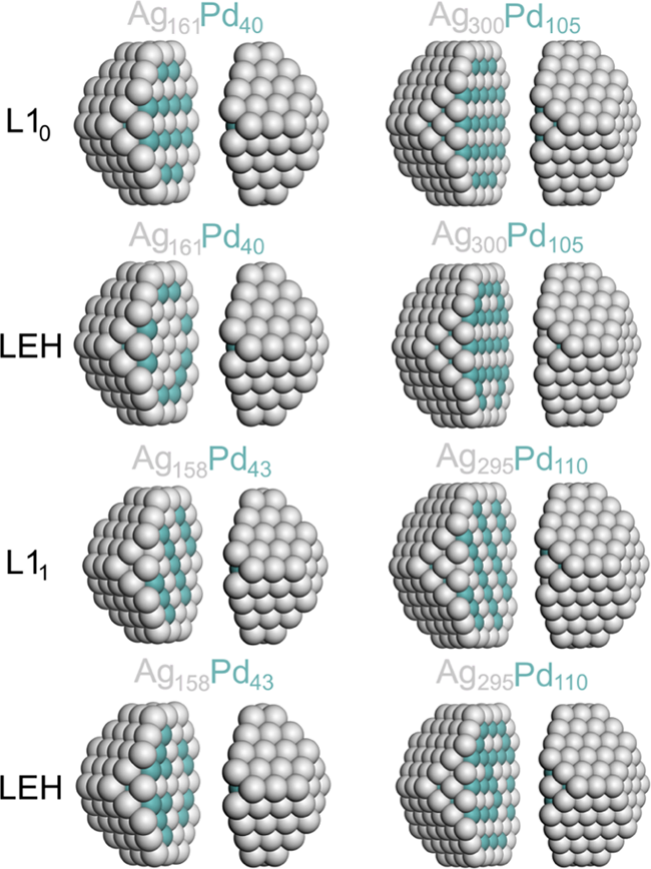
Sketches of the calculated structures of 201- and 405-atom AgPd NPs with different atomic orderings. The images are split to show inner parts of the NPs.

The low-energy structures of AgPd NPs optimized by DFT are sketched in Figure 1. From the section of the particles, it is possible to see the different ordering relative to the L1_1_ and L1_0_ inner-ordered phases, with planes of atoms of different natures alternating parallel to (111) and (001) planes, respectively.^68^

Relative energies *E*_rel_ of Ag_161_Pd_40_ NPs (Table 2) show the stabilizing formation of perfect inner layers for the L1_0_ homotop. The L1_0_ ordering is ∼0.4 eV more stable than the LEH ordering. In contrast, for Ag_300_Pd_105_ NPs, the layered L1_0_ ordering is ∼1.7 eV less stable than the LEH. The energies of different chemical orderings in Ag_158_Pd_43_ and Ag_295_Pd_110_ NPs show that the perfect inner layers L1_1_ are more stable compared to the corresponding LEH orderings by ∼0.3 eV and ∼0.2 eV, respectively. The less intense stabilization of the ordered inner-phase L1_1_ with increasing the NPs size is consistent with the reversed size-dependent stabilization of the L1_1_ ordering observed for AgPt NPs.^16^ However, stabilization of the L1_1_ ordering with respect to LEH ones for AgPt NPs (see Table 2 for values and Figure S1 for structures) was calculated to be several times stronger,^17^ indicating a notably decreased propensity of AgPd NPs to form the peculiar L1_1_ ordering compared to analogous AgPt NPs.

The topologies of the LEH and layered AgPd NPs differ solely in the number of the heterometallic bonds *N*_BOND_^Ag–Pd^ because the outer skins are all completely formed by Ag atoms, 122 in 201-atom NPs, and 204 in 405-atom NPs. Interestingly, the weak stabilization of L1_1_ and some L1_0_ structures vs corresponding LEH ones, accompanied by decreasing numbers of (very slightly) stabilizing heterometallic bonds (Table 2) is not reproduced by the present topological energy approximation (eq 1), which lacks, for instance, energy terms ε directly accounting for interactions in alternating Ag and Pd layers. Such terms allowed to model layered L1_0_ orderings in PdZn NPs,^4^ but including them in eq 1 for AgPd particles kept TOP energies of the inner-layered homotops above the corresponding LEH energies.

To further examine stabilities of the perfect inner-layered orderings L1_1_ in the 201- and 405-atom NPs, DFT energies of ten homotops with quasi-perfect orderings qL1_1_ were calculated for each NP size. These homotops were obtained by swapping positions of one pair (single swap) or two pairs (double swap) of different Ag and Pd atoms in the core of the perfect structure L1_1_ (see Figure S2 in the Supporting Information). As shown in Table 2, some homotops with such qL1_1_ orderings are lower in energy than the corresponding defect-free L1_1_ homotops, even if it is only by ∼0.12 eV (Ag_158_Pd_43_) and <0.02 eV (Ag_295_Pd_110_). This indicates that for AgPd particles, the most stable ordering does not correspond to the perfect layered structures and one may be able to find even lower-energy orderings than the ones presented in this work. At variance, test calculations of exactly the same homotops qL1_1_ of the Ag_158_Pt_43_ NP revealed that they are destabilized by at least 0.17 eV over the perfect-ordering homotop L1_1_ (see ref (17) and Table 2).

#### 3.1.2 AuPd

**Figure 2:**
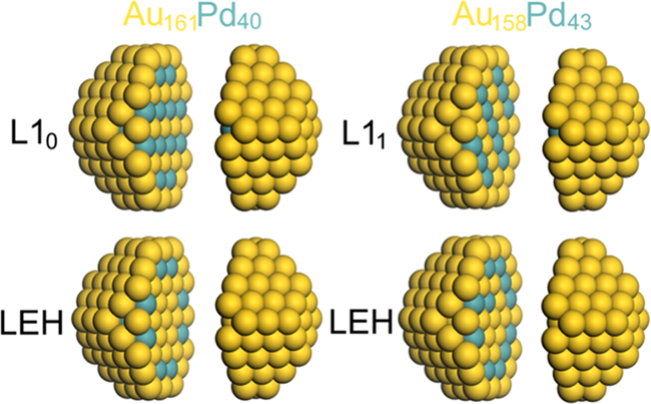
Sketches of the calculated structures of 201-atom AuPd NPs with different atomic orderings. The images are split to show the inner parts of the NPs.

The various low-energy structures of AuPd NPs studied are displayed in Figure 2. Their DFT energies (Table 2) show that the formation of neither the structure L1_0_ in Au_161_Pd_40_ NP nor the structure L1_1_ in Au_158_Pd_43_ NP is stable. Interestingly, the destabilization of the structure L1_1_ versus the LEH one, ∼1.9 eV, is noticeably larger than that of the structure L1_0_, ∼0.5 eV.

#### 3.1.3 AuPt

**Figure 3:**
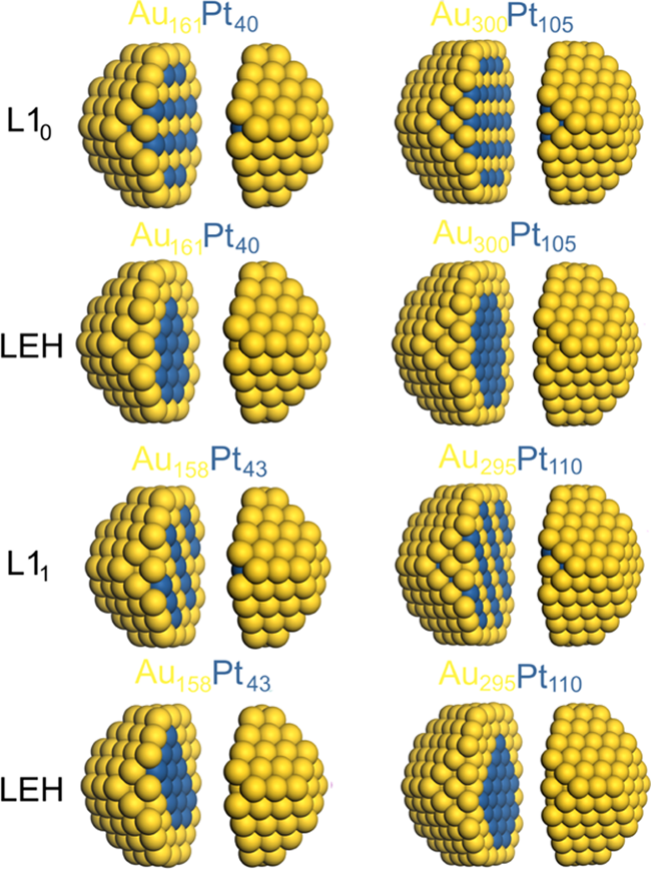
Sketches of the calculated structures of 201- and 405-atom AuPt NPs with different atomic orderings. The images are split to show the inner parts of the NPs.

Structures of AuPt NPs are presented in Figure 3. Comparison of DFT energies (Table 2) of Au_161_Pt_40_ and Au_300_Pt_105_ NPs shows that the layered structures L1_0_ are less stable by as much as ∼4 and ∼13 eV, respectively. The structures with perfect alternating inner layers L1_1_ in Au_158_Pt_43_ and Au_295_Pt_110_ NPs are also notably less stable compared to the LEH structures (∼2.5 and ∼5.1 eV, respectively), although the energy differences are even larger in the case of L1_0_ analogues. The significant destabilization of the layered structures of AuPt NPs, with many more heterometallic bonds than in the LEH structures, is consistent with the trends in the stabilities found at the TOP level according to which formation of the heterometallic Au–Pt bonds is an energetically unfavorable process with respect to the formation of an equivalent number of Pt–Pt and Au–Au bonds.

#### 3.1.4 Core–Skin Interaction

The stability of AgPt particles with a layered inner core has been previously rationalized by Pirart et al.^16^ in terms of the interaction between the layered core and the surrounding Ag skin, where the resulting stress is experienced by the former. To evaluate this effect, we have calculated the relative energies of selected NPs after locally relaxing the structure without the Ag skin (see values in parentheses for Ag_158_Pt_43_, Ag_161_Pt_40_, and Ag_158_Pd_43_ in Table 2). We have found that in the absence of the Ag skin, LEH structures are strongly energetically favored with respect to the layered structures for AgPt particles, with energy differences of up to ∼−3.6 and ∼−7.9 eV compared to Ag_158_Pt_43_ L1_1_ and Ag_161_Pt_40_ L1_0_, respectively. Interaction with the Ag skin therefore significantly affects the relative stability of the layered L1_1_ and L1_0_ orderings by ∼−5.3 and ∼−6.2 eV vs the LEH one, even inverting the stability between L1_1_ and LEH for the Ag_158_Pt_43_ particle. In contrast, for the Ag_158_Pd_43_ particle, the skin-less L1_1_ core is already ∼1.8 eV more stable than the skin-less LEH core, and the interaction with the Ag skin stabilizes the LEH ordering more than the L1_1_. The interaction with the Ag skin, therefore, affects the stability of AgPt and AgPd particles with layered core orderings in opposite directions, stabilizing them for the former and destabilizing them for the latter.

#### 3.1.5 Test with the PBEsol Exchange–Correlation Functional

To estimate the dependence of the ordering patterns of the NPs on the choice of the xc-functional, calculations of 20 selected homotops for each of the 201-atom NPs Ag_158_Pd_43_, Au_158_Pd_43,_ and Au_158_Pt_43_ with the inner-layering L1_1_ were performed using the PBEsol xc-functional.^69^ This functional was chosen to account for overestimated interatomic distances in bulk metals by the PBE functional, while the PBEsol functional reproduces the experimental distances more accurately.^53,54^ Indeed, both homo- and heterometallic distances obtained with PBE xc-functional for the AxPx NPs are notably longer than the corresponding PBEsol distances (Table S1). Nevertheless, the respective TOP energy terms ε_*i*_ calculated at the PBE and PBEsol levels for these 20 selected homotops (Table S2) are close enough to each other to conclude that the PBE ordering patterns of the NPs discussed above will not be significantly modified at the PBEsol level.

### 3.2 Electronic Structure

Since the photoabsorption properties are intimately connected with the electronic structure of a system, the chemical ordering and composition in bimetallic NPs play an important role in determining their optical response. Moreover, since the complex polarizability TDDFT algorithm at the moment can be employed only for closed-shell electronic structures, the charge of the NPs has been chosen to provide a closed-shell configuration. So, while during the atomic ordering optimization neutral particles have been considered, for the analysis of the electronic structure and the optical properties the particle charge has been adapted accordingly. Therefore, a comparison between the partial density of states (PDOS) of the most stable bimetallic NPs with different orderings and compositions of atoms is helpful for the subsequent analysis of the photoabsorption spectra. The PDOS allows determining the weight of an atomic function in a one-electron level; hence, the total density of states (TDOS) can be decomposed into the contributions of the two metal species constituting the NP.

The energy scales in all PDOS plots discussed in the following are referenced with respect to the Fermi level, and the peaks are broadened using Lorentzian curves with 0.12 eV width. PDOS plots for different chemical orderings and charges of [Ag_158_Pd_43_]^q^ NPs (LEH/+2 and L1_1_/neutral) are compared in Figure S3. The comparison shows no substantial differences in the profiles of both the partial contributions and, as a consequence, of the total DOS. As a result, we conclude that different chemical orderings in the core of NPs with the same composition and stoichiometry do not significantly affect their electronic structure, even if the total charge of the system differs.

**Figure 4:**
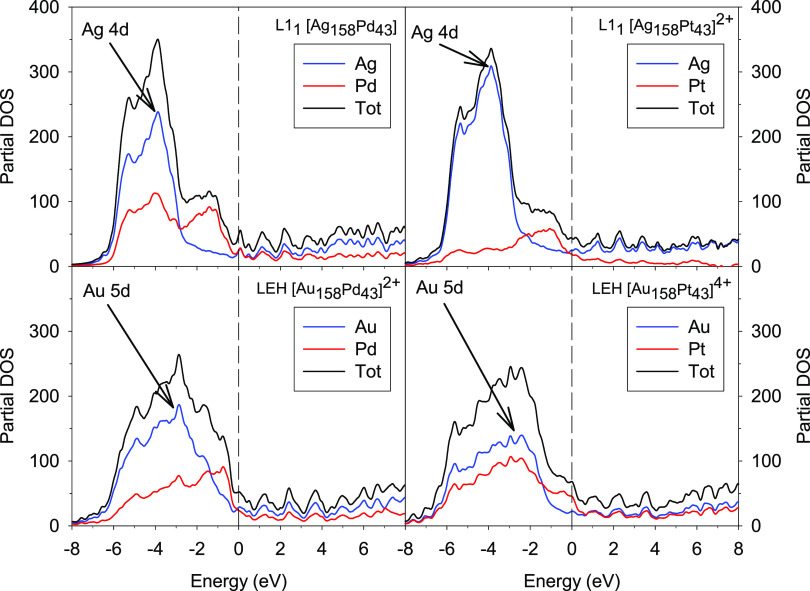
Comparison between the PDOS plots of Ag_158_Pd_43_, [Ag_158_Pt_43_]^2+^, [Au_158_Pd_43_]^2+^, and [Au_158_Pt_43_]^4+^ NPs.

At variance, different chemical compositions result in more pronounced variations in the shape of the PDOS plots shown in Figure 4. The plots of Ag nanoalloys show an intense band between −6 and −2 eV, which corresponds to the 4d orbitals of silver, while in Au nanoalloys the band is broader and less intense. This broadening of the 5d gold band with respect to the 4d silver one can be ascribed to the strong hybridization between 5d and 6s gold atomic orbitals. The relativistic effects strongly reduce the 5d/6s gap favoring hybridization and widening the Au 5d band. Although the total DOS plots of Ag nanoalloys are quite similar, the overall contribution of Pd is higher in both occupied and virtual orbitals than the contribution of Pt.

All of the plots show that the density of states of Pt and Pd just below the Fermi level is higher than that of Ag and Au despite the latter being more abundant. This is attributed to the fact that the Pd and Pt have a less attractive nucleus, having an atomic number one unit less than Ag and Au, which effectively shifts their d band to higher energies with respect to the Fermi level. However, at lower energies, the contribution of Ag and Au increases, which is to be expected due to their larger relative abundance and number of d electrons. Another common feature is that in the virtual states the contribution of the two metal components is comparable, apart from the case of the Ag_158_Pt_43_ NP where only the Ag contribution is relevant. It is worth noting that the Pt DOS for [Au_158_Pt_43_]^4+^ just below the Fermi energy is less pronounced than in the other three particles, and this situation corresponds to the highest ε_BOND_^Ax–Px^ found in Table 1. Such DOS behavior can be ascribed to the unfavored Au–Pt interaction.

### 3.3 Optical Spectra

**Figure 5:**
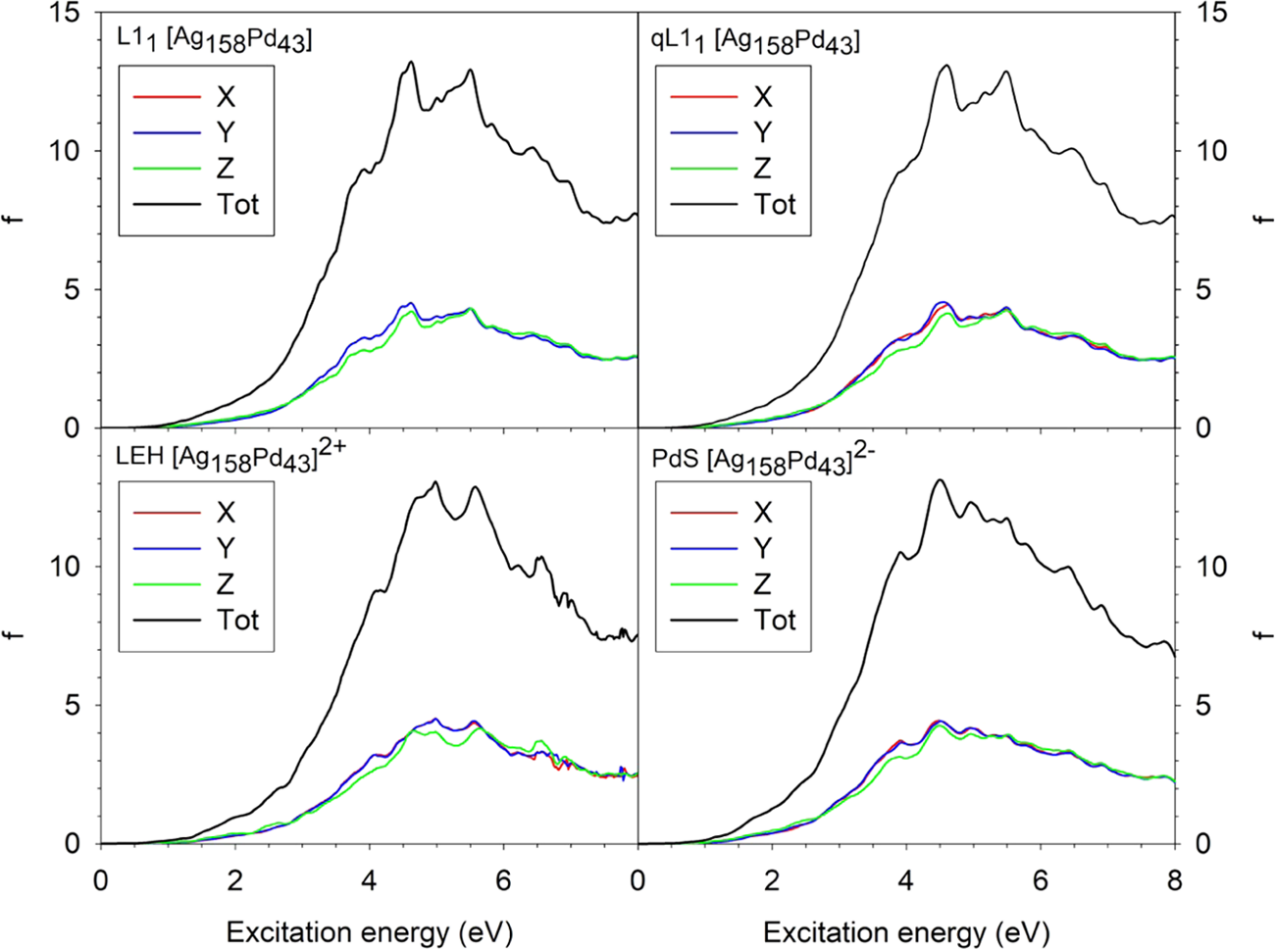
Optical spectra of the closed-shell states of Ag_158_Pd_43_ NPs with different chemical orderings: L1_1_—layered; qL1_1_—almost (quasi) layered; LEH—the low-energy homotop; and PdS—one Pd atom moved on the surface from inside. The intensity is given in oscillatory strength (f), while *X*, *Y*, and *Z* refer to the three components of the electric dipole transition moment.

Photoabsorption spectra were calculated only for the 201-atom NPs. To analyze the influence of the chemical ordering within one composition on the optical properties, we calculated and compared the spectra of four different homotops of Ag_158_Pd_43_ NPs (Figures 5 and S4). In particular, we calculated the photoabsorption spectra of the L1_1_, qL1_1_, LEH, and (described below) PdS homotops. The partial dipole contributions are quite similar to each other in all the spectra considered, although the Z contributions exhibit slightly lower values throughout. The total spectra for the different orderings are also quite similar, with an increase in the oscillator strength up to ∼5 eV followed by its decrease at higher energy values. The resemblance between the L1_1_, qL1_1_, and LEH spectra is due to the fact that the photoabsorption occurs mainly on the surface, which in all of these systems is a complete Ag skin.

To inspect possible effects on the optical spectra due to the formation of a noncomplete Ag skin, calculations were also carried out for a homotop (PdS) exhibiting a surface Pd atom. The PdS homotop was formed by permuting the positions of a surface Ag atom and a bulk Pd in the LEH homotop, increasing the NP energy by only 0.15 eV. The PdS model did not reveal significant differences in the dipole contributions compared to the NPs with complete Ag skin.

It should be noted that the spectra of models L1_1_ and qL1_1_ are almost superimposable (Figure S4), while the LEH and PdS ones show a small shift to higher and lower excitation energies, respectively. Such shifts seem to be caused by the difference in NP charge with respect to the layered structures since the LEH homotop is positively charged and the PdS homotop bears a negative charge instead.

The effect of differences in layer orientation and stoichiometry on the optical properties of NPs with similar chemical ordering is evaluated by comparing the total spectra of the layered L1_0_ [Ag_158_Pd_43_]^3+^ and L1_1_ [Ag_161_Pd_40_] structures and of the nonlayered LEH [Ag_158_Pd_43_]^2+^ and LEH [Ag_161_Pd_40_]^3+^ structures (Figure S5). The comparison of the spectra of the two-layered structures on the left panel shows a redshift for the L1_1_ [Ag_161_Pd_40_] homotop above 2.5 eV and a small intensity increase up to 5.5 eV. A small intensity increase for the LEH [Ag_158_Pd_43_]^2+^ with respect to the LEH [Ag_161_Pd_40_]^3+^ is also observed between 3.0 and 5.0 eV (Figure S5, right panel). The shifts for both ordering types (L1 and LEH) seem to be due to the differences in NP charge, with more positively charged particles exhibiting shifts toward higher excitation energies and is consistent with the trend observed in Figure S6. The overall analysis shows that neither different mixing patterns in the core of the NPs nor their slightly varied stoichiometry leads to significant changes in the shape of the optical spectra.

**Figure 6:**
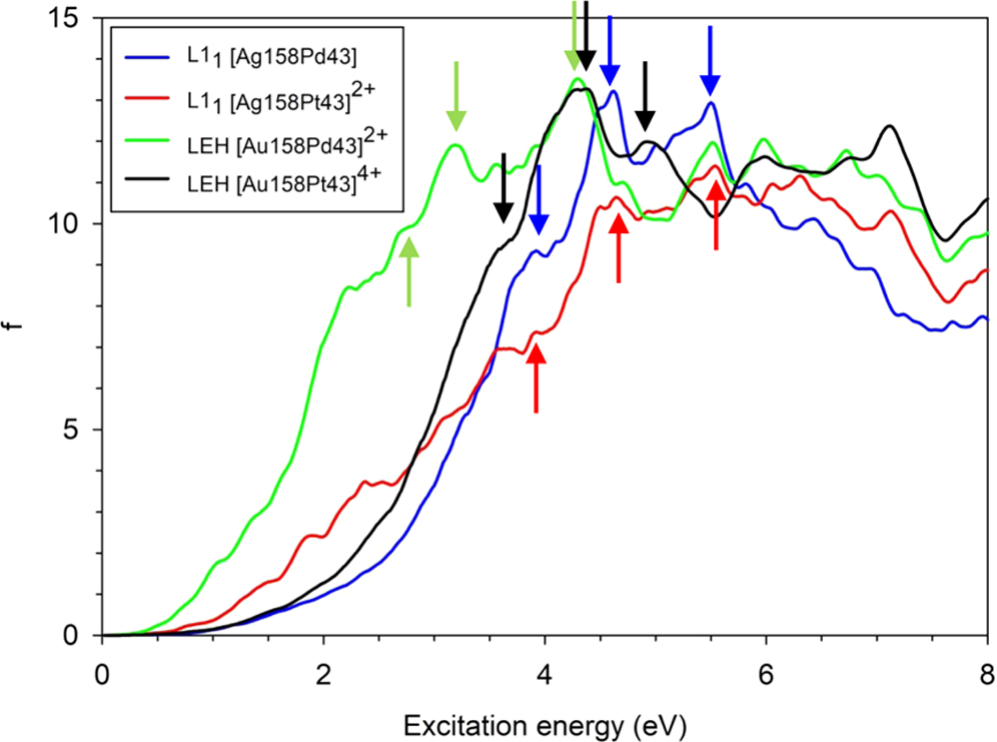
Comparison of the optical spectra of the closed-shell Ag_158_Pd_43_, [Ag_158_Pt_43_]^2+^, [Au_158_Pd_43_]^2+^, and [Au_158_Pt_43_]^4+^ NPs. Three arrows in each of the presented spectra label individual peaks, for which the ICM-OS analysis has been performed (see text and Figure 8).

The effect of different chemical compositions on the optical properties is evaluated by calculating the total spectra of Ag_158_Pd_43_, [Au_158_Pd_43_]^2+^, [Au_158_Pt_43_]^4+^, and [Ag_158_Pt_43_]^2+^ (Figure 6), where the last spectrum is taken from the previous work.^17^ Differences in composition for NPs with the same numbers of the Ax and Px atoms induce a more pronounced effect on the optical response than the differences in the core chemical ordering for a particular composition.

To get a better insight into the variations observed in the optical spectra, it is useful to analyze them in terms of fragment decomposition.^70^ Every NP can be separated into two fragments containing all atoms of one element (Ax or Px) each, and the spectra can be presented as a sum of four different contributions: Ax–Ax, Ax–Px, Px–Ax, and Px–Px. Ax–Ax and Px–Px represent excitations within the same fragment, whereas Ax–Px and Px–Ax correspond to excitations involving electrons transfer from Ax and Px to Px and Ax, respectively.

**Figure 7:**
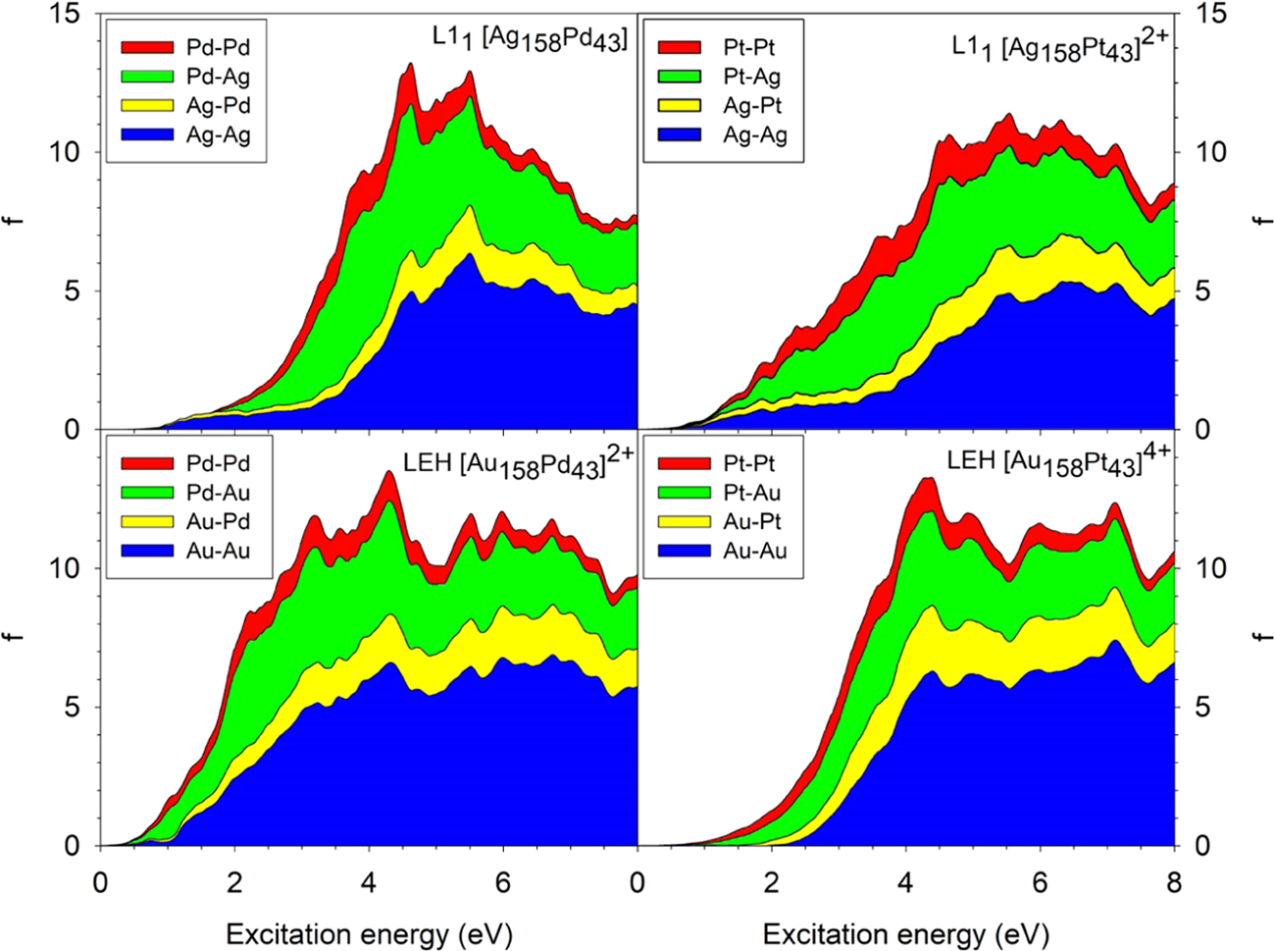
Fragment decomposition spectra of the closed-shell Ag_158_Pd_43_, [Ag_158_Pt_43_]^2+^, [Au_158_Pd_43_]^2+^, and [Au_158_Pt_43_]^4+^ NPs.

Despite the differences in the total profiles of the spectra, the fragment decomposition (see Figure 7) reveals some common trends in the split profiles. First, the weight of the Ag–Ag and Au–Au fragments (blue areas) increases with increasing the excitation energy and becomes dominant. Second, the most important contribution at low excitation energies is the electron transfer from the Px fragments to the fragments of the coinage metal Ax (green areas). Both of these trends are consistent with the PDOS of the NPs displayed in Figure 4: the large contributions of Ax–Ax (blue areas) at high energies is due to both the larger abundance of Ax atoms in Ax_158_Px_43_ NPs and their fully occupied d-band. In turn, the larger contribution of Px–Ax (green areas) at low energies is related to the higher contribution of Pd or Pt occupied orbitals just below the Fermi energy compared to Ag or Au, which is due to the incomplete occupation of the d-band.

Lastly, contributions resulting from electron transfer to Px fragments are the smallest. Although the electron transfers from the coinage metals to Pd or Pt (yellow areas) follow the same evolution as that of the Ag–Ag and Au–Au fragments, they add only a minor contribution to the total oscillator strength. However, the contribution of Pd–Pd and Pt–Pt fragments (red areas) is even smaller, remaining quite constant over the whole interval of excitation energies.

Comparing the different compositions, a significant shift toward lower excitation energies of the spectrum can be seen for the Au_158_Pd_43_ NP in comparison with spectra of the other NPs in Figure 7. This difference can be partially explained by the DOS analysis of Figure 4, where the density of states of the Au_158_Pd_43_ NP is considerably higher just below the Fermi energy than in the case of the other NPs. This is consistent with the higher energy d-band center of Au and Pd compared to the Ag and Pt, respectively.^71^

**Figure 8:**
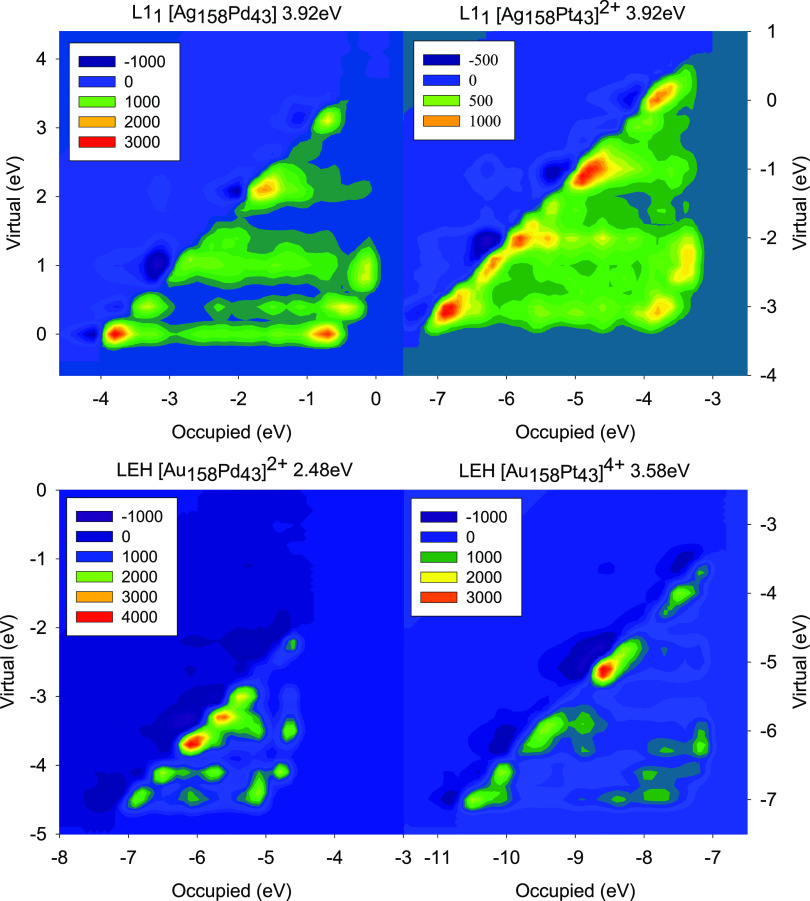
First set (corresponding to the peak position indicated by the left-most arrow of each color in Figure 6) of the ICM-OS of the closed-shell Ag_158_Pd_43_, [Ag_158_Pt_43_]^2+^, [Au_158_Pd_43_]^2+^, and [Au_158_Pt_43_]^4+^ NPs. The excitation energy of the analyzed peak is given above each plot.

To carry out the ICM-OS analysis, three characteristic peaks of the optical spectra were selected (see arrows in Figure 6) for each NP with different chemical compositions. The peaks have been selected taking the most intense and also the minor ones (shoulders) that have counterparts in all the particles to follow the evolution along the series. The resulting ICM-OS plots are compared in Figures 8, S6, and S7. The ICM-OS data in Figure 8 correspond to the peak indicated by the lowest-energy (left-most) arrow in Figure 6. They show, at first sight, a plasmonic behavior (i.e., off-diagonal features) for all examined peaks of different NPs, which is much more intense in the case of Ag nanoalloys. However, according to the fragment analysis of Ag_158_Pd_43_ and Ag_158_Pt_43_ NPs (see Figure 7), the largest contribution to these peaks (both at 3.92 eV) is in both cases provided by the electron transfer from a Pd or Pt fragment to Ag one, together with Ag–Ag contribution. The electron transfer should not give rise to a plasmon due to its noncollective nature. The answer to this apparent contradiction lies in the sign of the dipole contribution; indeed, the upper part of the diagonal displays in both cases regions with negative values (dark and purple areas). As a result, the opposite dipole contributions lead to destructive interference between excited states and promote plasmon suppression. A similar phenomenon can be seen also in the Au nanoalloys.

The ICM-OS plots shown in Figure S6 correspond to the peak indicated by the central arrow in the spectrum for each NP in Figure 6. The plots are similar to the ones in Figure 8, but the intensity of the off-diagonal spots is lower even though the Ag–Ag or Au–Au contribution is higher in each case according to the fragment analysis. This happens because the collective behavior decreases with increasing the excitation energy. Indeed, all of the peaks selected in Figure S6 are ∼0.7 eV higher in energy than the corresponding peaks in Figure 8.

The effect described above is even more pronounced in Figure S7, where the excitation energy is even higher. In each case, almost whole intensity is carried by single spots on the diagonal, which rules out the presence of a plasmon even if the main contribution to the oscillator strength is given by the Ag–Ag or Au–Au fragments.

## 4 Conclusions

The present DFT calculations of bimetallic nanoparticles composed of coinage metals Ag and Au with platinum-group metals Pd and Pt allow drawing the following conclusions concerning low-energy structural arrangements of the particles:

In all studied 201- and 405-atom truncated-octahedral model particles with high contents of the Ag and Au, these coinage metals are strongly driven to the surface and form there a monometallic skin. The remaining atoms of these two metals in the core of the particles can be arranged in several similarly energetically stable chemical orderings. Their patterns range from inner metal atoms of two types mixed (alloyed) or forming monometallic domains to peculiar L1_1_ or L1_0_ orderings with alternating monometallic layers in (111) or (100) planes of the fcc crystallites, respectively.

More specifically, according to DFT energies inner-layered structures L1_0_ and L1_1_ are more stable than the LEH orderings for 201-atom AgPd particles. However, for particles with 405 atoms, the L1_0_ arrangement is less stable and the L1_1_ arrangement is just slightly more stable than the LEH one. The latter result is consistent with the observed and rationalized reverse size-dependent stabilization of the ordered inner-phase L1_1_ in AgPt nanoparticles up to a critical size of ca. 2.5 nm.^16,17^ Notably, the calculated stabilization of inner-layered particles with respect to nonlayered ones was several times stronger for AgPt than for AgPd,^17^ indicating significantly decreased propensity of AgPd particles to form the peculiar inner-part ordering L1_1_. Quite surprisingly, DFT calculations of AgPd nanoparticles with quasi-perfect qL1_1_ orderings generated by swapping positions of one and two Ag–Pd pairs of atoms in the corresponding perfect L1_1_ orderings revealed nonnegligible stabilization of the qL1_1_ patterns, suggesting that the creation of such small imperfectness in the inner L1_1_ phases should be quite common for AgPd nanoparticles. On the other hand, similar structural distortions qL1_1_ examined for AgPt nanoparticles showed no stabilization with respect to the perfect L1_1_ ordering. The DFT calculations of AuPt and AuPd nanoparticles showed that the formation of inner-layered structures L1_0_ and L1_1_ in them is disfavored with respect to the low-energy homotops identified by the TOP method.

To summarize the first part of the present study, the DFT results obtained for bimetallic particles combining coinage metals Ag and Au with Pt and Pd suggest that the appearance of peculiar ordered core structures is not a general feature of these nanoalloys. It is likely characteristic solely to the nanoalloys of Ag, where the inner L1_1_ ordering has been already experimentally observed in rather small AgPt nanoparticles and it is predicted to be slightly stabilized in AgPd ones. At variance, the substantial destabilization calculated for the inner-layered structures of the Au-containing nanoparticles indicates that such structures cannot be observed at equilibrium.

Subsequently, optical properties of the DFT-optimized structures of the nanoalloy models were calculated at the TDDFT level of theory. Analysis of their optical properties showed that both the electronic structure and the excitation spectra only weakly depend on the chemical ordering of their inner atoms but notably depend on the chemical composition. Indeed, quite different profiles of the optical spectra and PDOS plots of nanoparticles composed of different combinations of the metals were found. However, the fragment decomposition analysis showed common features in the evolution of different spectra and the ICM-OS analysis allowed us to identify that some relevant peaks follow the same trends for each system. At low excitation energies, the main contribution is generally due to electronic transitions from Pd or Pt to the coinage metal, but as the excitation energy increases the Ag–Ag or Au–Au fragments play a major role.

In addition, the ICM-OS analysis carried out for the first relevant peak of each spectrum highlighted the same curious behavior: a plasmonic nature suppressed by opposite dipole contributions. Therefore, the plasmon-quenching effect by the platinum-group metals previously calculated for AgPt nanoparticles^17^ is not only specific for this combination of metals but also common for the other studied chemical compositions.

## Supporting Information Available

PBEsol vs PBE results; sketches of quasi-perfect layered (qL1_1_) structures of AgPd NPs; PDOS plots of Ag_158_Pd_43_ NPs; optical spectra of Ag_158_Pd_43_ and Ag_161_Pd_40_ NPs; ICM-OS plots of Ag_158_Pd_43_, Ag_158_Pt_43_, Au_158_Pd_43_, and Au_158_Pt_43_ NPs; atomic coordinates of the modeled NPs (PDF)
