# Supplementary material for: AgPd, AuPd, and AuPt Nanoalloys with Ag- or Au-Rich Compositions: Modeling Chemical Ordering and Optical Properties
Source: J Phys Chem C Nanomater Interfaces. 2021 Jul 30;125(31):17372–84. doi: 10.1021/acs.jpcc.1c04222 (PMC8397350; doi:10.1021/acs.jpcc.1c04222)
Supplement: Supplementary file 1 — jp1c04222_si_001.pdf [file jp1c04222_si_001.pdf]

Supporting Information

AgPd, AuPd and AuPt nanoalloys with Ag- or Au-rich compositions: Modeling chemical ordering and optical properties

Nicola Danielis,[1] Lorena Vega,[2] Giovanna Fronzoni,[1] Mauro Stener,[1]* Albert Bruix,[2] and Konstantin M. Neyman[2,3]*

[1] Dipartimento di Scienze Chimiche e Farmaceutiche, Università di Trieste, via L. Giorgieri 1, I-34127, Trieste, Italy
[2] Departament de Ciència del Materials i Química Física & Institut de Química Teòrica i Computacional, Universitat de Barcelona, 08028 Barcelona, Spain
[3] ICREA (Institució Catalana de Recerca i Estudis Avançats), 08010 Barcelona, Spain

Table S1. Mean values of the bond lengths Ax-Ax, Ax-Px and Px-Px (in pm) in 201-atomic nanoparticles (NPs) AxPx (Ax = Ag, Au; Px = Pd, Pt) calculated using PBE and PBEsol xc-functionals.

Particle	Homotop	Ax-Ax			Ax-Px			Px-Px		
		PBE	PBEsol	diff[a]	PBE	PBEsol	diff[a]	PBE	PBEsol	diff[a]
$Ag_{158}Pd_{43}$	$L1_1$	287.5	281.5	6.1	287.2	281.7	5.5	286.3	280.6	5.7
$Au_{158}Pd_{43}$	$L1_1$	286.9	281.7	5.2	288.0	283.0	5.0	286.4	281.1	5.3
$Au_{158}Pt_{43}$	$L1_1$	287.1	282.0	5.1	291.5	286.5	5.1	281.2	277.3	3.9

[a] diff = bond_lenght(PBE) - bond_lenght(PBEsol)

Table S2. Topological energy energy descriptors ε (meV) calculated by fitting PBE and PBEsol energies of 20 homotops of 201-atomic AxPx particles.

Particle	xc-functional	$\varepsilon_{\mathrm{BOND}}^{\mathrm{Ax-Px}}$	$\varepsilon_{\mathrm{CORNER}}^{\mathrm{Ax}}$	$\varepsilon_{\mathrm{EDGE}}^{\mathrm{Ax}}$	$\varepsilon_{\mathrm{TERRACE}}^{\mathrm{Ax}}$
$Ag_{158}Pd_{43}$	PBE	-11	-387	-406	-107
	PBEsol	-11	-433	-431	-119
$Au_{158}Pd_{43}$	PBE	-29	-403	-419	-216
	PBEsol	-31	-410	-420	-236
$Au_{158}Pt_{43}$	PBE	+14	-574	-533	-280
	PBEsol	+13	-622	-548	-310

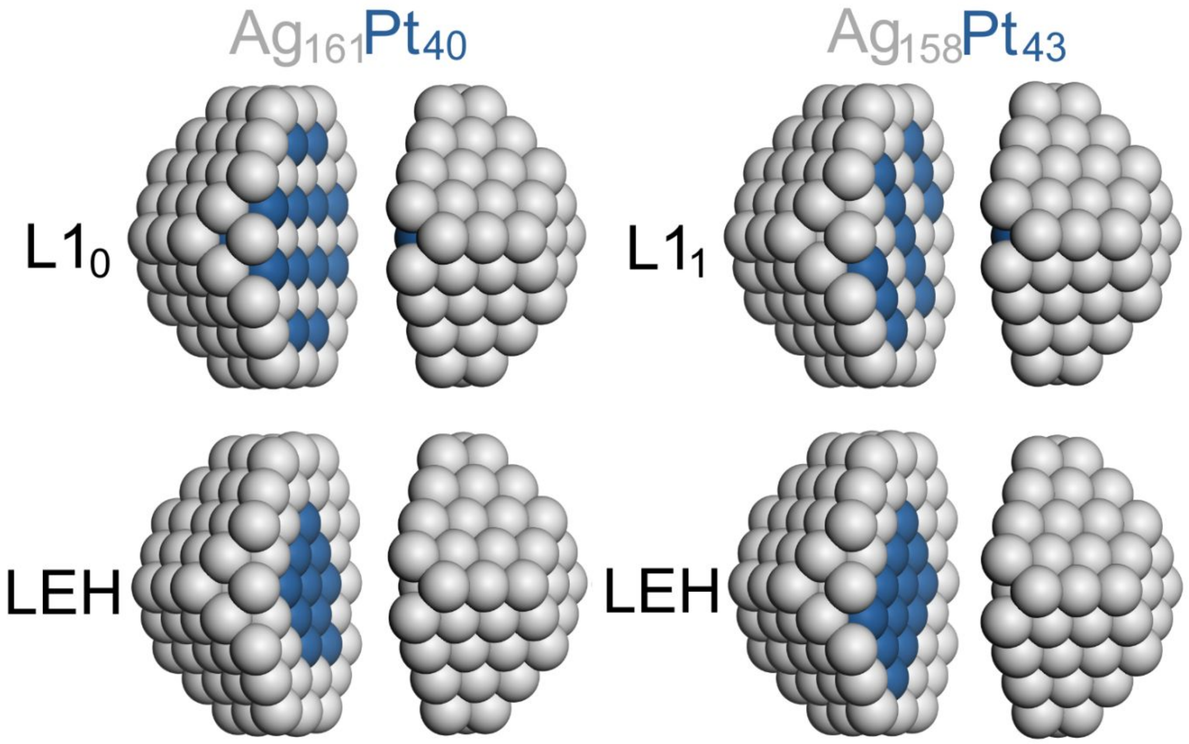

Figure S1. Sketches of 201-atomic AgPt NPs with different atomic orderings. The structures are calculated in *J. Phys. Chem. C* **2019**, *123*, 25482; doi: 10.1021/acs.jpcc.9b07382. The images are split to show inner parts of the NPs. Ag atoms – grey; Pt atoms – dark blue.

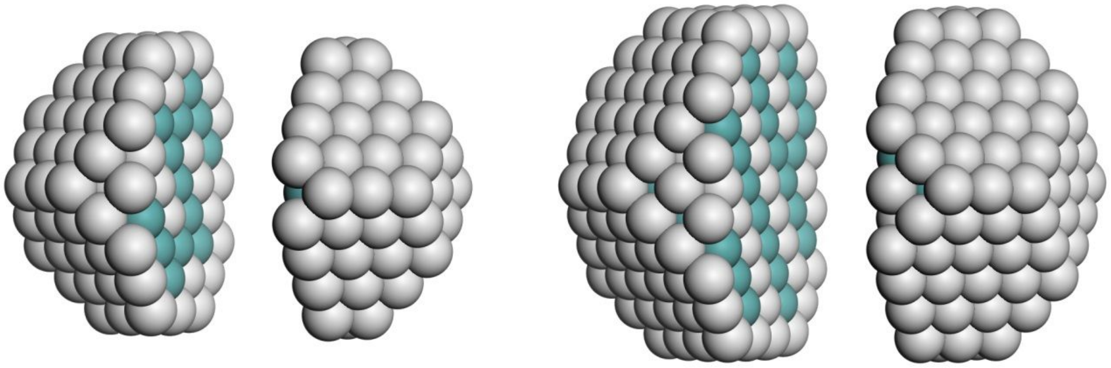

Figure S2. Quasi-perfect inner-atoms layered orderings $qL1_1$ of $Ag_{158}Pd_{43}$ (left panel) and $Ag_{295}Pd_{110}$ (right panel) nanoparticles. Ag atoms – grey; Pd atoms – blue.

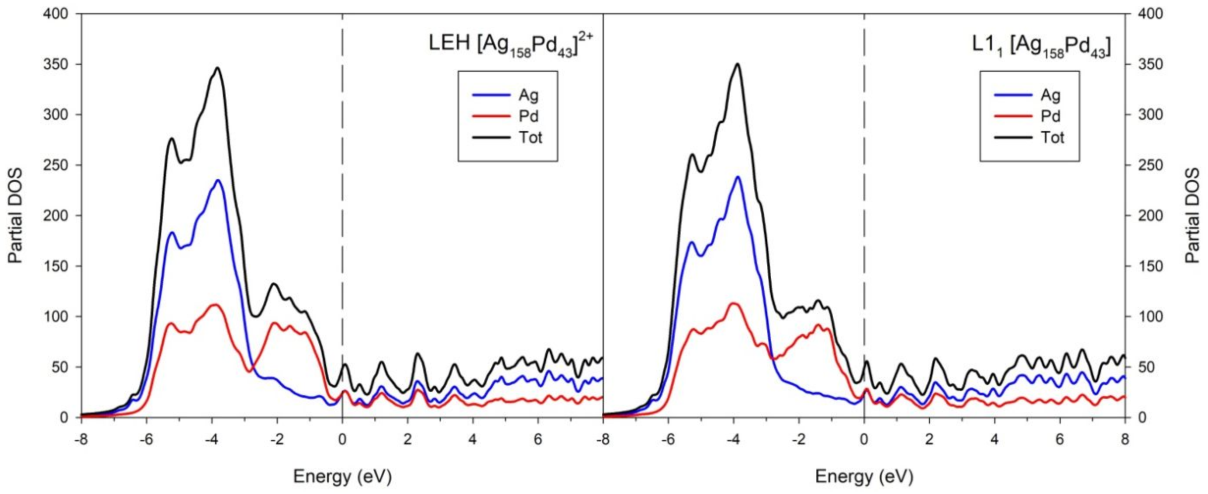

Figure S3. Comparison between the PDOS plots of $[Ag_{158}Pd_{43}]^{q}$ NPs with different chemical orderings

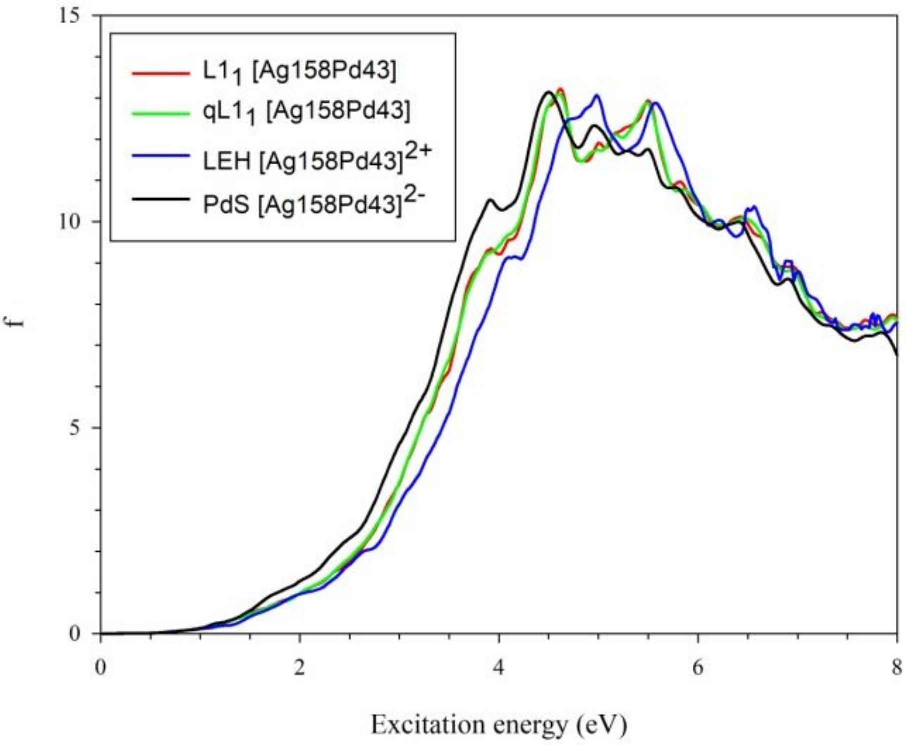

Figure S4. Comparison between optical spectra of the closed-shell $Ag_{158}Pd_{43}$ NPs with different chemical orderings

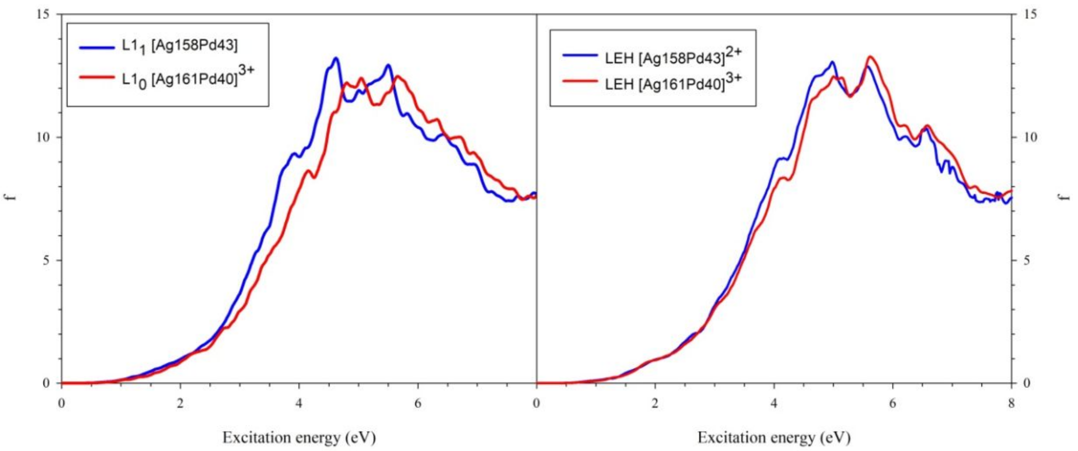

Figure S5. Comparison between the optical spectra of the closed-shell $Ag_{158}Pd_{43}$ and $Ag_{161}Pd_{40}$ NPs with different chemical orderings

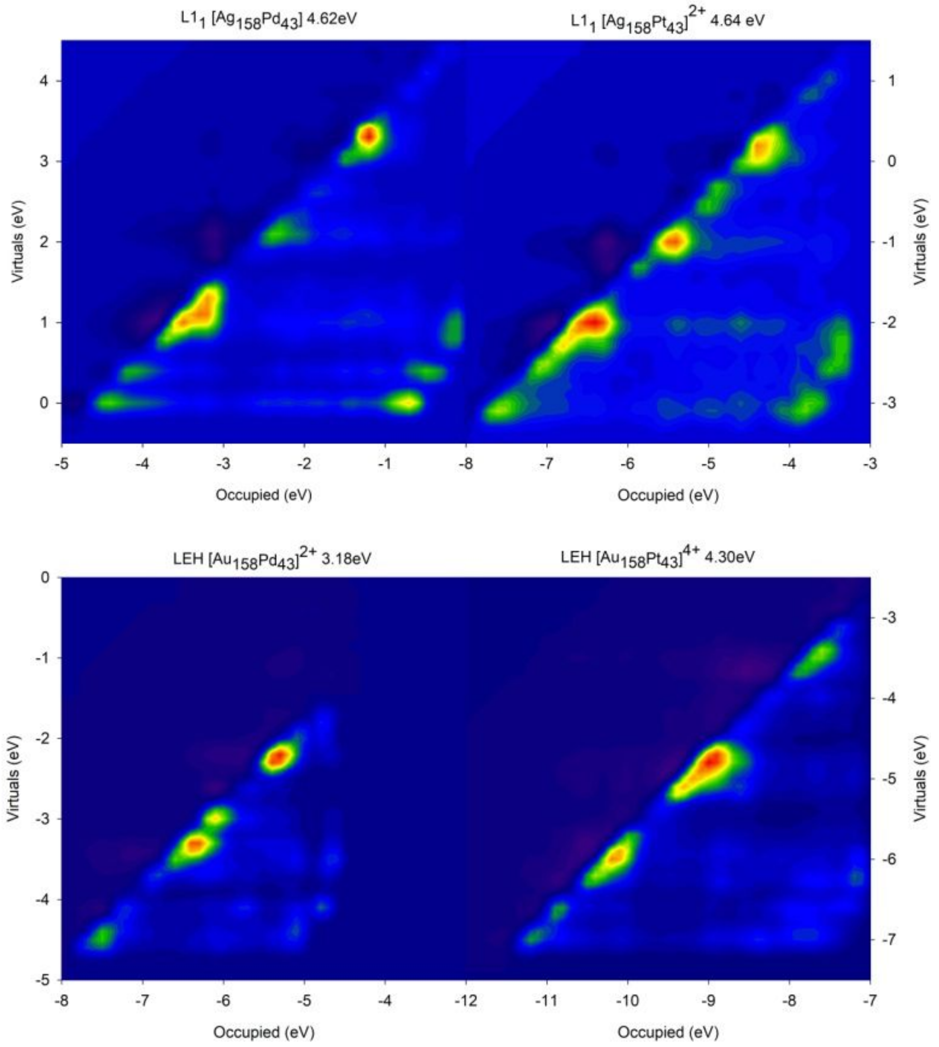

Figure S6. Second set of ICM-OS plots of the closed-shell $Ag_{158}Pd_{43}$, $[Ag_{158}Pt_{43}]^{2+}$, $[Au_{158}Pd_{43}]^{2+}$ and $[Au_{158}Pt_{43}]^{4+}$ NPs, corresponding to the peak position indicated by the central arrow for each color in Figure 6. The excitonic energy of the analyzed peak is given above each plot .

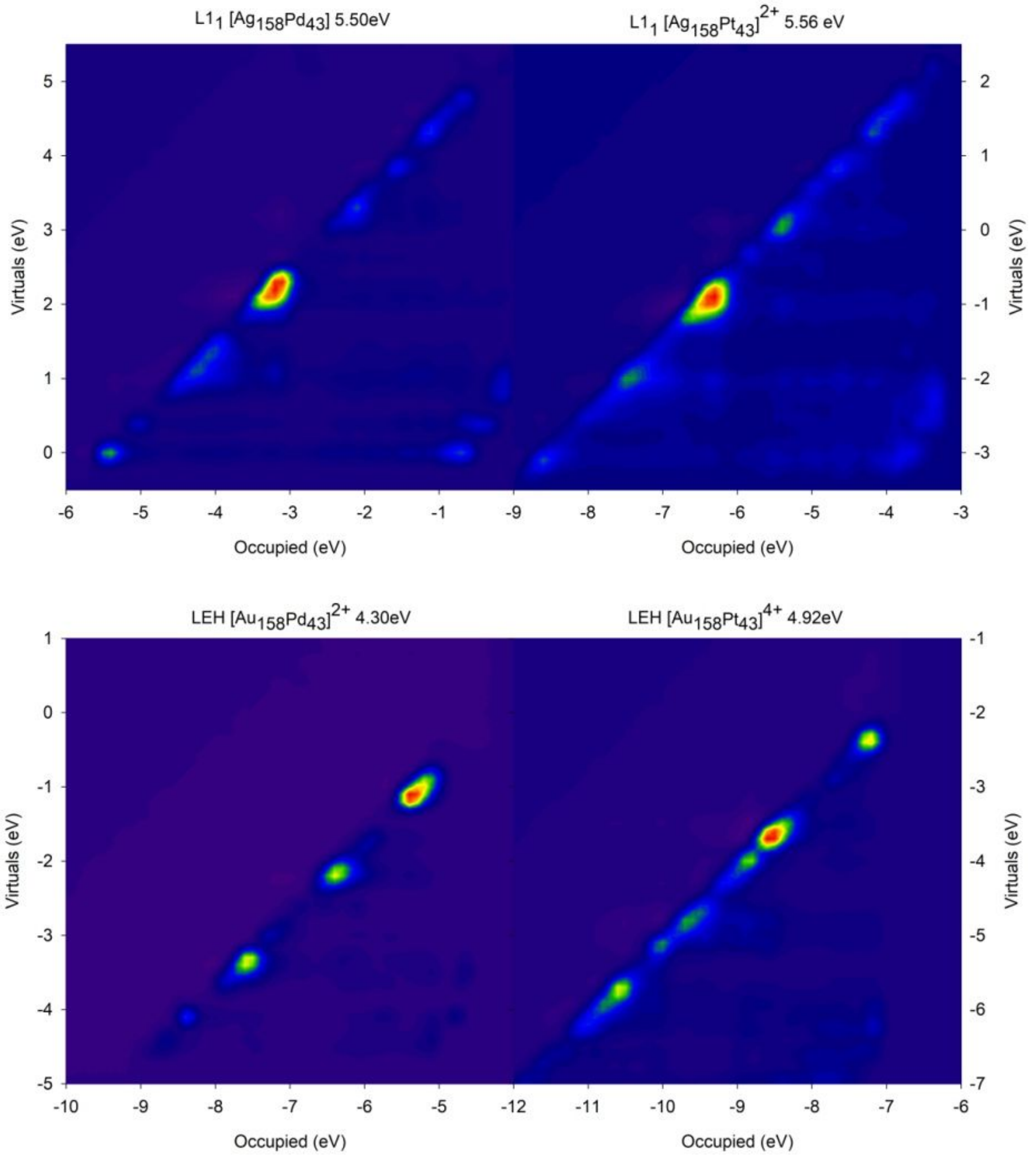

Figure S7. Third set of ICM-OS plots of the closed-shell $Ag_{158}Pd_{43}$, $Ag_{158}Pt_{43}$, $Au_{158}Pd_{43}$ and $Au_{158}Pt_{43}$ NPs, corresponding to the peak position indicated by the right-most arrow for each color in Figure 6. The excitonic energy of the analyzed peak is given above each plot.

DFT/PBE atomic coordinates (in Å) in the homotops of the AxPx (Ax = Au, Ag; Px = Pd, Pt) nanoparticles presented in Table 2

$Pd_{40}Ag_{161}L1_0$-606.998

Ag	14.51180935664417	14.51191806331107	4.40121720775623
Ag	12.50000728695835	6.40395934079597	18.57233001245677
Ag	8.42229584355976	10.44658246474951	18.59022210979660
Ag	12.49997529226201	4.39420940932568	16.54134951975988
Ag	20.60537066652045	12.50013101789234	16.54135042377021
Ag	10.48802359965782	14.51186725884602	20.59882402577399
Ag	10.44675644556651	18.61723419846138	16.55160334149416
Ag	20.66640276682324	14.51833087275328	14.51607789104139
Ag	20.66643248769578	10.48179104847789	14.51617436129113
Ag	10.48827309160322	14.51188324947584	4.40113920372259
Ag	10.44665401145130	8.42229631451465	18.59005214292345
Ag	4.33360165397038	14.51821596056526	14.51593009860748
Ag	14.55313492523030	6.38268507849326	16.55185421757911
Ag	6.38282513279885	10.44682937468835	16.55187384574214
Ag	12.50000236474587	6.40398646392043	6.42758625583631
Ag	14.55333637363153	16.57778000992306	18.58998577118878
Ag	14.51824588889090	4.33358698428939	10.48426581468381
Ag	6.35755618100231	8.39845509461185	14.54928952591431
Ag	14.55315540287967	6.38276870518917	8.44841830883194
Ag	10.48161322666963	20.66642277813965	10.48392372267249
Ag	8.39836017790793	6.35760797663685	10.45090122219860
Ag	10.44668638516893	16.57765114809558	6.40979574340402
Ag	18.64244922920278	8.39838452556785	14.54933755654224
Ag	14.51826719749399	20.66640263615977	14.51587493366769
Ag	6.38297859199348	14.55325235028962	8.44801012376314
Ag	6.36889570719435	6.36900761397054	12.50000854232691
Ag	8.39837627912906	18.64235830840815	14.54910177525922
Ag	18.63100625851481	6.36901065960735	12.50022712632067
Ag	6.40416774481271	12.49996686898381	6.42722987561578
Ag	18.64235576311787	16.60171267920142	14.54918656978656
Ag	16.57325046819157	8.42678515356841	16.53593848646930
Ag	6.38282313724913	10.44677385375506	8.44806585019092
Ag	6.35761214126972	16.60155947584958	10.45062676532108
Ag	16.57318246591253	16.57345647734490	16.53580532139391
Ag	14.55316247695520	18.61729367342663	16.55169348669020
Ag	16.60166716978596	6.35761311932336	10.45099636381835
Ag	12.49987422450542	4.27941802997733	12.50017346039568
Ag	10.48174183488536	20.66640683796878	14.51591035377129
Ag	14.51837141729215	4.33355800720260	14.51599774848532
Ag	12.50008737736760	12.49993354623718	4.37805300180953
Ag	8.39836400223201	18.64242453852798	10.45077194733785
Ag	10.44677304238884	6.38284002210996	8.44833605055438
Ag	12.49992770431861	20.72047056409293	12.49986343751357
Ag	18.64236566453204	8.39835588058676	10.45089753385339
Ag	10.44662818447608	8.42234157504032	6.41010451439527
Ag	16.60167137545939	6.35746043659129	14.54925442703209
Ag	14.51201202660559	10.48801981322823	4.40122006551760
Ag	14.55339155742386	8.42224291131044	6.41011677204234
Ag	18.63107233370780	18.63117171021839	12.49988851899174
Ag	6.35758193410600	16.60147653636832	14.54912199074871
Ag	16.60165254180676	18.64245244476897	14.54903073570501
Ag	18.61708240215082	14.55320138056667	8.44813260386218
Ag	10.48826339359891	10.48801284713444	20.59886602221422
Ag	6.35768034972875	8.39842025707168	10.45071051517197
Ag	8.42673209260401	8.42679364928556	16.53593264905647
Ag	18.64235161088142	16.60162803828519	10.45069113938896

Ag	18.59580900887617	12.50009129905448	18.57273897481734
Ag	14.51204506549199	14.51196165531385	20.59883480797139
Ag	8.42234245456250	10.44660410822697	6.40973141218461
Ag	14.51173672386025	10.48805947596050	20.59882109434546
Ag	4.33358237954266	14.51828655853444	10.48373236799542
Ag	16.60157403732088	18.64246452978900	10.45080009741372
Ag	4.33355576356892	10.48171672802507	10.48389642253279
Ag	12.49998537365515	18.59607866039709	18.57251141602412
Ag	16.57773085707982	10.44654585828281	6.40982754983958
Ag	8.42235658554735	14.55336025708162	18.59028823438681
Ag	10.44659226751073	16.57769804107791	18.59000070828124
Ag	20.66638204338240	10.48169184790324	10.48408842362834
Ag	4.27959211047264	12.49998516783439	12.49979057123972
Ag	14.55319778392934	18.61709943553170	8.44821767637343
Ag	16.57766498366259	14.55339704915510	6.40977579588556
Ag	6.36898647645855	18.63110002280034	12.49982944577731
Ag	6.40410713636038	12.49996339461788	18.57270728603041
Ag	18.61706910184486	10.44693647638770	16.55189462490178
Ag	8.39828468091946	6.35759884714723	14.54910131263620
Ag	4.33351819845838	10.48178003977310	14.51601538133452
Ag	8.42668946634541	8.42654138395449	8.46414100047062
Ag	10.48165734296446	4.33360684590138	10.48415594408294
Ag	20.72036333908606	12.50001168759873	12.50009801606014
Ag	16.57316101619996	16.57325533935257	8.46403140514214
Ag	20.66639850296973	14.51830165402109	10.48393606142809
Ag	16.57340037732878	8.42649253545077	8.46423170763229
Ag	18.59580604544908	12.49989762787082	6.42733024636911
Ag	18.61724544822510	10.44670632058827	8.44815327944115
Ag	10.48158913445490	4.33348346662504	14.51603004613161
Ag	18.61732089643792	14.55329495262248	16.55196163908837
Ag	6.38281460401430	14.55322096024590	16.55183610538681
Ag	8.42676111322042	16.57324812140067	8.46402386624023
Ag	16.57765439238977	10.44659484872822	18.59011670180413
Ag	14.51835622505575	20.66638637726348	10.48390203472519
Ag	8.42237531169006	14.55338979290993	6.40971764057811
Ag	14.55337249661822	16.57762869903029	6.40985486686076
Ag	14.55346495367597	8.42233389470423	18.58999456874416
Ag	10.44680418501634	6.38285613200530	16.55180843541648
Ag	12.50003176460787	12.49998082119937	20.62195622264192
Ag	16.57771011143447	14.55349433621160	18.59028996122572
Ag	10.48805470733140	10.48809431832026	4.40126743677190
Ag	10.44681258834674	18.61722575446770	8.44816472185658
Ag	8.42653895540415	16.57346999022856	16.53579616774691
Ag	12.49997331934120	18.59590929177547	6.42741941649659
Ag	16.52395657506080	12.50007248189816	20.55270053668289
Ag	12.50004213612572	8.47557378479352	4.44773991173363
Ag	16.52396694688052	12.49985951614196	4.44735033900248
Ag	6.37153788047973	10.48542325703592	12.49994551591639
Ag	12.49975401660405	16.58804323137257	12.49986279882302
Ag	12.50008580897490	8.47564798649778	20.55243086536122
Ag	20.63293328182388	16.58050839501247	12.49997701090161
Ag	12.49995369473335	20.60545282207836	8.45870539262640
Ag	18.62836647350260	14.51477749610400	12.50000059650629
Ag	12.49991010423737	16.52441943518658	20.55241965344061
Ag	12.49997841895087	4.39434949549702	8.45881317720464
Ag	16.57182904426899	16.57161877362645	12.49999406896152
Ag	10.48078075476660	14.51943765142520	16.51567270841995
Ag	8.43243779666435	12.49991006366331	16.49447438808805
Ag	16.58047863159208	20.63326085472945	12.49983429669297
Ag	20.60522932291873	12.49993569202250	8.45885868879251
Ag	4.39462402742868	12.50003586783590	16.54121504194673
Ag	10.45384911184279	10.45426284322684	12.50024731159397

Ag	8.41951646243516	20.63320536854632	12.49983433190437
Ag	12.49984222776744	12.50032231859864	16.53038293826826
Ag	10.48537091016595	6.37145425634555	12.50012920519971
Ag	12.50012131640875	12.49989007474978	12.50012883130430
Ag	14.54629495395311	14.54593416876396	12.50007352385569
Ag	12.50006049270030	8.41196396650309	12.50021097425953
Ag	4.36704031605265	16.58046275473274	12.49983378846273
Ag	14.54604491142671	10.45421908799499	12.50007246577153
Ag	14.51923716874258	10.48067873225360	8.48452743594715
Ag	14.51461509949580	6.37156488631148	12.50017078294862
Ag	12.50000085860236	12.49971653211755	8.46967731427829
Ag	10.48535102957877	18.62844745578397	12.49994189986605
Ag	12.49996010964665	8.43244940550675	16.49440340945397
Ag	12.50006341571674	16.52415163295919	4.44740404033536
Ag	12.49990390513925	20.60571087905228	16.54108298519023
Ag	16.58761142164034	12.50017382945898	12.50004889216718
Ag	8.47600494396344	12.50001950775531	20.55281105035350
Ag	16.56756443649027	12.49999907919810	8.50562846994153
Ag	12.50005872072364	16.56731890174915	16.49424561803337
Ag	16.56757644736304	12.49989773508035	16.49451708965409
Ag	10.45386709774027	14.54615303513340	12.49987022847193
Ag	8.43254120533062	12.50011008439912	8.50558151113396
Ag	10.48045289894347	10.48074710605538	16.51556756441116
Ag	10.48051786458302	14.51934470315427	8.48445194861350
Ag	6.37143588854956	14.51468965632339	12.49992842812540
Ag	4.39474412078615	12.49989337391413	8.45867448894997
Ag	12.50006426774319	16.56741151803135	8.50572487959533
Ag	14.51917695846366	14.51963416443719	16.51556912134400
Ag	16.58062339164775	4.36675320148919	12.50020274908385
Ag	14.51459214522483	18.62868716296588	12.49988326640916
Ag	14.51957053663017	10.48060290415286	16.51575603716478
Ag	18.62856028668947	10.48532557643827	12.50010082833009
Ag	16.57177378276743	8.42833749646899	12.50014603231906
Ag	8.41942364182044	4.36681442785291	12.50016548521860
Ag	10.48086637357575	10.48072071058822	8.48441707938350
Ag	8.42834076893677	16.57153131628290	12.50000523297194
Ag	8.42822470877065	8.42841662499052	12.50005862004396
Ag	14.51954250340945	14.51952326831480	8.48432241331546
Ag	20.63299029242108	8.41950721786234	12.50020597059902
Ag	12.49997712124308	8.43267792779224	8.50582991514547
Ag	8.47605681922160	12.49995167406363	4.44725019253243
Ag	8.41212581293120	12.49994187259385	12.49991787174656
Ag	4.36695241327004	8.41956860934804	12.49998556961076
Pd	14.53878367286364	12.50010890208310	14.50299839963234
Pd	16.58600724868985	14.55047181070096	10.47242122963653
Pd	18.66279948482058	12.49998570184255	10.46344156842824
Pd	8.41398585468578	10.44965660025531	14.52755200854861
Pd	6.33722619848003	12.49990639821591	10.46334397652133
Pd	12.49996938682800	14.52526000730352	18.60192246713518
Pd	14.52525273239013	12.49995259683983	6.39781095408424
Pd	14.52532504901888	12.50001588935348	18.60223067277862
Pd	12.49988601009560	10.46146976872484	14.50300774472341
Pd	12.49993661030345	6.33722846582750	10.46380676749998
Pd	12.50004996691580	14.53883103835790	14.50332063822261
Pd	10.44963364815401	16.58588058218031	10.47244949408689
Pd	18.66286223730922	12.50007113190062	14.53666998397922
Pd	10.44944172047135	16.58608684286951	14.52734513432792
Pd	10.46114839136448	12.49990642327768	14.50313807314052
Pd	10.47473114151483	12.50003521651258	18.60217756790295
Pd	14.55045035345484	16.58604951908936	10.47226910283852
Pd	6.33715011938681	12.50004724892158	14.53651505677837
Pd	14.55043234354585	8.41393451208707	14.52758021149554

Pd	12.49999319224589	18.66274436270713	10.46351750518536
Pd	14.55044474121424	8.41399808683003	10.47261462468616
Pd	10.44953384149204	8.41396017532285	10.47256141733033
Pd	12.50008548090483	14.52529623047269	6.39771674362223
Pd	12.49994789182550	14.53886479452435	10.49668132474929
Pd	16.58604208420159	14.55059217775068	14.52736261559077
Pd	12.49992956508256	18.66289657618679	14.53629814132881
Pd	12.50001138554247	10.47465123466142	18.60226916893636
Pd	16.58603476477584	10.44966836138405	14.52771075600376
Pd	14.53894671945943	12.49985248523566	10.49696446466751
Pd	10.44952356064923	8.41406252994822	14.52754920038840
Pd	8.41374126363811	14.55050087236285	14.52740959564227
Pd	12.49998860202491	10.47482946789767	6.39813889173051
Pd	10.47481381734418	12.49993578553337	6.39777140129445
Pd	12.50012683597317	10.46132929369530	10.49680145601305
Pd	12.49998711695618	6.33726855646791	14.53650506396481
Pd	8.41384770110194	10.44963759435010	10.47256001183176
Pd	8.41404107873067	14.55043506831002	10.47223762019503
Pd	16.58609295209687	10.44960790329224	10.47254461274852
Pd	14.55055466442995	16.58609340958003	14.52755405681697
Pd	10.46113390180209	12.50001868232334	10.49673536370124

$Pd_{40}Ag_{161}$LEH-606.586

Pd	8.47495725194395	12.58416397684666	16.66501210817239
Pd	14.58310776358015	12.60263003676233	14.64868562288645
Pd	10.49213970893531	16.69259223825999	14.63848915639302
Pd	12.52856750834918	8.50132426684119	8.53546203375618
Pd	18.65856583622162	12.58277674798770	14.61808839964610
Pd	8.47884680222667	12.58103015220892	8.56276835841693
Pd	10.49392590873357	16.69629934413355	10.55187736454310
Pd	14.58182217039687	16.69902609725166	10.55169825118150
Pd	8.47764990018812	8.49165303166922	12.59172913013810
Pd	14.58466539191872	8.51095690160479	14.64814902542970
Pd	14.55755274633955	12.57929730286503	18.76133503751124
Pd	10.50813972945972	12.58044817364860	6.49198965818971
Pd	10.50416068385296	12.60123226890959	10.57959607507058
Pd	12.53250810869395	14.63267055344384	18.77353380719212
Pd	6.42170179513771	12.58219507576975	14.62194590102084
Pd	6.40767920930688	10.53486048994500	12.59274792887635
Pd	12.52835372022242	6.42567506687655	14.67195119473722
Pd	18.66272408605067	12.58390837075091	10.56686586262173
Pd	14.57996995856964	12.59099651556867	10.57605527610185
Pd	18.67740687996171	10.54013309492936	12.58500787606608
Pd	14.58231149315360	16.69751511504530	14.63513594654900
Pd	6.41956939017859	12.58062527382261	10.57754266634266
Pd	12.52909574997130	18.78134365186078	14.64645671049601
Pd	16.59530242195965	12.58611381838819	8.55160806928543
Pd	6.40835890004866	14.63500174922090	12.59234878208925
Pd	10.50246191815424	12.60083781976713	14.64802559462521
Pd	10.51424556535203	8.51705364709014	10.55973122129793
Pd	12.52867934214910	10.53685182823953	6.46612196185323
Pd	16.59713097336398	12.58805740377228	16.66856189688075
Pd	10.48716157566739	8.50972711887695	14.65653533269130
Pd	10.50603681775644	12.57569836011084	18.75578843539425
Pd	12.53138613270585	18.79585583138186	10.52093302636970
Pd	8.47347206715183	16.71112276722927	12.58762157290567
Pd	12.51775980667961	6.43401483539521	10.53639819041015
Pd	14.55739546870482	12.58559572120468	6.48339986670828
Pd	12.53109607280818	14.64482319687990	6.46661873807214
Pd	18.67187364532136	14.63913910317866	12.58909348677002
Pd	16.60115364174997	16.71685085964952	12.58328714886909
Pd	12.53049049515138	10.53382958463146	18.78380576170416

Pd	16.60809612617222	8.49300787495396	12.59796086899128
Ag	20.59940558824886	8.50142571219970	12.57896453797295
Ag	16.53433365190750	12.57441880538521	4.52273759688728
Ag	10.51302560097979	4.40712129548882	10.57989524342280
Ag	10.50603971875825	10.57107584963918	16.68757301267641
Ag	12.53989779506463	12.59313525970938	16.74832396048074
Ag	18.62674507874305	16.69255424340336	14.61568745097757
Ag	12.54403397807132	14.64626464111831	14.64427496768400
Ag	10.48286505550152	18.75655521825877	8.50917313753707
Ag	10.49646115312829	14.66862436649039	12.60545142866720
Ag	10.51533336366184	18.74596571786999	12.57912464254212
Ag	18.62681430847836	14.62684857736274	16.65561135919533
Ag	10.51403535296816	6.46881291207449	12.59465362622677
Ag	14.57139087787445	6.44609703159300	16.67233619736714
Ag	12.53114091893029	10.55540262750046	10.57157431950169
Ag	16.57949840716729	20.80578253655551	12.56730714572979
Ag	8.46745882402749	8.47958257321248	8.52814588865044
Ag	10.50135342032830	6.47480348669618	8.54583024252629
Ag	14.54904861716286	6.45695411443435	12.61312295306479
Ag	16.59272085414986	8.47262862566465	16.67896118291866
Ag	12.52507651268629	18.76972305716335	6.45820789458419
Ag	16.65104966359714	12.59599644503279	12.60040385023435
Ag	14.56790469915220	10.57195809420995	16.68994448697822
Ag	10.48434768501883	18.74888570750786	16.65819890544337
Ag	16.58609285739525	14.62350003541662	18.71344165433540
Ag	14.56817153870596	8.48923349708484	6.50127378630471
Ag	18.59998951148306	12.57894059036982	6.53712726561491
Ag	20.62269271249382	12.57554191357678	8.58564178052196
Ag	12.52430027323451	4.45343930069609	16.66313559480750
Ag	10.48098017251921	16.70279559299828	18.72895825776316
Ag	16.59371789233927	16.71171040543572	16.67268581005002
Ag	18.60512066956675	6.43655071765509	12.58563603487664
Ag	8.46721844396210	18.73838020010816	14.61009669442553
Ag	8.47024332066377	10.53666633404549	6.51822405071788
Ag	8.46852839773916	10.53224956551436	18.70988026282831
Ag	12.53489811721889	8.50039407747731	16.66093223355174
Ag	12.52296929621510	4.39650162101185	12.58683795967719
Ag	14.53565348854533	10.54487742953085	20.75415636479233
Ag	10.52101005287364	20.79180182244863	14.58747668998707
Ag	6.44109880491933	16.68589139408899	14.61635606479320
Ag	12.53906239024007	12.59685516909411	8.49269795800600
Ag	8.47305344151311	14.62680993599169	14.62161558590829
Ag	12.52340465211710	8.49009377293969	20.71114721227531
Ag	20.64707718364586	10.55004949527087	14.59586959522378
Ag	6.43540577452027	10.53124728607175	8.55050575114845
Ag	12.54067688672062	10.56300351710410	14.65692632323160
Ag	6.43734731217116	14.62178409713816	8.54730120975159
Ag	10.50838500463650	14.61154518351312	16.68544971657956
Ag	16.60758102100711	10.56166954486557	10.57391172061612
Ag	4.46953857163049	8.49956136169898	12.58444441433156
Ag	12.52702679025348	12.57554051133006	20.76997382530756
Ag	10.52282528794781	4.41624585467381	14.59986279100107
Ag	12.52025193188139	6.45989172235325	6.50573957580631
Ag	18.62499546185047	8.48322004160756	10.54355311611091
Ag	16.60641688994609	14.63192733813337	14.62188295427272
Ag	16.58755901533989	14.62763103017674	6.50172888773272
Ag	8.47058822721437	20.79442012063849	12.56885906201243
Ag	12.53503184668950	16.70845739813999	8.55718811235531
Ag	6.44021944820001	16.68584065773560	10.54974287619279
Ag	14.52116324225675	4.40243684845735	14.60944284678795
Ag	10.51762488505550	14.60019321964308	20.73337798079500
Ag	14.54976751449142	18.75068857940288	12.57757316909894

Ag	14.53100448202171	14.60693076560812	4.49515303830022
Ag	8.47184112831883	14.61795571705010	18.70353003544797
Ag	8.47559202671345	14.62878836726460	10.58096911216913
Ag	4.44780455571842	12.57397030553612	16.59519866300014
Ag	10.47740785905932	8.47125277585776	18.74169787327560
Ag	8.47185414902920	14.61664355452418	6.52020259581355
Ag	14.53098640281959	10.55579036749958	4.49155272906217
Ag	10.48389340157858	6.44588824274875	16.67445430588083
Ag	16.59301970863799	18.74502911769249	14.61136129995717
Ag	8.47410043900836	10.55708289848945	14.62554004293660
Ag	16.60943371483532	10.56026626083216	14.62786640479278
Ag	12.53593138445625	16.69893622616994	16.65000929962369
Ag	18.60827361540570	18.75651900968832	12.57504373050989
Ag	8.43557513402951	12.59327153413679	12.60493412777183
Ag	8.50594159221417	12.56836183751691	20.70316298006651
Ag	12.52453939235021	16.66899542969403	20.69414018530954
Ag	16.59257681179515	6.45024118856734	14.63005187313085
Ag	4.43827677759967	12.57097644572058	8.60892371054760
Ag	10.51858109833792	10.56238447791017	8.54777457892072
Ag	14.57721414440783	16.70753549057727	18.73482888650981
Ag	4.42373231040164	10.55244312374613	14.59359256567759
Ag	18.59817320216234	12.57208692561751	18.67266211017839
Ag	14.57877910700094	16.71296850015692	6.47085191677803
Ag	6.44388673847727	8.48787902651991	14.62217573991481
Ag	12.53881306761228	16.75185788197172	12.59368700910967
Ag	10.49807651467228	10.53985319496787	12.61622520744788
Ag	10.48907835412481	8.49447057672157	6.49885465293986
Ag	20.64664590550588	14.61389080288843	10.57270983841464
Ag	12.52615144722665	18.76081665942389	18.72148330233043
Ag	14.58664076242483	10.54965359660504	12.62652099915618
Ag	14.52502533162993	4.42509600854410	10.58142785579681
Ag	12.52240327162831	20.75360213235056	8.50180593969348
Ag	20.64203314407634	14.60787832510458	14.59920051166772
Ag	16.59401655235370	18.75197943080845	10.54164422347015
Ag	12.52012714669896	4.45123256815805	8.54199756173047
Ag	8.51035920835039	12.57564721425389	4.53566445315731
Ag	8.47787438292073	4.40986509759844	12.58053793933585
Ag	14.52629953557903	20.81099602103885	10.56085521878949
Ag	14.58919160495677	14.66844880298555	12.60348726070477
Ag	14.55974906146994	6.45727930069123	8.52294482465454
Ag	14.57321159704101	18.76232509891792	8.50900253356266
Ag	16.59157985037122	10.53434685961904	6.50299638753291
Ag	12.54389082956758	14.64543376586424	10.57524092663541
Ag	18.63105317638984	8.48625097396651	14.62544559663101
Ag	6.44047289631710	8.48588020696955	10.55407493976646
Ag	12.52269999058953	6.42667848047414	18.73257338223307
Ag	12.52478443708796	20.73926555802656	16.64691338987033
Ag	10.51775884315078	14.60074479504094	4.50630119427452
Ag	14.56940139799524	14.61569332792457	16.68505918132064
Ag	10.47926679150901	16.71024768093136	6.47535258290752
Ag	8.46865877782056	6.45620584765110	14.62035406064795
Ag	16.62613030961010	8.45419322016422	8.48889384775228
Ag	14.52512628890724	20.79284541899935	14.58361724095149
Ag	16.56978700159558	4.40390914808876	12.59202018908907
Ag	20.65037228871018	12.58397430661466	12.58590309965993
Ag	18.62937617896859	10.53089521896212	16.65742105764211
Ag	8.48033686063897	10.55371573009512	10.58684503733512
Ag	8.46654859510363	18.74205163926401	10.54373823752385
Ag	14.57398599512670	18.75250068952082	16.65822075998016
Ag	4.42575535589338	14.60541533969297	14.59650195977038
Ag	16.60557056957843	14.63224453357778	10.57451862072235
Ag	18.62811420846992	16.69441858637454	10.54574662397875

Ag	12.52452688317728	20.83928937821973	12.57368177906256
Ag	18.63625065831638	10.53034574797469	8.53188131877344
Ag	4.46986044604514	16.65545789710740	12.57970277525419
Ag	16.58864873364381	10.53442215672401	18.71641910356708
Ag	12.52540683586935	8.46114008554021	12.63735042382533
Ag	14.53071569160251	14.60281624691122	20.74571999439756
Ag	10.50961608065323	14.61767684294773	8.53997539275921
Ag	12.52763286428907	12.58013725081653	4.48805474716815
Ag	6.45190621055134	12.57460253110495	6.55731842273978
Ag	6.45311335071119	6.43938834878170	12.58406975823349
Ag	20.66099536588492	10.54816826271582	10.57668221107558
Ag	6.45010914885610	18.74665028693716	12.57481503752015
Ag	14.57861982723615	8.47137282994351	18.74444044369032
Ag	16.60925645484916	6.43312740428673	10.54149707925893
Ag	6.43774341273702	14.62451916042110	16.64918990213824
Ag	12.54194637780174	12.60320105391834	12.61278680801087
Ag	6.45590533029215	12.56936949376116	18.66233163934055
Ag	16.59224555198045	16.71614988425567	8.51027928013453
Ag	20.60160121601819	16.66355344999818	12.57589762216499
Ag	8.46561952499282	8.47438924811624	16.68014419114711
Ag	18.62924092724752	14.63233031756572	8.52886965898796
Ag	8.46973533803354	16.70538711366740	16.66724093107722
Ag	4.41895331929305	14.60338768416765	10.59108303518085
Ag	4.42872473430303	12.57997985036637	12.59423756232827
Ag	12.52328982825727	8.51281532856090	4.52210406143723
Ag	14.56607811467370	14.61974409632863	8.53603010166641
Ag	10.51275453783089	10.54437403670781	20.74904520973616
Ag	12.52560222263040	16.65979881398120	4.51856814072190
Ag	10.51928460147724	20.80041544572984	10.55871581631121
Ag	8.47274178931562	6.45802877912462	10.55611554160667
Ag	4.41488221958743	10.54767959688896	10.59392988793337
Ag	20.61521539588042	12.57374177774221	16.60320401186862
Ag	10.51525655624856	10.55733362936117	4.49635316971096
Ag	14.55618608806994	8.50314539720554	10.56842002832702
Ag	14.55690161948242	10.56742753852730	8.53361521939006
Ag	6.43581543003276	10.53003264768820	16.65287018469885
Ag	8.46971677493215	16.70746025675356	8.51626933536843
Ag	16.54142161003945	12.57054915019492	20.71282215250130

$Pd_{105}Ag_{300}L1_0$-1310.065

Pd	11.94476355466328	11.94474129822546	5.85397119884356
Pd	14.00001263105661	9.90181175403067	5.84792654874422
Pd	16.05526865009502	11.94473036288152	5.85397356180705
Pd	9.90182219990625	13.99999544228532	5.84791503457670
Pd	11.94477255923914	16.05523529725946	5.85399727234025
Pd	14.00003079797826	13.99998236888075	5.87366825682907
Pd	16.05526917257195	16.05525710231879	5.85397276707967
Pd	18.09818299527251	13.99999295327208	5.84792778968097
Pd	14.00000789936137	18.09819206579468	5.84790785813948
Pd	11.94418959768411	7.87537214539756	9.91783438554047
Pd	14.00002014726944	5.81103267093751	9.92311309435221
Pd	16.05585670599476	7.87535116612419	9.91780877271460
Pd	7.87534616479388	11.94415261379266	9.91782697503615
Pd	9.90453524968961	9.90452837669195	9.91626535215687
Pd	11.96425393864626	11.96421902663857	9.91636414009168
Pd	14.00004827504953	9.90918457400608	9.93143464460738
Pd	16.03580111444647	11.96421369290391	9.91633824260058
Pd	18.09547450262657	9.90451042862998	9.91623605062160
Pd	20.12461120297488	11.94415592143370	9.91780995083183
Pd	5.81100339544507	13.99999867247818	9.92308337761105
Pd	7.87530721462983	16.05584025001563	9.91783363562988
Pd	9.90917687957845	13.99997556508996	9.93146856305384

Pd	11.96425545619645	16.03577031659913	9.91641519143470
Pd	14.00002929545022	13.99999998070883	9.89653484833657
Pd	16.03579878099830	16.03577070334331	9.91635655373640
Pd	18.09081015502498	13.99998162364242	9.93142758822704
Pd	20.12461917936192	16.05582378667172	9.91781865784419
Pd	22.18892614423494	13.99998845461418	9.92309102998678
Pd	9.90451604519375	18.09549370286929	9.91625922907139
Pd	11.94418000579869	20.12469234130073	9.91783501428072
Pd	14.00005474496873	18.09084386287273	9.93146103624674
Pd	16.05585634373538	20.12466963313362	9.91780824174139
Pd	18.09547155664303	18.09548616816357	9.91624924689154
Pd	14.00002420532621	22.18900180863437	9.92310162155465
Pd	7.88760743920877	7.88763235768522	13.99999412649924
Pd	9.93186152155277	5.82071177079713	13.99999311267403
Pd	11.97020466552894	7.89540381354082	13.99999154412056
Pd	14.00004443890601	5.82458912087006	13.99999116700438
Pd	16.02988645217402	7.89538622713531	13.99999640870038
Pd	18.06816093532426	5.82068759269425	13.99997488487337
Pd	20.11228771365877	7.88765373568457	13.99999010393291
Pd	5.82061407822657	9.93172491021987	13.99999277306181
Pd	7.89534257809023	11.97011738486828	13.99999149838806
Pd	9.92381156241883	9.92376729776505	13.99998939477796
Pd	11.97215844099604	11.97205567767491	13.99998408894068
Pd	14.00007325040776	9.93020047817155	13.99998947882345
Pd	16.02794639574762	11.97208016820364	14.00000132859362
Pd	18.07621086752996	9.92377543842337	14.00000061262778
Pd	20.10458116552853	11.97011543377532	14.00000473823082
Pd	22.17924221511948	9.93182697392458	13.99999070479347
Pd	5.82450037040889	13.99998993567086	13.99999520102543
Pd	7.89529147075196	16.02988862762943	13.99999971101441
Pd	9.93018182400174	13.99998178226384	13.99999132326428
Pd	11.97213088938779	16.02792610947606	14.00000411758357
Pd	14.00006652081489	13.99998076773982	14.00000326529306
Pd	16.02796699241648	16.02788454507058	14.00002184371071
Pd	18.06979227697695	13.99996438875636	14.00001170238312
Pd	20.10457304882713	16.02983036079663	14.00000856653204
Pd	22.17533061042613	13.99997059087251	14.00000036026904
Pd	5.82057486830013	18.06831047112578	13.99998901590419
Pd	7.88750593779889	20.11249388132688	13.99998181625657
Pd	9.92374032856209	18.07628578539766	14.00000572566592
Pd	11.97013921548489	20.10471876591230	13.99999913021153
Pd	14.00005904207549	18.06985032464731	14.00001350743736
Pd	16.02988995271601	20.10466061924127	14.00001201303988
Pd	18.07621985144829	18.07621048662269	14.00002343433427
Pd	20.11231234296655	20.11235583608873	14.00001108512362
Pd	22.17926796654433	18.06810863567907	14.00000371099280
Pd	9.93169720454102	22.17940342616470	13.99999078077822
Pd	14.00002326273853	22.17550119808342	13.99999292451231
Pd	18.06822358492603	22.17935857904470	14.00000235986079
Pd	11.94419561647088	7.87535258596335	18.08214589703235
Pd	14.00002110158877	5.81103031460075	18.07687455501135
Pd	16.05585806293104	7.87533819464473	18.08218658845536
Pd	7.87533099331734	11.94415220935346	18.08215796922381
Pd	9.90453310712191	9.90452263919905	18.08370171547876
Pd	11.96426384296701	11.96421433450677	18.08362014494652
Pd	14.00005915077470	9.90916467930006	18.06854637625487
Pd	16.03581497796769	11.96420529468624	18.08367618616609
Pd	18.09548308270745	9.90450600803095	18.08377928775607
Pd	20.12463463619880	11.94414787666970	18.08221710415649
Pd	5.81098172521715	13.99999328336511	18.07688043955153
Pd	7.87529498479603	16.05583598272302	18.08216389498386
Pd	9.90917066280684	13.99996964794410	18.06851503667860

Pd	11.96425822615553	16.03576787754670	18.08359355001189
Pd	14.00003644031426	13.99999110153321	18.10349329134596
Pd	16.03581609065935	16.03577468082325	18.08367811453587
Pd	18.09083446952805	13.99997524945656	18.06860129058100
Pd	20.12463901680317	16.05582732581782	18.08221910061253
Pd	22.18894750801373	13.99998673656514	18.07693018692678
Pd	9.90450643888921	18.09550689005609	18.08373746758505
Pd	11.94416160064630	20.12471329742634	18.08217291245547
Pd	14.00005608353187	18.09086176036824	18.06855360180838
Pd	16.05586246388198	20.12468726278433	18.08220579481813
Pd	18.09549659781630	18.09549941417432	18.08378472766164
Pd	13.99998850368183	22.18901789392419	18.07690509366588
Pd	11.94475666223156	11.94472896387943	22.14600056765750
Pd	14.00001114773967	9.90178857372702	22.15206173724908
Pd	16.05528648803391	11.94471218439119	22.14605016360542
Pd	11.94477141778858	16.05523282612320	22.14598224819849
Pd	14.00004438313500	13.99998081650939	22.12632614157222
Pd	16.05527150139786	16.05528809611132	22.14598553836226
Pd	18.09819914355334	13.99998819565332	22.15213163991265
Pd	14.00001274151691	18.09819560108890	22.15208591179046
Pd	9.90181004172526	14.00000194161062	22.15206718759467
Ag	14.00001574343454	5.85128665654899	5.85363747759565
Ag	14.00002097236777	7.92402494254314	3.87736433104932
Ag	16.06720693469405	7.85307147077517	5.82961666364975
Ag	7.85308846219557	11.93281138682176	5.82956772122096
Ag	9.89395635433065	9.89393338692838	5.82546081563216
Ag	9.95022073516558	11.98126558830550	3.85996598803851
Ag	11.98129368449043	9.95019106708919	3.85997456255177
Ag	14.00003520519092	11.97922426065238	3.87890376878023
Ag	16.01875553418338	9.95016746445752	3.86000384958439
Ag	18.10608488329962	9.89390584694563	5.82546350080986
Ag	18.04981181845092	11.98123930742110	3.86001061608414
Ag	20.14690266696901	11.93279736339425	5.82960707096934
Ag	5.85119552446049	13.99997787044185	5.85352119158895
Ag	7.92401607423535	13.99998876217029	3.87732813103948
Ag	7.85309663724663	16.06714446102697	5.82962285665269
Ag	9.95020792623938	16.01872213402116	3.85999686258927
Ag	11.97928075041055	13.99998347402512	3.87890195441048
Ag	14.00003483712796	16.02074375096904	3.87891170630849
Ag	16.02078397787155	13.99998355695923	3.87891354309857
Ag	18.04981464945901	16.01872528202822	3.85999653901535
Ag	20.07593672655985	13.99997762888431	3.87733906663755
Ag	20.14690958537860	16.06716970783584	5.82958532461006
Ag	22.14870024518708	13.99997752994919	5.85364793151397
Ag	9.89398568991144	18.10601490046668	5.82554249167295
Ag	11.98127427663340	18.04978808647069	3.85998653498895
Ag	11.93285519581094	20.14689288370209	5.82962005074590
Ag	14.00001625873957	20.07595931885930	3.87729416137025
Ag	16.01875356959315	18.04980727651760	3.85998215037915
Ag	16.06717758069690	20.14690964596387	5.82960261041663
Ag	18.10608587325526	18.10607133652800	5.82543034572293
Ag	14.00001404432098	22.14876054519850	5.85349014892120
Ag	11.97392965493045	3.81928797029474	9.92674866516313
Ag	13.99998011567241	3.87046008348758	7.90666246773704
Ag	16.02608968732924	3.81928224285533	9.92669409560851
Ag	7.86430457096978	7.86428886354576	9.89805751509854
Ag	9.89715257269578	5.81752320107708	9.89182656826990
Ag	9.90723009457713	7.86988426067069	7.88604578431844
Ag	11.93956058280773	5.83976826759581	7.88070819889774
Ag	14.00003307915291	7.88017609983022	7.94196430660663
Ag	16.06046474556955	5.83972684255131	7.88068351522380
Ag	18.10287490059751	5.81748244126222	9.89182441682501

Ag	18.09281610898247	7.86984319544326	7.88603578855280
Ag	20.13569295738460	7.86426745483406	9.89804575066481
Ag	3.81927435656877	11.97388476803936	9.92668501051441
Ag	5.81749087710824	9.89708354597239	9.89180143396733
Ag	5.83974641567521	11.93951584593735	7.88063233470505
Ag	7.86989350042612	9.90718493973788	7.88602788514840
Ag	9.92226756707774	11.96026384701004	7.91822699512193
Ag	11.96030589394398	9.92225340396502	7.91824760800413
Ag	14.00004189976246	11.95503648605656	7.88821207606623
Ag	16.03975985514809	9.92223572801530	7.91824038202307
Ag	18.07775126186936	11.96024992296025	7.91824624109726
Ag	20.13012362643082	9.90718024858023	7.88604259323768
Ag	22.18244300427418	9.89712237129561	9.89184570632747
Ag	22.16022716707588	11.93953991088140	7.88068989076611
Ag	24.18066571214975	11.97388259959931	9.92672146163412
Ag	3.87046472591719	13.99997638037907	7.90657527610789
Ag	3.81926242306283	16.02608893119374	9.92667349322990
Ag	5.83970722643220	16.06046618020276	7.88061352988755
Ag	7.88016317578654	13.99997617971876	7.94193186386649
Ag	9.92226999246838	16.03969334925732	7.91826495061563
Ag	11.95508718381081	13.99997666157661	7.88820960879963
Ag	14.00004484891001	16.04493820046296	7.88820231732734
Ag	16.04497320635511	13.99998116093477	7.88820067924373
Ag	18.07775467791846	16.03971631489138	7.91824189721612
Ag	20.11979838966903	13.99997927474146	7.94195074831657
Ag	22.16023600494621	16.06041662577018	7.88068098095518
Ag	24.12956371331456	13.99997169448496	7.90671042242371
Ag	24.18067559943773	16.02607041307170	9.92671949191716
Ag	5.81743955484625	18.10291047225272	9.89178919980949
Ag	7.86984390205175	18.09281874224875	7.88601491567582
Ag	7.86424682499219	20.13575160246995	9.89805029578160
Ag	9.90718446863754	20.13015192469997	7.88602874119072
Ag	11.96031909930034	18.07773874348020	7.91826605391228
Ag	14.00003088383136	20.11985594998212	7.94193185450469
Ag	16.03975702669567	18.07774461883340	7.91823052135635
Ag	18.09282162483422	20.13012958458427	7.88599618307575
Ag	20.13012471705301	18.09277978248392	7.88601443849713
Ag	20.13570421241358	20.13569870600216	9.89803375741213
Ag	22.18246062245357	18.10284053172358	9.89183162828370
Ag	9.89710669839728	22.18255800916187	9.89180335194550
Ag	11.93954538514538	22.16027436454589	7.88063766275294
Ag	11.97392748428127	24.18075700268233	9.92666571943902
Ag	14.00001100191052	24.12948769351610	7.90655407696463
Ag	16.06049500383074	22.16028251675494	7.88060548343627
Ag	16.02612128775401	24.18073776696993	9.92666085886081
Ag	18.10291183772793	22.18250579296654	9.89177540497921
Ag	7.95544146966538	3.86083767085052	13.99999615173717
Ag	9.92882982157714	3.82919011888338	11.95611659681872
Ag	11.97291202545360	3.77889468011508	13.99999341673004
Ag	14.00004857829979	3.78954152536477	11.97686964305004
Ag	16.02718139730775	3.77889304392812	13.99999696757987
Ag	18.07116235191722	3.82913696296083	11.95608144742188
Ag	20.04469377564024	3.86080672246539	13.99998482878912
Ag	3.86059483495059	7.95533444269124	13.99999710186402
Ag	5.88040672465200	5.88042885088980	14.00000502443414
Ag	5.84784533941900	7.88008455245134	11.95333470521442
Ag	7.88014975676208	5.84790127919424	11.95333010120057
Ag	9.93244948941724	7.89176151420140	11.98640401598586
Ag	11.97541155387591	5.85771270820529	11.97709855446614
Ag	14.00006681248403	7.89296223950857	11.95498834112242
Ag	16.02468860204887	5.85769155011023	11.97708958566579
Ag	18.06757915771858	7.89177980090657	11.98639040229760

Ag	20.11978182058815	5.84787419972481	11.95334184330262
Ag	22.11941411276877	5.88046265199512	13.99999958114153
Ag	22.15205778964083	7.88013615556394	11.95332991773401
Ag	24.13903155568256	7.95541945458037	14.00000575406847
Ag	3.82912785382714	9.92873892633072	11.95607971755930
Ag	3.77882588294926	11.97278347228833	13.99999257129483
Ag	5.85766588517566	11.97531261613475	11.97709108371346
Ag	7.89176558019335	9.93237826362450	11.98640356253318
Ag	9.92971881933270	11.96585448634962	11.95633593538440
Ag	11.96595178939198	9.92967186522433	11.95632530545167
Ag	14.00007130103329	11.97015643594311	11.95533048187133
Ag	16.03415669792691	9.92967066194051	11.95632901578774
Ag	18.07033043269815	11.96585512766718	11.95632411868962
Ag	20.10820072535189	9.93240350148275	11.98639770314746
Ag	22.14225386482856	11.97532451483099	11.97708417748847
Ag	24.17083138923112	9.92877930482391	11.95611001464024
Ag	24.22099065827285	11.97282088150641	13.99998767188137
Ag	3.78948945366886	13.99998203253835	11.97685157596989
Ag	3.77880167011661	16.02718278769392	13.99999603927244
Ag	5.85764128204265	16.02466374268275	11.97709922341241
Ag	7.89293779559218	13.99998224792882	11.95498804111742
Ag	9.92969744662132	16.03411572719082	11.95633745616366
Ag	11.97024509183844	13.99997865527151	11.95534990566060
Ag	14.00006797250791	16.02980625258640	11.95536304910378
Ag	16.02986487654798	13.99996855325827	11.95533565245158
Ag	18.07032984493368	16.03408250093992	11.95633143790529
Ag	20.10701499463413	13.99996711039877	11.95499441209497
Ag	22.14227447299895	16.02461775734257	11.97708692592704
Ag	24.21039666456923	13.99996626154663	11.97687107853458
Ag	24.22102072836039	16.02710417792528	13.99999211835873
Ag	3.82909203323775	18.07124103616447	11.95607897439977
Ag	3.86056763246075	20.04473024243619	13.99999465396385
Ag	5.84773177080907	20.11996743516514	11.95331572643669
Ag	7.89169208056256	18.06764911501155	11.98641215923252
Ag	9.93237032677219	20.10831176677909	11.98640995804922
Ag	11.96591986284018	18.07033106305208	11.95634372622392
Ag	14.00003890398765	20.10707369937484	11.95499652545410
Ag	16.03416492838986	18.07031478029342	11.95634505198822
Ag	18.06760706166556	20.10823601981901	11.98639564401677
Ag	20.10821231720781	18.06754706554246	11.98640999456460
Ag	22.15204869491089	20.11979976540032	11.95333077134673
Ag	24.17085095500878	18.07117401344572	11.95610620842911
Ag	24.13902660267433	20.04452791238073	14.00000046257822
Ag	5.88026674416150	22.11969601386195	13.99998743168128
Ag	7.88002973221859	22.15222464997146	11.95330710464997
Ag	7.95529992885914	24.13939475259475	13.99999413451629
Ag	9.92875479886069	24.17090255248410	11.95608102353963
Ag	11.97534977083408	22.14238080874578	11.97708540428932
Ag	11.97284148381331	24.22119067336297	13.99998189879234
Ag	14.00003779213973	24.21053298330260	11.97684041770380
Ag	16.02469988162589	22.14233322030769	11.97708868250878
Ag	16.02721774069139	24.22117242549199	13.99998275865416
Ag	18.07123843329875	24.17086335687393	11.95606241989893
Ag	20.11987211418155	22.15212897783071	11.95332368406677
Ag	20.04458748793074	24.13932226234123	13.99999684353864
Ag	22.11948526922604	22.11956190976888	13.99999409556691
Ag	9.92883288504378	3.82918767522220	16.04387801282196
Ag	11.97395265520773	3.81928259859176	18.07327821493102
Ag	14.00004417971573	3.78952410051266	16.02312729687016
Ag	16.02609883268086	3.81926862813532	18.07329466256943
Ag	18.07119815077043	3.82917640285027	16.04390034613265
Ag	5.84784350675168	7.88009137053123	16.04666126645914

Ag	7.88015597886369	5.84790483361434	16.04666450117219
Ag	7.86430547040235	7.86429226212346	18.10193327840341
Ag	9.89715636326224	5.81751003142320	18.10817086080923
Ag	9.93245124813889	7.89175476766167	16.01357777582864
Ag	11.97540701722520	5.85769571271445	16.02289099069943
Ag	14.00006379327306	7.89295687152768	16.04499165091339
Ag	16.02467886842068	5.85769977144481	16.02290456847597
Ag	18.10288466109396	5.81748691990151	18.10818876141543
Ag	18.06758864175916	7.89175809040450	16.01360409388541
Ag	20.11981417046170	5.84790266792434	16.04665809293319
Ag	20.13569254335714	7.86427933436423	18.10196977549575
Ag	22.15201873373362	7.88017470418102	16.04665394804139
Ag	3.82912644728029	9.92874278478002	16.04391293396643
Ag	3.81926530125310	11.97388807123007	18.07332380855329
Ag	5.81748179819282	9.89708942590170	18.10820081401118
Ag	5.85765314109094	11.97530087410360	16.02288755335386
Ag	7.89175712347741	9.93237745241518	16.01357819898043
Ag	9.92971254157545	11.96585370485929	16.04363878043818
Ag	11.96595663826104	9.92966292272102	16.04364589752042
Ag	14.00007745332293	11.97015685743632	16.04466629344886
Ag	16.03416417917899	9.92965681437629	16.04366425200834
Ag	18.07033982174388	11.96585904596714	16.04368911584545
Ag	20.10819918269351	9.93241437218012	16.01360347113537
Ag	22.18246094949500	9.89711948366372	18.10817397731839
Ag	22.14226687760108	11.97531787833896	16.02291336359985
Ag	24.17082698991610	9.92878263636127	16.04387836954588
Ag	24.18067863213844	11.97388970476550	18.07328190332670
Ag	3.78946222038569	13.99998054837100	16.02313382670109
Ag	3.81924563582784	16.02607848004833	18.07333432570176
Ag	5.85762200338667	16.02467165288431	16.02289411616722
Ag	7.89293982474284	13.99997982911609	16.04499149363665
Ag	9.92968859930696	16.03411453301462	16.04366348041887
Ag	11.97025187690036	13.99997298348849	16.04464632265897
Ag	14.00007163177828	16.02980401441755	16.04467612209278
Ag	16.02986430329199	13.99996775960029	16.04470331092992
Ag	18.07034698100152	16.03408536650860	16.04370998071883
Ag	20.10702986727028	13.99995754720717	16.04503407266175
Ag	22.14227170403836	16.02462030994401	16.02291927799555
Ag	24.21039946224473	13.99997218910288	16.02311374571810
Ag	24.18068261986922	16.02606141057919	18.07327667812856
Ag	3.82908352431179	18.07123772480191	16.04392180562866
Ag	5.81742585859195	18.10291214914914	18.10819598808100
Ag	5.84779212676350	20.11993054331075	16.04661927543922
Ag	7.89169309561326	18.06764039230491	16.01359910011791
Ag	7.86423188038644	20.13576131398304	18.10194765867889
Ag	9.93236358219688	20.10832402376766	16.01358867036521
Ag	11.96591706656852	18.07034661177104	16.04367080190914
Ag	14.00004046519444	20.10708564494958	16.04501702502976
Ag	16.03417241508747	18.07032577343542	16.04369043255189
Ag	18.06762195733841	20.10825366438914	16.01363421957201
Ag	20.10822606793840	18.06756425083920	16.01362639472135
Ag	20.13573111040272	20.13572204255775	18.10198462358976
Ag	22.18248936065888	18.10284484188118	18.10817906236344
Ag	22.15206946072353	20.11981345653966	16.04667895546078
Ag	24.17086043228804	18.07116829740062	16.04389045140371
Ag	7.88003371214472	22.15223615841517	16.04668375059903
Ag	9.89709884133644	22.18256993744616	18.10820587057153
Ag	9.92875904649143	24.17089883657242	16.04389951175413
Ag	11.97534057862902	22.14238194212001	16.02288844768464
Ag	11.97392061164282	24.18075301475312	18.07332093625686
Ag	14.00003699393791	24.21053640231376	16.02312907277120
Ag	16.02468929632268	22.14235072916306	16.02292323444347

Ag	16.02610478179038	24.18073884690035	18.07332925917295
Ag	18.10291973752279	22.18254015872167	18.10822324854433
Ag	18.07123756348046	24.17087386511517	16.04392306871727
Ag	20.11988726092856	22.15214609295775	16.04668010978304
Ag	14.00002420845130	3.87056073709532	20.09339201993010
Ag	9.90723713876145	7.86988168463262	20.11394351265252
Ag	11.93955379451794	5.83975760226325	20.11931177495348
Ag	11.93283931816828	7.85308039004339	22.17037681731152
Ag	14.00001617173522	5.85127408709700	22.14644728792333
Ag	14.00002283328137	7.88015096903520	20.05802893029167
Ag	16.06049707947042	5.83972387322746	20.11932588752469
Ag	16.06717348527319	7.85307604067726	22.17033423998406
Ag	18.09282515240167	7.86983474417590	20.11398232553862
Ag	5.83972406308044	11.93953334759501	20.11936969397703
Ag	7.86989649052851	9.90718533607139	20.11396045773278
Ag	7.85310144102276	11.93284120995356	22.17036906014276
Ag	9.89395810432010	9.89392877876128	22.17451843823830
Ag	9.92226461554785	11.96025296463554	20.08172619112552
Ag	11.96029938987364	9.92223533238444	20.08170937478397
Ag	14.00005100056788	11.95502352234353	20.11179384239308
Ag	16.03976961902647	9.92222209245537	20.08175099158733
Ag	18.10608001305784	9.89389760741023	22.17455923586748
Ag	18.07776995456219	11.96023038169475	20.08178733067522
Ag	20.13014283389179	9.90716657670646	20.11399997668984
Ag	20.14690072939596	11.93281477547972	22.17038819426787
Ag	22.16026819893347	11.93953910092259	20.11934514974044
Ag	3.87047368618721	13.99997311190298	20.09343420552260
Ag	5.85118258660693	13.99998668866724	22.14652147881057
Ag	5.83969537547605	16.06044532997305	20.11938366437617
Ag	7.88013449479833	13.99998138945882	20.05804636947524
Ag	7.85310240557008	16.06713928346738	22.17036993817720
Ag	11.93283844848480	7.85309587118446	5.82961752305714
Ag	9.92225860007818	16.03970430819565	20.08170753777018
Ag	11.95508252020057	13.99996992415483	20.11179300093906
Ag	14.00005298224222	16.04494002914378	20.11179919901608
Ag	16.04499994428138	13.99997367603201	20.11182143369863
Ag	18.07777646746438	16.03972575968042	20.08179859807756
Ag	20.11982249758132	13.99997403724823	20.05806863571502
Ag	20.14690641993505	16.06714400705612	22.17040284013131
Ag	22.14872888912223	13.99997699327038	22.14641459426342
Ag	22.16026973264537	16.06041203955144	20.11934163141592
Ag	24.12958506821457	13.99997220832848	20.09333015896525
Ag	7.86983398979184	18.09281964353935	20.11397641960674
Ag	9.89397992129328	18.10601745733165	22.17444236814444
Ag	9.90717419620333	20.13017107143502	20.11399624983677
Ag	11.96030441318572	18.07775843604524	20.08172139720070
Ag	11.93285213612630	20.14691623614016	22.17038641551339
Ag	14.00002554179114	20.11987649935629	20.05807303771218
Ag	16.03977500321593	18.07776634347141	20.08178718469317
Ag	16.06717609786847	20.14691308009662	22.17039930077011
Ag	18.10608741841240	18.10606668250972	22.17458399923811
Ag	18.09284813351320	20.13015280774825	20.11402610600589
Ag	20.13015870245597	18.09279673587757	20.11401726422572
Ag	11.93953788731945	22.16030337276398	20.11939974146212
Ag	14.00002635470241	22.14881066327269	22.14651183383996
Ag	14.00002739404842	24.12952689332943	20.09343891101744
Ag	16.06048194162675	22.16028762844744	20.11937809834147
Ag	14.00001870890596	7.92401414625008	24.12262855198702
Ag	9.95022322919758	11.98126177179004	24.13997308188944
Ag	11.98129350157331	9.95017609907693	24.13997355850433
Ag	14.00002928303089	11.97920376003477	24.12105594022826
Ag	16.01873930891556	9.95013888898479	24.13998661221911

Ag	18.04980775557201	11.98122934131897	24.14003725560839
Ag	7.92403987007900	13.99998511652663	24.12267772536441
Ag	9.95022517063811	16.01871827762452	24.13997751408798
Ag	11.97928562947927	13.99997511514151	24.12105136265657
Ag	14.00002985533582	16.02074421202812	24.12109093271142
Ag	16.02077846679560	13.99997576109697	24.12110113628381
Ag	18.04981211163576	16.01872312302032	24.14006329203344
Ag	20.07593708296826	13.99997294595134	24.12269246567609
Ag	11.98128200917939	18.04979348850502	24.13995812895626
Ag	14.00001058519727	20.07597839926301	24.12266350711985
Ag	16.01873921995627	18.04981622743930	24.14002146840248

$Pd_{105}Ag_{300}$LEH-1311.749

Pd	13.99430747005812	9.89713960202321	5.82289624747368
Pd	16.04746831016970	11.94796948205325	5.83298571002103
Pd	9.90589946745590	14.00391138111094	5.83046575118397
Pd	11.95423412997714	16.05632952575797	5.85120987512868
Pd	14.00445807152686	14.01433445780278	5.85243518994428
Pd	16.06210937614328	16.05938596944569	5.83132752085711
Pd	18.10579887481410	14.00181206892760	5.83365298773912
Pd	13.98844900193470	18.08403898000453	5.85445321226040
Pd	14.00537779861430	5.82760282946942	9.91051591088351
Pd	14.00018291648446	7.86926315001244	7.86669588433065
Pd	9.90686224072172	9.90068443913224	9.89934673380588
Pd	9.90750883566920	11.94079973060482	7.88569330610520
Pd	13.99586656595868	9.90737142774124	9.89450903456713
Pd	18.10235958688042	9.89420633320152	9.90101158230579
Pd	5.82766436785695	13.99989632140947	9.90465245929078
Pd	7.87760902696412	16.05188767909364	9.90435245931075
Pd	9.91293144530677	14.00451413692937	9.91356905448405
Pd	13.99994073507249	14.01364893224019	9.91144002870496
Pd	16.04397006871549	16.04352648327775	9.90642364621534
Pd	18.09689039001720	14.00979959400174	9.89874486304773
Pd	20.13172625296759	14.00036910328401	7.87412951296238
Pd	22.16351119509908	13.99974661807643	9.90487251486408
Pd	9.90016121500411	18.09417403569859	9.90617424483455
Pd	11.96300881156933	18.09281802695338	7.87406657555717
Pd	14.00148996047642	18.08600261369141	9.90598738384071
Pd	13.99767034452977	20.09311343403879	7.87972559646620
Pd	18.10565084095522	18.09891593825311	9.89861726645348
Pd	14.00212052868422	22.14886185914442	9.90794231447858
Pd	9.91068101738277	5.83913124353278	14.00854419694285
Pd	11.94032516311572	5.82915167066721	11.94312729768065
Pd	16.07044728412355	5.84573000720160	11.95399182477657
Pd	18.09556329273400	5.85014941770725	13.99353545891869
Pd	18.09914328718442	7.85905470062435	11.94798957487536
Pd	20.12272301538944	7.87286070842503	14.00258631035554
Pd	5.81807641243953	9.89981010611258	13.99979177252163
Pd	7.87859449947407	9.90084946550060	11.94218661253086
Pd	9.90612055106019	9.91087483800652	13.99775983880754
Pd	11.96495265318242	9.90931740353992	11.95350192099439
Pd	14.00823501190249	9.89039994532386	13.99498144960131
Pd	18.09393648982595	11.97481504431580	11.94629451291463
Pd	22.14128654319355	9.87952232932686	14.00653450221327
Pd	22.15770882402600	11.93694679496873	11.93766247876450
Pd	5.84107888655705	13.99565125604549	14.02129556790137
Pd	5.83469680640653	16.03424679334131	11.92738508798499
Pd	9.90220356792748	13.99813287861676	14.00261356853288
Pd	11.94880030054824	14.00322264688220	11.94930130889141
Pd	13.99987380671211	14.00973227749228	13.99163653946720
Pd	16.04971963534555	16.04810028699162	14.00396705919439
Pd	18.10563410981595	13.98758151567122	13.99611860940999

Pd	22.12558512177089	14.00561801110374	13.99583103964517
Pd	22.15419148533381	16.06136528281360	11.94531428737885
Pd	5.83102791059049	18.09672806199990	14.00171293931805
Pd	7.86279428515098	20.12062912827546	13.99856866215030
Pd	9.89510724658921	18.09911635892569	13.99547584133894
Pd	11.96197940184418	18.09594442831044	11.97235274364448
Pd	14.00641834265045	18.09210344520568	13.99217115784921
Pd	18.09453681928031	18.10824335204905	14.00303190948035
Pd	18.10033711604457	20.13220307755672	11.93249279010207
Pd	22.15700593231364	18.10809291812888	14.01405111225749
Pd	9.88869769523066	22.15764558424677	13.99791334307764
Pd	11.94128471310868	22.15195688428695	11.93944924382082
Pd	14.00581780735764	22.12790166521981	13.99113276169953
Pd	16.07056334426009	22.14170484416930	11.95161169747314
Pd	18.09694777383976	22.15672226270448	13.99577986027921
Pd	9.90215742078349	7.88355966458704	16.05811689464475
Pd	11.92938747964471	5.85494548620013	16.04248809820212
Pd	14.00329634293758	5.84049822497759	18.08554437616170
Pd	16.07014067210670	5.83531133779771	16.06300510235254
Pd	5.82228803412456	11.94590252435453	16.05764717656432
Pd	7.87467119687266	11.93148767197546	18.09956440201388
Pd	9.89389498183796	9.90157535761096	18.09861342467682
Pd	11.96841369393326	11.93820472327203	18.08745199046889
Pd	14.01553065661395	9.90704981427880	18.10405459229172
Pd	16.03652637570775	9.91352700500557	16.04330974506314
Pd	18.10573590023387	9.90518797805220	18.09378964898609
Pd	20.13693827871118	9.89685122403338	16.04991618484489
Pd	22.14168305970900	11.92434895999037	16.04741602971843
Pd	5.82482704717242	16.06175360634276	16.07423346336926
Pd	7.87238610108084	16.07189334483787	18.09944532563398
Pd	11.97484493237651	16.05867172965322	18.08494546234285
Pd	13.99368096902703	13.99659578375872	18.10124816761778
Pd	18.09220663019117	13.99769560306929	18.10026304201378
Pd	22.15165300804719	14.00116749686337	18.09680778330830
Pd	22.13440185968181	16.07681667013608	16.05324539723368
Pd	9.89745853055207	18.09889604540930	18.10343734228497
Pd	14.00724824528016	18.10512099034667	18.09642318719476
Pd	18.09930352793367	18.10992753257047	18.08848105602929
Pd	18.09826327401188	20.12221376008170	16.06152381312351
Pd	20.10402203940064	18.11278460987379	16.05371283172463
Pd	11.94142175757449	22.15060513524179	16.05156396493533
Pd	14.00828766767986	22.16259790722219	18.09872908383560
Pd	16.07980852411714	22.14795355515923	16.04808807622452
Pd	14.01154019010205	7.90221026820284	20.12215366383525
Pd	11.95702023756549	11.94000311498382	22.16023786674780
Pd	14.00847835702158	9.90898291273765	22.16119102914145
Pd	16.05958041070293	9.89788113223689	20.11560921865246
Pd	18.09326486077765	11.91748891523132	20.11105105668116
Pd	7.86310566886322	14.00066381752648	20.10405666331734
Pd	9.90696932802751	14.00283278917841	22.15085836001697
Pd	11.95528976167111	16.05786646173985	22.16389423138112
Pd	13.99094637254607	13.99700806589719	22.15648309171306
Pd	18.09800031233585	13.99034878205443	22.18607436461259
Pd	18.10132892797339	16.07044583840178	20.12244484185581
Pd	14.00729331627929	18.09997397846027	22.17928902588260
Pd	14.01395842233854	20.13443341646906	20.13349311375937
Ag	11.93531322750274	7.85444948173155	5.81876106718644
Ag	14.00139241470063	5.85252291726670	5.86251715403093
Ag	14.00343816929224	7.91986074967264	3.85485394366616
Ag	16.06115462262876	7.84649997904703	5.82241690135356
Ag	7.87939610505361	11.94745300396823	5.84116506667991
Ag	9.88273535794479	9.88642348446300	5.80320583801857

Ag	9.92929190630760	11.97560204640670	3.81290844211975
Ag	11.96521319146100	9.93244501832145	3.82236926634707
Ag	11.94690984319085	11.96866501378395	5.84852240611115
Ag	14.01013748547610	11.97613571704409	3.85249414385410
Ag	16.03026643988156	9.93541165430161	3.85106920366899
Ag	18.12045762172044	9.88085208468603	5.79834568552688
Ag	18.06621873707762	11.97693297025446	3.85644718356258
Ag	20.14934533316777	11.94224721347032	5.82007885871079
Ag	5.84012431296962	14.00035143193671	5.83559789268463
Ag	7.91327002083313	13.99730661442072	3.87230596737825
Ag	7.83684880231957	16.07566733359880	5.80204422852537
Ag	9.93740706372279	16.02557204499003	3.86095355508276
Ag	11.97781924649146	14.02847703662397	3.84520608548050
Ag	14.00506674881904	16.03718800301827	3.86851827186515
Ag	16.03683539726067	14.00420972895989	3.87970100561005
Ag	18.07168425467791	16.02753153274811	3.85401503701721
Ag	20.08867130452611	14.00279965076312	3.85063146328293
Ag	20.14136738118725	16.05584491100900	5.83649513962892
Ag	22.13911366355881	13.99886703354773	5.86800752485039
Ag	9.90448039799062	18.10475536299711	5.83195197148700
Ag	11.97799163636093	18.05421581983571	3.85552120953857
Ag	11.95046246314543	20.11255915407870	5.86106298422246
Ag	13.99960455216410	20.06708551449787	3.87203688770220
Ag	16.02358990085248	18.06814177037760	3.86376548954633
Ag	16.05064917473862	20.12847339664318	5.84845758978395
Ag	18.12172294760973	18.11620494473348	5.80864747665080
Ag	13.99596612703820	22.12028213602262	5.87403125703363
Ag	11.96967121279563	3.85203524095216	9.93782379414972
Ag	13.99969475956103	3.85267096142042	7.92724177493677
Ag	16.03795125174202	3.85465431013058	9.94211057622637
Ag	7.86158615823294	7.87337024592593	9.89903843422592
Ag	9.88288117591531	5.81394811207006	9.88001239220571
Ag	9.87856173984794	7.84250102977124	7.84610117568247
Ag	11.94314726314665	5.83243161499912	7.86250931659961
Ag	11.97033261756445	7.88444936989780	9.91397935844645
Ag	16.05827326237567	5.82153452579455	7.85120609758649
Ag	16.03835612923986	7.86812109294485	9.91064237114491
Ag	18.10879369288023	5.82468280741691	9.90121215520804
Ag	18.11960242657468	7.85124985973090	7.83925078952511
Ag	20.12807458852652	7.86462881152503	9.89622725658728
Ag	3.82520144250337	11.97790580235765	9.93267751888057
Ag	5.81276651428566	9.88320756127590	9.88768723161966
Ag	5.80616245550683	11.93073922587060	7.84814089517014
Ag	7.87760112503731	9.90082219595260	7.85779872317164
Ag	7.90034321163079	11.94557983965463	9.91273227230597
Ag	11.96154978810373	9.90470910020401	7.87791128017734
Ag	11.94331255257233	11.96234364031247	9.92008971904653
Ag	14.01705868768266	11.96797662805304	7.86675746668952
Ag	16.03066160921044	9.90313427347103	7.86548522028438
Ag	16.06231565889840	11.96381509374904	9.92187923029126
Ag	18.09421649141746	11.97256968545911	7.85973786855111
Ag	20.15381825596230	9.87663762501583	7.84782828437355
Ag	20.10797354583107	11.96738655679953	9.91077296236983
Ag	22.19811356160474	9.87854903048721	9.87434175641633
Ag	22.16554630222325	11.94189523647299	7.86354805782702
Ag	24.14016504913810	11.97428527151225	9.94502272900821
Ag	3.87209543763976	14.00520943625297	7.90679699926837
Ag	3.83644393198859	16.02124229547742	9.93067595726759
Ag	5.84359049158452	16.05384558710683	7.85965252467046
Ag	7.90176943182084	14.00132565960734	7.90243638239975
Ag	9.92379736127118	16.05842557990338	7.88737246709742
Ag	11.93889822655291	14.00532708631182	7.87425894896966

Ag	11.96421497490855	16.06506328198117	9.93670874278121
Ag	13.99678628527540	16.05298230179210	7.87440999785795
Ag	16.04462604342904	14.02199995728851	7.85977336350569
Ag	18.08949585391795	16.03724724417961	7.88585146705166
Ag	20.13311960574191	16.02946439282670	9.90343934609921
Ag	22.18192919090253	16.06328270397916	7.85402215961090
Ag	24.14412458441349	13.99804531144207	7.92908986262030
Ag	24.13541629257332	16.02689458045506	9.94731641156958
Ag	5.82416452405206	18.09750412808418	9.89099482612077
Ag	7.85650005710328	18.09631262944961	7.87512588065411
Ag	7.84729652995083	20.15575770155146	9.87612134362416
Ag	9.90012469233616	20.11637622033387	7.87237099791858
Ag	11.96770455468877	20.08499161475467	9.91236338987006
Ag	16.04148906844675	18.07482264428084	7.89298757801824
Ag	16.03895140798954	20.11793108783183	9.92091130143400
Ag	18.12117139388564	20.14216938710150	7.84981136498602
Ag	20.15915340491348	18.12472529711988	7.84806383873498
Ag	20.13342073040062	20.13882903179916	9.90121892174216
Ag	22.19649321967248	18.12325666349242	9.88129984054506
Ag	9.88370763116336	22.18267211941967	9.88129917381401
Ag	11.95034570229621	22.15035671429864	7.87430299111746
Ag	11.97491157649473	24.13375739740599	9.94167687765488
Ag	14.00310003571554	24.13074290619844	7.93647512146217
Ag	16.05330271524839	22.15373023967914	7.86601236150649
Ag	16.02839033205381	24.13262868185501	9.94820352335788
Ag	18.10948902070258	22.17825286957670	9.90042407188034
Ag	7.90149258614179	3.87797972051050	14.00129206193821
Ag	9.93173657953710	3.85645199008820	11.98077259349253
Ag	11.95779794561887	3.85135810737105	14.00423329644635
Ag	14.00930399110027	3.85337044179335	11.95849447366160
Ag	16.05338091913551	3.85894345656327	13.99976641599452
Ag	18.07085318867094	3.84531359038493	11.97404992085575
Ag	20.09543980415948	3.86382421860193	14.00286337370457
Ag	3.85794140903749	7.90170220410261	13.99952283782923
Ag	5.83510609767041	5.83695354545387	14.00048050727671
Ag	5.83817298500113	7.87915727711906	11.94406414787672
Ag	7.84529665031710	5.81915524623349	11.93230986271395
Ag	7.89722968543930	7.90388460783850	14.00388008298785
Ag	9.92279735712140	7.90210477163336	11.94676800302456
Ag	11.92602725199049	7.88774145869299	14.00172376023576
Ag	13.99925741485299	5.86674515468942	14.00064037297910
Ag	13.98783150418763	7.88037935759836	11.94544165133833
Ag	16.06580165905254	7.87084915353397	13.99856439842375
Ag	20.12471186991720	5.86084742044814	11.96245103573405
Ag	22.15018261190652	5.85280914147746	14.00179711636889
Ag	22.16850244080561	7.86067735742074	11.95059717217237
Ag	24.14774071015008	7.90171062039429	14.00302108555920
Ag	3.81608172552048	9.92643688808258	11.97351227172758
Ag	3.82870502146543	11.97142000558557	14.01746922653510
Ag	5.84919423256360	11.94779022427724	11.96565510252295
Ag	7.86531724321965	11.93651171703885	14.00004730690613
Ag	9.92365046955033	11.94463853552707	11.96612365655366
Ag	11.96517928799144	11.95656984736172	13.98205452699892
Ag	13.97818685719245	11.96236789641477	11.94885796333072
Ag	16.06687592810286	9.91479450176835	11.93895076122597
Ag	16.03817252636363	11.95350101411164	13.99131412675099
Ag	18.10750927681427	9.90723460372037	13.99483568966198
Ag	20.11114063557821	9.91069283598516	11.94664342447698
Ag	20.15323802111118	11.94893708714477	13.99764914740317
Ag	24.13672297699934	9.92952790452579	11.98517614606148
Ag	24.09147535241491	11.96214006569645	14.00025095614811
Ag	3.84357971425583	14.00851807245370	11.97855231677532

Ag	3.88250263716653	16.03692607371093	14.00106505593551
Ag	7.86206572893372	14.02594805321490	11.94316492256138
Ag	7.84145193234518	16.03972000731959	14.00612747686833
Ag	9.91910002921308	16.04864206429109	11.94722712786833
Ag	11.95417684661659	16.04009179199771	13.97241290109091
Ag	14.00908572118287	16.04686992937761	11.95949859347591
Ag	16.05580984349250	14.01769572753259	11.95961928142792
Ag	18.07358638442962	16.04520532485733	11.96306193130519
Ag	20.13572147521453	13.99527342066806	11.95686001020267
Ag	20.14677732019160	16.07159021205842	14.00564160087011
Ag	24.11781869040171	14.00076097801213	11.97747053505929
Ag	24.10248904233411	16.03151132690943	14.00627928416598
Ag	3.86242606666950	18.05181368396614	11.96691413290711
Ag	3.85467805962040	20.06487202132137	13.99655283728357
Ag	5.82908075396760	20.14036949805989	11.93768880151730
Ag	7.86233640169731	18.07816153325233	11.96120344874840
Ag	9.91390865148590	20.10193392731360	11.96198251848342
Ag	11.95561071426055	20.13300614401687	13.99036493839602
Ag	13.99592618968304	20.12470381750193	11.95992987338040
Ag	16.06416845824105	18.07589402889610	11.96012195464701
Ag	16.08635393267661	20.14598502620909	13.99103101481575
Ag	20.11814178621962	18.10130791779316	11.95530267555764
Ag	20.08561092827449	20.11387584643964	14.01212165774097
Ag	22.19286987243751	20.16235105828758	11.94126017733172
Ag	24.13539286303895	18.06726264211084	11.98700897814772
Ag	24.13162784721227	20.09883596431909	14.01168872189450
Ag	5.86690845080779	22.13110715281792	13.99803121356756
Ag	7.85873088362928	22.16058520737403	11.93844942711131
Ag	7.91247385037207	24.14098402388408	13.99388000095608
Ag	9.94213699569192	24.13939308566411	11.97819515194540
Ag	11.97146090647523	24.10152140619200	13.99717433596617
Ag	14.00857223898871	24.10600712867161	11.96716099761361
Ag	16.03073433871803	24.11083391881767	13.99640585733315
Ag	18.06551655745965	24.15190928154427	11.98381525672438
Ag	20.13725955686176	22.16479218224377	11.94717118710121
Ag	20.09833637723666	24.12763109306621	14.00254962245498
Ag	22.16570993036812	22.17029189676919	14.00702770972469
Ag	9.93132831247597	3.84377402649799	16.02643854644062
Ag	11.96697525882539	3.87404460168509	18.05362218557379
Ag	13.99710339246650	3.86321002168159	16.04407045001164
Ag	16.03565519693324	3.85604584601772	18.06652011723730
Ag	18.07531664713613	3.85515563242897	16.02050214209375
Ag	5.78917444324964	7.83361854115304	16.07844885749084
Ag	7.85887428429840	5.84418781810401	16.05640244212132
Ag	7.86015080482941	7.85864308145543	18.09923868138152
Ag	9.89233401670442	5.83723472930863	18.09392083491037
Ag	11.96342717877504	7.87613005696176	18.08536543598056
Ag	14.01640334586910	7.88001089482364	16.05234565655203
Ag	16.04266676841257	7.90583387532069	18.08961761784312
Ag	18.13089140174144	5.80784715875336	18.12652051218897
Ag	18.09326208408842	7.89482250577532	16.05077270440108
Ag	20.13717026908024	5.83353940025820	16.05136365578702
Ag	20.14216569492609	7.87146057153908	18.10818148229493
Ag	22.14696544065709	7.86926647721668	16.04516223411792
Ag	3.85150175875551	9.93109295920507	16.02423848894450
Ag	3.82076009681811	11.98383117886436	18.07407637250600
Ag	5.80936569962080	9.90344509036729	18.11240101087875
Ag	7.86691745139473	9.91452061693757	16.05466830649146
Ag	9.91638059168686	11.95143876725871	16.05710220279987
Ag	11.94385688618018	9.91192955760335	16.05936036268588
Ag	14.00491044620707	11.93843848611551	16.05540887599678
Ag	16.06343440593666	11.92858673373748	18.09691848432989

Ag	18.09317571862559	11.94113479656865	16.04763245674762
Ag	20.12379712227531	11.94101798891832	18.08469250151439
Ag	22.18491199334969	9.88474792514517	18.10362043395634
Ag	24.15082615382531	9.93424330759314	16.01918673251314
Ag	24.12586412022713	11.97055636260445	18.04893195071331
Ag	3.83382251575374	14.00789337797425	16.02949626047806
Ag	3.81645469916874	16.02322537786865	18.07994404823332
Ag	5.87141790719523	13.99996582432966	18.08306344081525
Ag	7.85419286713409	13.99774340619769	16.04672051642753
Ag	9.89798093028281	14.00283031138987	18.09312191703079
Ag	9.91497640636658	16.05698497396910	16.05441823421657
Ag	11.97992127368958	13.99884935566173	16.05958027871686
Ag	14.01094620479803	16.04758386031515	16.04042730703713
Ag	16.04652076037370	14.00627039300052	16.03197852491730
Ag	16.06733881857410	16.04852255641980	18.09777425523880
Ag	18.08860406703580	16.04968670851655	16.01310939144624
Ag	20.14548097659485	13.99834738865196	16.04368127370810
Ag	20.10083804156139	16.05812850854413	18.07747315212750
Ag	24.09023968690331	14.00089276799634	16.01961511716229
Ag	24.11904527259045	16.02843293736470	18.04940256369878
Ag	3.84296337825580	18.06259847730389	16.03227481003502
Ag	5.80755491607915	18.09965930811951	18.11369705186087
Ag	5.81989962466687	20.14242323356935	16.05943134861889
Ag	7.86470185716888	18.08879962713365	16.04005293573752
Ag	7.84464898049650	20.15597718904866	18.12138342485931
Ag	9.89985241490633	20.12544833719740	16.02957456488507
Ag	11.96534278304590	18.08303237736892	16.05378763318160
Ag	11.97266373252030	20.13715358390781	18.09794689777444
Ag	14.00695700269324	20.15157277186009	16.03293749409858
Ag	16.05343886522972	18.09369109177007	16.02002315119634
Ag	16.04714242641531	20.12789630186597	18.07981275021888
Ag	20.11507725813182	20.12248094442170	18.08706626439177
Ag	22.15632988309561	18.11319528686977	18.09997740865821
Ag	22.16183897015476	20.13946254448780	16.06018793494023
Ag	24.14867268494163	18.06626008108127	16.02633071568895
Ag	7.84756672439965	22.17427221739683	16.05785756382956
Ag	9.88295355358466	22.19641542608725	18.11588520257435
Ag	9.94007291360934	24.13891324154333	16.02017550265481
Ag	11.97603672899350	24.13691149625291	18.05066073094797
Ag	14.00830348376556	24.09988930192851	16.02210722738153
Ag	16.03090438510557	24.13622711615943	18.05182616359052
Ag	18.11732502575553	22.17767170041689	18.10838905530474
Ag	18.06799788378072	24.15339503776887	16.01108153319818
Ag	20.13089434010974	22.17611299781532	16.06082274201487
Ag	14.00593699015962	3.86984087598274	20.07285141710389
Ag	9.87428617805640	7.85407275818477	20.15611135791485
Ag	11.94856581013165	5.84468072575725	20.13955097632195
Ag	11.94717961516273	7.86544412200739	22.15018289365873
Ag	14.00945732174501	5.87682346282693	22.12474075981086
Ag	16.05578167087615	5.84429626456642	20.12714929555577
Ag	16.05360849303279	7.89752244072771	22.13844287652081
Ag	18.11274572554537	7.86785083417570	20.13216770015061
Ag	5.83203950400716	11.94474607847079	20.13404503384848
Ag	7.85571216785420	9.89575609241428	20.12674764894140
Ag	7.85899646909117	11.95549794021152	22.15525975150394
Ag	9.88148174679373	9.88111896120516	22.19133172000286
Ag	9.91505211270769	11.95465290057638	20.11066257256025
Ag	11.96271026465152	9.91734409804264	20.10710817419493
Ag	13.99642878581865	11.94614726374083	20.11635074091026
Ag	16.05424445201860	11.95366967624411	22.13153612542379
Ag	18.10813529309056	9.89597306816358	22.17543167431418
Ag	20.14205991413429	9.89642246254814	20.14568847687591

Ag	20.12838495025438	11.93968898808841	22.16387327663104
Ag	22.19196199762968	11.93275329090226	20.16198473021751
Ag	3.82166566152454	13.99986673655298	20.10962041849997
Ag	5.84418896292531	14.00004705575725	22.12672175885676
Ag	5.83052705423937	16.05550174635470	20.13657679126252
Ag	7.86489613335974	16.04400460422930	22.15142229981513
Ag	9.92260371993869	16.04793808967569	20.10881868464755
Ag	11.97182033020338	13.99688710174009	20.13233171677457
Ag	13.98638890765284	16.04182220865534	20.12242757344149
Ag	16.08568745736166	13.99767335182212	20.11711330724177
Ag	16.05345386581956	16.02860879596951	22.14997361035282
Ag	20.07423829694435	13.99725014335233	20.12204496543978
Ag	20.13241739106654	16.06012244819139	22.16851075748630
Ag	22.16273132511422	13.99901152038970	22.16735246426028
Ag	22.17714319614699	16.07018928649345	20.15576317631831
Ag	24.12514939059101	14.00214674381459	20.07477060063638
Ag	7.85976601491155	18.10330077446408	20.12860625933127
Ag	9.88941274666887	18.11711201244428	22.19063771211439
Ag	9.88148882128032	20.15071250467257	20.15463386111904
Ag	11.97547634672859	18.08993450191522	20.11543659156614
Ag	11.94595359142469	20.14600355332631	22.16400332124365
Ag	16.04640413679785	18.10447864907449	20.11602702114485
Ag	16.06545448182681	20.14206676110928	22.18282120612536
Ag	18.12029678310780	18.11778246443732	22.20021378019328
Ag	18.12797447150127	20.16295146703987	20.16176487844880
Ag	20.12429364283896	18.10410793622908	20.14888192239469
Ag	11.94192032584377	22.17807999316874	20.15047875891989
Ag	14.00266448468621	22.14413192015115	22.13974393978862
Ag	14.01139258281824	24.14255842247101	20.06712099225082
Ag	16.06957117949883	22.17574819726454	20.14733008393485
Ag	14.00473499128001	7.92363399467518	24.12593492338068
Ag	9.93548515253038	11.97566970650408	24.12758391841111
Ag	11.97270362064406	9.93022920402906	24.13924256455659
Ag	13.99017482527397	11.96697146011613	24.15699588054791
Ag	16.03638957456655	9.93204932489015	24.17369292342128
Ag	18.08147508775647	11.97145813969677	24.18924552960331
Ag	7.91355776101937	13.99969174947100	24.13040633997517
Ag	9.93650124669745	16.02552921167562	24.13922922980581
Ag	11.96280546483937	13.99908962862846	24.11803436410255
Ag	13.98989767410224	16.02841587898330	24.15963845368933
Ag	16.02149257728770	13.98741221562136	24.18600037808940
Ag	18.07712954944750	16.01941309790432	24.18419451779942
Ag	20.11003445100823	13.99911741206748	24.13120815752262
Ag	11.97057834749138	18.06499203450038	24.14990382342751
Ag	14.00188315943613	20.07670014381744	24.14961303195987
Ag	16.03745527845730	18.07077833243822	24.17543929386293

$Pd_{43}Ag_{158}L1_1$-614.310

Pd	8.45536046188404	12.49998407311585	16.54466115601103
Pd	12.50001376932069	14.51399028168348	14.51402104147062
Pd	10.46499989198229	18.61608722848305	12.48643835991106
Pd	12.50000620948422	10.48596575466539	10.48601760595337
Pd	12.49999575977038	8.45534114813002	8.45537865512130
Pd	14.53498848349560	6.38397843415947	12.51359254631877
Pd	14.53978978678269	10.46020558804343	16.55293782555279
Pd	18.61602162837903	12.48642366168179	14.53498013464448
Pd	8.45175622641662	12.52116617059853	8.45177394120041
Pd	10.46021256185161	16.55297777759159	10.46020401598236
Pd	12.52120390432336	8.45177381879032	16.54823985791485
Pd	8.45535814584131	8.45535275061143	12.50004833437475
Pd	14.53978753961102	8.44706154738880	14.53979597524405
Pd	14.53499619069120	12.48640736896044	18.61600611909617

Pd	10.46498970882713	12.51355570618175	6.38391814605817
Pd	10.48525885169860	14.51473335015369	16.53332929960251
Pd	12.47882507499955	16.54825800799504	8.45172282985280
Pd	8.44701068085790	14.53977917743381	10.46023018838523
Pd	8.46669158932523	10.48523189632420	14.51479286138425
Pd	16.55291756420117	10.46020600453932	14.53979077856198
Pd	12.50001127301006	16.54463568893290	16.54463495408760
Pd	12.51359428257662	6.38399561989049	14.53499172900978
Pd	14.51401176997520	12.49998103602991	10.48599070276082
Pd	18.61598656986885	10.46501786095457	12.51360937038458
Pd	14.51476678118637	16.53330639557302	14.51474292860220
Pd	6.38392549900579	12.51356085918801	10.46503097835009
Pd	10.48599068860431	10.48597245291345	12.50004610706694
Pd	16.54465247786903	12.49998608814267	8.45533073457907
Pd	6.38392621286685	14.53499399438660	12.48645291805025
Pd	10.48599422591581	12.49997428883854	14.51403860520551
Pd	14.51401610918271	14.51399332112418	12.49999474481076
Pd	10.48525008878287	8.46666550561103	10.48528936632900
Pd	16.54822299215135	12.47878926654377	16.54821056885272
Pd	12.48643099242783	18.61608627349440	10.46500163411807
Pd	8.45176105236095	16.54824376845664	12.47883270553874
Pd	16.53332363643937	14.51474272124163	10.48523028313591
Pd	12.48643428326137	14.53499787729151	6.38388856943318
Pd	10.46021154052030	14.53978720645290	8.44701842081242
Pd	12.50000690452979	12.49997310553914	12.50002361068745
Pd	16.54465516982220	16.54464140074294	12.49997391010301
Pd	12.51358925344823	10.46500474660002	18.61602137923889
Pd	16.54821425676052	8.45176010262104	12.52120659969197
Pd	14.51476038808370	10.48522669380239	8.46668920860417
Ag	20.59366099360917	8.46635951519752	12.50587984871059
Ag	16.54852201304119	12.49099294038516	4.36671670396400
Ag	10.46447346594365	4.29120841567806	10.46449402078794
Ag	10.46081103407083	10.48759148458051	16.55377279145556
Ag	12.50792022472399	12.49207025349302	16.57645152878009
Ag	18.62556472256755	16.59704016603782	14.54802778982484
Ag	10.46880688287457	18.58706169595928	8.44402624044364
Ag	14.53233110392562	12.49579031219376	14.53232748228371
Ag	10.46766675545869	14.53232815243916	12.49582284554937
Ag	18.58250184191111	14.52177805824148	16.54858785293946
Ag	10.46976183871160	6.38597648565375	12.49537179410213
Ag	10.48760272486965	16.55375358776412	14.53921010537043
Ag	14.53119678805083	6.41290615464367	16.55605920258026
Ag	16.54853100204251	20.63327024158418	12.50896657777848
Ag	8.43430009071991	8.41430720398832	8.43433462227099
Ag	10.47138775443722	6.40890801135116	8.45592296600426
Ag	16.56001371379514	8.43999315675921	16.56001273980047
Ag	12.48892458684118	18.58218681649956	6.41782584233747
Ag	16.57643646119605	12.49209214078984	12.50789970599758
Ag	10.45194800654672	18.62556983255561	16.59703214949126
Ag	16.54861795827741	14.52177330513797	18.58247258141591
Ag	14.52860483492564	8.45588003555671	6.40891007633940
Ag	18.57752964218261	12.49999504423138	6.42244958164238
Ag	20.63326457776139	12.50897172516188	8.45147199035268
Ag	12.50588085214650	4.40634560667773	16.53363499968716
Ag	10.47140311469202	16.54409948457071	18.59107618285077
Ag	16.56570381049283	16.58566835539253	16.56567321846966
Ag	18.58214970155269	6.41788640854446	12.51106124236017
Ag	8.45136781220723	18.58253620961559	14.52179443958564
Ag	8.45135768359996	10.47819362910652	6.41748729990984
Ag	8.40296181962976	10.45194382515102	18.62554812090052
Ag	12.50911881217894	4.33036713632254	12.50914561885579
Ag	14.53921649995590	16.55376479590056	10.48763324369083

Ag	14.51519685216506	10.48481249711292	20.62268641847920
Ag	10.48686509054249	20.63837843855172	14.51019373306381
Ag	6.41746581046155	16.54862733916451	14.52180779594086
Ag	12.49208649036140	12.50789480633527	8.42352193167445
Ag	8.44623775536966	14.51239182504750	14.53922478214534
Ag	12.50584709025181	8.46634730254744	20.59366054819428
Ag	20.62270005851163	10.48484619801735	14.51520087769624
Ag	6.41746536156973	10.47819993213603	8.45140466015939
Ag	12.50420115886368	10.46765703848395	14.53235199588116
Ag	10.46766304003522	12.50417512455257	10.46768083072045
Ag	6.41288134116374	14.53116225103311	8.44395861007081
Ag	16.55375754605309	10.48765315456074	10.46078947587602
Ag	4.36674463720508	8.45146887117694	12.50904302903515
Ag	12.50913527398740	12.49087233191292	20.66962011573317
Ag	10.48981292789764	4.36168485544355	14.51314895564185
Ag	12.49999628089127	6.42247579826569	6.42249356689341
Ag	18.58251080473004	8.45137175838035	10.47823447389448
Ag	16.55374862188731	14.53919351856746	14.51237254225781
Ag	12.49534846269896	14.53022924192738	18.61400331684273
Ag	16.59706870747059	14.54805846321104	6.37441194185120
Ag	8.46631446795131	20.59370955413158	12.49417786738982
Ag	6.41287936072704	16.55605847507102	10.46884229038713
Ag	14.51522459540573	4.37731025344338	14.51516957457014
Ag	10.46449313309241	14.53551868286010	20.70878396886042
Ag	14.53025284200165	18.61405294136774	12.50464284168683
Ag	14.51020788235545	14.51313915319638	4.36159264394964
Ag	8.45590064949435	14.52860080694047	18.59109285717215
Ag	4.36673754438030	12.49100160235233	16.54854980062869
Ag	10.47822969494374	8.45138583746902	18.58251352196092
Ag	8.44402949074703	14.53116996902990	6.41294109222144
Ag	14.53553233688569	10.46445473025352	4.29116652451908
Ag	10.47824362011712	6.41749008591972	16.54863469118768
Ag	16.54411643305330	18.59109798331161	14.52857181875779
Ag	6.38595977731777	12.49534649699590	14.53025789745829
Ag	6.38596231730282	10.46975171103103	12.50469449720276
Ag	18.57753343435407	18.57753188813608	12.49995408725791
Ag	18.61401266240489	12.50466156549032	10.46974764374326
Ag	8.42352810004523	12.50790242456045	12.49211808099100
Ag	8.45149388441900	12.50899695002573	20.63325494355337
Ag	12.49100790473401	16.54851618455906	20.63322490330994
Ag	16.55605990376487	6.41289616063181	14.53118296560999
Ag	4.40630551805124	12.50581046721627	8.46636331123310
Ag	10.48765107950985	10.46077387268295	8.44625126508415
Ag	14.54806803663869	16.59703632624267	18.62553435186049
Ag	4.29116346407011	10.46446210247202	14.53554923108524
Ag	18.58213279289177	12.48894096240847	18.58210354152193
Ag	12.50466767198492	18.61404534603131	14.53022987933199
Ag	14.52179694136182	16.54864201593734	6.41742796320719
Ag	6.40890776146320	8.45588184537179	14.52864670505188
Ag	12.49209458853146	16.57646852149311	12.49209444256003
Ag	10.45193044108154	8.40294073480329	6.37444095385915
Ag	20.70882110914007	14.53551826404700	10.46446471880495
Ag	12.50001233062667	18.57752377273732	18.57749904192564
Ag	14.53232770629272	10.46765815627301	12.50420673462210
Ag	14.51311862673436	4.36167905784496	10.48983860762152
Ag	12.49413930169469	20.59371214552573	8.46633775951833
Ag	20.63832991394309	14.51019135625690	14.51309306447932
Ag	16.59705570558958	18.62557845774626	10.45190555650074
Ag	12.49099551325047	4.36675411199845	8.45150446980558
Ag	8.46631646110150	12.50580991744243	4.40630794190299
Ag	8.45148465308214	4.36674846511397	12.49103846423180
Ag	14.51022112652025	20.63838396526774	10.48682913400319

Ag	14.54805191808708	6.37444519635106	8.40298168918454
Ag	14.52178230906203	18.58256058420901	8.45133212128909
Ag	16.54411424259637	10.47138048674250	6.40888739817448
Ag	12.50465535483074	10.46974469405050	6.38596496346272
Ag	12.49580121207324	14.53232880553687	10.46765687506741
Ag	10.46080679378418	8.44623315415824	14.51237540593564
Ag	10.46976975248212	12.50464616766045	18.61402160647450
Ag	18.58709878779825	8.44394915166736	14.53118838676972
Ag	6.37442842984216	8.40294318050127	10.45197430980107
Ag	12.51107257353364	6.41785663253411	18.58212515257829
Ag	12.50901128989492	20.63325931059303	16.54850506276162
Ag	10.48479653737720	14.51518534025727	4.37727018493603
Ag	14.51234147479062	14.53919063737722	16.55374414373355
Ag	10.46881687970262	16.55596176754407	6.41291250249372
Ag	8.40297361007732	6.37445090629219	14.54808560752923
Ag	16.56569768451312	8.43429516607554	8.41432877998456
Ag	14.53555173554824	20.70883130752559	14.53552603543291
Ag	16.53366510286141	4.40634862320507	12.50584042069483
Ag	20.66964262971502	12.49089487108960	12.50912643561997
Ag	18.58710203754114	10.46882237197384	16.55603656523939
Ag	8.44623565061416	10.46078109624469	10.48763565232119
Ag	8.44402860715504	18.58706024547701	10.46882058138139
Ag	14.52861979877667	18.59109435214247	16.54408847318990
Ag	4.36163263470663	14.51314021982036	14.51022401794951
Ag	18.59110381909917	16.54411806460815	10.47135557511678
Ag	12.49533514737608	6.38597776415124	10.46978705387284
Ag	12.49089323469047	20.66969270569939	12.49086630348702
Ag	14.53024681273687	12.49533154167799	6.38594120911236
Ag	18.61402260148217	14.53024514128786	12.49531551142776
Ag	18.62556079569011	10.45193121308511	8.40295129308324
Ag	4.40630167972330	16.53366453372986	12.49419912768886
Ag	16.55605855292488	10.46881887196307	18.58708932516500
Ag	12.50791124286911	8.42354994588285	12.50793469986104
Ag	14.51313039410068	14.51017734445000	20.63829388219191
Ag	12.49087579600215	12.50910999302284	4.33030961669004
Ag	6.41783199967001	12.51105482830663	6.41786285670396
Ag	6.42247677321471	6.42247895477951	12.50005016783638
Ag	20.63828704121130	10.48690221326918	10.48981610374113
Ag	6.41783280917839	18.58216232715180	12.48893783319932
Ag	14.53118590196643	8.44394627098872	18.58711044261796
Ag	16.54861053107818	6.41749157849352	10.47823404880720
Ag	6.37443335260773	14.54805754491434	16.59706486097608
Ag	6.42248254543499	12.49999274418111	18.57753383299743
Ag	16.58569818789767	16.56569866928115	8.43426603935064
Ag	20.63325827213554	16.54852273808699	12.49095897951981
Ag	8.41433487153398	8.43430761089510	16.56572072940326
Ag	18.59111829832914	14.52859501870807	8.45587997657218
Ag	8.43430974071122	16.56568981005257	16.58568661502584
Ag	4.37727258119748	14.51518369511911	10.48483537196102
Ag	4.33032295079972	12.50911676873792	12.49090719666265
Ag	12.50899773824179	8.45146726750205	4.36675155653224
Ag	14.53921292624590	14.51239385869434	8.44622193892554
Ag	10.48982595904176	10.48687809811311	20.63833057867503
Ag	12.49417184767636	16.53367478633917	4.40627368439614
Ag	10.48476949002088	20.62272623543255	10.48482870919987
Ag	8.45589450923834	6.40891232740918	10.47144024367762
Ag	4.36163612188180	10.48979606550723	10.48689878302037
Ag	20.59366104960004	12.49417631751529	16.53363739270275
Ag	10.48685198058676	10.48978410032072	4.36163796932392
Ag	14.51239123503885	8.44624349447453	10.46081721112771
Ag	6.40889540775543	10.47139793351415	16.54411048284697
Ag	8.43996109922461	16.56003203649731	8.43996176317236

Ag 16.53365895991441 12.49416504094052 20.59363130001339

$Pd_{43}Ag_{158}qL1_1$-614.433
Pd 8.45595094876788 12.49851583725525 16.54441609320337
Pd 12.50003188432440 14.53541156138062 14.50637553322339
Pd 10.48412518427357 18.62357728126307 12.49716651252093
Pd 12.48518807614924 10.45595443182244 10.49675446707719
Pd 12.49531170389798 8.44991310089694 8.44571931107831
Pd 14.50833902634005 6.40315924578236 12.52012107273437
Pd 14.53949208384803 10.45839249772591 16.54771165268118
Pd 18.61855136985940 12.48586802411566 14.54535029023050
Pd 8.44932822070793 12.51316714056303 8.44450225280559
Pd 12.50548312051147 16.56932042371492 12.51083556426579
Pd 12.52155480539488 8.44937728534280 16.55387037118270
Pd 8.44841299441713 8.44897027036606 12.50033061774152
Pd 14.53005682552864 8.43512590938426 14.53649562753567
Pd 14.53801316014758 12.48552910214716 18.61028311251517
Pd 10.45680135076880 12.51059069743007 6.37530893240316
Pd 10.49019735076386 14.51904917342287 16.53171316069603
Pd 12.48919637321756 16.54575329921604 8.42592262784708
Pd 8.43951357465319 14.52988811269658 10.45749226263890
Pd 8.46483263134997 10.47718747134614 14.51380058549557
Pd 16.55038027397601 10.45721360007756 14.54079413712206
Pd 12.49651549135005 16.55349691699875 16.54833733693481
Pd 12.51756121787404 6.39747090190763 14.51281114924397
Pd 12.50279382866566 8.44033231446475 12.50858640609719
Pd 18.61973547890246 10.46276624781643 12.51086464702570
Pd 14.50937007254620 16.54511274166042 14.51495355512768
Pd 6.38362868709198 12.51053026707168 10.46359675517400
Pd 10.49351261504379 10.46727807744819 12.50226054272190
Pd 16.56353324481723 12.49859693218116 8.43704393915184
Pd 6.38013842982765 14.53258203530280 12.48774553666570
Pd 10.48337770671767 12.49690091637941 14.51770623396351
Pd 14.50352661774725 14.54610392745179 12.51557086993513
Pd 10.49126311294691 8.46079021973816 10.49729913967204
Pd 16.55187563823497 12.47607935878646 16.54738228829593
Pd 12.49482206543212 18.61876301656663 10.48751615151633
Pd 8.42536543906450 16.54729913580259 12.49067494022267
Pd 16.54025041793682 14.53203064033446 10.49846679885440
Pd 12.48959491125101 14.53017930780210 6.37760516195866
Pd 10.45593513204576 14.53128609804629 8.43795926346077
Pd 12.48167712286223 12.49831916982204 12.52000447885788
Pd 16.55127918784001 16.55287874366102 12.50065685482679
Pd 12.51387763807862 10.46189246644208 18.61726718129164
Pd 16.55441553067410 8.45178436274887 12.52173785068764
Pd 14.50497594318981 10.46589816783415 8.45744697095433
Ag 20.60458570472422 8.46828072325986 12.51081843725604
Ag 16.55565766917800 12.48306485825117 4.36240952022538
Ag 10.46750618943351 4.29535209951608 10.46724225882451
Ag 10.46292472871001 10.48532810152892 16.55050838680884
Ag 12.51158655615093 12.48991120312763 16.57270844640736
Ag 18.62381008228137 16.59772938554710 14.54927486091298
Ag 10.45835963086805 18.60278552047812 8.42396789237909
Ag 14.53040726601182 12.49961243967154 14.52728366498354
Ag 10.47034059231223 14.53792942137287 12.50923208854186
Ag 18.58239730220169 14.52106998512495 16.55114940087974
Ag 10.48764410755114 6.40101649240040 12.49586748458942
Ag 10.49200724448445 16.56271833074197 14.52338854941274
Ag 14.52874968168950 6.41576218727791 16.55498646461668
Ag 16.53991920290952 20.61889852263836 12.50630664847180
Ag 8.43575984436126 8.41797542673486 8.43895781246843
Ag 10.47007487793473 6.41292637693406 8.45777054223232

Ag	16.56008216350864	8.43721745705666	16.56138455396242
Ag	12.49543637213277	18.57654678515987	6.41228376907127
Ag	16.58742278659856	12.48884097473357	12.52288697907232
Ag	10.45268223526531	18.62328436190020	16.59821858094066
Ag	16.54713184722451	14.52036079075338	18.57963495126504
Ag	14.52819536191046	8.44907559659678	6.39703272067369
Ag	18.59126850752216	12.50028138939637	6.41071381037308
Ag	20.63760302858768	12.51231159908157	8.44823899551641
Ag	12.50910742993554	4.42125793889945	16.52276734160209
Ag	10.47057560196475	16.54893292023074	18.59524846217873
Ag	16.56380609665021	16.58268136954780	16.56654607648592
Ag	18.58523882595102	6.42299502432391	12.51300260219744
Ag	8.44979912214907	18.58430853768969	14.52950171967402
Ag	8.44661097167880	10.47326491143471	6.41208346284908
Ag	8.40897032854457	10.45121374059016	18.62159481655190
Ag	12.50820796722293	4.31591532033366	12.50812407905075
Ag	14.52427605007824	16.55737018086650	10.49425254645189
Ag	14.51369191543028	10.48508725212860	20.62059471780683
Ag	10.48737301719399	20.64204376625511	14.52365681706363
Ag	6.40747640830353	16.54786689732246	14.53167685360081
Ag	12.48019749191787	12.50704734957024	8.41283413576859
Ag	8.44591108975856	14.51147963178700	14.53869507649274
Ag	12.50850546332714	8.46585651489310	20.60440160235400
Ag	20.62462520603974	10.48525805141136	14.51551403580066
Ag	6.41491343903644	10.47369865458743	8.45047444565908
Ag	12.50323016505619	10.45322367791261	14.52924271841076
Ag	10.47100015592264	12.50436878268939	10.46843445859935
Ag	6.41219685915914	14.52502233080713	8.44289168838298
Ag	16.56265425379972	10.47510221660432	10.46319089673906
Ag	4.35651803585609	8.44992952665635	12.50098338736601
Ag	12.51168027101202	12.49109380889223	20.66937765975743
Ag	10.49202738126981	4.37905013187749	14.51095400706866
Ag	12.49998909233349	6.42392455303705	6.41678983312930
Ag	18.59246389927530	8.44716051115572	10.48111624098672
Ag	16.55237367499026	14.54226105939402	14.51425987261381
Ag	12.49764732830986	14.53246508155764	18.61191087863165
Ag	16.60425988178201	14.54719359329157	6.37066716196284
Ag	8.47950601582930	20.58307183691152	12.50189113101188
Ag	6.41509684141681	16.55667023505939	10.46835558168120
Ag	14.51228732859426	4.37772273483720	14.51504572850756
Ag	10.47443873047403	14.53366165170539	20.70450733290769
Ag	14.51759401229958	18.60409945410163	12.50433339953253
Ag	14.51232496863075	14.50781397872258	4.36022017328890
Ag	8.45674539108122	14.53052931435734	18.59072767598673
Ag	4.36546910674206	12.48179748557870	16.54614729132651
Ag	10.48005163204840	8.45132781990549	18.58583720690059
Ag	8.43916598632472	14.52808607677678	6.40822227631690
Ag	14.53457192574757	10.46252226074356	4.29089692393595
Ag	10.48120235257635	6.42397862597357	16.54610963166774
Ag	16.54529756569438	18.59161902766821	14.53009600088832
Ag	6.38900991984589	12.49075615127820	14.53215488941813
Ag	6.38472693846879	10.46541465566713	12.50292278966589
Ag	18.58370228498159	18.57535624499319	12.49829810336152
Ag	18.60665484388674	12.50830017060931	10.46618390166826
Ag	8.42598586430993	12.50553057917201	12.49239703970457
Ag	8.45651788489962	12.51361601324342	20.63433967859152
Ag	12.49192413134987	16.54911122771178	20.64024689786447
Ag	16.55196711855747	6.41753190364370	14.53086854866480
Ag	4.40498042279669	12.50479538757543	8.46317186327920
Ag	10.48584322526436	10.45656492421540	8.44634667340646
Ag	14.54801948666988	16.59764553464660	18.62566124027152
Ag	4.29364210466167	10.46076996335182	14.52876062676948

Ag	18.58394494403351	12.48659932348574	18.58466058895551
Ag	12.50375098784947	18.60657106886815	14.51599598393900
Ag	14.52991696772515	16.55079443374926	6.40202091154500
Ag	6.40095806170030	8.45105140914099	14.52878402603563
Ag	10.47658125205758	16.57527913378917	10.48369671459874
Ag	10.44811282379055	8.39925087047595	6.36726560164231
Ag	20.70751449764607	14.53235011508502	10.46873190419119
Ag	12.50221862754534	18.57574896646558	18.58701357389636
Ag	14.52359966592221	10.44492859981620	12.51741180139808
Ag	14.50875455496763	4.38134403388429	10.49250456641979
Ag	12.50086944809343	20.57788065376103	8.48185541087322
Ag	20.63656481622622	14.50919896731101	14.51516419702062
Ag	16.60284920508608	18.62528794768549	10.44882438402472
Ag	12.48791359327740	4.38395888571902	8.45953683833274
Ag	8.45655517823176	12.50461183342502	4.39996679370459
Ag	8.45887246373333	4.38641352263912	12.48818303138146
Ag	14.52325419528615	20.63992447845493	10.48827306618428
Ag	14.54596349657747	6.38282194294378	8.39944084788331
Ag	14.52963509986444	18.58141713181686	8.45210407597432
Ag	16.54860478625355	10.46635499255873	6.40396622066590
Ag	12.50183907565970	10.46666330957868	6.38269664011855
Ag	12.49465503162025	14.54610648833833	10.47666178229589
Ag	10.47335178120251	8.43912252142943	14.50409092834487
Ag	10.47013634099736	12.50600138022999	18.60738567287438
Ag	18.59066051274239	8.44584111000399	14.53218576681730
Ag	6.36552305450271	8.39827097987772	10.44881239417161
Ag	12.51008788834026	6.42159251798217	18.58778943248084
Ag	12.50690365928003	20.61847633915141	16.54097740547879
Ag	10.48590415527958	14.51190902214888	4.37611037058067
Ag	14.51081364802949	14.54027612521177	16.54992050397371
Ag	10.46678255878544	16.55403380893523	6.41404260210838
Ag	8.40245102950563	6.38417908911650	14.54405628612972
Ag	16.56315198305455	8.43386993863560	8.41595128565356
Ag	14.53962534191834	20.70088238549356	14.53917172173012
Ag	16.51900224211106	4.42760720208273	12.50958981890035
Ag	20.66768643283345	12.49154830033386	12.50970082115962
Ag	18.59133443097404	10.46431542747295	16.56139512866482
Ag	8.44845951087481	10.45837212496487	10.48955345673078
Ag	8.42048843115279	18.60397593363862	10.46047979219527
Ag	14.52389326083625	18.58736614030918	16.54430419020881
Ag	4.36181882238099	14.50934962066343	14.50773036929887
Ag	18.59979861524597	16.55167923903355	10.47029871394172
Ag	12.49429191246850	6.40315197769324	10.48576070550145
Ag	12.49552349466160	20.68708353032125	12.49543934547767
Ag	14.53577995739448	12.48946156987367	6.39013536934337
Ag	18.61472815555283	14.53172995716042	12.49881367554416
Ag	18.62625388400859	10.45353382867757	8.40113780060501
Ag	4.39861743238892	16.53168432008059	12.50171536043657
Ag	16.55733805375067	10.46768650730696	18.58690788281227
Ag	14.51420234755997	12.49654431252250	10.48884321523595
Ag	14.51217910142932	14.50868241788905	20.63062180385254
Ag	12.49351756331987	12.50321315985662	4.32827239876224
Ag	6.41180033503543	12.50611964886315	6.41257259096610
Ag	6.41327811044677	6.42365024376632	12.49670693141071
Ag	20.63828879384460	10.49077341522782	10.49271025327348
Ag	6.40935340368838	18.57731402826255	12.49892330058055
Ag	14.53081140385145	8.44401566578617	18.58902425451763
Ag	16.54345211786816	6.42740485745447	10.48139236492906
Ag	6.37577104724120	14.54915193812693	16.59567410768559
Ag	6.42778384365965	12.49904860219461	18.57357502165051
Ag	16.58705277979850	16.56605714264062	8.43645737347857
Ag	20.63561741404134	16.54577117914334	12.49770611076633

Ag 8.41894022358773 8.43644518000655 16.56198326665249
Ag 18.59712605040777 14.53266019519285 8.45177497572134
Ag 8.43812102428617 16.56266050114822 16.58385629745753
Ag 4.38118823951081 14.51266934379361 10.48839396952039
Ag 4.32708698058371 12.50354276945710 12.49146991665831
Ag 12.49581851508599 8.45384132470167 4.36125629922364
Ag 14.53805589417651 14.52162761093583 8.43407805106181
Ag 10.49740572467304 10.48947088663119 20.63601675538379
Ag 12.49891962905904 16.52849784107488 4.39997002394359
Ag 10.48353317993963 20.61047858167558 10.48584445902408
Ag 8.45900584034172 6.41744677181608 10.47679824198184
Ag 4.36579753037714 10.48503170256179 10.48684040155155
Ag 20.59677902375680 12.49099871195124 16.54214334898971
Ag 10.48417427199213 10.48507403844375 4.35902026677969
Ag 14.50048275006311 8.44410031610571 10.47294569693197
Ag 6.40897970693766 10.46743695157021 16.54473903393098
Ag 8.42285712858911 16.56744826338261 8.42478333226867
Ag 16.53586668173569 12.49123033529701 20.59205991418344

$Pd_{43}Ag_{158}$LEH-613.976

Pd 8.45620272708675 12.58634282147702 16.66217978868712
Pd 12.53711458024853 14.63468719154769 14.63609160382629
Pd 10.49730626871012 14.63391090422686 12.60217499105598
Pd 10.47877373817165 18.74301867118017 12.57965182979674
Pd 10.47353242158997 6.47362068584523 12.57069793497868
Pd 12.52852042044529 8.51609388018739 8.54682366989266
Pd 14.58714604521730 6.46376625261688 12.57288809102295
Pd 8.46506398833461 12.58410846723788 8.54639275476841
Pd 10.48424440311972 16.65373978229157 10.56104520961855
Pd 12.52550028075653 8.52733611872320 16.66531943354563
Pd 14.58642025088167 16.65514527830382 10.56225865736591
Pd 8.46667812470830 8.52602920373173 12.59321499140503
Pd 8.47744742603769 14.65112184510680 14.64219550515924
Pd 14.57215733583172 12.58479153502435 18.75273460753775
Pd 10.48414664149443 12.58407120915933 6.48285756446730
Pd 12.53749081742434 10.56550820114639 14.64773053566034
Pd 10.50260868299380 12.61375781994781 10.57467452098973
Pd 16.59874769779723 14.64851509080692 14.64111932350081
Pd 12.52924548427097 14.63299477910350 18.74080242668203
Pd 12.53195988015635 16.67562533945099 8.54851747549536
Pd 14.57823204907909 18.73723739956997 12.57991121937335
Pd 8.47753827726809 10.53893829166727 14.63701581092741
Pd 16.60097578449808 10.54639146402578 14.64984529535977
Pd 6.41820234664809 10.53880476099052 12.61568803898533
Pd 12.53161307389674 16.66366743216678 16.66718216040959
Pd 12.52489752976294 6.47019146573423 14.61748827530903
Pd 18.67382573299598 12.58666768774563 10.57431272334646
Pd 14.57439506415945 12.61380057135838 10.57175993816777
Pd 18.67308161804210 10.53892245195583 12.61558987819093
Pd 6.40784868358930 12.58665118921776 10.57567550410602
Pd 12.52804413268414 18.73671881978940 14.61975155765829
Pd 16.60593085828942 12.59144364939149 8.54290949713104
Pd 6.39568107178509 14.65028435174805 12.61105871422306
Pd 14.57906902066061 14.63099568081539 12.60198138713188
Pd 10.47979639074256 8.53779303268951 10.56207307591112
Pd 12.52813808605802 10.54329628287534 6.47354681740770
Pd 16.61842839933228 12.58958818281314 16.66328890355340
Pd 10.48749744867904 12.58347588877154 18.75414124148376
Pd 14.57780451341701 12.58667988692487 6.48603165607600
Pd 12.53104664375586 14.62551568687298 6.46891593124949
Pd 18.67987114549406 14.64282874525130 12.61067243304386
Pd 12.52736958629700 10.54318816533587 18.73574249064611

Pd	14.58528794586111	8.53314720797462	10.54939415756626
Ag	20.62965918452691	8.52736028488686	12.58822275198635
Ag	16.55683499761988	12.57645100501584	4.51917036346256
Ag	10.51306357416392	4.43461740343488	10.56979267596017
Ag	10.50969487891109	10.56576851433428	16.66889851386304
Ag	12.53388208275493	12.59877684756766	16.73141656463103
Ag	18.65289378905105	16.66042529572605	14.64581271113551
Ag	10.49659770479415	18.70510970232511	8.53342121855279
Ag	14.58065727977569	12.61498597022542	14.64429401717276
Ag	18.64245250850084	14.61828085822909	16.66264301744570
Ag	10.52247358862076	16.66292413913290	14.62189498803849
Ag	14.57138476383306	6.48406153024168	16.67079573810258
Ag	12.53736844995160	10.55915468217547	10.56557934895034
Ag	16.57565532865045	20.69291286022423	12.59168623050224
Ag	8.44380766914985	8.49355461254264	8.50829167676959
Ag	10.49351718163364	6.47939911318650	8.52708387045057
Ag	16.62646076025943	8.49760664864946	16.67443773557955
Ag	12.52686076798298	18.71177260189706	6.52568956780679
Ag	16.64261832256023	12.60920763900099	12.61261103349801
Ag	14.55492392631548	10.56527803750755	16.67277862682709
Ag	18.63656867688752	12.58492581353774	14.61845014237837
Ag	10.48540108477794	18.70142758703953	16.66549894940974
Ag	16.61011342790493	14.61504667470048	18.71095669127408
Ag	14.56401212265122	8.50213291513165	6.50226604847845
Ag	18.61997505624910	12.57717208571934	6.53102410181304
Ag	20.63339784423497	12.57893225197401	8.58210577082406
Ag	12.52412609928790	4.51300799385834	16.61401460371567
Ag	10.48255807747073	16.66367809558378	18.71623248504658
Ag	16.62528165636465	16.68032024933589	16.67617721795846
Ag	18.65230288621093	6.46665946121646	12.58725046093762
Ag	8.42835019474131	18.73305506892166	14.66352567047536
Ag	8.45396786326679	10.53105816602579	6.49308691915109
Ag	8.45166204235872	10.54658146814124	18.70493085231948
Ag	12.52313127764400	4.45713871323199	12.58858152221584
Ag	14.54575209328927	10.56341205717178	20.73010863044178
Ag	10.50876184829559	20.71687369650871	14.59090435235633
Ag	6.41611457729839	16.66160031260155	14.64598223883198
Ag	12.53529977354622	12.59528220772395	8.49132247828470
Ag	14.54599045388785	8.52020187067876	14.63176727549684
Ag	12.52315630937428	8.56522951509488	20.70831454936210
Ag	20.70705121826882	10.56901888129696	14.60935960388634
Ag	6.42452133154706	10.53460619703517	8.52584955659590
Ag	6.42587572278591	14.62885132971335	8.53030020849106
Ag	10.51312109892966	14.62514029840798	16.68175462022139
Ag	16.61317023456111	10.58789052221732	10.57097021159183
Ag	4.43132208401480	8.55250215877419	12.59306544856920
Ag	12.52860176931928	12.58235590157379	20.71954275543018
Ag	10.50993386131365	4.49793729767997	14.58722319522862
Ag	12.52846625100634	6.47065982793550	6.51947871012550
Ag	18.66567191207999	8.48026400462692	10.52596156185672
Ag	16.60812841282997	14.63162854842269	6.48920787497271
Ag	8.47245140621567	20.69659915160528	12.59145891238660
Ag	6.39952736539546	16.69625755858141	10.52376476493349
Ag	14.53854659696903	4.49910231461013	14.58583404613207
Ag	10.50711272761435	14.59885908160456	20.72986255141683
Ag	14.54679192230499	14.59856720448834	4.49184083268244
Ag	8.44899835117936	14.61524807702873	18.70730634449508
Ag	8.46851310906192	14.61093924035688	10.57780945894331
Ag	4.39614593696779	12.58293543478614	16.63163173450296
Ag	10.47907137396876	8.51061595532331	18.71935934916683
Ag	8.45221160326407	14.62692417385852	6.49480237742312
Ag	14.54311818457994	10.56676012317540	4.49735287222893

Ag	10.48036848164535	6.48569221041747	16.67016970590219
Ag	16.62716221962695	18.72772064485692	14.65887737849753
Ag	6.44582144664071	12.58888418622945	14.61712848992713
Ag	18.63589459857069	18.69993704254864	12.58746806793535
Ag	8.43816366482233	12.61452021300393	12.61166133838818
Ag	8.47095259608085	12.57437125281152	20.69970509263640
Ag	12.52285410819819	16.61207676019668	20.70511185145655
Ag	16.63783256694274	6.45727858404220	14.66010530187860
Ag	4.43458634212999	12.57955341435980	8.59077153135793
Ag	10.50210819845450	10.57478192752146	8.54379493913571
Ag	14.57402141550241	16.66393111055030	18.71834865521449
Ag	4.38239764666684	10.57631150222612	14.61294290318483
Ag	14.54412401246298	16.66119235455352	14.61984139788246
Ag	18.64876065688398	12.57720942990327	18.67823606836171
Ag	14.55808417200611	16.66808985782302	6.50129395325263
Ag	6.43909860964712	8.53257214158788	14.62586552095439
Ag	12.53349162991300	16.68651374442647	12.59444247939925
Ag	10.49781476752039	10.55807492647716	12.60696038240668
Ag	10.49415536155037	8.50409962606749	6.49870480579632
Ag	20.64914458733406	14.60049104919653	10.59693362182999
Ag	12.52589544180328	18.67707410534564	18.67621868479220
Ag	14.56827678406039	10.57097974475576	12.60743853606568
Ag	14.53911258792381	4.43029774475181	10.57466567805894
Ag	12.52270886331150	20.74611292776399	8.55460743853177
Ag	20.70296906137763	14.59114819764649	14.60647648348121
Ag	16.59563362252439	18.71404173667669	10.53952352528666
Ag	12.52434956348632	4.45546029192318	8.56298284762775
Ag	8.49184323523136	12.57590540227841	4.52012415966954
Ag	8.47818175549649	4.50736860424622	12.58252263254688
Ag	14.53215710506553	20.76161302998517	10.57570997887489
Ag	10.49414308220626	12.61430741311319	14.64217825544971
Ag	14.56328575906464	6.47516621986047	8.52614156398878
Ag	14.55874379242838	18.70498608344953	8.53334406720542
Ag	16.60814743279539	10.53167118991227	6.49386381993188
Ag	12.53744033432722	14.64249939152625	10.57677609854793
Ag	10.51273901054260	8.52761763875586	14.61987333904614
Ag	18.65418788457152	8.51426827793228	14.64252118120665
Ag	12.52670602743383	18.69374702264126	10.58291636895340
Ag	6.43038673266366	8.50978110257612	10.54965192623536
Ag	12.52790426789178	6.51057582276226	18.68252892735112
Ag	12.52346439949198	20.69377824596995	16.61594532605629
Ag	10.50879836876922	14.59653819285294	4.49482333091829
Ag	14.55324752827062	14.62543518492577	16.68280562224132
Ag	10.49984218782348	16.66400522029653	6.50545509876243
Ag	8.44627709175747	6.49078827689718	14.63102928941883
Ag	16.62104125574479	8.49074799132070	8.51632109854717
Ag	14.53910548687024	20.71183211814213	14.58886304072657
Ag	16.57982628581120	4.50156831572815	12.58986189018273
Ag	8.48384954463015	16.65050623447709	12.59719798674577
Ag	20.69704565215740	12.58089744960848	12.59010454963484
Ag	18.65212138799584	10.54374946648764	16.66565415253393
Ag	8.46946855511925	10.57676119872998	10.58461876845857
Ag	8.46262898133524	18.71540684854589	10.53903732134539
Ag	14.56974185744938	18.69879859114537	16.66484246166486
Ag	4.37361113662187	14.59534786036947	14.60897588731760
Ag	16.60729346967306	14.61064201209096	10.57790222052775
Ag	18.66923018980624	16.69633504573644	10.52314760676788
Ag	12.52912158788194	6.50572641067153	10.57334009866036
Ag	12.52413995909114	20.76059634000064	12.59342260853932
Ag	18.64942597672575	10.53391494918255	8.52160863512593
Ag	4.42986448673293	16.63902412547809	12.58884802097017
Ag	16.60877184772374	10.54756369311726	18.70240419886514

Ag 12.53899756172737 8.48610422562256 12.59086601182051
Ag 14.54556418346882 14.60003626873696 20.73235805939988
Ag 10.50537710811999 14.61526784693786 8.53436360209292
Ag 12.52963439317706 12.58246667838335 4.51133388453276
Ag 6.43808019827138 12.57779746222517 6.53293338431929
Ag 6.43541966563806 6.50624813652638 12.58368049664167
Ag 20.64641529844318 10.56221020072532 10.59823109249121
Ag 6.42623011246775 18.69726188470768 12.58755670417693
Ag 14.57348467949814 8.51049702310166 18.71552934774637
Ag 16.60140622311713 6.47228313874255 10.53733441760153
Ag 6.42623823575709 14.61873653356026 16.66173749653132
Ag 12.53590356015909 12.63017908180527 12.60830441064862
Ag 6.41656159835731 12.57772312929524 18.67723048791472
Ag 16.61807095095973 16.68884432058282 8.51831874227880
Ag 20.62999806237711 16.64324410751740 12.58675674620991
Ag 8.43847560028021 8.49892178890311 16.68147891661510
Ag 18.64161956344440 14.63261455117844 8.52660340633708
Ag 8.43765455547772 16.67945601451381 16.67436025092575
Ag 16.58733859606172 16.64786849278427 12.59719527899126
Ag 4.42136935259100 14.59963797930819 10.60305135869032
Ag 4.39332677350419 12.58092661194157 12.59468338446276
Ag 12.52655057441284 8.53276133119020 4.51945588303510
Ag 14.56363621330210 14.61756834584192 8.53323010013540
Ag 10.50641401950207 10.56455257399279 20.73649705529111
Ag 12.52547219147418 16.62886048347063 4.52120666578773
Ag 10.51224847859102 20.76880382130871 10.57081431164533
Ag 8.47074844278665 6.50148077005450 10.54825288828197
Ag 16.60098820973100 8.53216816083636 12.59568440228856
Ag 4.42935585924663 10.56991945361384 10.60315801961948
Ag 20.67845983095179 12.58123950643547 16.63378905401726
Ag 10.51016486530525 10.56623506919746 4.48927196594819
Ag 14.56659395660639 10.57602380083809 8.53992934147454
Ag 6.42312010425193 10.54555720320586 16.66244558986159
Ag 8.44567876930285 16.68729867342856 8.52146701934332
Ag 16.57737033009528 12.57440702460750 20.70146578507500

Pd$_{110}$Ag$_{295}$L1$_1$-1323.751

Pd 11.97276502642989 11.97275610475088 5.85898921594414
Pd 14.00420617225081 9.95227198142677 5.85889718185773
Pd 9.95228950563719 14.00419183896023 5.85891113728875
Pd 16.05081866506100 16.05080815389067 5.87043978562304
Pd 18.07669525415370 14.01533256779789 5.86737970016540
Pd 14.01534548708151 18.07668746527159 5.86739449051819
Pd 11.95778209918362 7.91790857902182 9.94430167397595
Pd 13.99582938450526 5.85887586304036 9.95226109948506
Pd 7.91787999475470 11.95777702322497 9.94430799372609
Pd 9.93564819687372 9.93566692123758 9.94252204412720
Pd 14.00051215359812 11.96839436824172 7.92187923100238
Pd 16.04225840299720 9.94430040194724 7.91790366008148
Pd 16.02878192160808 11.97722627875917 9.93804045562471
Pd 18.06436944790712 9.94252735725072 9.93564020656556
Pd 5.85886791381868 13.99582754316723 9.95227974529158
Pd 9.94431897582064 16.04224725192086 7.91792507182763
Pd 11.96839392254626 14.00050838453615 7.92188608511702
Pd 11.97721854147232 16.02879163807907 9.93806704469977
Pd 13.99943528874885 13.99945567902130 9.92377949742527
Pd 18.06135568831494 16.04825457195120 7.92018762239624
Pd 20.08993784368512 14.01374885925430 7.91007081072992
Pd 20.07981438557184 16.04825748791330 9.93863743308197
Pd 22.13263157650768 14.01534867895529 9.92330256488287
Pd 9.94253714433778 18.06437515910556 9.93567518689293
Pd 14.01374653013396 20.08995010743733 7.91007574324383

Pd	16.04825733512737	18.06137771914225	7.92019611209338
Pd	16.04825356983911	20.07986161217583	9.93864788144546
Pd	18.06878896711930	18.06883298956151	9.93120968012834
Pd	14.01534358996848	22.13264624520700	9.92328838600498
Pd	7.91002449751139	7.91002473916152	14.01371048474966
Pd	9.92330624959655	5.86734477190459	14.01531085165130
Pd	13.99952873912979	7.92185939012706	11.96838386029892
Pd	16.02726557977951	5.85893750604821	11.97273819411623
Pd	16.03163601439183	7.92184048585457	14.00049983151133
Pd	18.04772736112900	5.85885308045193	14.00418326605867
Pd	5.86732062267570	9.92325820980051	14.01531008412635
Pd	9.93801904166044	11.97126681383824	11.97721028859056
Pd	11.97126153707019	9.93804194998026	11.97720892767806
Pd	11.96603416963704	11.96604233238896	13.99982821718849
Pd	14.00058641170684	9.92376661066679	13.99944504103744
Pd	18.06198136764376	11.97723291325856	11.97125521265060
Pd	20.08208147028390	9.94429547948925	11.95776007877971
Pd	20.07811884004336	11.96838206119672	13.99952621808108
Pd	22.14106932653866	9.95224647526959	13.99579895638503
Pd	5.85893140377379	16.02726329485604	11.97274587155815
Pd	7.92183279763882	13.99952486836520	11.96838447956202
Pd	7.92182731240187	16.03165178303839	14.00051098660031
Pd	9.92375679055013	14.00060293174607	13.99944399672707
Pd	13.99980045717268	16.03401095597202	11.96607787243734
Pd	16.03395953718103	13.99984911161339	11.96606019709906
Pd	16.03395349818170	16.03402516808534	14.00022433437254
Pd	18.07623533549633	13.99947175881463	14.00057551289549
Pd	22.12956262016064	16.05082829554544	11.94917102020822
Pd	5.85883629535569	18.04773970932869	14.00419482311494
Pd	9.94430684087248	20.08213176674379	11.95777417405508
Pd	11.97721750030397	18.06201660692903	11.97127272563927
Pd	11.96837682814530	20.07819633427892	13.99952530192214
Pd	13.99942539570322	18.07630833007214	14.00060004987427
Pd	18.06134888720032	20.07986004172887	11.95177229366090
Pd	20.07979695095809	18.06138475490362	11.95176920339289
Pd	20.08991088041607	20.08995665369797	13.98626746507064
Pd	22.13260311282949	18.07668226587553	13.98465749192579
Pd	9.95226323885161	22.14114047678119	13.99580070614431
Pd	16.05082358121162	22.12959297253730	11.94918974084922
Pd	18.07670811209595	22.13265140263157	13.98466563685313
Pd	9.93862862524512	7.92012902331556	16.04823102684008
Pd	11.94921386371474	5.87037242839457	16.05079512498367
Pd	11.95178314647099	7.92011634909509	18.06136055148744
Pd	13.98469139899742	5.86732130208344	18.07669966113656
Pd	18.05570287637395	7.91787414396448	16.04224465095621
Pd	5.87036392341171	11.94918449389959	16.05079746149491
Pd	7.92011588291765	9.93862077581449	16.04822595790754
Pd	7.92012229896074	11.95178271981049	18.06135348238166
Pd	9.93119531467083	9.93119603541953	18.06879408418826
Pd	14.00020821329129	11.96603950910057	16.03399462155159
Pd	16.02281503235595	9.93802534523445	16.02877068339087
Pd	16.02281061858353	11.97125165362314	18.06199509193992
Pd	18.05747184885847	9.93563976977486	18.06435747647116
Pd	22.14097118747598	11.97273755720839	16.02724959847319
Pd	5.86732270357885	13.98468349285771	18.07669726921062
Pd	9.93802279889959	16.02283742311366	16.02876620016529
Pd	11.96604025244296	14.00023237883212	16.03398679604771
Pd	11.97125005900451	16.02283512191493	18.06197973149668
Pd	14.00058662890065	14.00060305674850	18.07625864840254
Pd	18.06196470848673	16.02879378823216	16.02280933242253
Pd	20.07811147784446	14.00050163378153	16.03163509904371
Pd	20.08206484988348	16.04224810985923	18.05570317531881

Pd	22.14105288066928	14.00418167601455	18.04771279614057
Pd	7.91787308163356	18.05572254199659	16.04223823160412
Pd	9.93564655440813	18.05750129649029	18.06434965982700
Pd	14.00049736299628	20.07819534775234	16.03163919378113
Pd	16.02876330452093	18.06203061603273	16.02281900171263
Pd	16.04223793762251	20.08213597432428	18.05569851324215
Pd	18.06433575705875	18.06438378812102	18.05747364193714
Pd	11.97274278731894	22.14105270224854	16.02725947913386
Pd	14.00419926242030	22.14115173785224	18.04771904134549
Pd	13.98629463470581	7.91001231875465	20.08995798802610
Pd	9.93863162677625	11.95178105357657	20.07982931296107
Pd	11.95178555128529	9.93861433343469	20.07984645481712
Pd	11.94920813429503	11.94918222965888	22.12957064324222
Pd	13.98468315751744	9.92325362635880	22.13263033999453
Pd	18.05569244874225	11.95776281044691	20.08211300713598
Pd	7.91003095172009	13.98629210700865	20.08993666671863
Pd	9.92329833638075	13.98467378950562	22.13260629784297
Pd	13.99950850503688	16.03165086826714	20.07814714323481
Pd	16.03162534860576	13.99950980830843	20.07815037781163
Pd	16.02724990422432	16.02726029248519	22.14102613903150
Pd	18.04771281648193	13.99582131328024	22.14109792685965
Pd	11.95777110074755	18.05572102399183	20.08210312251562
Pd	13.99581007107098	18.04774607603159	22.14111771135590
Ag	11.92393435806859	7.84455619934043	5.81666382968053
Ag	14.00000843468159	5.83865969896706	5.83866945339311
Ag	13.99931016134383	7.94202941597368	3.90158177872772
Ag	16.04816967084884	7.90584610814463	5.87553174629628
Ag	7.84456777111445	11.92390134088121	5.81668092823799
Ag	9.88740322489373	9.88740881846252	5.81704137983093
Ag	9.95142172448215	11.97742192079007	3.87480576222162
Ag	11.97743065870741	9.95141102915270	3.87479443023270
Ag	13.98847556326413	11.96780088059810	3.78913358839114
Ag	16.02832346784304	9.93942989593854	3.82634333450254
Ag	16.02352541671766	11.94097615632945	5.87038274530993
Ag	18.10628821381990	9.88504031397198	5.82093665082925
Ag	18.06136985359844	11.97556350108529	3.85288755887643
Ag	20.11461863765511	11.95112321452569	5.86211792960041
Ag	5.83898755872259	14.00016559339222	5.83885943852456
Ag	7.94209167309871	13.99929259565424	3.90155294278504
Ag	7.90591335702404	16.04821519275528	5.87556153241040
Ag	9.93945732589439	16.02830334861297	3.82636772240512
Ag	11.96782297320545	13.98845979665412	3.78915208413090
Ag	11.94099618302940	16.02351232562292	5.87041128254542
Ag	13.97810534790618	13.97809389104964	5.87282002803250
Ag	14.01339378924362	16.03499833148225	3.84202266088295
Ag	16.03502340352309	14.01337405012707	3.84201271265174
Ag	18.05360591040018	16.03422092263448	3.86160307778500
Ag	20.06466893530110	14.01076901588071	3.89275677085452
Ag	20.09415030938163	16.05114618004369	5.88462193403225
Ag	22.10175845678388	14.01570255177245	5.89822116840678
Ag	9.88506475153888	18.10625917924770	5.82097876933383
Ag	11.97559231090987	18.06134185579922	3.85290513732618
Ag	11.95114725497282	20.11458827803392	5.86214320505668
Ag	14.01078429454268	20.06464624314273	3.89275681657418
Ag	16.03423069542145	18.05358810066658	3.86160503909371
Ag	16.05115274908277	20.09414414692919	5.88461853167797
Ag	18.07786784155011	18.07786645250523	5.87789609714820
Ag	14.01570820971211	22.10174418120828	5.89819922822459
Ag	11.97170164970214	3.82631296852276	9.93941443404484
Ag	14.00071594643080	3.90154941687147	7.94202991582391
Ag	16.02259488915043	3.87475678253255	9.95136900042882
Ag	7.85678903945633	7.85679546087179	9.88234550160630

Ag	9.89373446577763	5.82092322963684	9.88503006586627
Ag	9.92418408024392	7.89624769055791	7.90161870488214
Ag	11.95185811892654	5.87553411968695	7.90584799146779
Ag	14.00001867944861	7.89815482034364	7.89815555622607
Ag	16.07608917466643	5.81666569950555	7.84453864799955
Ag	16.02968049231699	7.86948551556886	9.93553333350121
Ag	18.11261947895181	5.81699599600743	9.88737494200447
Ag	18.07584109555360	7.90161060880471	7.89623497867230
Ag	20.10374900437344	7.90159931902190	9.92415986461648
Ag	3.82633033119821	11.97174175656003	9.93942343165114
Ag	5.82088071226547	9.89372165537662	9.88502876176729
Ag	5.87550409738440	11.95183901557862	7.90583919516093
Ag	7.89623124878314	9.92417962307626	7.90161749742169
Ag	9.93553557745034	11.97036220381510	7.86950598712497
Ag	11.97036297382040	9.93554739151465	7.86950112154370
Ag	11.95924368637660	11.95925166994226	9.93317290275122
Ag	14.00003478332229	9.92760943034611	9.92759403124930
Ag	18.06563719123841	11.94451775372561	7.90422211853727
Ag	20.14320219462956	9.88235122578246	7.85679873342936
Ag	20.09578496935356	11.94451101621639	9.93437789251925
Ag	22.17904084484894	9.88505473890490	9.89372754393154
Ag	22.13788360518415	11.95111787381532	7.88539126846365
Ag	24.14719149642170	11.97557196753950	9.93865239434320
Ag	3.90154920298984	14.00072521859179	7.94198567815323
Ag	3.87472857941815	16.02258203426857	9.95139442816140
Ag	5.81670689697830	16.07607934873792	7.84465989819060
Ag	7.89820600427527	13.99999585135776	7.89823786465070
Ag	7.86950159693750	16.02965910227696	9.93556949932619
Ag	9.92759554753342	14.00002542988178	9.92761162971200
Ag	13.99894578163758	16.03694438955762	7.89257318689507
Ag	16.03693646211660	13.99894629186045	7.89256176906997
Ag	16.02406613055953	16.02408801334927	9.93751139554735
Ag	18.06887114489542	13.99531541594600	9.93115207791629
Ag	22.11538470549777	16.05116578972624	7.90584296175798
Ag	24.10719055220487	14.01076619929953	7.93532916738453
Ag	24.13846095853769	16.03421352293579	9.94640515593608
Ag	5.81700295060446	18.11260254909502	9.88740773276976
Ag	7.90164272418258	18.07583179714054	7.89627467909691
Ag	7.90162919452044	20.10374935875140	9.92418858757293
Ag	9.88238416181201	20.14318664074967	7.85683952114600
Ag	11.94452079903501	18.06562907420612	7.90425090729628
Ag	11.94451509296631	20.09581937876914	9.93440746688104
Ag	13.99530964198039	18.06890919395378	9.93116135427896
Ag	18.07157178521691	20.09641067734163	7.90359327408102
Ag	20.09638223868381	18.07157920437652	7.90359910783980
Ag	20.09638003678780	20.09640621322495	9.92842663517921
Ag	22.12207767490546	18.07784366502528	9.92215541742664
Ag	9.88505206680599	22.17905876551633	9.89374689501405
Ag	11.95113998022299	22.13787497199633	7.88537981452115
Ag	11.97558423824757	24.14712741391417	9.93861915895201
Ag	14.01078028778444	24.10723538066045	7.93526650082208
Ag	16.05115318820987	22.11537892235224	7.90581915210481
Ag	16.03422910418606	24.13839833823530	9.94637211527628
Ag	18.07786539642377	22.12210493643563	9.92212496685993
Ag	7.93532532307767	3.89273052854222	14.01073795735344
Ag	9.93864372964642	3.85283253340368	11.97554612686970
Ag	11.96501738264527	3.84192636934197	14.01334780969636
Ag	14.01157422666958	3.78905992283988	11.96775872100309
Ag	16.03223948868931	3.78904694825042	13.98843244280340
Ag	18.04860701107495	3.87474660076965	11.97739158374690
Ag	20.05795655009095	3.90154058156833	13.99927953746335
Ag	3.89272816363651	7.93521965378152	14.01074297700605

Ag	5.89821960156374	5.89816198131221	14.01567101823114
Ag	5.86205762185844	7.88532390134739	11.95108933531874
Ag	7.88536776910955	5.86207050799844	11.95109407399859
Ag	9.93437637935261	7.90419237862625	11.94447336481052
Ag	11.97650485711303	5.87034257629934	11.94095560638338
Ag	11.96311217630030	7.89252162994290	13.99892254784250
Ag	14.02195078275651	5.87274032355254	13.97806947456819
Ag	18.06447531618287	7.86947614230843	11.97034763165789
Ag	20.15543921653725	5.81663708262381	11.92391004648207
Ag	20.10180034056200	7.89815490381291	14.00000211863360
Ag	22.16126566961277	5.83884205971737	13.99999294953626
Ag	22.12444443333427	7.90584557319722	11.95182677595917
Ag	24.09849923942963	7.94207783068473	14.00072047064543
Ag	3.85279488953738	9.93858834483546	11.97553551968027
Ag	3.84192434200635	11.96496337756180	14.01335355539487
Ag	5.87031716497154	11.97647225188918	11.94097127369868
Ag	7.90416229454287	9.93437451253098	11.94447891779316
Ag	7.89250226619939	11.96309596364226	13.99892432780343
Ag	9.93111547715523	9.93113383566389	13.99528119507102
Ag	14.00003058237596	11.96842730290551	11.96841557114565
Ag	16.04080667965521	9.93318298150469	11.95923016849411
Ag	16.03163113868273	11.96843247430708	14.00001615273217
Ag	18.07242690968501	9.92758653464881	14.00001695460304
Ag	22.12962622152413	11.94101414417323	11.97649225596587
Ag	24.17360381643716	9.93941327949599	11.97168508620904
Ag	24.21080040922143	11.96778580380550	14.01152565355693
Ag	3.78904532178311	14.01156384487288	11.96779179140673
Ag	3.78905689738692	16.03223368727513	13.98843946284212
Ag	5.87273577489416	14.02193415956102	13.97807323049905
Ag	9.93318250519412	16.04080961258705	11.95925201186994
Ag	11.96841936402458	14.00005245029837	11.96842823965303
Ag	11.96841633425842	16.03166351778995	14.00002514227716
Ag	14.00002074229863	14.00004372833716	14.00004288519839
Ag	18.06248761351486	16.02409958853266	11.97595784873678
Ag	20.10743364299623	13.99894519064256	11.96310227052709
Ag	20.10741673941993	16.03693605832994	14.00108753603884
Ag	22.12714937558321	13.97808104163166	14.02193040351931
Ag	24.15792467003881	14.01337512719787	11.96498228682961
Ag	24.15791588590012	16.03500069402513	13.98661023184750
Ag	3.87471440336789	18.04860263283337	11.97740620677471
Ag	3.90147187021367	20.05796408052388	13.99927147301305
Ag	5.81667855944249	20.15532292320464	11.92389095495742
Ag	7.86949366398015	18.06445850536926	11.97037343653429
Ag	7.89825134136021	20.10174213752703	14.00000237107953
Ag	9.92758504932828	18.07246002969780	14.00002411015487
Ag	13.99893542694370	20.10751092518257	11.96310980047879
Ag	16.02405520281524	18.06255531332456	11.97595796904952
Ag	16.03691824310224	20.10750322164411	14.00109133412987
Ag	18.06884726280027	18.06890957378554	14.00473553395096
Ag	22.11533552399596	20.09411423443726	11.94885103513274
Ag	24.13844139132353	18.05357671789668	11.96577935335563
Ag	24.10717363450248	20.06463093180063	13.98921247894326
Ag	5.83912750759260	22.16081071833140	13.99999661324382
Ag	7.90586716484132	22.12446229334490	11.95177101729448
Ag	7.94197831993695	24.09844387011836	14.00069924239409
Ag	9.93940552315734	24.17365451287955	11.97168467703328
Ag	11.94097522638342	22.12965300095703	11.97649014940066
Ag	11.96779495526047	24.21086698810763	14.01153133749509
Ag	13.97808800895158	22.12722687061197	14.02193106395482
Ag	14.01338842757272	24.15797859264888	11.96497033077338
Ag	16.03502654291129	24.15798520925420	13.98661234513269
Ag	18.05361445452726	24.13840409959260	11.96576802427973

Ag	20.09414511040290	22.11538158421472	11.94884827452000
Ag	20.06471119843202	24.10725684300366	13.98921156571277
Ag	22.10176441532348	22.10176304947930	13.98427999106721
Ag	9.94640952760010	3.86156011603525	16.03418292794899
Ag	11.96580334943896	3.86155270236443	18.05358120782868
Ag	13.98665775142816	3.84191427297251	16.03499666803399
Ag	16.02445586478717	3.85278529332503	18.06137520883828
Ag	18.06058416204971	3.82627408167719	16.02830958467232
Ag	5.88465583087770	7.90587525047638	16.05114451812572
Ag	7.90583744495131	5.88458397160749	16.05111968341075
Ag	7.90360296130902	7.90359054624363	18.07151675755744
Ag	9.92215385983699	5.87785796390460	18.07784615933225
Ag	14.00110835554372	7.89250946189331	16.03690969312856
Ag	16.05905877984982	5.87031022138345	16.02351748187047
Ag	16.05553875858999	7.90416782347065	18.06564285164118
Ag	18.11498024038083	5.82087150516890	18.10627927877554
Ag	20.09414616729821	5.87549050352047	16.04815584962054
Ag	20.09839294818564	7.89620358051036	18.07584146732438
Ag	22.18332681905165	7.84455693925130	16.07609765058244
Ag	3.86155240457070	9.94634678239837	16.03419623420092
Ag	3.86154447081196	11.96577867299043	18.05358518232353
Ag	5.87786449413822	9.92214512884008	18.07785499503525
Ag	9.93747665190925	11.97594612573623	16.02407695869726
Ag	11.97594509709577	9.93748290131420	16.02407826745833
Ag	11.97595088634051	11.97595065174097	18.06253382373368
Ag	14.00475422128851	9.93111975487227	18.06888518943697
Ag	18.06686065387246	11.95924247066290	16.04079124142799
Ag	20.13048239881654	9.93551482705480	16.02966706196323
Ag	20.13047748936966	11.97034171625437	18.06448295191032
Ag	22.18295548111941	9.88738627674669	18.11261285024704
Ag	24.12516825639962	9.95140364730053	16.02257131338969
Ag	24.12516632682991	11.97741065168117	18.04857516734024
Ag	3.84192758938908	13.98663429368240	16.03499310477343
Ag	3.85277265166095	16.02444406819251	18.06136927159734
Ag	5.87030963202068	16.05905650812866	16.02351536971542
Ag	7.89249922667848	14.00110670417349	16.03691206731760
Ag	7.90417097113209	16.05553612888280	18.06561717727478
Ag	9.93111971375649	14.00475746188665	18.06887012437290
Ag	14.00002291667032	16.03166795516390	16.03163056105083
Ag	16.03161991848603	14.00005491430618	16.03162338943527
Ag	16.04077925828742	16.04081634981506	18.06684702679401
Ag	18.07241647146806	14.00001868995709	18.07243901149460
Ag	22.12961040207843	16.02351198874057	16.05899138202927
Ag	24.21079682641505	13.98844945357598	16.03218796589134
Ag	24.17360097454543	16.02829616963852	18.06055006792558
Ag	3.82627340888850	18.06060213066051	16.02830563767775
Ag	5.82087677286581	18.11497491076682	18.10625503851410
Ag	5.87547780029459	20.09412105082134	16.04821931439588
Ag	7.89622254863407	20.09839442381326	18.07583226289077
Ag	9.93554726754523	20.13052360403712	16.02966186469142
Ag	11.95922825789894	18.06689240649504	16.04079611031115
Ag	11.97035509559357	20.13052883505682	18.06445492373752
Ag	14.00001038567185	18.07247029433568	18.07242995205631
Ag	18.06561017588240	20.09582428477093	16.05551448807171
Ag	20.09575375401472	18.06563996444452	16.05551617516564
Ag	20.14317187050054	20.14320566164229	18.11763428707022
Ag	22.17902238364390	18.10626525997505	18.11492945897253
Ag	22.13786001847286	20.11459488103973	16.04886092279136
Ag	24.14718003564181	18.06133462477026	16.02441164347312
Ag	7.84465178017907	22.18330100703484	16.07610222271821
Ag	9.88740873719610	22.18301072836898	18.11259638786652
Ag	9.95138711105105	24.12525188530885	16.02256120090567

Ag	11.97742689537698	24.12527519275283	18.04859079005074
Ag	13.98846426932602	24.21087864836596	16.03219669490162
Ag	16.02351890891653	22.12965333093214	16.05903559057953
Ag	16.02831951476186	24.17367694718468	18.06057328135703
Ag	18.10626530009949	22.17907887673896	18.11494723431481
Ag	18.06138433342375	24.14714099434440	16.02441565672556
Ag	20.11462728127142	22.13790918512353	16.04886750281069
Ag	13.98925180288191	3.89270209242712	20.06466993561613
Ag	9.92848301270815	7.90359116832788	20.09637348231583
Ag	11.94888649537195	5.88457450356609	20.09415012704370
Ag	11.94887407645407	7.90579225263679	22.11536576174406
Ag	13.98433510775988	5.89814727322075	22.10176460680659
Ag	16.04891139250626	5.86203112920013	20.11463975602463
Ag	16.04891212744855	7.88530986602267	22.13791550050449
Ag	18.11766037097556	7.85677440393591	20.14320676526251
Ag	5.88458702922766	11.94888214845066	20.09415631696893
Ag	7.90360640547266	9.92847076456548	20.09637027419372
Ag	7.90582387841936	11.94887990038245	22.11535091646981
Ag	9.92214626381382	9.92214775557394	22.12206867033734
Ag	14.00110392414331	11.96309357912135	20.10747420548179
Ag	16.05553399518946	9.93435638619612	20.09581759695606
Ag	16.05900296022422	11.97649027792234	22.12967585398206
Ag	18.11497923032820	9.89371808884255	22.17909491160017
Ag	20.09838842971447	9.92414876457426	20.10377496924301
Ag	20.09416965813389	11.95183711343493	22.12447978730534
Ag	22.18332167863674	11.92391282217573	20.15542408350645
Ag	3.89270007747053	13.98923471165132	20.06468053241988
Ag	5.89819478450146	13.98432355398323	22.10176191392546
Ag	5.86204906818020	16.04889694701757	20.11461846012276
Ag	7.88536306448934	16.04889446448416	22.13788768127999
Ag	9.93438538264334	16.05553825312172	20.09579704240554
Ag	11.96310912609441	14.00110719744383	20.10747045206661
Ag	11.97652152935900	16.05901586433158	22.12966165724842
Ag	14.02194815891101	14.02193661757402	22.12721159439403
Ag	18.06443611581994	16.02965832552501	20.13050304560667
Ag	20.10177903175524	13.99999460930526	20.10181823375362
Ag	20.15535536738261	16.07606600882395	22.18327951735356
Ag	22.16122674341168	14.00012003183425	22.16115913174194
Ag	22.12441894120487	16.04818837575665	20.09411226690563
Ag	24.09840298524522	13.99928294354161	20.05791837835597
Ag	7.85680619672800	18.11764631114093	20.14317742673963
Ag	9.89375412433142	18.11497812159267	22.17906234890001
Ag	9.92417145329895	20.09840356750003	20.10375988122415
Ag	11.95178407677276	20.09414392674623	22.12448048505212
Ag	14.00000985897885	20.10175069384770	20.10173181442876
Ag	16.02964490145468	18.06447672509812	20.13050213436776
Ag	16.07611636436044	20.15534796543995	22.18329712127948
Ag	18.11259492974749	18.11261108361248	22.18297326196379
Ag	18.07582975150362	20.10378050309093	20.09838054648078
Ag	20.10373643875905	18.07585152321970	20.09837894506139
Ag	11.92390570335769	22.18332836744852	20.15533177416386
Ag	14.00001296997607	22.16085239590805	22.16082579223209
Ag	13.99930050744002	24.09848799890848	20.05797352148647
Ag	16.04822271407316	22.12449326594023	20.09410492890903
Ag	13.98925505780797	7.93523032972610	24.10723143252489
Ag	9.94640983573551	11.96578915979421	24.13836514927920
Ag	11.96579906783082	9.94635829497720	24.13836696027613
Ag	13.98664567012726	11.96496338094746	24.15800274907830
Ag	16.02445556472444	9.93859514182361	24.14714691186298
Ag	18.06057713205782	11.97174718072533	24.17364656474647
Ag	7.93530639921293	13.98923916230498	24.10722004875738
Ag	9.93864327272871	16.02442926440199	24.14712582666538

Ag	11.96501423328963	13.98662770649377	24.15798934070156
Ag	14.01156632703804	16.03223364128542	24.21087476682775
Ag	16.03221323199930	14.01156376902135	24.21089447740265
Ag	18.04859293863425	16.02258461046372	24.12523900686700
Ag	20.05800050011867	14.00072633644752	24.09842755373237
Ag	11.97170989958587	18.06060478052575	24.17367119519932
Ag	14.00072436826094	20.05799648926866	24.09846761903380
Ag	16.02258204502613	18.04861132697314	24.12524249840997

$Pd_{110}Ag_{295}qL1_1$-1323.767

Pd	11.97694474908826	11.97515684125742	5.86667613875559
Pd	14.00475256477952	9.94758331515089	5.86095713216095
Pd	9.95959630911236	14.00603302259563	5.86773470021689
Pd	16.03177103223818	16.05040864917797	5.88253233709476
Pd	18.07223502731230	14.01937106111054	5.87177033598357
Pd	14.01673003267441	18.04697017432012	5.88640912966752
Pd	11.95706172674834	7.91821531616905	9.94513892359235
Pd	13.99659969932084	5.85917007053074	9.95301692917699
Pd	7.92041071851651	11.95688774468195	9.94523118297709
Pd	9.93553226226888	9.93403096684835	9.94239231742448
Pd	14.00167656395939	11.96215134856363	7.91950344876605
Pd	16.04225650226112	9.94299958217100	7.91683902937448
Pd	16.02534456918277	11.98123293092254	9.93605268266794
Pd	18.06222727107847	9.94185982245197	9.93275826303013
Pd	5.85944452615949	13.99565585380897	9.95411708515316
Pd	9.93519402527638	16.04541192100834	7.91492243664710
Pd	11.98042361485905	14.01614018096302	7.92287134470068
Pd	11.98539633708732	16.02771149804390	9.92542133292741
Pd	13.99854296219987	14.01151282345466	9.90551265183303
Pd	18.07177755675977	16.04380224525844	7.92070328024924
Pd	20.09021293706535	14.00936813994009	7.90630242392257
Pd	20.08531889959425	16.04565470148557	9.93345344794851
Pd	22.13396107358912	14.01084050146179	9.92230995022624
Pd	9.94421983233248	18.06098940236030	9.93127536745262
Pd	14.00823229711837	20.09390078773397	7.90853057658633
Pd	16.03682566980915	18.04206472321522	7.90960585676090
Pd	16.03640750234090	20.08013642801925	9.91836008608671
Pd	18.07096968130816	18.06120959085786	9.91611727552207
Pd	14.01061430634014	22.13857775954626	9.91826812830947
Pd	7.90980571796703	7.91120223503544	14.01611136403244
Pd	9.92151441810793	5.86852979736842	14.01649121043263
Pd	13.99828926224980	7.92096221205169	11.96818284645586
Pd	16.02830190258596	5.85974167717726	11.97182682710675
Pd	16.02932935768714	7.92339251016831	13.99857839112314
Pd	18.04580760793186	5.85994675325993	14.00468019792366
Pd	5.86801786102733	9.92185938479935	14.01603379534048
Pd	9.93910247778598	11.97221729431500	11.97487295732602
Pd	11.97216034506942	9.93817189325630	11.97645527201278
Pd	11.96540724115514	11.96647035326559	13.99750843926865
Pd	13.99972134910368	9.92430221773762	13.99679002065040
Pd	18.05870238437375	11.97896933812358	11.96874184812671
Pd	20.08046255842057	9.94553797880261	11.95675592980297
Pd	20.07733895039478	11.97113024844244	13.99821988885469
Pd	22.14077572381844	9.95114185751925	13.99596888423407
Pd	5.85888979236998	16.02867102982556	11.97233399916146
Pd	7.92418394486651	13.99897450569806	11.96805073757503
Pd	7.92324529975333	16.03109942332128	13.99862222651728
Pd	9.92279934386355	14.00048109464068	13.99765591989912
Pd	13.99850658094403	16.03403341006070	11.97228784001734
Pd	16.03019356726277	14.00550798574010	11.96235171265827
Pd	16.03441608578283	16.03517358033408	13.99976764416474
Pd	18.07633726722060	14.00099318452940	13.99868114026936

Pd	22.13222149182933	16.04772571077287	11.94997646153866
Pd	5.86067498798354	18.04622272925709	14.00282372521775
Pd	9.94675087336125	20.08151252035786	11.95637668401428
Pd	11.98016476522615	18.05942761241736	11.96720543763987
Pd	11.96959533335481	20.07733395628113	13.99672906257908
Pd	13.99843744816707	18.08008797329028	14.00099884229311
Pd	13.99225324935566	16.03310280096020	7.90318369545342
Pd	20.08963718555855	18.05072614937827	11.94587586290203
Pd	20.11118046136780	20.08520162509858	13.99437042675945
Pd	22.13594557673820	18.07281368784369	13.98558611423277
Pd	9.95340802431079	22.14017057211215	13.99554001938241
Pd	16.03614006594785	22.14113980407906	11.94392734266559
Pd	18.07851390022183	22.15197556194844	13.99853339086965
Pd	9.93809087244804	7.92102199481683	16.04854915434007
Pd	11.94818462452274	5.87232669847360	16.04999792522408
Pd	11.95021509912193	7.92166844142875	18.06080755207798
Pd	13.98307582117533	5.86988948292196	18.07452097768028
Pd	18.05350610978892	7.92047642591972	16.04246534379518
Pd	5.87191393310918	11.94898951867584	16.05139506046814
Pd	7.92003946848411	9.93611958651341	16.04846898510620
Pd	7.92109533574973	11.94985142359185	18.06050698355349
Pd	9.92942078068719	9.92853677125613	18.06817593230879
Pd	13.99951876191062	11.96519514873200	16.03519686093954
Pd	16.02220147847390	9.94020345476171	16.02802748662044
Pd	16.02238590944481	11.97085459998081	18.06238070969669
Pd	18.05835424357990	9.93621761735358	18.06575837459262
Pd	22.14054910683758	11.97071124890618	16.02795627010294
Pd	5.86872220618533	13.98361959076661	18.07474094556683
Pd	9.93753561582745	16.02250769927208	16.02872446007991
Pd	11.96540395044334	13.99813262167113	16.03445301272717
Pd	11.97054286008272	16.02191093406846	18.06264690879452
Pd	13.99924982703316	14.00028027265366	18.08049598290657
Pd	18.06257336792827	16.02552367424575	16.02777004077086
Pd	20.07842926356947	14.00016962652062	16.03119396649789
Pd	20.08278785364384	16.04238351336412	18.05560484985944
Pd	22.13975417212782	14.00365705200752	18.04768601968301
Pd	7.91939906807212	18.05456308716203	16.04229447787296
Pd	9.93424794323684	18.05818656716796	18.06615649174275
Pd	13.99711038284715	20.07959910502446	16.03616954606791
Pd	16.02866231290136	18.06371180352450	16.02516183281308
Pd	16.04408188581269	20.08199991366839	18.05240664481721
Pd	18.06447263594113	18.06585641372835	18.05555250679309
Pd	11.97185152414629	22.14125996379627	16.02905506333672
Pd	14.00357786318888	22.14320606103941	18.04973145468430
Pd	13.98424012686656	7.90976177684306	20.08990817867995
Pd	9.93949745303124	11.94962991636975	20.07880693440677
Pd	11.95020493763698	9.93868794858489	20.07987543729696
Pd	11.94838335824151	11.94744451205436	22.13176136974521
Pd	13.98304088385217	9.92045753481697	22.13489922028409
Pd	18.05448571281328	11.95596341635343	20.08260308570801
Pd	7.91137979093822	13.98313327410758	20.08701423749407
Pd	9.92438358364717	13.98260276155844	22.13339504671016
Pd	13.99805855547101	16.03133925527007	20.07939360684248
Pd	16.02946211711902	13.99779552477174	20.07988259493875
Pd	16.02812870992155	16.02874159995222	22.14217960742629
Pd	18.04591970383692	13.99669730630946	22.14018479607984
Pd	11.95626132433839	18.05551648638979	20.08331712729457
Pd	13.99665088319564	18.04992768983902	22.14177987199844
Ag	11.92304894330890	7.84327263845604	5.81642267230573
Ag	13.99955134498371	5.83723862245742	5.83769614145540
Ag	13.99912020775186	7.93923700274318	3.89929206983303
Ag	16.04887569238605	7.90172600094727	5.87241641282300

Ag	7.84734884033197	11.92338334302671	5.82062665498813
Ag	9.88804989375999	9.88689460314083	5.82016151600779
Ag	9.95586087156375	11.97754271530555	3.88182121398427
Ag	11.97953020399170	9.95376131691866	3.88065400820826
Ag	13.98987889954944	11.97228974102052	3.80254474430016
Ag	16.02722653138424	9.93752214551068	3.82714771110715
Ag	16.01954685200759	11.94272154404442	5.87414330855923
Ag	18.10438531201517	9.88257632044055	5.82144416616458
Ag	18.05491094283148	11.97696380287491	3.85842621882280
Ag	20.10925128701361	11.95088824665921	5.86386785290132
Ag	5.83897378392071	14.00004896676342	5.83852018448396
Ag	7.95140322061180	13.99920370474207	3.90814453470527
Ag	7.90124548720555	16.04942779308866	5.87372341325535
Ag	9.94989886533495	16.02511903067135	3.83799351536145
Ag	11.97286172735244	13.98834392571709	3.79862509269867
Ag	11.95785433751902	16.02561979820314	5.88357046394251
Ag	13.98035041243693	13.99671572200926	5.88604575950180
Ag	14.01049361160771	16.03317529808297	3.82894601791667
Ag	16.02892929269087	14.01517725225141	3.84736765183700
Ag	18.04335617834288	16.03330874082102	3.87492828033998
Ag	20.06019147034496	14.00654117987268	3.89585164012854
Ag	20.09689347674224	16.04899599486479	5.88391082138773
Ag	22.10233557580989	14.01296693356935	5.89174018447077
Ag	9.88441957069933	18.10492405800751	5.82591140494439
Ag	11.97353928434017	18.05647533109087	3.85945341796044
Ag	11.95437239990181	20.10078514984134	5.87200546467117
Ag	14.01084537799202	20.03423869810399	3.90939269432206
Ag	16.03403937504764	18.04513222095359	3.87060128702234
Ag	16.04369678064084	20.08022658551126	5.89748450845970
Ag	18.07409443689062	18.07105679712980	5.88845963070788
Ag	14.01308503060065	22.09940459635927	5.89523386129880
Ag	11.97173751577969	3.82896878270668	9.93911792821976
Ag	14.00055797970067	3.90182023193724	7.94009868938231
Ag	16.02326263114147	3.87674194307806	9.95036135866668
Ag	7.85563980481364	7.85469246378539	9.88147715311285
Ag	9.89084027372029	5.82337897997634	9.88352676130403
Ag	9.92314618846041	7.89517337924581	7.90003731772028
Ag	11.95156729754234	5.87534472754936	7.90528314177227
Ag	14.00057372048681	7.89701796359391	7.89911232744787
Ag	16.07793634998021	5.81460343950610	7.84315374322230
Ag	16.03111035056511	7.86885291154095	9.93558950271709
Ag	18.11474556840766	5.81805388693796	9.88485721579752
Ag	18.07647963423039	7.89808043040990	7.89313988407845
Ag	20.10266489139179	7.90137140042506	9.92320907084554
Ag	3.82749732561645	11.97061444546724	9.93855936375133
Ag	5.82397119956653	9.89202101048312	9.88408654801712
Ag	5.87612477821018	11.95088118171901	7.90540567855714
Ag	7.89856411267968	9.92481948726695	7.90027406379319
Ag	9.94102976705348	11.97202728478279	7.87344543736445
Ag	11.96953310040671	9.93402297938487	7.87024373541843
Ag	11.96280754629539	11.96448040374050	9.92965221839232
Ag	14.00020067878591	9.92813414627208	9.92758430573260
Ag	18.05690248859626	11.94760406205982	7.90591574069013
Ag	20.13733345844950	9.88404951012858	7.85699411195370
Ag	20.09022646983744	11.94488688509394	9.93485663538563
Ag	22.17476287274160	9.88484383045561	9.89365018995988
Ag	22.13469050484617	11.95045435282503	7.88609817943625
Ag	24.14766788628402	11.97370282095502	9.93618435552133
Ag	3.90329607493045	14.00012566350075	7.94249671860939
Ag	3.87660441715441	16.02332114148909	9.95140877281637
Ag	5.81516305212368	16.07765668712191	7.84381178747337
Ag	7.89791594910650	13.99913839024026	7.90009257705852

Ag	7.86723932846018	16.03041971262091	9.93607727327437
Ag	9.93126057819727	14.00409841785721	9.92493796944499
Ag	18.06428540880202	20.09160178371673	11.95927542197900
Ag	16.02461653617324	14.01117498063145	7.89164111011996
Ag	16.01426613204487	16.02428178545442	9.92498825390854
Ag	18.06444182866429	13.99841161328186	9.92975845746729
Ag	22.11464371832146	16.04602563875924	7.90247878449408
Ag	24.10550446648831	14.00773366635333	7.92979237477580
Ag	24.13482172958291	16.03100381067590	9.94489041094256
Ag	5.81684810533122	18.11479949174624	9.88585278561056
Ag	7.89477526667984	18.07867220419400	7.89409785521058
Ag	7.90087449145520	20.10264679997405	9.92274051308006
Ag	9.88603197388530	20.13399938085975	7.86024280842992
Ag	11.95599706896887	18.05072576388689	7.90294384777162
Ag	11.94580007278618	20.09072213216521	9.93324539934426
Ag	13.99446196481298	18.05595914513661	9.91720203251471
Ag	18.06726060485496	20.09171563124761	7.90950039130455
Ag	20.10231283106035	18.07189557250360	7.89860790075951
Ag	20.12012040532193	20.11398173253944	9.90939877660348
Ag	22.12408370631820	18.07655373895282	9.92001238102871
Ag	9.88438781524780	22.17597768972870	9.89283937262073
Ag	11.94870255721806	22.13956511119004	7.88331814079109
Ag	11.97395049000885	24.14879640295616	9.93356289651964
Ag	14.00619602890325	24.11065672942953	7.92454532941487
Ag	16.04712360354213	22.11770106814805	7.89825063732374
Ag	16.02750661558906	24.14285905318998	9.94112415452004
Ag	18.08680021471451	22.14419000586122	9.90244712967697
Ag	7.93305132459514	3.89270379798817	14.01011674741160
Ag	9.93755829934745	3.85748624891153	11.97626510279991
Ag	11.96360632320666	3.84488946995963	14.01333357630730
Ag	14.01106802591069	3.79075238759609	11.96768624822298
Ag	16.03177073512542	3.78941468133471	13.98844363487944
Ag	18.04931500037592	3.87704094223965	11.97670168282988
Ag	20.05967606180051	3.90456397481467	13.99792512797998
Ag	3.89366718461612	7.93437518529041	14.01004531557369
Ag	5.89771411735845	5.89773301522092	14.01505846634930
Ag	5.86438398705384	7.88594409453748	11.94983369237503
Ag	7.88447725630108	5.86311496979069	11.94988520452484
Ag	9.93484258033188	7.90508814237973	11.94376331865348
Ag	11.97549386470486	5.87193298668662	11.94018310636291
Ag	11.96309812086635	7.89346019822806	13.99734816984503
Ag	14.02090883400366	5.87172479471714	13.97828880758980
Ag	18.06347746225640	7.87041403769383	11.96872321226546
Ag	20.15593283781454	5.81798604005753	11.92205505886758
Ag	20.09995053595085	7.89844700568928	13.99956038474847
Ag	22.16290272066371	5.83744262607661	14.00015614653193
Ag	22.12448967007030	7.90573384516438	11.95140648984973
Ag	24.09964741300371	7.93983168978537	13.99978856836283
Ag	3.85653158711498	9.93768439096791	11.97518602537567
Ag	3.84427643559012	11.96365334349866	14.01249251704055
Ag	5.87153691392233	11.97628223524331	11.93982246001762
Ag	7.90658008064784	9.93541996788898	11.94372006184620
Ag	7.89323446586856	11.96339627423873	13.99709532228041
Ag	9.93223482954267	9.93293746117378	13.99350970258469
Ag	13.99893078577070	11.97235716827271	11.96191910388277
Ag	16.03923396779308	9.93496247608275	11.95743079692862
Ag	16.03200530435934	11.96916117682210	13.99716372126803
Ag	18.07092126039528	9.92962507910043	13.99856469393647
Ag	22.12763911285722	11.94048971535889	11.97535904813425
Ag	24.17143136231337	9.93828847569943	11.97201034726688
Ag	24.20987118411119	11.96571682215168	14.01203103890609
Ag	3.78883558252141	14.01143112167982	11.96715261217179

Ag	3.78846719993771	16.03172939172132	13.98734628644788
Ag	5.87168509845492	14.02153495160132	13.97619176161809
Ag	9.93648350048992	16.04006937195324	11.95552179230744
Ag	11.96973977535006	14.00411811304434	11.96173962903656
Ag	11.96631871347715	16.03198551503599	14.00099990277967
Ag	13.99897113274072	13.99929674188624	14.00129959804782
Ag	18.05950071497395	16.02898526788097	11.96944547403284
Ag	20.10447038173938	13.99888566946348	11.96242497647862
Ag	20.10825236223381	16.03368429991787	14.00273173524923
Ag	22.12916874060333	13.97651433243194	14.02229128296668
Ag	24.15612368497675	14.00997117574470	11.96420469876317
Ag	24.16060880495945	16.03164053851087	13.98683713973473
Ag	3.87648246369252	18.04996473218501	11.97680434240868
Ag	3.90276069849537	20.05884490083675	13.99918429924635
Ag	5.81762833423551	20.15566680627233	11.92279615786759
Ag	7.87213117745704	18.06356876824377	11.96670814722184
Ag	7.89965912408112	20.10032440643864	13.99935217082501
Ag	9.93025220578631	18.07090167733640	13.99738047376848
Ag	14.00108465262067	20.10475155283379	11.95654958355006
Ag	16.01484621199872	18.05403816644961	11.97003832122934
Ag	16.02339253751033	20.11346673215721	14.01461770758907
Ag	18.06995588340867	18.05828857681848	14.01603567959943
Ag	22.11150388345644	20.09712628708177	11.94886522942539
Ag	24.13543255516984	18.05236051464593	11.96677381527413
Ag	24.10658898035419	20.06450770285307	13.99191547097413
Ag	5.83813469791982	22.16239667396527	14.00004670095358
Ag	7.90509510038909	22.12333366763572	11.95035234715011
Ag	7.94006009749960	24.09693063149253	14.00017090514140
Ag	9.93662894571912	24.16938748767511	11.97138780972527
Ag	11.94232266317302	22.12895481708339	11.97344685376353
Ag	11.96603259007829	24.21122430341329	14.01171222312770
Ag	13.97470094349794	22.13177387643802	14.02179140020008
Ag	14.00535578982935	24.16473484875891	11.96370327501580
Ag	16.03260382524155	24.16098147244195	13.98841428295602
Ag	18.05418781397596	24.12808417081963	11.96383028135773
Ag	20.12700857056796	22.14974502167085	11.93675735446370
Ag	20.06557796038848	24.11662436025289	13.99610560026762
Ag	22.10921155405972	22.10536459885137	13.99204583108478
Ag	9.94455357731325	3.86507208027463	16.03346286435755
Ag	11.96615867949805	3.86532414741387	18.05232238802322
Ag	13.98623042827339	3.84330332644593	16.03378185598504
Ag	16.02268472108943	3.85802049318916	18.05940467541794
Ag	18.05970568468516	3.82850277546092	16.02707060836128
Ag	5.88430376704434	7.90306378496004	16.05149889739407
Ag	7.90390986587973	5.88522349251303	16.05073538776175
Ag	7.90152296521413	7.90204491396449	18.07181977502633
Ag	9.92162385768439	5.88150266175970	18.07718037600608
Ag	14.00166354519842	7.89533146285792	16.03432094279698
Ag	16.05864656282625	5.87290687775405	16.02260227808205
Ag	16.05589170809538	7.90710485487287	18.06410777949014
Ag	18.11552233110896	5.82527738878667	18.10657775655942
Ag	20.09526002909655	5.87598580652005	16.04882077393315
Ag	20.09957971187612	7.89627686158678	18.07670464559300
Ag	22.18339264639776	7.84319008303896	16.07602552993647
Ag	3.86566894493467	9.94531214160921	16.03428971263815
Ag	3.86397021364623	11.96485614724412	18.05367775091696
Ag	5.87928516524329	9.92047751788120	18.07757953051165
Ag	9.93816626465453	11.97485994128782	16.02282105201980
Ag	11.97565918404936	9.93956675952970	16.02252776500005
Ag	11.97586959200614	11.97459807137939	18.06104647371535
Ag	14.00417915478872	9.93201087746729	18.06867759526062
Ag	18.06649865096441	11.96033227809575	16.03916400194961

Ag	20.12993580745962	9.93614212873439	16.02969930535016
Ag	20.13032796647916	11.96849069902764	18.06410369356616
Ag	22.18083620804243	9.88479361233195	18.11418243816853
Ag	24.12408880081434	9.94954424140748	16.02296680882211
Ag	24.12290855631727	11.97510454491617	18.04956748314569
Ag	3.84569210643634	13.98525624517936	16.03408142214753
Ag	3.85746583675793	16.02395567898736	18.06057506943164
Ag	5.87218948312989	16.05845009465288	16.02239921955670
Ag	7.89336457063350	14.00137063850239	16.03355863950490
Ag	7.90642031561827	16.05527838169384	18.06344047554176
Ag	9.93276504377200	14.00319627258264	18.06712871571641
Ag	14.00012243080596	16.03277835336154	16.03301366738643
Ag	16.03238174217427	13.99934709028435	16.03340943281626
Ag	16.03910131167796	16.03958101433501	18.06843157528040
Ag	18.07147277652664	13.99843671036668	18.07301725757219
Ag	22.13181569760843	16.02341400688583	16.06014004526759
Ag	24.21196769194051	13.98592579293851	16.03402857420550
Ag	24.17469181879984	16.02571978126155	18.06173367822140
Ag	3.82972296784732	18.06053558729280	16.02651519351402
Ag	5.82390192244131	18.11603030107067	18.10645565009602
Ag	5.87634965399976	20.09559343971665	16.04781809419794
Ag	7.89634488245412	20.09916934599202	18.07659487040418
Ag	9.93658099486055	20.13035409645659	16.02982111182703
Ag	11.95890160668364	18.06629526752269	16.04153788862492
Ag	11.96764506630363	20.13145091840950	18.06560504387702
Ag	13.99834392690605	18.07127276363748	18.07361225451069
Ag	18.06839054397411	20.09904846015450	16.05272351503800
Ag	20.09888998039582	18.06452626124731	16.05862712344210
Ag	20.14374135150496	20.14402011150058	18.11755475877086
Ag	22.17923248069204	18.10716545515204	18.11760473921040
Ag	22.14707382860832	20.11447270689742	16.05609045869852
Ag	24.14968797498650	18.06183052767327	16.02491063184991
Ag	7.84391791013027	22.18291768383264	16.07654801224875
Ag	9.88487742462833	22.18163843384119	18.11449529655812
Ag	9.95034265406242	24.12293995558119	16.02318100642596
Ag	11.97582069291436	24.12435586485943	18.05134496472382
Ag	13.98567298623177	24.21296665693093	16.03470992993971
Ag	16.02342469780415	22.13638058133832	16.06223731850459
Ag	16.02878454114892	24.17731226153105	18.06378262976931
Ag	18.10791220992090	22.18213116675217	18.11610901576388
Ag	18.06180971795040	24.15864064700977	16.03681617789691
Ag	20.11910352539237	22.14525002306183	16.05404486586046
Ag	13.98944680229068	3.89719894237411	20.06381476053763
Ag	9.92873526714855	7.90355570284436	20.09617617841984
Ag	11.94958019568961	5.88710196529140	20.09388675757449
Ag	11.95008889771688	7.90470999560551	22.11445354605696
Ag	13.98469314838522	5.89584508946832	22.10390502486655
Ag	16.04904768223919	5.86454651742031	20.11331270406152
Ag	16.04694288297977	7.88573932135581	22.13637021965528
Ag	18.11817250291859	7.85746490430843	20.14340725710936
Ag	5.88709082348847	11.94935736192790	20.09274245509548
Ag	7.90538217564152	9.92851639840119	20.09446544253039
Ag	7.90786797656487	11.94993005848326	22.11279992872476
Ag	9.92316020351518	9.92191416256576	22.11985848286312
Ag	14.00140903073242	11.96260610127331	20.10930672007605
Ag	16.05589164973049	9.93516105268905	20.09534601392495
Ag	16.05845284993931	11.97427584585772	22.13062955144109
Ag	18.11606125779733	9.89109871199223	22.17930069514344
Ag	20.09995204715923	9.92197702436007	20.10563837600754
Ag	20.09332920773546	11.95007394158886	22.12601017983625
Ag	22.18198628292083	11.92138861774281	20.15590877572060
Ag	3.89565158775145	13.98771830257827	20.06331007243863

Ag	5.89911489129961	13.98342858292250	22.10136385192945
Ag	5.86644824573298	16.04679108879400	20.11187361762494
Ag	7.88758502338657	16.04681327350903	22.13489453477129
Ag	9.93617488359788	16.05569072336558	20.09433575039886
Ag	11.96408307873154	14.00087154506899	20.10874191967721
Ag	11.97664384687898	16.05987459605656	22.12979966662069
Ag	14.02190370110201	14.02104538840112	22.12954591719909
Ag	18.06306220566158	16.03087638619798	20.12952071933147
Ag	20.10082519319557	13.99908911422323	20.10158239108768
Ag	20.15406352706087	16.07724107877165	22.18363290087298
Ag	22.15887400337438	13.99916010504986	22.16115091284058
Ag	22.12515748944274	16.04839248511615	20.09599903572795
Ag	24.09673764276251	13.99804286051464	20.05815631427059
Ag	7.85811000706684	18.11926190733719	20.14416835378019
Ag	9.89204864630796	18.11709116027682	22.17912627726168
Ag	9.92201208414485	20.10069655814490	20.10691180318179
Ag	11.95161681089773	20.09553310363096	22.12647732956964
Ag	14.00017855894879	20.10236947441766	20.10085895840034
Ag	16.02982673007585	18.06416662832418	20.13072026194343
Ag	16.07694250340545	20.15568874288176	22.18451928253740
Ag	18.11311150619909	18.11451320715016	22.18307949994988
Ag	18.07553807297025	20.10435397291233	20.09752434084678
Ag	20.10290676179311	18.07594413317705	20.09859951464428
Ag	11.92168634927781	22.18438805440255	20.15728048331724
Ag	13.99977917057260	22.16193425638371	22.16284412217798
Ag	13.99895551311605	24.09907453268774	20.06287032965225
Ag	16.04780168871214	22.12519705717855	20.09527124778101
Ag	13.98844761750947	7.92738520155357	24.10958076929697
Ag	9.94642031067235	11.96494285197772	24.13916482403821
Ag	11.96663101044232	9.94298115812687	24.13864409378434
Ag	13.98653977220206	11.96318481948388	24.15737611010213
Ag	16.02320137245840	9.93570829257902	24.14706501410949
Ag	18.05900522087769	11.96917072042797	24.17281484059318
Ag	7.93424972851934	13.98801010159181	24.10618112323563
Ag	9.93936914880049	16.02365511053410	24.14526438840460
Ag	11.96601093604058	13.98581849471731	24.15771909360760
Ag	14.01178775798569	16.03316227396581	24.20893375397233
Ag	16.03246162394404	14.01101815698079	24.21165276926864
Ag	18.04732854962482	16.02366225221238	24.12648944232162
Ag	20.05271319883989	13.99988047957560	24.09870636319329
Ag	11.97209250674465	18.06267714116722	24.17246988398857
Ag	14.00042798123836	20.06086046164828	24.10096637223153
Ag	16.02219396489613	18.05040945488578	24.12719161838870

$Pd_{110}Ag_{295}$LEH-1323.547

Pd	11.94637215581843	11.92655140733219	5.85684161116230
Pd	13.99577328609807	9.89479023589967	5.84773144671881
Pd	16.04690629035419	11.92706367446256	5.85410383979420
Pd	9.90275571112334	13.99175420912828	5.84201126706256
Pd	11.94470797901919	16.05901538393190	5.84439044604126
Pd	13.99211396998741	13.99405229185032	5.87743380128022
Pd	16.04961198721880	16.06321625728611	5.84524916428190
Pd	18.10254591758267	13.99468970154981	5.83636415518501
Pd	14.00043933146570	18.11752222699608	5.82822320369158
Pd	11.94495781967501	7.87842439710777	9.90163707565420
Pd	13.98919131612735	5.85670315843682	9.89334014289189
Pd	9.92458401785558	9.90681235849521	9.90330757483565
Pd	11.92914564134016	9.91605003385880	7.89856170582971
Pd	11.95966563265190	11.94878017418651	9.92314621197701
Pd	16.06722301886171	9.91260288709816	7.85788414104198
Pd	18.09695325990448	9.90999012306076	9.91004081334483
Pd	5.83890812788822	13.99476238120602	9.90519974776026

Pd	7.87868888283567	13.98997264580547	7.88391060677534
Pd	9.91935783124934	13.99921573367691	9.90797323901211
Pd	11.94902424998114	16.06157661012558	9.90799981372941
Pd	18.09052166166723	13.99716188696423	9.90702393784872
Pd	20.12879926460690	13.98760446798985	7.86627743719309
Pd	22.15583283795856	13.99448977534456	9.89810916717035
Pd	9.89320781459019	18.11089733376549	9.88848083819965
Pd	14.00173750958726	18.09331933141177	9.90214605384531
Pd	14.00198084641362	20.13323337820126	7.86540553155997
Pd	18.10514576959701	18.10893271175429	9.89962927162587
Pd	14.00545711015406	22.16105200773148	9.89648795533931
Pd	9.89858713159355	5.84893883387999	13.99429166054247
Pd	11.94438630821246	5.86355560421637	11.93120318007402
Pd	13.99769229785512	5.88232653933841	13.99968431590767
Pd	16.06016416575382	5.85586756271159	11.93843665108583
Pd	18.09587744624454	5.85463712697027	13.99787475752371
Pd	20.12951177978713	7.90861049346895	13.99742558705069
Pd	5.84978403311402	9.89654311097836	14.00283109811680
Pd	5.85552976850408	11.91999290593950	11.94564846772227
Pd	7.87340139221669	9.90600934553491	11.93086590754440
Pd	9.89566871565444	9.91220042939703	14.00020722109089
Pd	14.01123792032093	9.90424324714174	13.98312897917947
Pd	14.01537959784180	11.95470385160890	11.96836447270814
Pd	16.02405300204874	9.92538175982787	11.97544257840909
Pd	18.09948709884073	9.91503143699176	13.99713983112547
Pd	20.12947882753661	9.91061528280573	11.96259512645685
Pd	22.14870935461349	9.91067432330014	13.99639980098354
Pd	22.14547978707010	11.92540828737626	11.95203747533609
Pd	5.87968812215824	13.99670358733577	14.00718926929282
Pd	5.85270760714415	16.06253352571451	11.94939170758348
Pd	9.90005569269013	13.99936281047997	14.00781465115114
Pd	14.00908745269938	14.00155736216331	13.98733091479450
Pd	16.04646803884803	13.98825572380618	11.96965675693403
Pd	18.08574964916792	14.00175441424077	14.01248910835364
Pd	22.12719089356782	13.99425426238460	14.01291307221662
Pd	22.15367068901875	16.05511020836791	11.94555790176234
Pd	5.84575513279153	18.11016381710098	14.00034907176439
Pd	7.89294602211603	20.13830542543778	14.01075720348981
Pd	9.90362392363155	18.10605739362389	14.01556625896431
Pd	14.00424167653171	18.09255994993467	14.00763655258934
Pd	16.04372350807191	18.09520052640019	11.96216236563540
Pd	18.08972330660602	18.09103360371468	14.00425319281951
Pd	20.12556233812850	20.12118493968286	14.01040992415550
Pd	22.16185631562967	18.09667536361157	14.00465886422715
Pd	9.89473994803972	22.15279596444472	14.00897667557089
Pd	11.94560534602519	22.15175076674183	11.94482005454472
Pd	13.99399814868031	22.12623555395604	14.00006362627880
Pd	16.05764843251163	22.16026321995396	11.94246289839883
Pd	18.10498529971423	22.15657452531556	14.00772122065193
Pd	11.94631995922357	5.85876067955427	16.08337522345294
Pd	11.92428717035355	7.87186296473087	18.10080192562166
Pd	13.98837662913606	5.84357294156453	18.09913990049720
Pd	16.04894786652922	5.85677780795852	16.06840279934757
Pd	16.05951989416887	7.85053846414803	18.09534934796959
Pd	5.86738305064678	11.92644539546495	16.05111165487653
Pd	7.87315965241226	9.90542105243498	16.07337061679710
Pd	9.89823469943094	9.90191387211019	18.10524828637553
Pd	13.99961428239406	9.88623850715379	18.09136569087364
Pd	16.02866796774162	11.95919527668700	18.08916532728265
Pd	18.10037465188010	9.89712650076177	18.09763107787406
Pd	20.14780714022142	11.93615359162636	18.09227315414933
Pd	22.15054380910026	11.94680209778846	16.07491194504181

Pd	5.84906816269734	13.99847420575756	18.09808064572815
Pd	5.84601028182815	16.06208714045025	16.06004523324530
Pd	9.90305727709619	13.99891908270752	18.10107939263408
Pd	9.90548142265235	16.03119303245951	16.04591611534850
Pd	14.00690820133924	14.01249834249988	18.09332243867341
Pd	14.00849967014310	16.05314284468823	16.06381894132238
Pd	16.01797122493333	16.04743787847121	18.06901059806922
Pd	18.09776667784726	14.00486932236974	18.07726788401434
Pd	20.14848920456075	16.07191212919359	18.09049612097513
Pd	22.16170135493608	14.00181402538405	18.09955433592184
Pd	22.15466344900370	16.05288359470254	16.07545690724143
Pd	9.89951157931036	18.10032845490529	18.08741878590198
Pd	9.89880735539536	20.12929825140414	16.04430700603760
Pd	13.99252250608815	18.10353935846684	18.09239231933920
Pd	16.03609489555275	20.12781919077707	18.09915360893878
Pd	18.09990362998254	18.10790792528945	18.09151672542584
Pd	11.92173625944971	22.14327418043369	16.05722210800124
Pd	13.99887016964234	22.14524380486040	18.09385250454990
Pd	16.05027961925392	22.13710990745354	16.07175460276817
Pd	9.89820937508016	11.95246912217764	20.12299463778750
Pd	11.92921004680415	11.95893760896972	22.13875836996503
Pd	13.99902540434783	9.89244115727598	22.15061752818089
Pd	16.06155936575632	11.94012054914172	22.13696843433391
Pd	7.90414981877555	13.99499614785229	20.13471650039186
Pd	9.90258347442829	13.99200863538219	22.15903205826465
Pd	14.01328008005902	13.99459164873824	22.12399250046326
Pd	16.04616263926527	16.05599704664523	22.14003120659730
Pd	18.09856355492308	14.00162492150333	22.14142723093774
Pd	11.94852002890538	18.10966508776613	20.09658317698244
Pd	13.99585277316796	18.09983864662424	22.14384845814938
Pd	13.99296556600223	20.10761871025790	20.09361396048673
Ag	11.93708000005253	7.87163111824311	5.84070823528718
Ag	13.99455376262442	5.82362861288842	5.82301639887206
Ag	13.99337067809601	7.89126555379218	3.87970758765989
Ag	16.05661023889282	7.85584588363914	5.83589024966501
Ag	7.86717402965542	11.93325726805321	5.83688688928394
Ag	9.89863716993277	9.88906883970970	5.83158711087141
Ag	9.94996659588264	11.97043634529046	3.86813739636394
Ag	11.98430422103680	9.93071972308228	3.84631026526330
Ag	13.99587707877605	11.96682212397834	3.89821782534174
Ag	16.00973785535027	9.92864035155356	3.85292814334961
Ag	18.10323662762475	9.88542294006538	5.81421478402699
Ag	18.04302484479651	11.97103158838773	3.86256338671032
Ag	20.14413202886708	11.93383308828364	5.82456512516444
Ag	5.87974838626496	13.99669908467416	5.86495713722502
Ag	7.93429274783223	13.99569454593348	3.85555977049153
Ag	7.86550465666043	16.05437266408263	5.84055975933896
Ag	9.94384621142766	16.02435020223299	3.86616652708596
Ag	11.97071043585334	13.99382482540543	3.89133570481316
Ag	13.99638446163351	16.03118039165846	3.89620869872369
Ag	16.02068362667425	13.99532167493479	3.89771427725457
Ag	18.04932588136793	16.02576419729054	3.86228323955199
Ag	20.06965554941465	13.99385040642229	3.85869992073088
Ag	20.14540611482279	16.05428093591446	5.82757790438882
Ag	22.14084393081951	13.99504034265155	5.86202284739407
Ag	9.88226808056696	18.11938210609516	5.80687904116532
Ag	11.97352253293660	18.06459852343577	3.85263949727951
Ag	11.94239641835804	20.14661913141989	5.82777064462636
Ag	13.99724772230836	20.09160534146363	3.84490415037725
Ag	16.02176793282869	18.06338585233855	3.84664269520625
Ag	16.06347572202551	20.15373534841479	5.81293354776580
Ag	18.11577081324250	18.12118099204815	5.80034373774497

Ag	13.99979794165559	22.14351007779664	5.85128201371328
Ag	11.98330785863170	3.85817228100273	9.93930243493610
Ag	13.99356525046105	3.87338105185018	7.89888899803234
Ag	16.00877265998945	3.87311802106299	9.93685815372765
Ag	7.86057553299727	7.87789757443221	9.89966101209149
Ag	9.89614768573065	5.83430351762845	9.88978416657043
Ag	9.91990696565421	7.88877535065727	7.89029472803811
Ag	11.94069319826856	5.83554065753370	7.85988127070064
Ag	13.98961665543276	7.91212171185268	7.88830083517904
Ag	16.06069875008343	5.82500125263573	7.84756198163047
Ag	16.05796537317597	7.90745630290007	9.92424141181045
Ag	18.12039807897287	5.82027753729703	9.88073966798137
Ag	18.09755324029148	7.87525311134645	7.86516512797524
Ag	20.12982835765234	7.87933986510560	9.89785801384859
Ag	3.86237125037302	11.97022212132716	9.94863720482525
Ag	5.81759125988804	9.88495412294696	9.89176455640269
Ag	5.83296880168926	11.93628783223544	7.85848724155225
Ag	7.84896820291581	9.87714125395045	7.84505885885214
Ag	7.87657270036541	11.95747444454438	9.91413650354500
Ag	9.93093654738870	11.95477746360484	7.90027388956611
Ag	13.99199307264064	9.93574852736558	9.91543231226085
Ag	13.98938760086815	11.91964348085186	7.84617378314008
Ag	16.05380197909756	11.94394182727511	9.93883960867692
Ag	18.08435287589655	11.95798248976362	7.87760641210427
Ag	20.15191353804163	9.87862203126159	7.84600133232216
Ag	20.12143921409887	11.95749444882505	9.91978517857181
Ag	22.17187171074393	9.88863672976941	9.89705320647933
Ag	22.17112438224919	11.93776505223899	7.85611518899896
Ag	24.13675964701530	11.96938666449911	9.94491757651411
Ag	3.86420970416177	13.99201465609830	7.93630936097161
Ag	3.86467500143093	16.02849135575736	9.95065013857631
Ag	5.83359270223148	16.05399288088636	7.85925899295375
Ag	7.86966984284571	16.02680492933258	9.90576892194542
Ag	9.91499602202978	16.03393486365255	7.88778631050431
Ag	11.95198951385899	13.99864741030826	7.87364360859117
Ag	13.99474491717073	13.98036732739169	9.92892300281087
Ag	13.98828696792115	16.05443658558820	7.85963280223436
Ag	16.04496523712037	13.99672192349528	7.84281628187997
Ag	16.05668258928286	16.05188245271793	9.93478486289492
Ag	18.09656553127459	16.02827456651585	7.87041715072257
Ag	20.11966544976263	16.02449163201085	9.90705261320376
Ag	22.16520137355185	16.05136252294205	7.85755887064036
Ag	24.14062872120389	13.99435425783885	7.93168385559064
Ag	24.14025004328110	16.02442341073247	9.94471100091711
Ag	5.80615258010409	18.11784387820531	9.88671789930259
Ag	7.84818943050878	18.12335997176069	7.84520370154001
Ag	7.84784538960606	20.15529708147523	9.87828780704504
Ag	9.87825075183496	20.15842629092419	7.84237547164220
Ag	11.96458862304935	18.09231168054431	7.88184980906099
Ag	11.97501434912792	20.13535433587184	9.89956500423549
Ag	16.03032173269919	18.09822167579343	7.86659795776875
Ag	16.03475786020011	20.11110384093566	9.91622966740263
Ag	18.12070437063042	20.15616510894802	7.84390212438337
Ag	20.14795507040326	18.12087359424898	7.84926669348280
Ag	20.14809370557061	20.15572714400151	9.88087406455964
Ag	22.18696901557542	18.11317492115058	9.88675175324303
Ag	9.88128916769792	22.20005490745557	9.88086030468776
Ag	11.94496069310634	22.18190451599810	7.85094959053899
Ag	11.97960839745680	24.14045598468286	9.94249074912371
Ag	14.00108810699122	24.15195526486073	7.92434569155646
Ag	16.05415650096028	22.16388371919965	7.85883570720417
Ag	16.02460070973671	24.14640829005241	9.93949363647772

Ag	18.11571696876283	22.19334340385178	9.88249794862636
Ag	7.91976961373716	3.87074801273162	14.00393619041292
Ag	9.94778072462495	3.87127598649296	11.97033192080101
Ag	11.97490737448461	3.90962714708383	13.99998859158835
Ag	13.99867232715249	3.90512614772299	11.97291662881522
Ag	16.02528732766999	3.90181022752602	14.00379975397146
Ag	18.04940469510424	3.87163940505211	11.97487736716364
Ag	20.06500569370324	3.86303131898492	13.99920649405524
Ag	3.87359214269220	7.89814373918959	13.99916530628893
Ag	5.83563714121235	5.83954858199037	14.00169911140195
Ag	5.83215923194583	7.85851990988404	11.93645066011407
Ag	7.84438649253219	5.81246842501972	11.92596503403421
Ag	7.88170388101268	7.91830173591760	13.99994310449994
Ag	9.92136030853811	7.89520211479489	11.94548702878587
Ag	11.95664096786858	7.85764548453432	14.00361703268409
Ag	13.99715756430363	7.85510311282575	11.94428999479759
Ag	16.03834658789103	7.87359210470735	13.99303604074760
Ag	18.08846837567263	7.92194424097756	11.97306997055050
Ag	20.12505519566624	5.84943355242150	11.94531773048927
Ag	22.12161330033229	5.87658504872865	13.99421424849698
Ag	22.13839126691968	7.88586664145581	11.95139850158058
Ag	24.13175573767199	7.92992391771770	13.99830008619622
Ag	3.85478612214778	9.93341716625670	11.99113339109846
Ag	3.90208731132315	11.97163082796514	14.00481463429768
Ag	7.85612340988052	11.91427016091245	13.99690948217656
Ag	9.90951221281036	11.94400246956871	11.94996585636890
Ag	11.96869306403955	9.92641068845432	11.95721208297220
Ag	11.97994161615449	11.96193508892537	13.99809512526517
Ag	16.02932324382129	11.96358317484329	13.98552940924107
Ag	18.08215057717168	11.94745166842901	11.97285999003817
Ag	20.13941461560002	11.94958748584858	14.00264600793089
Ag	24.14871080424267	9.94034389692876	11.97690764878323
Ag	24.10741614376961	11.96888764218906	14.00745614509584
Ag	3.90624261288898	13.99424899383479	11.97848361765580
Ag	3.90191119808713	16.03215672171952	14.00042116805742
Ag	7.85492298156292	13.99690252422585	11.96196686011055
Ag	7.87215360213469	16.04710045768389	14.01204523377014
Ag	9.92629761007230	16.04486499011084	11.94300449235768
Ag	11.95377512717993	13.99091373808779	11.95220633360813
Ag	11.96121937139215	16.03654134688868	14.02560985666062
Ag	14.00864106500904	16.06144876343443	11.95421230083027
Ag	16.02306276208813	16.04357092453623	14.00353777879681
Ag	18.06235605542871	16.03898583559957	11.97111178805735
Ag	20.14624629219412	13.99974167795181	11.96492382469188
Ag	20.14210839230034	16.03538085946957	14.01523824973714
Ag	24.09175594362305	13.99109435541046	11.97240038900815
Ag	24.10617795656861	16.02562090782198	14.00512325319983
Ag	3.85462980738629	18.06180489300638	11.98018105386248
Ag	3.85205167677952	20.07839403711187	14.00971753740824
Ag	5.83605940662477	20.14630653664253	11.95147998371876
Ag	7.88180877828649	18.09866860635953	11.97260561138437
Ag	9.90579433675538	20.13413079476511	11.96476916991284
Ag	11.95158998120437	18.08123247623137	11.95280836976844
Ag	11.94248838881890	20.14166020889989	14.01544857856474
Ag	14.00786024080455	20.12899491330831	11.95982612725000
Ag	16.04206427907521	20.13210834601499	13.99411929533142
Ag	18.08340351140079	20.11121232505474	11.96964618435650
Ag	20.12783309665227	18.08911210244789	11.97817724016965
Ag	22.16563075138667	20.14250119611270	11.94542477759877
Ag	24.13713851488953	18.05537760215189	11.97344727125734
Ag	24.14006843770868	20.06817969679638	14.01008451612521
Ag	5.86599751242176	22.13565418091871	14.00895391656319

Ag	7.85100855801876	22.17390618711692	11.94536014119268
Ag	7.91765260761584	24.14087411893003	14.01088576810048
Ag	9.93738573063282	24.13531193667862	11.98274113854732
Ag	11.96464756466852	24.10437380367938	14.00406503943148
Ag	14.00023892662419	24.10922414072781	11.97016016193531
Ag	16.03032505136957	24.09068371753682	14.01324524524107
Ag	18.05735311843315	24.14346525768277	11.97847336834594
Ag	20.13466498601573	22.16379832831684	11.94596668951151
Ag	20.06727268859365	24.14966187791752	13.99926728789462
Ag	22.12618881575416	22.13533526569125	14.00383583397268
Ag	9.94806425888269	3.87890442468986	16.02267096968696
Ag	11.98295942801174	3.84157409842557	18.06687383235852
Ag	13.99535370869783	3.89891886861390	16.02391528756884
Ag	16.00888048980190	3.85481053925272	18.05827120675595
Ag	18.05274553470039	3.87481300259394	16.02427863410924
Ag	5.83105821816611	7.86117512164075	16.06481956292665
Ag	7.83640970616667	5.80649069989160	16.07356261734106
Ag	7.85471949067291	7.86586906474840	18.10516673080916
Ag	9.88777052306354	5.81722171523214	18.11792037066516
Ag	9.90783056864493	7.88566488330304	16.05332705551358
Ag	14.00029064218139	7.85623495740556	16.08250584772783
Ag	18.10352981919420	5.82048247598500	18.10810043105528
Ag	18.08435254705233	7.88576367302568	16.03818854710166
Ag	20.13660577935453	5.84661825302013	16.04773536629979
Ag	20.15260645820182	7.85126635977690	18.12012642322453
Ag	22.16569532479104	7.86536522437918	16.05510301249158
Ag	3.85534709078217	9.93614269464050	16.01645232235119
Ag	3.87494929457312	11.97435583769199	18.04780513667225
Ag	5.82732095655494	9.89084567911933	18.10541377076614
Ag	7.89179644753750	11.96457607771329	18.09986542885825
Ag	9.90579642550575	11.94103226318354	16.03241851677398
Ag	11.96716061208508	9.89625023150117	16.05686567578442
Ag	11.93863694375955	11.96160915031201	18.09549696845881
Ag	14.00877663614880	11.95626405482509	16.06510526055664
Ag	16.03837904375808	9.91211610679381	16.07616960970804
Ag	18.07916426591065	11.96388121041998	16.07628246726562
Ag	20.12193225582051	9.92143070957988	16.04795691714721
Ag	22.18937340395401	9.89239404197674	18.11388771597392
Ag	24.13446894114028	9.94240760649090	16.02322349314474
Ag	24.15358433860926	11.98798096325339	18.06805511457397
Ag	3.90251163490729	13.99422731325381	16.02927790812762
Ag	3.86088618487384	16.02530954847921	18.05988753763815
Ag	7.86787977394510	14.00622875651528	16.04203688753283
Ag	7.89462720292133	16.03737642401390	18.07747943805326
Ag	11.96331481213573	14.02249327473945	16.04945742549135
Ag	11.95163367828566	16.05963941263698	18.07340943679592
Ag	16.02319568863450	14.01488785846648	16.06933951239621
Ag	18.08778047019021	16.04176783439781	16.07868914063262
Ag	20.14668201697323	13.99844097620523	16.08936951354945
Ag	24.11238111302850	13.99968600102016	16.03334743439028
Ag	24.15722303984702	16.01286447965899	18.07184314173502
Ag	3.86022414927162	18.06021487817400	16.02650275511334
Ag	5.81113710211669	18.11812395822916	18.12054396764009
Ag	5.83659898816804	20.13500014272677	16.05648751955032
Ag	7.90359883770763	18.09349522065616	16.03691610100381
Ag	7.87093517970433	20.13673947217680	18.10662083523053
Ag	11.94595046580747	18.07532828144913	16.03659620948938
Ag	11.94462668638733	20.11263560571313	18.07785913455800
Ag	14.00614145052849	20.14567298361070	16.05784454694097
Ag	16.02065211155463	18.07722394522438	16.07819380502238
Ag	18.07708987970053	20.11812983543066	16.04388960240353
Ag	20.12573769487708	18.08651581089521	16.04293904575097

Ag	20.16112277357947	20.15297939490391	18.12185674306491
Ag	22.19112371418988	18.11117600546435	18.11645867721584
Ag	22.17426934137369	20.14230716782432	16.06280998148919
Ag	24.14163087528251	18.05135157001717	16.02820444181793
Ag	7.87876596311249	22.14781557132834	16.05586811525315
Ag	9.88404411539510	22.17494624956236	18.10764379209988
Ag	9.93914912542022	24.15136149853075	16.02410767847335
Ag	11.97057975919565	24.13110987651394	18.06441013788765
Ag	13.98946075312516	24.09191139045091	16.03244960794055
Ag	16.02090465764467	24.14202333596321	18.05346689659620
Ag	18.09930198646848	22.16955217320585	18.10724864095398
Ag	18.05579224400342	24.13684083314406	16.03165635818604
Ag	20.14123807615341	22.17138046410153	16.06279987907600
Ag	13.99699264860929	3.86833218098325	20.09986494761198
Ag	9.89616158194921	7.84889505579460	20.13600673674253
Ag	11.93176234409840	5.82059958257366	20.14194454115402
Ag	11.92623515548943	7.83217568489028	22.19489637860878
Ag	13.99807964825408	5.83053795611678	22.16030416281913
Ag	13.99095921924184	7.86857208227105	20.08002027277251
Ag	16.06148526465040	5.82425178119359	20.14079292551482
Ag	16.06475695835122	7.84570046348282	22.17747296480950
Ag	18.10068776745254	7.85658028381831	20.12864231053068
Ag	5.83263119730652	11.94300209035815	20.14061133803794
Ag	7.87447727418342	9.89259681665905	20.14570661124333
Ag	7.88170154445095	11.94985053431910	22.14284846497233
Ag	9.88931820680448	9.89324811696316	22.17680291397408
Ag	11.94628837400166	9.91505529147341	20.11852595009427
Ag	14.01504190333984	11.94774548328748	20.12375728686749
Ag	16.04516172606384	9.90772585059113	20.08588613572644
Ag	18.11608127930186	9.88383886026174	22.16679220551624
Ag	18.08715023354764	11.95890330099838	20.07743881657663
Ag	20.13919343522586	9.90057060150291	20.12770079421461
Ag	20.15536183573399	11.93235479256291	22.17238515381721
Ag	22.17486741140399	11.93675390536716	20.13867773550273
Ag	3.86218075646363	14.00166520406828	20.06788107697951
Ag	5.87686542639294	13.99436038063232	22.12971474547181
Ag	5.85752061083192	16.04606356204823	20.13220576848339
Ag	7.87110719933591	16.05750996353382	22.16739149951340
Ag	9.91254550353663	16.05386297470496	20.11033697266870
Ag	11.94400424732602	13.97822164809544	20.12451108163530
Ag	11.96963974208458	16.05756550519685	22.12814003408316
Ag	14.00638409646189	16.05877670230611	20.10749092265450
Ag	16.04688149473681	14.00512420802306	20.10204272397790
Ag	18.07692903470586	16.04888691080675	20.07424359105123
Ag	20.12328232468328	14.00429866651485	20.06643151431774
Ag	20.15232638770627	16.07056954538007	22.16894689047339
Ag	22.16402905591791	13.99826673234282	22.15983988023265
Ag	22.17754330254566	16.06470252560246	20.14155995860833
Ag	24.13242190445122	14.00271880711052	20.10262193094152
Ag	7.84279401936324	18.12433564843190	20.14966335162740
Ag	9.88948768421756	18.11850908003366	22.19350470500538
Ag	9.89191448549989	20.11861608756680	20.13765830359269
Ag	11.96156511419670	20.10066607369280	22.12770225276735
Ag	16.03286281339784	18.08919955860193	20.07589831831877
Ag	16.04909250498508	20.12699365354947	22.13965710143128
Ag	18.11554694185378	18.11165142332718	22.16433705917670
Ag	18.10549373770539	20.13248600666901	20.12021584106908
Ag	20.14215537852115	18.09955281225476	20.12538212793065
Ag	11.94572029629100	22.15925424059288	20.13241494627228
Ag	13.99832430200395	22.11726060515254	22.12084054005577
Ag	13.99781325659987	24.12877854992362	20.06796027658335
Ag	16.04041821213149	22.13130003142466	20.11370896112276

Ag	13.99948687599133	7.90717435786471	24.13337879644826
Ag	9.94245240932100	11.97367176771691	24.14978261189279
Ag	11.98035434959521	9.94267621247205	24.12217558841262
Ag	14.00079500157165	11.96948000375579	24.09228523216965
Ag	16.02548009382799	9.93867621396254	24.12716676669613
Ag	18.06016507213186	11.97687704992794	24.11949320873307
Ag	7.92528967901761	13.99219948234077	24.13873163369809
Ag	9.93688546874501	16.02451609788225	24.16388233362343
Ag	11.97890132438768	13.99187820817315	24.12820816727600
Ag	14.01791921134994	16.02969575586626	24.13869986053536
Ag	16.03092611988325	13.99676608641196	24.10256631016579
Ag	18.05873617110218	16.02119478913137	24.11523835137242
Ag	20.09070964128888	13.99855410465053	24.11838828788409
Ag	11.97469215678801	18.06918351591799	24.17345627434794
Ag	14.00096408160365	20.07263304044284	24.13053213431035
Ag	16.02542300449901	18.06208201397630	24.12152683666359

$Pd_{40}Au_{161}L1_0$-680.860

Pd	14.55511979613052	12.50006330168741	14.46592208297522
Pd	16.61351089603435	14.57976699225715	10.49405670303931
Pd	18.62256393402465	12.50003014879399	10.51401274108662
Pd	8.38652025440000	10.42024526194436	14.50648971640985
Pd	6.37753884580064	12.50007117872005	10.51407332792771
Pd	12.49999606651401	14.52018662895751	18.41769691349713
Pd	14.52016118887086	12.50006383856271	6.58203509625801
Pd	14.52014917647882	12.49999072182464	18.41800296937732
Pd	12.49999888455318	10.44483092467542	14.46551834270499
Pd	12.50000446441748	6.37733239332863	10.51380114965403
Pd	12.49999849627017	14.55505986376709	14.46644229657579
Pd	10.42024927882354	16.61350175446700	10.49386426871135
Pd	18.62246560047417	12.49998923922231	14.48602693985850
Pd	10.42024743915771	16.61355023190259	14.50620245779642
Pd	10.44490865175655	12.50007072478474	14.46611130342093
Pd	10.47983326999694	12.50004315977418	18.41784775485868
Pd	14.57978259265240	16.61353179841921	10.49375651098169
Pd	6.37753303769277	12.50004600268410	14.48592008586156
Pd	14.57979095506840	8.38672535886317	14.50628292988403
Pd	12.49999984175393	18.62235616519401	10.51426495423037
Pd	14.57979487308861	8.38667182371111	10.49368316941341
Pd	10.42022581028012	8.38667861551962	10.49377688879942
Pd	12.49999544154349	14.52007771119735	6.58223986431145
Pd	12.50002746414286	14.55501460553191	10.53362391409445
Pd	16.61347873961886	14.57974941025514	14.50597929195865
Pd	12.50001167694794	18.62239746933302	14.48574621275528
Pd	12.49998883009906	10.47976374397778	18.41822511456887
Pd	16.61340933419968	10.42021614324873	14.50647912870177
Pd	14.55517113192200	12.50006760625016	10.53405831962950
Pd	10.42021400731483	8.38670523053775	14.50619392377325
Pd	8.38649835041677	14.57975917359443	14.50599632560058
Pd	12.50001493727372	10.47969596332053	6.58187538760287
Pd	10.47986349852821	12.50003147282203	6.58209926207408
Pd	12.49999623345602	10.44479436923738	10.53445910966967
Pd	12.49998031186474	6.37746294854633	14.48617235177193
Pd	8.38650498162491	10.42022222444213	10.49348555705413
Pd	8.38649541849202	14.57975840181473	10.49409219044660
Pd	16.61344182574278	10.42021731750117	10.49345293986003
Pd	14.57979457737735	16.61357417411524	14.50631579053384
Pd	10.44490378013016	12.50003460013619	10.53394665320932
Au	14.50079764721880	14.50053128550850	4.54671500207933
Au	12.49992266317000	6.43647463562123	18.49010053638317
Au	8.36074749923154	10.35881273254260	18.55596242746975
Au	12.49998421070690	4.47236148415436	16.47452017446433

Au	20.52785378197107	12.50000148197444	16.47458844825617
Au	10.49926109714305	14.50067714641123	20.45326532664819
Au	10.36143772424192	18.65567202351427	16.56806837141552
Au	20.64047106193761	14.52968830660648	14.50178470099769
Au	20.64038998468192	10.47014569037578	14.50157720465684
Au	10.49924364371224	14.50054801408717	4.54670073729902
Au	10.35882129510885	8.36090778662707	18.55609913895089
Au	4.35943942078638	14.52982080555496	14.50176254496836
Au	14.63834503397930	6.34445808085334	16.56807121904841
Au	6.34441363671762	10.36141693689834	16.56809485500160
Au	12.49997523963874	6.43629537194166	6.50997286805315
Au	14.64120422576056	16.63923309058803	18.55608404062267
Au	14.52977573806636	4.35961247551275	10.49840297755317
Au	6.29903306406847	8.31460913258615	14.62092733538874
Au	14.63834813738955	6.34434202190816	8.43192058354529
Au	10.47020771564672	20.64052534015936	10.49828552998455
Au	8.31449751763117	6.29904405659411	10.37924528955670
Au	10.35878208516972	16.63914440081020	6.44392021042390
Au	18.70091235519975	8.31460879859486	14.62083804384726
Au	14.52986983508439	20.64056902205853	14.50164826575768
Au	6.34427359415310	14.63845886434732	8.43197132367373
Au	6.36944710336847	6.36948748800309	12.49994335163528
Au	8.31453030036464	18.70094849780376	14.62098249836020
Au	18.63047028413716	6.36945731130294	12.49993581255278
Au	6.43629926064812	12.49996916517931	6.50985434278734
Au	18.70091342271835	16.68544291228539	14.62096715535545
Au	16.65580423152709	8.34433366203494	16.56319505057772
Au	6.34435319754318	10.36136632291571	8.43185656882064
Au	6.29910000704693	16.68548111854135	10.37907332346892
Au	16.65587628094613	16.65601405756111	16.56318007718404
Au	14.63865176928177	18.65565152262325	16.56816063419956
Au	16.68548148423872	6.29895383019381	10.37913799650193
Au	12.50003169901453	4.23425749313563	12.50003517146111
Au	10.47016831359606	20.64062566834698	14.50168669599269
Au	14.52975384460216	4.35973152254155	14.50160910964888
Au	12.50005415049349	12.49986780136130	4.51608590475819
Au	8.31460032715250	18.70082560122358	10.37900766486582
Au	10.36166307276501	6.34435258018079	8.43202623887748
Au	12.50003087285939	20.76587524016331	12.49994142926043
Au	18.70101631241605	8.31453816518664	10.37899963463625
Au	10.35885657540021	8.36078888471307	6.44396523042955
Au	16.68538197193972	6.29910683743718	14.62073027939875
Au	14.50056542754519	10.49932458312761	4.54683204912964
Au	14.64121956460848	8.36075725864285	6.44395081883733
Au	18.63060119983398	18.63050340280126	12.50001751404130
Au	6.29904386468980	16.68551443843414	14.62097099889833
Au	16.68550204005771	18.70095679614690	14.62103345302127
Au	18.65575894025405	14.63839843246083	8.43197339446695
Au	10.49935689061035	10.49935425036062	20.45332927409736
Au	6.29895596176983	8.31459296999306	10.37905633842834
Au	8.34409845372980	8.34433721675570	16.56319806061103
Au	18.70094724119053	16.68546994581331	10.37908925512063
Au	18.56359529029999	12.49993410320454	18.49016830088858
Au	14.50065750412855	14.50061302971658	20.45333240892000
Au	8.36079053208461	10.35871191301350	6.44402606223453
Au	14.50060866495873	10.49928838000984	20.45339841979981
Au	4.35949189774901	14.52982893275315	10.49824328344557
Au	16.68543238946538	18.70087080005933	10.37898805624046
Au	4.35957359761660	10.47021108481248	10.49842931275029
Au	12.50004342688348	18.56372623292667	18.49020658476622
Au	16.63922916882271	10.35869392320813	6.44406405400638
Au	8.36077342367862	14.64117836301045	18.55609851137537

Au	10.35880816547725	16.63926701650895	18.55602847985460
Au	20.64047664615641	10.47018949122910	10.49841016819020
Au	4.23419142152575	12.50000587495860	12.49995721094437
Au	14.63858363728475	18.65558531780155	8.43189980261942
Au	16.63918173088061	14.64106598705512	6.44388203450921
Au	6.36939757476831	18.63056394490019	12.50003991819568
Au	6.43628135648447	12.49999901971334	18.49011739809326
Au	18.65549887810195	10.36147392145853	16.56808402934227
Au	8.31454986350449	6.29912280978074	14.62073172992174
Au	4.35949794152314	10.47022072944584	14.50153561336380
Au	8.34405304920855	8.34426867574073	8.43681428990631
Au	10.47022823477986	4.35958777360508	10.49845596465852
Au	20.76579789053444	12.49990308185130	12.50005251443256
Au	16.65584882278398	16.65597376313080	8.43684602353392
Au	20.64054878874122	14.52975690807028	10.49828124562559
Au	16.65585076577264	8.34426285561329	8.43672209315445
Au	18.56375170427815	12.49996383640224	6.50985578928270
Au	18.65563668954879	10.36141327428495	8.43182896166102
Au	10.47027415143397	4.35972514067146	14.50157757419693
Au	18.65570597292355	14.63837708792834	16.56811939562419
Au	6.34425282843468	14.63850089791660	16.56809942601344
Au	8.34414928563067	16.65590082153183	8.43689078976801
Au	16.63917571025637	10.35874746604233	18.55604944043526
Au	14.52981352963787	20.64050333240883	10.49833892779329
Au	8.36082980956501	14.64100193804504	6.44390324836468
Au	14.64116919201809	16.63912931377662	6.44392815439658
Au	14.64105369572802	8.36091136930906	18.55609083329781
Au	10.36162705738968	6.34441076361679	16.56802856485728
Au	12.49996654500394	12.49996646010259	20.48391576745930
Au	16.63915032139501	14.64112029292152	18.55619760602672
Au	10.49947466618586	10.49923763859652	4.54686701360752
Au	10.36144256187056	18.65557406078211	8.43194366684301
Au	8.34406113964416	16.65605481132884	16.56308958305547
Au	12.49994766486178	18.56354852944613	6.50971175274539
Au	16.47802325126027	12.50007823934173	20.38627236716092
Au	12.50004732991954	8.52195219517840	4.61391737567921
Au	16.47811221627592	12.49996393383053	4.61388750934979
Au	6.39676368893546	10.47064924202570	12.49997186001158
Au	12.49994272430666	16.57992977212057	12.50002384053653
Au	12.49988508144868	8.52208429602538	20.38627790482720
Au	20.58191269025728	16.57473870977357	12.50007278002692
Au	12.50000588563855	20.52796291854911	8.52530661852039
Au	18.60283716957855	14.52926008669062	12.49999734353802
Au	12.49992725548667	16.47828436482792	20.38628699131531
Au	12.49998343112477	4.47223120367846	8.52552588112998
Au	16.59327765925392	16.59307569033918	12.50001420299560
Au	10.44733206782537	14.55285277757763	16.43683553485268
Au	8.42707135130506	12.49985098113907	16.42201222011285
Au	16.57477312030386	20.58200679011115	12.49995882309226
Au	20.52790566732784	12.49998680605453	8.52543869987292
Au	4.47201230731406	12.50003519266306	16.47454304847336
Au	10.44067680644884	10.44000495955659	12.49997379762529
Au	8.42519969868658	20.58205481844941	12.49992484485866
Au	12.49993705429140	12.50043279446971	16.41568507642112
Au	10.47040026196312	6.39690029199760	12.49995720994287
Au	12.50009775993290	12.49959508728145	12.49997339832157
Au	14.56030473693782	14.55962257581250	12.50000527320825
Au	12.49993231497092	8.42144110911036	12.49993698626657
Au	4.41800760056498	16.57484531508234	12.49999445578285
Au	14.55936556029913	10.44015805303546	12.49997311057325
Au	14.55323101758147	10.44696552279401	8.56327903681376
Au	14.52963203376397	6.39680615032655	12.49994486656137

Au	12.49993316459275	12.50041335897517	8.58431397484888
Au	10.47085298457569	18.60288784342137	12.50001533771469
Au	12.50004112104098	8.42693947578613	16.42201984015862
Au	12.49997348789755	16.47807668295583	4.61366018011780
Au	12.50000082658925	20.52808789906503	16.47460254693707
Au	16.57926961327356	12.50039077706057	12.49996469852512
Au	8.52182924703654	12.50014185599106	20.38617435937064
Au	16.57304439015559	12.49983932977563	8.57796730111274
Au	12.50006315798821	16.57282861962546	16.42200329073153
Au	16.57294851288047	12.49983096501630	16.42205176284639
Au	10.43978454396358	14.55975412342024	12.50002319649627
Au	8.42709324193466	12.49983255223970	8.57798620004163
Au	10.44679142665780	10.44710081548173	16.43673445799667
Au	10.44737525128052	14.55278379921765	8.56319517775831
Au	6.39722597606435	14.52923474624875	12.50003149580965
Au	4.47203011945901	12.50001414402824	8.52544503258536
Au	12.50001918523532	16.57274571294952	8.57798676026387
Au	14.55268291130424	14.55296284651661	16.43683737030949
Au	16.57466881283738	4.41824073520015	12.49999119698766
Au	14.52920773280091	18.60296292576988	12.49999983490434
Au	14.55320475921388	10.44699028198673	16.43673553551272
Au	18.60329199356199	10.47053071036839	12.49993338557142
Au	16.59328392476140	8.40664480804767	12.49991795048425
Au	8.42526351579482	4.41826538437469	12.50005334395309
Au	10.44682910963425	10.44704153037066	8.56324407600093
Au	8.40683142097300	16.59305854075214	12.50002660254416
Au	8.40675727885148	8.40667693153040	12.49995823292968
Au	14.55265079080010	14.55290264388164	8.56318720107914
Au	20.58182269220851	8.42535164458673	12.49991541189719
Au	12.50006158738709	8.42684844539129	8.57798763227832
Au	8.52190765567799	12.49996138745336	4.61381044791581
Au	8.42049854170979	12.50039122471619	12.49999169591348
Au	4.41803892013221	8.42541375323740	12.50006097992012

$Pd_{40}Au_{161}$LEH-681.346

Pd	6.43565926801491	10.48592763275453	12.49991132656169
Pd	18.56649201045745	14.53065447588663	12.50003890231252
Pd	16.57444971598642	16.57075683807168	12.49995849663055
Pd	8.43044501087517	12.50022115023600	16.56966615878665
Pd	14.53657053112615	14.54018789335190	12.49999218024908
Pd	14.53651983506966	10.45982777612098	12.50011933956038
Pd	14.56939368827450	10.42585526616039	8.45729067910926
Pd	14.51805739587338	6.43712174580101	12.50039754362015
Pd	16.56519130982587	12.49970824268387	8.44879618067680
Pd	16.56494633145634	12.50020258520853	16.55142292696248
Pd	8.43054873460741	12.49991765841896	8.43018268080685
Pd	6.43562810078715	14.51419441000171	12.49968539688337
Pd	14.56928710695403	14.57405697258231	16.54290531660815
Pd	14.51818345503493	18.56285623846763	12.49981019274307
Pd	14.56933353325038	10.42622944747466	16.54302329956248
Pd	18.56650624977447	10.46920736783112	12.50017273140913
Pd	16.57441350019028	8.42915363440177	12.50026030489619
Pd	8.43357347446084	16.55287629065392	12.49963690265717
Pd	8.43362377482745	8.44722795123585	12.50007028003615
Pd	14.56944534607950	14.57375154182138	8.45709679545683
Pd	18.56342943379861	12.49984798326096	10.48427517878071
Pd	6.43531222644600	12.49999099354105	10.46778475494199
Pd	12.50663534109709	14.54135016004779	18.55545269696733
Pd	12.49062596274667	6.44584503006153	10.45521486778550
Pd	10.42938567634383	16.54478882936071	10.42620029534679
Pd	18.56324534376303	12.50003477072871	14.51602488002789
Pd	10.42931071433629	16.54486491870763	14.57342626873536

Pd	10.46401814171217	12.50016505250110	14.53942374344854
Pd	10.48313839726321	12.50021106242684	18.56132897656558
Pd	6.43522142741416	12.50014815874910	14.53184270631477
Pd	12.49081847518828	18.55398386525297	10.45460399164853
Pd	10.42936924495710	8.45513790679642	10.42653804016756
Pd	12.50688303370316	14.54094098727833	6.44461750956381
Pd	12.49058360515140	18.55413320290546	14.54494848753756
Pd	12.50665821871210	10.45909407589078	18.55534681745183
Pd	10.42926985524851	8.45539149934386	14.57371037512940
Pd	12.50683000942879	10.45862069930101	6.44470098390447
Pd	10.48323673378327	12.49979150376673	6.43864575840675
Pd	12.49061866627692	6.44601542609104	14.54542311432185
Pd	10.46407922948355	12.50000876912040	10.46053118437295
Au	14.49984639106011	14.50948541847627	4.41951558477679
Au	12.49933060879389	6.43421074412712	18.57261778649875
Au	8.35670135708760	10.36666875695958	18.63912735448579
Au	12.50684513767183	4.49868737902066	16.52274601382615
Au	20.49597777854575	12.50025264121270	16.50812382133658
Au	10.49295041236720	14.51025485427689	20.55459453024114
Au	10.36442975087229	18.63630970635155	16.63012073229881
Au	20.57488696636242	14.50486113959810	14.50195290642528
Au	20.57491380363879	10.49529105049527	14.50215867035171
Au	10.49310706646060	14.50985767237732	4.44532895309572
Au	10.33888491159782	8.33699289002368	18.66519261307235
Au	4.42558356111577	14.49939475400311	14.50763524217695
Au	14.66023634657452	6.33827331551365	16.66391244506911
Au	6.36079904142756	10.37822277876106	16.63001974026593
Au	12.49956967843671	6.43365329930406	6.42769522995678
Au	14.63286316587520	16.62748379192830	18.63565460879787
Au	14.50314864452655	4.44752551113233	10.48746735035827
Au	6.38623247630053	8.39459570403343	14.60365182548999
Au	14.66026780892958	6.33800278854446	8.33671903703621
Au	10.50103828314324	20.58096060330018	10.48568568781861
Au	8.39022615735504	6.38967979195498	10.39034203358203
Au	10.33902957448355	16.66326699503654	6.33459234012880
Au	18.64128574741081	8.36704462167324	14.61925846622790
Au	14.50330694836459	20.55241073709670	14.51276924551086
Au	6.36082658543502	14.62197193501421	8.36952827382492
Au	6.44856785152801	6.43387660358706	12.50000705510626
Au	8.39011980399579	18.61022368983983	14.60943157625421
Au	18.56455999007052	6.43816084926518	12.50049764070214
Au	6.44042528920259	12.49983968106150	6.44134002079677
Au	18.64126075986717	16.63306304475046	14.61893475855338
Au	16.67319777985932	8.31741769570097	16.66929879357891
Au	6.36101585864002	10.37781718055504	8.36966422418947
Au	6.38629627988071	16.60552255881568	10.39597753506690
Au	16.67309735068840	16.68288259243049	16.66895764758250
Au	14.66012835778475	18.66201958500704	16.66350567605254
Au	16.64574219164237	6.35872153598646	10.37070312853676
Au	12.51110108577411	4.36646462496886	12.50042737091301
Au	10.50087191162142	20.58112194374485	14.51368035169375
Au	14.50310354494637	4.44760205792882	14.51344165288791
Au	12.48823697393611	12.49982757294656	4.35906487209640
Au	8.39016178535897	18.61034044612678	10.38979476055517
Au	10.36459620474086	6.36362535538730	8.36984283929626
Au	12.51116916435593	20.63336468588646	12.49969660237466
Au	18.64145463011880	8.36685247363954	10.38131891991620
Au	10.33908484218725	8.33645634381996	6.33497298636247
Au	16.64568156387523	6.35878970254605	14.63009309773534
Au	14.49964905790014	10.48998670733833	4.41959548828206
Au	14.63312200584577	8.37247398825326	6.36443871303761
Au	18.56465574611600	18.56192968032283	12.49982241028215

Au	6.38632583673809	16.60562036638422	14.60329455902882
Au	16.64567055670070	18.64123131134935	14.62955054957935
Au	18.61477792709261	14.60747848374318	8.39728872769238
Au	10.49297723818441	10.49026930648457	20.55459314985604
Au	6.38640949054215	8.39444874018201	10.39635891238792
Au	8.32791367888056	8.32684385408196	16.67887867102470
Au	18.64146037057245	16.63288776736953	10.38101425763547
Au	18.55019257753892	12.50024669274154	18.56326078625080
Au	14.49936162021976	14.51009873491171	20.58058939815554
Au	8.35701611085867	10.36632246645039	6.36073241709748
Au	14.49951734522470	10.49050140144283	20.58061248342187
Au	4.42563849196312	14.49940308721269	10.49170248412378
Au	16.64588026628338	18.64110906714617	10.37004577518034
Au	4.42575322590677	10.50055963138768	10.49181389304475
Au	12.49928871238811	18.56637260361512	18.57235829746274
Au	16.60794295070167	10.38846265770825	6.39551228454428
Au	8.35677337008610	14.63370936846522	18.63898270707233
Au	10.33881254747987	16.66357057247232	18.66504534711642
Au	20.57505946720981	10.49508055776741	10.49828232397159
Au	4.37856892724622	12.50007346444443	12.49967191343802
Au	14.66056668863006	18.66166788556268	8.33608088050905
Au	16.60796286972174	14.61080871960609	6.39540216941829
Au	6.44860041503768	18.56621587329629	12.49957602126669
Au	6.44010563427867	12.50023389519289	18.55851964884924
Au	18.61451933637548	10.39247605292143	16.60303290739052
Au	8.39002977325203	6.38991849840707	14.60989497056246
Au	4.42562656610165	10.50086942949196	14.50774645661005
Au	8.32801206416696	8.32648632358515	8.32117289479229
Au	10.50099006653843	4.41887667231820	10.48630869883699
Au	20.62114769899529	12.49988714658268	12.50020135004197
Au	16.67345370121141	16.68258948137582	8.33089127045604
Au	20.57505521910964	14.50464330031467	10.49826568104259
Au	16.67323990680365	8.31709980069929	8.33127617726813
Au	18.55048323980465	12.49970538196265	6.43698895554957
Au	18.61480861403176	10.39205066749165	8.39737985613732
Au	10.50088470949419	4.41908844115133	14.51420742696970
Au	18.61454895007537	14.60791766080668	16.60286817186746
Au	6.36083141216267	14.62206681600169	16.62984143004275
Au	8.32800452666059	16.67332825117636	8.32084645451134
Au	16.60775719995132	10.38905172765817	18.60482409282565
Au	14.50339713092140	20.55234484980941	10.48665776209894
Au	8.35688143356992	14.63340944661418	6.36066990839215
Au	14.63332119587254	16.62693610030268	6.36424279041496
Au	14.63309605091094	8.37295186012768	18.63579236704119
Au	10.36434463759490	6.36405209067527	16.63048957323987
Au	12.48803704785280	12.50019374105971	20.64103499670292
Au	16.60766953724669	14.61147272073916	18.60477735031646
Au	10.49319147556974	10.48973358463791	4.44533349697801
Au	10.36457433014537	18.63606135870969	8.36926819964565
Au	8.32800268623577	16.67340064601469	16.67861239799143
Au	12.49969226491257	18.56583293123980	6.42720427590963
Au	16.50669101127603	12.50043818671871	20.51563590523063
Au	12.49281370644066	8.48575625377497	4.49281872524659
Au	16.50708293768725	12.49954778859354	4.48465303799467
Au	12.49919822309186	16.57941903772219	12.49988646788104
Au	12.49272951604422	8.48642537798691	20.50729195022637
Au	20.51650007803384	16.50065420421349	12.50006078899901
Au	12.50723457824646	20.50119545025701	8.47717912739307
Au	12.49257243758085	16.51434711453893	20.50732604909415
Au	12.50699137219838	4.49839804597358	8.47785755213710
Au	10.43324937089922	14.52395774437594	16.58029627561258
Au	16.49285998301278	20.49627727958882	12.49970373867575

Au	20.49615129927340	12.49988206922751	8.49224984553223
Au	4.48891563092445	12.50014886135317	16.50718173141637
Au	10.44512492665759	10.44700158397554	12.50006736351739
Au	8.49794615075092	20.52332020049352	12.49968561764820
Au	12.50053681555516	12.50019040116387	16.57564607639891
Au	10.48120610978858	6.48984651247293	12.50028330719232
Au	12.50075069308505	12.50007006058688	12.50000348906073
Au	12.49907661800106	8.42064016214381	12.50020742707589
Au	4.50155932851917	16.50272084988199	12.49966052333243
Au	12.50060631498978	12.49988084172469	8.42442895410855
Au	10.48124343025159	18.51021795449479	12.49969641898129
Au	12.49882420231510	8.44942561974064	16.55346563394138
Au	12.49300836756175	16.51354036914084	4.49253328322013
Au	12.50695468868728	20.50152422648968	16.52214559998127
Au	16.58496119265375	12.49995129084054	12.50009362468223
Au	8.50252419273196	12.50020009065956	20.49157035844164
Au	12.49881428303928	16.55094844411782	16.55331419531372
Au	10.44511985351298	14.55311153260645	12.49990389529315
Au	10.43307644045605	10.47646934902359	16.58032968860415
Au	10.43329160297282	14.52379074558324	8.41959309310496
Au	4.48908251576990	12.49987454287334	8.49251100459499
Au	12.49899388836523	16.55068343408786	8.44649019220721
Au	16.49270205145836	4.50379502961261	12.50043468093564
Au	8.49784001936731	4.47677615701295	12.50024982268740
Au	10.43327403156131	10.47603778022286	8.41975287403703
Au	20.51653794444657	8.49935368236096	12.50032133132946
Au	12.49895333372166	8.44905434854445	8.44683264017996
Au	8.50279573371780	12.49979599518650	4.50818676667229
Au	8.41541650886903	12.50005606163313	12.49994742547293
Au	4.50157181518050	8.49742451949469	12.49992751937026
Au	14.55612816623496	12.50009803911717	14.55158159706363
Au	16.57139369554490	14.55774818309713	10.47172122928210
Au	8.42917253660396	10.47238918537512	14.55640414532039
Au	14.51683868387537	12.49967924173801	6.49177604409468
Au	14.51660811542004	12.50031490279271	18.50841987262062
Au	12.50028249749519	10.46338710418841	14.53570508002440
Au	12.50029348983100	14.53681603063940	14.53554689616183
Au	14.56624607779212	16.58154359645210	10.47566190547124
Au	14.56603951582611	8.41850909391468	14.52451803379531
Au	14.56608299640476	8.41825198717658	10.47606246960623
Au	12.50036295323187	14.53665990696399	10.46430760707417
Au	16.57124756409506	14.55791325247630	14.52835385911198
Au	16.57123002395115	10.44215223126561	14.52848928590666
Au	14.55614143295734	12.49990663652734	10.44855089352045
Au	8.42930633892492	14.52785897597762	14.55620356046418
Au	12.50030230444700	10.46321064589373	10.46455012289458
Au	8.42928335881565	10.47210585143111	10.44356426313201
Au	8.42924502435713	14.52782414049365	10.44341341569864
Au	16.57135842070854	10.44196847894903	10.47188073180216
Au	14.56602516617777	16.58171232972043	14.52412053091101

$Pd_{43}Au_{158}L1_1$-685.519

Pd	8.44335533823601	12.49998568970323	16.55663698871194
Pd	12.49996126792181	14.52542964317260	14.52539998832978
Pd	10.49799876933513	18.55165407325025	12.50778041491094
Pd	12.49995754754998	10.47459039449776	10.47456261769699
Pd	12.49999209117660	8.44335441339907	8.44336630297440
Pd	14.50199087302789	6.44829221588483	12.49220919826589
Pd	14.52592962068056	10.47403528688662	16.53282753860713
Pd	18.55171669011866	12.50779290076028	14.50200596180425
Pd	8.46666048816778	12.49157086917137	8.46667868655712
Pd	10.47405886306592	16.53275439594149	10.47408033137260

Pd	12.49153705398079	8.46662333174698	16.53335672311696
Pd	8.44337081217224	8.44336716269285	12.50000231486632
Pd	14.52591073772876	8.46717487561992	14.52592960330730
Pd	14.50198399045151	12.50779217868405	18.55170626777093
Pd	10.49798809590583	12.49222284169387	6.44834373917364
Pd	10.45549495779800	14.54447270597192	16.55265801427904
Pd	12.50840810962739	16.53333789033348	8.46664436272918
Pd	8.46721116037924	14.52591277531355	10.47407690754440
Pd	8.44735235200002	10.45551491047697	14.54447340967045
Pd	16.53279503203422	10.47407505174837	14.52590718831189
Pd	12.49999048949900	16.55666062928259	16.55666417891826
Pd	12.49218904987547	6.44833182962530	14.50197462949636
Pd	14.52540638258844	12.49998542341408	10.47458663086648
Pd	18.55171564934413	10.49797369486948	12.49220153357473
Pd	14.54449010434474	16.55265827771014	14.54450617033047
Pd	6.44833670853893	12.49222153486466	10.49801078603685
Pd	10.47458700481020	10.47454045943304	12.50001009509194
Pd	16.55666354724816	12.49998789388023	8.44331965016721
Pd	6.44834028815891	14.50197222902932	12.50777914370323
Pd	10.47456259794303	12.49998446685451	14.52542277914816
Pd	14.52543686428227	14.52537962764297	12.50001045015692
Pd	10.45549035193573	8.44737338950550	10.45551552030942
Pd	16.53338510063797	12.50845040158716	16.53338425161868
Pd	12.50776012291076	18.55168775784068	10.49798970336163
Pd	8.46664515640345	16.53332171703249	12.50841628142315
Pd	16.55263666044465	14.54451902255513	10.45546619032297
Pd	12.50775605947876	14.50200175993333	6.44831307625360
Pd	10.47407916031869	14.52588596320052	8.46725163039542
Pd	12.49997977301169	12.49996649973029	12.49999165885140
Pd	16.55668234031603	16.55668231574025	12.49999105944021
Pd	12.49218772697647	10.49801491524577	18.55166777068124
Pd	16.53336959054784	8.46660719547561	12.49153600446569
Pd	14.54449334797424	10.45547602733864	8.44737572608056
Au	20.49214551339303	8.50465403551676	12.49681890185231
Au	16.52290802539934	12.48971662357500	4.46489026689815
Au	10.46162731865678	4.36280568488254	10.46162526759497
Au	10.42844866636933	10.47500411895227	16.54180810936237
Au	12.49648419812390	12.50343446401661	16.53429762135999
Au	18.66056657284489	16.66719340088870	14.63757833502621
Au	10.43057798810679	18.56906375826108	8.43381142562077
Au	14.52568270755446	12.50945858455385	14.52556189845294
Au	10.47429148372842	14.52570183876500	12.50944803268622
Au	18.60141257861486	14.60892553290943	16.59144966455052
Au	10.46153174243302	6.45888052297505	12.48769461742651
Au	10.47497226167870	16.54181770877588	14.57147666971623
Au	14.56943965620682	6.43087865583939	16.56622812251147
Au	16.52290995489595	20.53512466284107	12.51027125958167
Au	8.36504768799038	8.34002992132472	8.36507917062124
Au	10.38294028170924	6.37073456154360	8.37897162754741
Au	16.58279194388212	8.41716013962368	16.58283335226686
Au	12.50354174694586	18.54107068118250	6.45892217575424
Au	16.53415746356974	12.50341336985833	12.49659668038766
Au	10.36241548749757	18.66051161465575	16.66716689802711
Au	16.59143062951864	14.60893513647893	18.60140156170964
Au	14.61704996239785	8.37893890405465	6.37072313215468
Au	18.56243468001484	12.49999284906343	6.43755343950602
Au	20.53512632180107	12.51026514085531	8.47708233386808
Au	12.49680124518200	4.50786207175528	16.49531566624326
Au	10.38292156965908	16.62105088683490	18.62930297384667
Au	16.63496457203931	16.66000451638256	16.63499282007891
Au	18.54109210292256	6.45890506543697	12.49643874916204
Au	8.40858052197564	18.60134092684287	14.60886151423450

Au	8.40861006539980	10.39115353445619	6.39869113702138
Au	8.33283524455394	10.36242529029080	18.66049649396514
Au	12.49885129278582	4.32794487918923	12.49885298275384
Au	14.57151116715624	16.54182956128652	10.47492669811211
Au	14.49417014783499	10.50582856630707	20.56355451832419
Au	10.49187675263572	20.58221410794505	14.51832805097137
Au	6.39865385886191	16.59138262945272	14.60886462315470
Au	12.50333825849462	12.49659651334504	8.46577434396255
Au	8.45814664847953	14.52499231320657	14.57148659262231
Au	12.49680657944040	8.50466293480069	20.49213470049440
Au	20.56356794119005	10.50582026386700	14.49416693562413
Au	6.39868739392937	10.39114681379351	8.40863219943780
Au	12.49047453191212	10.47431117150490	14.52560934150326
Au	10.47433212752742	12.49054550561245	10.47425506253534
Au	6.43095940970583	14.56939685784892	8.43384517437801
Au	16.54177405333319	10.47495663906906	10.42843799938453
Au	4.46490623678486	8.47710212645554	12.51028035274507
Au	12.49885598695392	12.50113504355350	20.67208868115767
Au	10.48164741921068	4.41780731252051	14.50808762519820
Au	12.49998556099474	6.43760007753282	6.43759786857216
Au	18.60137601960288	8.40855694012792	10.39107888329425
Au	16.54179146977640	14.57152617846343	14.52500064797926
Au	12.48766622197161	14.53844192356176	18.54115894969839
Au	16.66715783299454	14.63757898139671	6.33946608208491
Au	8.50465797592650	20.49212063865963	12.50317085784433
Au	6.43095534303100	16.56616217029519	10.43058951039468
Au	14.49416039230261	4.43645305734923	14.49414950711711
Au	10.46162324694469	14.53837438302524	20.63723657123568
Au	14.53844208588499	18.54119283719539	12.51228738964147
Au	14.51831811931023	14.50809925276805	4.41779058012186
Au	8.37893612640947	14.61706117811224	18.62928149577943
Au	4.46489711271583	12.48971683603489	16.52292001688189
Au	10.39109324307575	8.40858697662970	18.60134753161898
Au	8.43381800687761	14.56938949720571	6.43095655692391
Au	14.53834707188786	10.46164977096235	4.36280270514938
Au	10.39109671089507	6.39864353497232	16.59139150467784
Au	16.62107772022005	18.62932892570610	14.61707573763629
Au	6.45886686249934	12.48769693886018	14.53841837221506
Au	6.45886093693904	10.46156306772942	12.51229761541431
Au	18.56245568929565	18.56245759875410	12.49999487397809
Au	18.54120901515995	12.51229694593981	10.46148300019841
Au	8.46566642896812	12.49658264126905	12.50343716835178
Au	8.47707066815830	12.51026982927319	20.53513720285128
Au	12.48971217567302	16.52293220898498	20.53515672832701
Au	16.56622517642601	6.43085992815930	14.56943736994018
Au	4.50790184156930	12.49682978797951	8.50468970024729
Au	10.47496311026384	10.42850448586027	8.45821037012890
Au	14.63759001892812	16.66717935065416	18.66054131959635
Au	4.36281445155129	10.46165300436492	14.53834598144311
Au	18.54111237894872	12.50355186541140	18.54110264644332
Au	12.51226349037917	18.54115347242982	14.53843689679583
Au	14.60887472143741	16.59141363404975	6.39863669186645
Au	6.37074340216228	8.37896554749258	14.61705229885620
Au	12.50334471789803	16.53428499582656	12.50342779803336
Au	10.36243510194426	8.33285636890117	6.33951532496171
Au	20.63725202764644	14.53837779996267	10.46161043795277
Au	12.49998599574335	18.56243992773384	18.56244203149766
Au	14.52565217216110	10.47438852739243	12.49053037546843
Au	14.50808648256925	4.41779024151960	10.48165096876225
Au	12.50315414990013	20.49213672271510	8.50466110877124
Au	20.58226932316099	14.51836944254647	14.50810224996916
Au	16.66716912213632	18.66053385870092	10.36240130667846

Au	12.48970644543212	4.46489013051079	8.47709188239522
Au	8.50467584049394	12.49682978470803	4.50790978650663
Au	8.47707481273529	4.46489757438535	12.48971910245056
Au	14.51832358392840	20.58223304387992	10.49188614688779
Au	14.63756819221911	6.33948282607140	8.33283551428326
Au	14.60888074871473	18.60136200636396	8.40857596959969
Au	16.62105356869192	10.38293107055879	6.37069729274711
Au	12.51226860994553	10.46152677008630	6.45883241931731
Au	12.50939298181529	14.52564947622805	10.47426501555403
Au	10.42846239644272	8.45819033377439	14.52496323034921
Au	10.46155098904522	12.51229655448152	18.54111263762742
Au	18.56915035991746	8.43373862863842	14.56942605444341
Au	6.33952276309065	8.33286666393681	10.36243292393630
Au	12.49643169967387	6.45891293596839	18.54106907647527
Au	12.51026229476326	20.53512679333755	16.52293405014483
Au	10.50583992385695	14.49415588843821	4.43650674282040
Au	14.52500559639490	14.57155626538366	16.54182946401499
Au	10.43058787981666	16.56618039853514	6.43093317740757
Au	8.33283400938080	6.33950555803928	14.63755150004310
Au	16.63494065644164	8.36502119971069	8.34001235898706
Au	14.53835784626951	20.63722829207857	14.53835520222560
Au	16.49532096582944	4.50785341349436	12.49681926452935
Au	20.67212778443950	12.50113654275344	12.49885135466509
Au	18.56915265132823	10.43055705635175	16.56625169869057
Au	8.45816889976305	10.42847827385404	10.47496823096222
Au	8.43380377056308	18.56904433516422	10.43058487233575
Au	14.61707420441370	18.62930964592702	16.62108698551539
Au	4.41781497691309	14.50809749200690	14.51831139545242
Au	18.62933680752883	16.62108133465664	10.38291964404452
Au	12.48766841534784	6.45883219895510	10.46152475801824
Au	12.50111491082429	20.67206453437917	12.50112054672122
Au	14.53845882993008	12.48770164850638	6.45879023278078
Au	18.54120696942784	14.53849527596750	12.48768835796450
Au	18.66053668806549	10.36242092025152	8.33281785068982
Au	4.50788138984239	16.49531771793668	12.50317522153619
Au	16.56623295611267	10.43053866504068	18.56913537239301
Au	12.49648852062331	8.46574971451400	12.49658225900018
Au	14.50810457291870	14.51835397657378	20.58225324068949
Au	12.50111519426373	12.49887735108696	4.32797067775799
Au	6.45896301488627	12.49644342587534	6.45896548409756
Au	6.43759852372902	6.43761833102375	12.49999520677033
Au	20.58224692237950	10.49188662321709	10.48162611584656
Au	6.45893553819594	18.54105237452233	12.50355244970665
Au	14.56944728090050	8.43375249446915	18.56911061267617
Au	16.59140114070046	6.39862517661912	10.39107623748477
Au	6.33950279944989	14.63756994324716	16.66714985399475
Au	6.43758559944585	12.49999288911548	18.56240668925586
Au	16.65998538778226	16.63498276092039	8.36501324454003
Au	20.53514240085477	16.52292114959821	12.48972625047498
Au	8.34000490735027	8.36505908411330	16.63493259511043
Au	18.62933254736150	14.61706297575449	8.37891487579110
Au	8.36502249722750	16.63494502857237	16.65999818763031
Au	4.43651200402140	14.49414577615422	10.50584828951660
Au	4.32799546039870	12.49887614579414	12.50112087230372
Au	12.51026459789223	8.47709382283507	4.46491602132038
Au	14.57149266972622	14.52503948803150	8.45817217872342
Au	10.48164708683837	10.49188449184178	20.58221672648189
Au	12.50315536748254	16.49533115747195	4.50787538734629
Au	10.50583443828911	20.56351239557168	10.50583223848631
Au	8.37895315010363	6.37075425131662	10.38294486603850
Au	4.41783061196637	10.48169310863191	10.49189666332977
Au	20.49216592224705	12.50316989643754	16.49534783350779

Au	10.49189203590918	10.48168205582418	4.41782867724319
Au	14.52501365311384	8.45818405446709	10.42842117068240
Au	6.37074470881996	10.38293253178693	16.62104028027173
Au	8.41722808472054	16.58274012183390	8.41726618246216
Au	16.49533928841149	12.50317071801556	20.49216877945173

$Pd_{43}Au_{158}$LEH-687.399

Pd	6.43917425396272	10.48365528441463	12.49315811151152
Pd	18.56573857593971	14.52047825266606	12.49208026484913
Pd	16.56569934649581	16.55197119134480	12.49040927459586
Pd	10.42716547006311	14.55753160852286	16.55278549778774
Pd	8.43689805810295	12.50205941000806	16.56908558689893
Pd	10.46357004916177	10.48227568670157	12.48916643936979
Pd	10.47254829619220	6.44947800700273	12.49384380215338
Pd	14.54135640024584	14.52921798086873	12.48364700700526
Pd	14.53639701805965	10.48237567503584	12.48929528559147
Pd	14.52739016605629	6.44947082837256	12.49396419881143
Pd	10.47641036907238	18.53727638740384	12.49349272170548
Pd	16.56853344383909	12.50244335647531	8.44346967344504
Pd	16.56299264520094	12.50197369902049	16.56929095648076
Pd	10.45871720034549	14.52924454787385	12.48353349968322
Pd	8.43154961765476	12.50251126186039	8.44320070141725
Pd	10.43295089455665	10.45394187777824	16.54612013550190
Pd	6.43421900195381	14.52053072544298	12.49186430562401
Pd	12.50014725761717	16.54652351948544	8.44600100966987
Pd	14.57289930029913	14.55752642280200	16.55279357786395
Pd	14.52366380739971	18.53717427092095	12.49360796624808
Pd	14.56692772035241	10.45397645008938	16.54614756515237
Pd	18.56062055538940	10.48361547517730	12.49342979627558
Pd	16.56365268847797	8.45010371509904	12.49213824166581
Pd	8.43443227864286	16.55195878774370	12.49024064973144
Pd	8.43624660828183	8.45010387715626	12.49179031121467
Pd	12.50003921946253	8.45808359160782	8.45563375214175
Pd	18.56112001775953	12.50294519798079	10.47451765361089
Pd	6.43890956907051	12.50299939573519	10.47431433172206
Pd	12.49995400833487	14.52405076490632	18.57757562170054
Pd	14.54090506063225	12.50110091056520	6.44321483939372
Pd	12.50002549230292	6.44939606043827	10.47104769879662
Pd	12.50000770853530	14.52608502219713	14.50994401883845
Pd	18.55903506639762	12.50257763352032	14.52656469948129
Pd	6.44078286205396	12.50257802241488	14.52635574751641
Pd	12.50008240550561	18.54088472223971	10.46766252987234
Pd	12.50019540904646	14.53614043295744	6.44722567835792
Pd	12.49998647540719	18.51687879649923	14.53933955124767
Pd	12.49989579084485	10.48274693034243	18.57006662930262
Pd	14.53731498320092	12.50677533821772	10.46837058822846
Pd	12.50013969304936	10.46658072288066	6.44836032904844
Pd	10.45937830781761	12.50113649641127	6.44310376086629
Pd	12.49990024983398	6.46911735585359	14.54313316117021
Pd	10.46284653807653	12.50678002005381	10.46830665078226
Au	14.50200889592540	14.49660312313210	4.44192049946781
Au	12.49978450903726	6.45233839439464	18.57033348596430
Au	8.38159860998471	10.39029319106538	18.61759006936681
Au	12.49988710097587	4.51092472313707	16.51605237937653
Au	20.51497442117617	12.49925332955896	16.51151846549290
Au	10.49221515101919	14.49980538184214	20.60220605200761
Au	10.35238845177457	18.62736136469153	16.65039209065754
Au	20.56858471293885	14.49557257163719	14.49862472785870
Au	20.56146370509593	10.50497441256246	14.49605996002999
Au	10.49838117365110	14.49663363337698	4.44185486506687
Au	10.37392672899754	8.39443384865350	18.63291068165567
Au	4.43124744052488	14.49553310465338	14.49828105200582

Au	14.64177151423932	6.36344628417151	16.64859703060279
Au	6.38074423606179	10.39891959122198	16.61066747831248
Au	12.50011541549337	6.46799244649263	6.46172450682933
Au	14.62984518666294	16.60514762066771	18.63946267209874
Au	14.50092762298938	4.43638199348439	10.50365470472440
Au	6.37875091122161	8.38657859310783	14.60980167824029
Au	14.62314638301948	6.38482695805715	8.38283446180909
Au	10.50015418262924	20.55211151328013	10.50332593891894
Au	8.37878299599963	6.37712884010114	10.38941271932761
Au	10.36490849156139	16.62788251152255	6.36931952141223
Au	18.62105403809423	8.38657596567786	14.61016032131625
Au	14.49273559736536	20.52916970209442	14.50397675950991
Au	6.37096564617018	14.62586940348650	8.37471317831516
Au	6.44301807204802	6.44599008037707	12.50664224075929
Au	8.38199617517509	18.60377358485015	14.61136564725929
Au	18.55690864254624	6.44588573646853	12.50674161409669
Au	6.44745467573370	12.49923539532612	6.44853934940880
Au	18.63006564186120	16.61201403670714	14.60874269681869
Au	16.66013308492717	8.34169288158198	16.66958842831541
Au	6.37801760009945	10.37794184107323	8.38106977781066
Au	6.37557507488529	16.61257949749306	10.38688424052496
Au	16.66844999883186	16.66077530408346	16.67666406624825
Au	14.64756072858019	18.62731600243109	16.65049759731995
Au	16.62122566174902	6.37707752427091	10.38962189971258
Au	12.49990661741782	4.40106001844707	12.50648314139751
Au	10.50731062700053	20.52926697078490	14.50393506834930
Au	14.49396390080540	4.45668733978591	14.50714505779541
Au	12.50016424679907	12.50183628469219	4.38321573630024
Au	8.37161489246026	18.62105586098715	10.38100232590565
Au	10.37701040319404	6.38476635901993	8.38268752721788
Au	12.50006957144665	20.59101674574412	12.50394188745212
Au	18.61522489563901	8.38885692803943	10.38968937607651
Au	10.37096915552379	8.37647884181251	6.37844198850769
Au	16.62289927379990	6.37791128425201	14.61377970534746
Au	14.49945702286488	10.50444294907279	4.44299802535607
Au	14.62932836795012	8.37641626838834	6.37855998164920
Au	18.56259622121931	18.55193652270055	12.50411887186974
Au	6.36991268608835	16.61211118722752	14.60851402785592
Au	16.61797692596598	18.60369933924409	14.61159601283024
Au	18.62920529848585	14.62564445469896	8.37500034235963
Au	10.49862095533233	10.49890004982513	20.59812300264249
Au	6.38473372670247	8.38884439140404	10.38934642018877
Au	8.33952881018559	8.34173679778895	16.66937862777941
Au	18.62448572809767	16.61244501759361	10.38706301619290
Au	18.56122040016530	12.49645477064782	18.57158261285591
Au	14.50741815952742	14.49990415660694	20.60223906543259
Au	8.36794292821879	10.37453449687840	6.36874289496037
Au	14.50098714781646	10.49883948754598	20.59826312451887
Au	4.42486456675842	14.49973034974935	10.50070932694016
Au	16.62854989128433	18.62092456535235	10.38104535914755
Au	4.42725022125504	10.50173241473494	10.50418059589486
Au	12.49983071948477	18.52757308223637	18.55904255021765
Au	16.63235795935792	10.37444958661049	6.36895943907194
Au	8.37590082562960	14.60959464479413	18.62137765852607
Au	10.36992094785486	16.60511134430449	18.63934988788753
Au	20.57271769537664	10.50166292876785	10.50462418147991
Au	4.37060926133345	12.50178821204280	12.50001035731920
Au	14.63050954519690	18.61711457391021	8.37397322006453
Au	16.63796974880881	14.62836037074751	6.36509261470958
Au	6.43751678309679	18.55195797052781	12.50398319158730
Au	6.43849860916319	12.49640873385886	18.57126466003737
Au	18.61897388660280	10.39884304062115	16.61094677917612

Au	8.37697519493082	6.37803804929135	14.61360338053630
Au	4.43819160314623	10.50502913262058	14.49569659112844
Au	8.27800810415085	8.28508385351834	8.28475675897841
Au	10.49912179854167	4.43645364914279	10.50355606743071
Au	20.62924193246930	12.50169400380555	12.50025530527829
Au	16.73358659289201	16.72495908835879	8.27258814564825
Au	20.57527110056136	14.49951839202154	10.50105004134577
Au	16.72217491224305	8.28501790762946	8.28496468284211
Au	18.55289249819666	12.49910925252036	6.44886769699253
Au	18.62209426502189	10.37787756157298	8.38142647676375
Au	10.50575003933842	4.45685241146442	14.50715831375681
Au	18.62523166343832	14.60111256167267	16.61461939647041
Au	6.37457228250080	14.60107255345892	16.61433113422896
Au	8.26656021120866	16.72507147938124	8.27249061460830
Au	16.61805504085254	10.39028779270371	18.61784495099180
Au	14.50005265522828	20.55197441654157	10.50337300926680
Au	8.36236096697178	14.62849758369249	6.36494920025530
Au	14.63545220798698	16.62788934746346	6.36931688166617
Au	14.62572441280720	8.39440328229896	18.63294928874314
Au	10.35795031884695	6.36354060418993	16.64861045489981
Au	12.49989176868468	12.50122469046466	20.70534889350563
Au	16.62380784425660	14.60965906434942	18.62159572108301
Au	10.50089204985511	10.50450182613881	4.44292939412686
Au	10.36968634368800	18.61706105433530	8.37391818299989
Au	8.33146596345440	16.66080122416146	16.67650616285331
Au	12.50023112241563	18.53409918281266	6.45908074175345
Au	16.52109490413841	12.49594338469714	20.52910609077276
Au	12.50015438434519	8.50256875827446	4.49440771387029
Au	16.50483150065364	12.50024910263746	4.49107391864265
Au	12.49999846295744	16.55872198279980	12.50284518851874
Au	12.49975489973305	8.49819088400959	20.51475086871891
Au	20.51667341386169	16.49841591172900	12.49782276512706
Au	12.50008153155769	20.49024919021971	8.49853091932777
Au	12.49983177089654	16.49567417609367	20.52406996348942
Au	12.50009167703822	4.50333793267033	8.50058597095826
Au	16.49288044742907	20.49003903741618	12.50204107513101
Au	20.50180950040558	12.49712702217297	8.49877980103700
Au	4.48465901886533	12.49917550733048	16.51122766497108
Au	8.50722928488642	20.49016185815881	12.50188491720008
Au	12.49990774849160	12.52684052722154	16.54823481710328
Au	12.49999375581607	12.52563115940784	12.48838209596711
Au	12.49997033502489	8.42037151798732	12.50012278099632
Au	4.48344793923916	16.49859155554587	12.49760107920421
Au	14.55446744990886	10.47285844959561	8.43313660963184
Au	12.50007143256124	12.50232217456317	8.39750488135531
Au	12.49987050134833	8.49825360009401	16.56281251198205
Au	12.50012184479836	16.49576684042652	4.49469171166605
Au	12.50002903588775	20.47173332034300	16.51058030924487
Au	16.57307939157014	12.50458505987737	12.49150637112894
Au	8.47831212454121	12.49601040194582	20.52884782163234
Au	12.49995502791973	16.47852219373583	16.54685371377650
Au	10.44054311732831	14.53273637059404	8.42619062724433
Au	4.49851446913197	12.49729896211472	8.49837948368402
Au	16.49242005963378	4.49330456389200	12.50375973016060
Au	8.50751853755961	4.49341236873108	12.50355826804003
Au	10.44567738722363	10.47291300112808	8.43302656101862
Au	14.55966037775746	14.53274522049880	8.42631488011285
Au	20.50700241533378	8.50012073742154	12.49620727467474
Au	8.49567526057511	12.50034823399922	4.49084307662687
Au	8.42684640703604	12.50459214171069	12.49131293656928
Au	4.49288789574605	8.50017588246939	12.49577507638335
Au	14.53851180933714	12.52830724299557	14.54163808933940

Au	16.56358589125555	14.53108260789085	10.45751464286881
Au	8.43689590634034	10.46886014551884	14.52835289054727
Au	14.50696977837598	12.50276030861829	18.52425088460067
Au	12.49997815968021	10.48414732362499	14.51588448960471
Au	10.44505246547684	16.56002671872515	10.45957733094199
Au	10.44933022397831	16.52996932862348	14.52047766868368
Au	10.46142922408938	12.52827924794286	14.54155824287215
Au	10.49277178142427	12.50263735781789	18.52422253077689
Au	14.55506506024837	16.56005058829373	10.45965331488752
Au	14.56652571505105	8.44733057098702	14.51847539633906
Au	14.55138785773686	8.44276945581950	10.46666233325120
Au	10.44864293195726	8.44270649215093	10.46659481217693
Au	12.50008251527776	14.51716494563848	10.47020514596981
Au	16.57405245308638	14.54312303074182	14.53216403432419
Au	16.56295694203295	10.46893184041234	14.52852992792403
Au	10.43328390082739	8.44731808071521	14.51835886066303
Au	8.42587926447166	14.54313418948021	14.53204400991656
Au	12.50002279791486	10.49540614115578	10.48397850745531
Au	8.44347179187706	10.47635624057218	10.46315455104023
Au	8.43648505338926	14.53109359899091	10.45736366522469
Au	16.55654094364698	10.47631594236842	10.46335136601483
Au	14.55063934398176	16.52989516152183	14.52062599657779

$Pt_{40}Au_{161}L1_0$-696.745

Pt	14.50205533083361	12.50010524947053	14.53815317434710
Pt	16.52482479436280	14.54803525936456	10.44590366409013
Pt	18.52110452815261	12.49994291429826	10.47312147078101
Pt	8.47519039344925	10.45200982437314	14.55406407457561
Pt	6.47903792305551	12.50006455742317	10.47291074843441
Pt	12.49988718529658	14.50001321399623	18.57812316324678
Pt	14.49975961966203	12.49981304843250	6.42204894584920
Pt	14.49957650878852	12.50011352127926	18.57801869748786
Pt	12.49990881811968	10.49797775041267	14.53820416285664
Pt	12.49992314339763	6.47889514062218	10.47332043368708
Pt	12.49989096280643	14.50219602378653	14.53796461589898
Pt	10.45193320352181	16.52477655576710	10.44572314966167
Pt	18.52107171235687	12.50010946166636	14.52718962790744
Pt	10.45183871693122	16.52481152420896	14.55384779578733
Pt	10.49782339628011	12.50012732861502	14.53807925138628
Pt	10.50021622770040	12.50012110098779	18.57810060320365
Pt	14.54806111464170	16.52471639524071	10.44587142889989
Pt	6.47902405075623	12.50007461828416	14.52697679708702
Pt	14.54795071454400	8.47527862512323	14.55412314809376
Pt	12.50000306149435	18.52104123374322	10.47285622309847
Pt	14.54802101841124	8.47524434156369	10.44623999959854
Pt	10.45186227617832	8.47523878112614	10.44611339026029
Pt	12.50010741343542	14.49959792600408	6.42195027273857
Pt	12.50000030254840	14.50204722287618	10.46184599172100
Pt	16.52477976507447	14.54816410939705	14.55407504739964
Pt	12.49990326318133	18.52107589788788	14.52663467259629
Pt	12.49988959084395	10.50025497348554	18.57806014025229
Pt	16.52473601210196	10.45208867685860	14.55417372423803
Pt	14.50210188003480	12.49999589536964	10.46200029659836
Pt	10.45180990672632	8.47520081411039	14.55406976985406
Pt	8.47520845107030	14.54815012045281	14.55398252473926
Pt	12.50010404926550	10.50006231824653	6.42199936061695
Pt	10.50043304281374	12.49985401524227	6.42196994271576
Pt	12.49995894235126	10.49790222437730	10.46206750209317
Pt	12.49984918094698	6.47887603323487	14.52701998272093
Pt	8.47526236842531	10.45194174729013	10.44594624592964
Pt	8.47518799266942	14.54813273083369	10.44572278383325
Pt	16.52478026322502	10.45191861491544	10.44617514481970

Pt	14.54798675175788	16.52483267400602	14.55384905609086
Pt	10.49789625980779	12.50000881259212	10.46189095034358
Au	14.51982525227600	14.51953289219955	4.41959232461054
Au	12.49985230803222	6.41951800346237	18.57214251405807
Au	8.33581986244603	10.34088685953238	18.67458456807659
Au	12.49984077555955	4.51038261975386	16.50011002182621
Au	20.48957197567239	12.50019542423279	16.50019090791058
Au	10.48017661254896	14.51989077255921	20.58048198825481
Au	10.38263194252819	18.61118812441154	16.60254394104681
Au	20.60044421790683	14.49511236705311	14.50138670211568
Au	20.60028641334129	10.50494743516908	14.50158505583619
Au	10.48043084537889	14.51962967348120	4.41962435182627
Au	10.34064456222359	8.33577771383452	18.67471664493929
Au	4.39959606366040	14.49518760397531	14.50119226665631
Au	14.61705164010902	6.38894634694987	16.60282022713581
Au	6.38896161137560	10.38280097023902	16.60287032635703
Au	12.50008087755151	6.41946114632082	6.42824651016484
Au	14.65898349687119	16.66435504719657	18.67439097041520
Au	14.49505139049228	4.39942903914900	10.49890729548087
Au	6.34664547453418	8.35705639704347	14.62912075043795
Au	14.61721927353306	6.38886742166825	8.39760052979601
Au	10.50501592166388	20.60056447788600	10.49848529837707
Au	8.35699036165206	6.34638790746423	10.37108922565378
Au	10.34071443037225	16.66437150569816	6.32516731112014
Au	18.65336845928757	8.35701699940183	14.62936947481452
Au	14.49510322172517	20.60044494488781	14.50109969979143
Au	6.38892071557338	14.61737862117717	8.39691903804865
Au	6.39597072771698	6.39597632908308	12.50012198920980
Au	8.35702722389624	18.65365612973449	14.62871647373386
Au	18.60397881400537	6.39604501472119	12.50046882913969
Au	6.41980326084122	12.49985703804589	6.42796993701558
Au	18.65349786160101	16.64307852266460	14.62896708275644
Au	16.65740001624414	8.34246459798050	16.64817666394968
Au	6.38905653747412	10.38260681141522	8.39714573081190
Au	6.34655378845090	16.64308651648351	10.37061044160389
Au	16.65754509131087	16.65768698104976	16.64799533349558
Au	14.61713837795618	18.61109386536637	16.60251234347090
Au	16.64286023975739	6.34651848261862	10.37136479553313
Au	12.49984126161087	4.31731800510796	12.50001897483516
Au	10.50480759471748	20.60057784420174	14.50111730322032
Au	14.49493003199729	4.39939400514743	14.50141651533262
Au	12.50012587420216	12.49983590365023	4.34001851593441
Au	8.35711639692078	18.65362039801486	10.37066559344885
Au	10.38275853875484	6.38887199517612	8.39744962537693
Au	12.49997388625123	20.68249369854339	12.49987699094527
Au	18.65343571636864	8.35703141048973	10.37133783387389
Au	10.34092585955857	8.33562230435009	6.32549968928262
Au	16.64281573533433	6.34651166459131	14.62924643646638
Au	14.51980260216463	10.48012237185602	4.41959761247145
Au	14.65926982847262	8.33555373005471	6.32554262301265
Au	18.60397654726769	18.60387878185538	12.49981409652456
Au	6.34653132746408	16.64309003914886	14.62881671291537
Au	16.64296030510701	18.65357609928132	14.62887659101035
Au	18.61123194721006	14.61713388068118	8.39728272413972
Au	10.48024722019261	10.48042541114003	20.58055140978220
Au	6.34653919178923	8.35691647795873	10.37096768103938
Au	8.34242181499057	8.34238830536295	16.64821468626090
Au	18.65354127180207	16.64289973458994	10.37092093696080
Au	18.58028904688295	12.50026924266807	18.57204529518490
Au	14.51960498549361	14.51991166403093	20.58044781595473
Au	8.33589517890689	10.34048882286029	6.32536178283620
Au	14.51964147745701	10.48037284992942	20.58043723046499

Au	4.39976450650224	14.49509194905403	10.49849488093140
Au	16.64302657962901	18.65342307368135	10.37077242736091
Au	4.39964466760726	10.50479952315965	10.49856986709192
Au	12.49980966514139	18.58053839991788	18.57179151696571
Au	16.66434302130779	10.34036032674228	6.32555754101815
Au	8.33557597401854	14.65958162978887	18.67458467069495
Au	10.34057282059323	16.66449383685692	18.67455341168263
Au	20.60048220432043	10.50481581591182	10.49886924909977
Au	4.31778143258960	12.50001995275613	12.49990576865116
Au	14.61731210636611	18.61097199603380	8.39716882134434
Au	16.66436098841935	14.65907336166500	6.32551598608720
Au	6.39618968725510	18.60406606498632	12.49955918997030
Au	6.41959480178682	12.50022454093868	18.57185528754792
Au	18.61098603680597	10.38289545494671	16.60301422227207
Au	8.35698031891932	6.34642788269088	14.62910494327270
Au	4.39968595115950	10.50489675810982	14.50143574711142
Au	8.34239495470056	8.34225324564479	8.35188851179436
Au	10.50482459385483	4.39950201797571	10.49880141514280
Au	20.68247625457673	12.49992106655671	12.50021778740724
Au	16.65771860552226	16.65758190574596	8.35178436943618
Au	20.60062971928645	14.49501307880185	10.49874031004513
Au	16.65762473644434	8.34221573568280	8.35210078375761
Au	18.58050755346043	12.49968065421430	6.42832009640907
Au	18.61112358604053	10.38249836997936	8.39746621952396
Au	10.50492091302375	4.39943091841874	14.50139125429840
Au	18.61105003909487	14.61752094973573	16.60281399129934
Au	6.38887064703778	14.61753183309651	16.60269068326216
Au	8.34243917239625	16.65764575751480	8.35164902700286
Au	16.66417032394990	10.34076802397741	18.67465231643374
Au	14.49508293600237	20.60042402177632	10.49861315144440
Au	8.33579041493930	14.65932537171772	6.32521747805698
Au	14.65958326270613	16.66422081443713	6.32528150346505
Au	14.65913109272427	8.33573577089893	18.67462941688066
Au	10.38264668035577	6.38894453289840	16.60281346815334
Au	12.49997101909253	12.50018993548609	20.66006006192070
Au	16.66415262436189	14.65956500460388	18.67456902484821
Au	10.48045299709671	10.48014482869179	4.41951416803217
Au	10.38287670923189	18.61111740579502	8.39702052059745
Au	8.34237219008361	16.65772885710722	16.64794524019574
Au	12.50018347766792	18.58042914693449	6.42787377319650
Au	16.49882357377804	12.50012004719621	20.48818087945908
Au	12.50014955283108	8.50080354843214	4.51167205785469
Au	16.49918077336324	12.49972647484262	4.51194531283651
Au	6.45613639819836	10.46767481221852	12.49998786399417
Au	12.49995984644530	16.54077813197921	12.49978352621693
Au	12.49991970935278	8.50095831183446	20.48842341732566
Au	20.53980738709742	16.56083283438162	12.49996017767329
Au	12.50014368595464	20.48957532862276	8.49984413069343
Au	18.54403828910362	14.53240091730800	12.50002687255032
Au	12.49983703519823	16.49917221485522	20.48833559325986
Au	12.49997316626797	4.51043343953713	8.50036535212734
Au	16.56305708585274	16.56302062893453	12.49988460340549
Au	10.44042565694346	14.55965118925704	16.58053454195667
Au	8.46137392316739	12.50016646453760	16.54863944002365
Au	16.56086479190941	20.53972473893944	12.49981064727761
Au	20.48964407375899	12.49979573619611	8.50025134345409
Au	4.51053298524376	12.50015799332226	16.49985882637831
Au	10.47089456788752	10.47103604858823	12.49995236273501
Au	8.43920824133781	20.54001727524658	12.49976019460908
Au	12.49990026063606	12.50002890319918	16.55949691768579
Au	10.46748425310404	6.45596564085462	12.50014368878688
Au	12.49995260460963	12.50011199464116	12.49987181651705

Au 14.52898469223339 14.52900531010357 12.49990066865173
Au 12.49988019798583 8.45918647118728 12.50010379550722
Au 4.46036815969359 16.56093736910648 12.49980797228876
Au 14.52904217407842 10.47108096933280 12.50006754484820
Au 14.55950581400592 10.44039631728315 8.41959515908601
Au 14.53225271613456 6.45601858438533 12.50018769751482
Au 12.50008060234078 12.49988688114994 8.44053621135652
Au 10.46752309809633 18.54401576178790 12.49973333725834
Au 12.49987721722629 8.46139057719046 16.54867861945902
Au 12.50018720213000 16.49890341836860 4.51177408477460
Au 12.49993919690607 20.48952386599428 16.49960190521705
Au 16.54089343884507 12.50003684929337 12.50009216924924
Au 8.50093970943171 12.50019308227966 20.48823173537290
Au 16.53865408898741 12.49988934529891 8.45149425961075
Au 12.49983597335113 16.53879330731661 16.54844429948175
Au 16.53852719090795 12.50020099814988 16.54867750603235
Au 10.47093034644541 14.52901711991699 12.49982365317185
Au 8.46138511994164 12.49998184920739 8.45128340025949
Au 10.44054762361995 10.44058486817436 16.58061884348733
Au 10.44058750164629 14.55947073847685 8.41923040397308
Au 6.45609705123963 14.53248206872909 12.49983924298121
Au 4.51072840602621 12.49994234615544 8.49994714913607
Au 12.50002413349103 16.53862891167971 8.45120153960469
Au 14.55937667047458 14.55960562563723 16.58048757912319
Au 16.56081413690783 4.46020047985688 12.50019826794580
Au 14.53235354184968 18.54389367236559 12.49978774825369
Au 14.55926313879251 10.44065024593363 16.58065703917458
Au 18.54393311360464 10.46767437908584 12.50025298517659
Au 16.56298864997555 8.43702276582625 12.50028904171778
Au 8.43903101886581 4.46001365483581 12.50005701446718
Au 10.44055026945517 10.44041062754739 8.41953377833356
Au 8.43691774387384 16.56306267882221 12.49974976973119
Au 8.43688698410471 8.43696337982360 12.50004689607945
Au 14.55951429100984 14.55940118356634 8.41937433484864
Au 20.53968915062453 8.43922768848592 12.50030829687714
Au 12.50001267727087 8.46128598222859 8.45163821431472
Au 8.50125994260113 12.49984252830109 4.51172424789992
Au 8.45916946655409 12.50007373455565 12.49990644806683
Au 4.46040599764893 8.43926535113609 12.50002925085282

$Pt_{40}Au_{161}$LEH-700.686
Pt 6.54283907533454 10.42361189467421 12.50001521009825
Pt 12.50219711328981 16.49067825723272 12.49993487533625
Pt 10.56794784491542 10.44243058791301 12.50004749145669
Pt 12.49170818120028 12.43625063619136 16.44846566149945
Pt 10.53472030808000 6.55815605548826 12.50008047386905
Pt 12.50539092483224 12.47104344454052 12.50002686278706
Pt 14.47350358648242 14.49305431226503 12.50004959130704
Pt 12.49361529838023 8.44231354939683 12.50005510503728
Pt 14.43047963528527 10.43259907412134 12.50011364730852
Pt 14.47736612895934 10.41989086642603 8.55020992960762
Pt 14.44685426032560 6.55319157146728 12.50011660204705
Pt 12.49173970789467 12.43601898438501 8.55162554755527
Pt 12.49024191029331 8.47950294152612 16.48751177563908
Pt 16.41009812130368 12.41245318264454 12.50007601836232
Pt 10.52908739394036 14.48038275894902 12.50000829893310
Pt 10.51387432058378 10.42728639418813 16.43674874321730
Pt 14.47734429841930 10.42006364064127 16.45000202559280
Pt 16.43052085332539 8.49029620278484 12.50005993885380
Pt 10.51391784306635 10.42721699132417 8.56339925285778
Pt 8.54286655102649 8.49351354283254 12.50002585693354
Pt 12.49029237777528 8.47941627886063 8.51270344765311

Pt	8.56716471496177	12.41260890537807	12.50001210388891
Pt	14.48878307369719	12.50201941030147	14.50887299292077
Pt	8.57146651987650	10.42611497337083	14.48892394629139
Pt	12.49717970080532	10.44566986603164	14.46183052216811
Pt	12.48613442371107	6.53718687610621	10.50567913454151
Pt	12.50288397653992	14.49831374439592	14.47988948082434
Pt	10.50667816985115	12.51191663117662	14.50739031171021
Pt	14.53488520267208	8.44537903473884	14.57022317586106
Pt	14.53497257352861	8.44532630941675	10.43000270863187
Pt	10.45163529671855	8.44773894165383	10.43339786781215
Pt	12.50287485401855	14.49815294518577	10.52009425337531
Pt	16.40996982528461	10.41615944002125	14.49576418484941
Pt	14.48884971215317	12.50182220334564	10.49127991883344
Pt	10.45169221830372	8.44784427068019	14.56673526784690
Pt	12.49721137401942	10.44556838604870	10.53833134391446
Pt	12.48610346262201	6.53722923685821	14.49449934397592
Pt	8.57149115322672	10.42605477299390	10.51111930477865
Pt	16.41000951411407	10.41610636859481	10.50437416423905
Pt	10.50668194084815	12.51182811778393	10.49264868945762
Au	14.50892313507901	14.48923424774396	4.43003676938874
Au	12.50085414866480	6.53732540011326	18.58477523802877
Au	8.27784084430680	10.27733526193867	18.78683829389945
Au	12.50315690526801	4.58872035444658	16.50668941405032
Au	20.47201489221151	12.48305622325277	16.52209571014716
Au	10.48101743551493	14.48913836563923	20.57235009992247
Au	10.24063165625278	18.76481725422099	16.76109877952465
Au	20.49808729580807	14.45758436784040	14.52367326648487
Au	20.64478784398632	10.43381072165123	14.58258878547526
Au	10.48124831386266	14.48841394840982	4.42730394801641
Au	10.35372086829730	8.39368293690683	18.68889424390801
Au	4.48154431665996	14.46627939761857	14.52844192082497
Au	14.61199411243269	6.45588450180360	16.64624000213140
Au	6.25924185740662	10.26902198758281	16.79236554121075
Au	12.50087855856833	6.53738600042398	6.41533912588169
Au	14.70552049256431	16.70742734022587	18.69943699594481
Au	14.50337653351871	4.50956525808026	10.47016079250594
Au	6.41182376699170	8.43152342868009	14.64116322437692
Au	14.61206297425347	6.45592965718259	8.35385454246553
Au	10.45729544431459	20.59716163039037	10.44857856717225
Au	8.38854699708532	6.43698575755352	10.36361250793844
Au	10.28957080914580	16.70112625881973	6.30368271460498
Au	18.66596298470178	8.39650755332710	14.67582568340158
Au	14.52606401563218	20.59965450468478	14.54482545974944
Au	6.35371787194833	14.66181572509489	8.31338138575493
Au	6.49206099377665	6.53124999531201	12.50000278013563
Au	8.26112557192173	18.76170106266917	14.76352914886276
Au	18.52544040635298	6.57351661698883	12.50016145312845
Au	6.47637620695519	12.49142766487209	6.41658179709616
Au	18.66216733894329	16.70423538541523	14.73547649089836
Au	16.64213262661303	8.43483071816168	16.65785387445456
Au	6.25918213461617	10.26901883989119	8.20766678539151
Au	6.34076434942673	16.69500641764661	10.28156722375478
Au	16.72891149249316	16.75701488554482	16.76210698425310
Au	14.74893277224548	18.76484843385511	16.75831797106082
Au	16.62033397201638	6.46099706562209	10.37604018898691
Au	12.49513648213436	4.42857626831521	12.50009359668863
Au	10.45721131157449	20.59733016393404	14.55101949906890
Au	14.50345402721411	4.50954304304770	14.52999349366097
Au	12.49614966029947	12.42780619327153	4.25167234365234
Au	8.26107490088918	18.76161221056322	10.23610043209735
Au	10.38744416101795	6.44470575414706	8.34800153903694
Au	12.49238389599608	20.78055084757806	12.49983468975323

Au	18.66601022475572	8.39635251429579	10.32440208405199
Au	10.35363286806052	8.39354033254690	6.31117091654938
Au	16.62031655521220	6.46105851462597	14.62408621490908
Au	14.54970841164670	10.43597729579535	4.31601896551862
Au	14.64241749137135	8.39995832130354	6.32013349554894
Au	18.48548121919847	18.53579353878004	12.49995768294916
Au	6.34084705745693	16.69506586444790	14.71819755864862
Au	16.72078794548618	18.75315323915856	14.75618479865398
Au	18.65297062452081	14.67766336068084	8.30325375079814
Au	10.44755869564836	10.43306828258743	20.68682849647213
Au	6.41177506275732	8.43153172624752	10.35884051482000
Au	8.36737511140427	8.43078607520022	16.67231084728539
Au	18.66226800639681	16.70414167774597	10.26451428464084
Au	18.52740924307515	12.49501871104519	18.57103448654563
Au	14.50883651108028	14.48978617105513	20.56994760619152
Au	8.27779471860795	10.27714148470074	6.21308643995861
Au	14.54935379363848	10.43631827007412	20.68427009308425
Au	4.48144582220660	14.46613848617279	10.47172321202387
Au	16.72074755373927	18.75302345198923	10.24372807026045
Au	4.42311021941540	10.44476929632203	10.42755465796811
Au	12.49725845755482	18.54608465730093	18.53331307336737
Au	16.70464736621452	10.29482780896430	6.24644760631061
Au	8.32691237668965	14.67469970759861	18.69884090320755
Au	10.28929547328539	16.70171374658135	18.69602563424176
Au	20.64487157662576	10.43374028043411	10.41764617395159
Au	4.36090601798731	12.38894670461212	12.50002908746829
Au	14.74897146621867	18.76452477497046	8.24134557621275
Au	16.66650490165263	14.67958539434581	6.30746518684801
Au	6.50250320990068	18.53301489346551	12.49990621062649
Au	6.47633811849784	12.49159600529625	18.58344663141406
Au	18.75191906878924	10.27168860958757	16.77076541407584
Au	8.38858967933951	6.43702676188888	14.63636052983631
Au	4.42309561136732	10.44470539777831	14.57254866543621
Au	8.36731841663025	8.43076861588549	8.32772773748735
Au	10.49812061438680	4.48791757823271	10.46495973345010
Au	20.70032215153448	12.39310347214166	12.50011931793117
Au	16.72873308900558	16.75664262143054	8.23802312079019
Au	20.49819763939431	14.45745124815059	10.47643406398962
Au	16.64226454352741	8.43481663819983	8.34235852985003
Au	18.52762489152547	12.49481237016395	6.42930197559706
Au	18.75212620444568	10.27156875128909	8.22946449874974
Au	10.49805568306344	4.48786301318843	14.53507684837239
Au	18.65311703272791	14.67797172626764	16.69700169965999
Au	6.35358511696756	14.66202834066880	16.68662129789870
Au	8.27784207059739	16.74064887065291	8.25478739961893
Au	16.70439439577881	10.29499103602126	18.75374363238613
Au	14.52600598245947	20.59947320939361	10.45477330265185
Au	8.32703807519329	14.67450407321738	6.30101739819118
Au	14.70540597097229	16.70685822700971	6.30044146654452
Au	14.64219837445078	8.40009674698636	18.67994644456068
Au	10.38744300533418	6.44469510436509	16.65202225427165
Au	12.49597895538994	12.42873215144727	20.74849335155518
Au	16.66653690198422	14.67998185448197	18.69267497819368
Au	10.44731462676495	10.43265614472660	4.31328627330391
Au	10.24061247247301	18.76452261379899	8.23845685995126
Au	8.27770363621494	16.74092429188757	16.74501010907279
Au	12.49731663569098	18.54557452687666	6.46615633127603
Au	16.46930116806808	12.49147496979721	20.51196731217727
Au	12.50027888782073	8.51162135657591	4.38289207946187
Au	16.46965629506428	12.49116317051404	4.48818645211407
Au	12.50013699715238	8.51160709846636	20.61718679742323
Au	20.34950460696534	16.42231108970736	12.50001665433241

Au	12.49203310873848	20.47507028864059	8.51416819696810
Au	18.47646796465736	14.36744482688895	12.50000569973722
Au	12.49612539382057	16.45731354959352	20.43725655249460
Au	12.50318314673210	4.58877075810197	8.49339964009164
Au	16.54952491279903	16.51033576753470	12.49997328684817
Au	10.44084922503645	14.53011471100524	16.57489087640388
Au	8.46775188272626	12.38763849408680	16.55508425049268
Au	16.45679775050681	20.49752769883075	12.49992951329577
Au	20.47225041380932	12.48299444568549	8.47813557862451
Au	4.53263886538938	12.48304736409892	16.53063096264567
Au	8.54039650010663	20.47979319212722	12.49979215638123
Au	4.60671970047936	16.43262883842337	12.49996456086248
Au	10.49541983607789	18.53073607824647	12.49987981055055
Au	12.49618214348883	16.45662337801746	4.56228401426816
Au	12.49203878380766	20.47551594920560	16.48512991615070
Au	8.52493269133928	12.48568627125194	20.53107827707655
Au	16.52478938824164	12.38496011958190	8.44229798922546
Au	12.49946044622635	16.53762094551554	16.55855643690646
Au	16.52474553920754	12.38527723260521	16.55774232597088
Au	8.46782623862598	12.38737969407846	8.44486396936369
Au	10.44089723824568	14.52989273776405	8.42498833779823
Au	6.51727828629815	14.41951483388993	12.49993512315156
Au	4.53253735815458	12.48317126591335	8.46938211519507
Au	12.49951251056029	16.53744284418896	8.44133611995334
Au	14.55372263492701	14.52813370725695	16.57393355675558
Au	16.44110149684758	4.61281004620723	12.50004977647379
Au	14.51942445737070	18.52871085300381	12.49989696538530
Au	18.48869299471146	10.39499585855159	12.50005780272514
Au	8.57125031949978	4.59215632188901	12.49993981442331
Au	8.46534463797755	16.52681317900957	12.49988592715159
Au	14.55366557882668	14.52772849939963	8.42620316793203
Au	20.57130242387527	8.54270076114157	12.50009986144053
Au	8.52490821664838	12.48562959445654	4.46895600013700
Au	4.50445135533498	8.55875719558545	12.50005769873678
Au	16.55120274101960	14.51330736790222	10.42838997643040
Au	18.48033849523311	12.36044300102232	10.45744437540056
Au	6.52025873264644	12.38388770577379	10.46852597440299
Au	12.49376357256039	14.42710644842753	18.52318303400577
Au	14.49992099403059	12.39220361192536	6.48051106865388
Au	14.49974627709627	12.39255115287512	18.51960257283314
Au	10.43198762148203	16.56018632360213	10.42390368221886
Au	18.48027546332593	12.36049057368273	14.54260621215797
Au	10.43191210296514	16.56031958769606	14.57595299088432
Au	10.48059368761337	12.38736161326556	18.52216033070373
Au	14.57334605664063	16.55922944170718	10.42214168494453
Au	6.52025605980695	12.38393732049962	14.53135206542802
Au	12.50155087049956	18.52968188278829	10.48593771251265
Au	12.49383001462839	14.42662671246087	6.47676334052213
Au	16.55124336951737	14.51347907272760	14.57171143077607
Au	12.50155931247714	18.52970949382352	14.51384685220414
Au	12.49390551114462	10.40545474653857	18.53539041375247
Au	8.45094815294079	14.52222041255528	14.56522541534045
Au	12.49391896980478	10.40521277734826	6.46475493520103
Au	10.48060129307642	12.38704814771964	6.47780159881855
Au	8.45101534369028	14.52208954039539	10.43468320632704
Au	14.57344219469034	16.55942224193775	14.57782367746003

$Pt_{105}Au_{300}L1_0$-1497.798

Pt	12.02487744335333	12.02484430331275	5.88255213264884
Pt	14.00001616185089	10.02233786429816	5.92327485538384
Pt	15.97516889396578	12.02486810929823	5.88253547528681
Pt	10.02233668463991	14.00000710365287	5.92328886589284

Pt	12.02487536982678	15.97516766082664	5.88254567763426
Pt	14.00005203408579	14.00000463275811	5.82302297368641
Pt	15.97517246363317	15.97513269566443	5.88253372093980
Pt	17.97762852135811	14.00000398107241	5.92326850352120
Pt	14.00002221858010	17.97765477321660	5.92325856985092
Pt	11.93910505241722	7.93710096301761	9.89724476001881
Pt	14.00002153398079	5.96094265614324	9.94018214413249
Pt	16.06096258355926	7.93711730299327	9.89723228470753
Pt	7.93708546280957	11.93906686917040	9.89725946931791
Pt	9.92902548323006	9.92899459372063	9.89225527325625
Pt	11.97911005495005	11.97905566013536	9.93048574569125
Pt	14.00006114559917	9.95571642893335	9.93419545786235
Pt	16.02098348756867	11.97907716175222	9.93045725319299
Pt	18.07099749696090	9.92903428630165	9.89224718675562
Pt	20.06285061849308	11.93908083981970	9.89724585042568
Pt	5.96089491524807	14.00000474842123	9.94018233168165
Pt	7.93707898236663	16.06095010651338	9.89725607281247
Pt	9.95572434576812	14.00000425875794	9.93420795285931
Pt	11.97910857770170	16.02095469946699	9.93049127272984
Pt	14.00007998489667	13.99999868208064	9.94253304389974
Pt	16.02097492256479	16.02091222965384	9.93046826648722
Pt	18.04429860967502	13.99999477276653	9.93420626957661
Pt	20.06285519852225	16.06091436936108	9.89725016946097
Pt	22.03896040472631	13.99999969269028	9.94019176762850
Pt	9.92900754484363	18.07102658038659	9.89225739504720
Pt	11.93909491458561	20.06291829359071	9.89724161297341
Pt	14.00005178813942	18.04429297777207	9.93419364059159
Pt	16.06096321809312	20.06288496866954	9.89723309577743
Pt	18.07101081993338	18.07096764871659	9.89226177224348
Pt	14.00002002103677	22.03905777577845	9.94017380839889
Pt	7.95752068860456	7.95751517110536	14.00001397915359
Pt	9.97869922778091	5.98033075527493	14.00001900910302
Pt	11.97060527303416	7.94605440625399	14.00001107844031
Pt	14.00005886016259	5.92023047850183	14.00000108564411
Pt	16.02950080797725	7.94606690254256	14.00001439424810
Pt	18.02134352078426	5.98038282536785	14.00001909438929
Pt	20.04243937990767	7.95758475915673	14.00001277422652
Pt	5.98032538632644	9.97869309875862	14.00002011682834
Pt	7.94604818942264	11.97055894423274	14.00001300338358
Pt	9.93019892566996	9.93019586095534	13.99999375984949
Pt	11.94499545313031	11.94497749101942	14.00002087366668
Pt	14.00006229405281	9.92242474021118	14.00006284136338
Pt	16.05508500283450	11.94494016678601	14.00002716879228
Pt	18.06985463387333	9.93016181444839	13.99999062251692
Pt	20.05388337586226	11.97056624197542	14.00001536606118
Pt	22.01953456480832	9.97870413292605	14.00001680314569
Pt	5.92015345781752	14.00000721301822	13.99999973894580
Pt	7.94602287394641	16.02946961720748	14.00001578305570
Pt	9.92245410504007	14.00001449043082	14.00006544962330
Pt	11.94499313021495	16.05503655004202	14.00002817465898
Pt	14.00001295046056	14.00000875114821	14.00000646997129
Pt	16.05508358483055	16.05506770972249	14.00002434151304
Pt	18.07761261808685	14.00000580486304	14.00005876681865
Pt	20.05390660053211	16.02943919066466	14.00001139997824
Pt	22.07961913240761	13.99999456597338	14.00000289554973
Pt	5.98029668133071	18.02134431437393	14.00001729625120
Pt	7.95749213031779	20.04251530981371	14.00001032676507
Pt	9.93018300475770	18.06982637282257	13.99999560370535
Pt	11.97059639905171	20.05395486058888	14.00001212677246
Pt	14.00005397172819	18.07757668442898	14.00006190436948
Pt	16.02950151413031	20.05392511809612	14.00001131092558
Pt	18.06985980358999	18.06983740522048	13.99999058846295

Pt	20.04244718898915	20.04242868480152	14.00001142298320
Pt	22.01955186244298	18.02131223212084	14.00001821578553
Pt	9.97868825853303	22.01967705584670	14.00001398705901
Pt	14.00005799420640	22.07976737896679	13.99999829802761
Pt	18.02133360438783	22.01960662390403	14.00001602196056
Pt	11.93909197174284	7.93711913921798	18.10277376074009
Pt	14.00002588306646	5.96093338222847	18.05984953059875
Pt	16.06098153856269	7.93713087293415	18.10277817878909
Pt	7.93710879652631	11.93904800524327	18.10276333315441
Pt	9.92905148027490	9.92902213207215	18.10783153449657
Pt	11.97917455147444	11.97911964917116	18.06966127866467
Pt	14.00006261748164	9.95575122591832	18.06578443584727
Pt	16.02091923116830	11.97914644054666	18.06968413725085
Pt	18.07097302262286	9.92905839104826	18.10782855172056
Pt	20.06282311950925	11.93906477870693	18.10276742492089
Pt	5.96089505412297	14.00000315166588	18.05985354530232
Pt	7.93709593111237	16.06096125817597	18.10276306833609
Pt	9.95575582861086	14.00000579955973	18.06578367793728
Pt	11.97916462695833	16.02089258918931	18.06966672766070
Pt	14.00007709352714	14.00000345701139	18.05780837735687
Pt	16.02092596219978	16.02086298954980	18.06968843791185
Pt	18.04426477898943	14.00000323781791	18.06577633207603
Pt	20.06282950927964	16.06093104734721	18.10276870616808
Pt	22.03895172355577	13.99999816340514	18.05984577309605
Pt	9.92903208654590	18.07099179754298	18.10783474713074
Pt	11.93907860641611	20.06289254102801	18.10277786770635
Pt	14.00005917750050	18.04426014070815	18.06579400732485
Pt	16.06098476255343	20.06286510022884	18.10278907841331
Pt	18.07098056105009	18.07093883069365	18.10783292421671
Pt	14.00002170840758	22.03905773184404	18.05985708290957
Pt	12.02494795422333	12.02487900092220	22.11747958283258
Pt	14.00002264854513	10.02237597196261	22.07675598725648
Pt	15.97512268254743	12.02491757843602	22.11748778431699
Pt	12.02494438897783	15.97512172976626	22.11748432136171
Pt	14.00006080910136	14.00000300906357	22.17693837146457
Pt	15.97512471151713	15.97508175926854	22.11749800082470
Pt	17.97760038626392	13.99999893915971	22.07676054082660
Pt	14.00002082974015	17.97762337642840	22.07677300304048
Pt	10.02237184772378	13.99999982022135	22.07677563811670
Au	14.00001182507128	5.90378424594464	5.88905284426347
Au	13.99996120093955	8.02268058070472	4.01376092180176
Au	16.17413071250404	7.80707075727617	5.79018294146333
Au	7.80708134522139	11.82587702304064	5.79019076247124
Au	9.80651405138207	9.80649773374532	5.77455342696235
Au	9.98464441697017	11.97689904459463	3.92587685092843
Au	11.97690975227074	9.98462676518849	3.92586666615202
Au	14.00003003733501	11.99241485783627	3.75704342492150
Au	16.02312128997783	9.98461465048890	3.92586560720454
Au	18.19350133771731	9.80650004440779	5.77454315278891
Au	18.01537976876796	11.97688794336728	3.92586502614624
Au	20.19289136482927	11.82587472450424	5.79017793154923
Au	5.90381407106904	14.00000901362700	5.88905437408190
Au	8.02267021159401	14.00000108060903	4.01368908983922
Au	7.80710558741182	16.17407851322659	5.79023483752142
Au	9.98465882083381	16.02311502032753	3.92584946531771
Au	11.99244579627943	13.99999872720925	3.75703909992003
Au	14.00002466470134	16.00758692080312	3.75702772291354
Au	16.00760820197949	13.99999639792218	3.75703642411159
Au	18.01537193941930	16.02310868849748	3.92583908240482
Au	19.97723246527220	13.99999549946569	4.01382811544719
Au	20.19289056403312	16.17406737621957	5.79021912378851
Au	22.09614517548717	14.00000398605097	5.88907401668324

Au	9.80651756685441	18.19351121679360	5.77454580064144
Au	11.97691443926484	18.01538083192653	3.92583523404328
Au	11.82589839247739	20.19294012659742	5.79015696422858
Au	13.99996647429547	19.97729814621980	4.01372844291876
Au	16.02312762157681	18.01536571269227	3.92582707598818
Au	16.17408336946607	20.19291502371385	5.79020227600480
Au	18.19350335163804	18.19349131779837	5.77454664704479
Au	14.00002547834026	22.09619975710024	5.88902768853621
Au	11.98655637263222	3.88596207587495	9.95683225675191
Au	14.00001681298690	3.99748777399259	7.98105841898086
Au	16.01348426847399	3.88595226027400	9.95682235667444
Au	7.79598043170646	7.79596723271538	9.79030628839365
Au	9.82314266424417	5.79563763555146	9.80079535422932
Au	9.83967820705536	7.82981873044009	7.82533383525324
Au	11.87212200425815	5.85929155295998	7.85433784016965
Au	14.00002423013277	7.93494332247709	7.90996374306563
Au	16.12791451587711	5.85927989065345	7.85432693206573
Au	18.17687612993168	5.79563361317695	9.80077809081815
Au	18.16033858313638	7.82980335637477	7.82532832181631
Au	20.20400404683322	7.79597031777244	9.79027132426627
Au	3.88596453204993	11.98653536972768	9.95679970991926
Au	5.79564481732872	9.82311426863543	9.80078323857762
Au	5.85929269566250	11.87209560633394	7.85431491632444
Au	7.82982083489692	9.83966995612832	7.82532306076448
Au	9.92575205504877	11.92747112083870	7.87961485524168
Au	11.92749842157433	9.92572921701426	7.87961773725543
Au	14.00004609890179	11.97424919160241	7.93398058941248
Au	16.07255107857956	9.92572534293018	7.87960645248393
Au	18.07428391135735	11.92746592533801	7.87962083384979
Au	20.17016349794523	9.83966228115912	7.82533852874448
Au	22.20429573348516	9.82311846561301	9.80076584906132
Au	22.14064552412651	11.87210208380673	7.85433985489633
Au	24.11392871592929	11.98653064704378	9.95679919292549
Au	3.99751046764075	14.00000283017775	7.98103649673669
Au	3.88595888924279	16.01347695858231	9.95681000688310
Au	5.85929298968624	16.12791260385856	7.85432434299158
Au	7.93495564209842	13.99999700537093	7.90997909421778
Au	9.92576345177912	16.07253469261522	7.87963019263010
Au	11.97427969856185	13.99999884570932	7.93398007864656
Au	14.00004358500000	16.02575279344394	7.93396655825639
Au	16.02576848806711	13.99999853874603	7.93398676683126
Au	18.07427582307665	16.07252160030600	7.87963775497217
Au	20.06503351848524	13.99998853743269	7.90999656797973
Au	22.14064847326766	16.12789475893844	7.85435793883000
Au	24.00240193571420	13.99999985306394	7.98106570698394
Au	24.11393462346442	16.01347108775921	9.95682162262088
Au	5.79561394633708	18.17689158656606	9.80078097158954
Au	7.82981623397100	18.16034183493728	7.82531339978973
Au	7.79595557063787	20.20404859874134	9.79029200642027
Au	9.83966866566258	20.17019984376951	7.82531316417719
Au	11.92749574889492	18.07428724151839	7.87960929373356
Au	14.00001643053140	20.06505196181517	7.90996520650920
Au	16.07254745112903	18.07426278798324	7.87961634268010
Au	18.16035152467911	20.17018660251356	7.82532510392133
Au	20.17017430248778	18.16033526964745	7.82534409211651
Au	20.20403119178460	20.20402688626315	9.79028115289006
Au	22.20432199104364	18.17686633095418	9.80078197289248
Au	9.82312639962966	22.20437110605508	9.80077628876644
Au	11.87211463497298	22.14070838514410	7.85431113412902
Au	11.98654840858635	24.11402222138730	9.95680919991807
Au	14.00002453080360	24.00248374588998	7.98102446403211
Au	16.12793224590815	22.14069990590195	7.85431533832268

Au	16.01349652061773	24.11402534682573	9.95679682115800
Au	18.17689047585333	22.20435943306787	9.80077197402502
Au	8.04884985161508	3.99939891657279	14.00000329525389
Au	9.96645446682560	3.91632322223123	11.95458771436260
Au	11.99113461089523	3.80655429912302	14.00001514868837
Au	14.00000031894963	3.81550188076982	12.00087883206978
Au	16.00893073873858	3.80657480280128	14.00001253221292
Au	18.03358029467573	3.91632559087996	11.95458124422864
Au	19.95115492771932	3.99940013471640	13.99999894189453
Au	3.99939560307918	8.04887814204820	14.00000803825849
Au	5.95873530346366	5.95876072548680	14.00000299534625
Au	5.84795649509269	7.84964128529348	11.85836570235854
Au	7.84965319695354	5.84797214822510	11.85837123910266
Au	9.92327480909903	7.90709339619487	11.91393824812747
Au	11.98543582257579	5.91865390574206	11.96590904984120
Au	14.00004913312911	7.94249942587191	11.94912951492593
Au	16.01463040590263	5.91865258986998	11.96592028833643
Au	18.07677048439323	7.90710402879859	11.91395495004211
Au	20.15032901907083	5.84796985100583	11.85838760856023
Au	22.04122291593412	5.95877765194541	13.99999569383452
Au	22.15197996953576	7.84967401153127	11.85838224011112
Au	24.00050245765855	8.04890761870085	13.99999605589311
Au	3.91633412546118	9.96643488111189	11.95455945468552
Au	3.80649694912266	11.99109224578268	14.00001067758629
Au	5.91862710196013	11.98540105258947	11.96592229743785
Au	7.90709792739438	9.92324447677527	11.91393514734329
Au	9.93897133720909	11.95943256699627	11.95035632266239
Au	11.95947294509059	9.93896094880659	11.95033649282784
Au	14.00004545597674	11.96596290839488	11.99061453839790
Au	16.04062507577017	9.93891723142884	11.95033940723341
Au	18.06109221862688	11.95941898499299	11.95032596048144
Au	20.09287400302041	9.92325148471603	11.91394303422875
Au	22.08122915036298	11.98540143703389	11.96591020675492
Au	24.08355906472253	9.96645444364581	11.95456599455952
Au	24.19321314652988	11.99109205008744	14.00001607358999
Au	3.81544104849979	14.00000503493663	12.00087250938028
Au	3.80648319419709	16.00890931968294	14.00001188190491
Au	5.91860951780537	16.01461819207242	11.96592399845240
Au	7.94249861859695	14.00001285009827	11.94913395564014
Au	9.93896143866018	16.04058568026499	11.95035467555040
Au	11.96600183800454	14.00000975642001	11.99063794641623
Au	14.00003887485522	16.03404863619408	11.99061529375795
Au	16.03407266306317	14.00000160240065	11.99058763391666
Au	18.06108518497639	16.04057801148601	11.95034100815542
Au	20.05746646459870	14.00000596759628	11.94913397662688
Au	22.08124497122104	16.01459870115452	11.96591593101668
Au	24.18428850884512	13.99999084844437	12.00086210871626
Au	24.19322763933978	16.00887984703422	14.00001677310754
Au	3.91630658636654	18.03358566267905	11.95457289895399
Au	3.99936622773171	19.95117343981472	14.00000068781184
Au	5.84793606362439	20.15038498610790	11.85834727918231
Au	7.90707700563981	18.07677556646798	11.91393673414362
Au	9.92325527103609	20.09292335609106	11.91393294008412
Au	11.95945735882368	18.06106728502292	11.95032517582427
Au	14.00004797464800	20.05749896271536	11.94912411321457
Au	16.04062162459395	18.06108758387739	11.95033860054795
Au	18.07677638823544	20.09288847637502	11.91395184465463
Au	20.09288684659800	18.07674260900338	11.91395275604186
Au	22.15199062433688	20.15032927088119	11.85838593740531
Au	24.08358059953866	18.03355233848724	11.95459537079650
Au	24.00052114580954	19.95112810149761	14.00000354389104
Au	5.95871847092921	22.04127277237341	13.99999671592400

Au	7.84962932170717	22.15204184894986	11.85834958751447
Au	8.04883700083009	24.00060376002187	13.99999936561689
Au	9.96644522057413	24.08366737330458	11.95457926509580
Au	11.98543196990712	22.08134734578342	11.96590265820815
Au	11.99114209638666	24.19342657874827	14.00000811018914
Au	14.00000464179551	24.18447257813897	12.00085391922044
Au	16.01463775574656	22.08133282022618	11.96590565519208
Au	16.00893108867910	24.19340212020066	14.00001147613991
Au	18.03359207170588	24.08365174979828	11.95458062060104
Au	20.15034136677940	22.15201811519348	11.85838256004501
Au	19.95115677305038	24.00058102471411	14.00000287864061
Au	22.04123281506885	22.04123747047955	14.00000208758948
Au	9.96644389865113	3.91631241213936	16.04542651231459
Au	11.98654896790292	3.88594250195367	18.04318501856275
Au	14.00000072219724	3.81549049522861	15.99915115514594
Au	16.01349135674937	3.88592658490972	18.04319130489307
Au	18.03359237996924	3.91630968931390	16.04542257585838
Au	5.84793971993059	7.84963387001892	16.14165310741872
Au	7.84964675907597	5.84795085246048	16.14164374935762
Au	7.79597395542353	7.79595438550443	18.20972415204593
Au	9.82313604329403	5.79561874984328	18.19921265009489
Au	9.92327174933000	7.90705901019789	16.08606250270415
Au	11.98540517674441	5.91863849179889	16.03409941252897
Au	14.00005087429472	7.94256842959038	16.05085733158426
Au	16.01466542000893	5.91863643243592	16.03408813849801
Au	18.17688743278931	5.79560351841340	18.19922253787231
Au	18.07677370606201	7.90706562944860	16.08604656542295
Au	20.15033993677991	5.84794604278489	16.14162061453917
Au	20.20401359559590	7.79595563648568	18.20974062594745
Au	22.15199808399037	7.84966145757486	16.14161783553215
Au	3.91632400064848	9.96643008856305	16.04545529099041
Au	3.88594798629534	11.98653192218502	18.04321885056019
Au	5.79562302511119	9.82310094713877	18.19922924056619
Au	5.91861347546970	11.98537047250790	16.03408386484968
Au	7.90706480388114	9.92324142768703	16.08606591466140
Au	9.93894470353419	11.95948270625217	16.04967997791050
Au	11.95952531151853	9.93893402678707	16.04969360074933
Au	14.00004232931225	11.96589757271239	16.00941108269618
Au	16.04057142568330	9.93889949477694	16.04967571436238
Au	18.06110885595959	11.95947228986982	16.04968656844713
Au	20.09290628963728	9.92324602249046	16.08605862105881
Au	22.20431981934233	9.82312337469779	18.19916254945705
Au	22.08123702430549	11.98536399882013	16.03410087287490
Au	24.08356392406210	9.96644020213895	16.04543562962538
Au	24.11393418365030	11.98651691275829	18.04321483570978
Au	3.81542936206880	14.00000321172963	15.99915372183291
Au	3.88594264647661	16.01348141773267	18.04320254790413
Au	5.91859535736746	16.01464780135159	16.03408419687249
Au	7.94257109185197	14.00001207151152	16.05085664054073
Au	9.93893087597835	16.04053348427779	16.04968150808488
Au	11.96593285393595	14.00000846169536	16.00939659273416
Au	14.00003994975497	16.03411782797289	16.00941773800833
Au	16.03413886270804	14.00000648466181	16.00943523436639
Au	18.06111173104415	16.04052965053230	16.04968378873149
Au	20.05738926177610	14.00000282943315	16.05085334032091
Au	22.08125169286011	16.01463147576597	16.03409530624819
Au	24.18429030344737	13.99999028171433	15.99917583394164
Au	24.11393916256881	16.01347207507810	18.04319687288985
Au	3.91629910376120	18.03359500468126	16.04543489743332
Au	5.79559630023216	18.17690084363600	18.19922741474032
Au	5.84791729634626	20.15039312991603	16.14166013417633
Au	7.90704073257773	18.07677643227936	16.08606300050707

Au	7.79594679531241	20.20405039936347	18.20973356335088
Au	9.92325135220721	20.09295525365872	16.08606724056663
Au	11.95951049045971	18.06108182046408	16.04969942183477
Au	14.00004824984458	20.05743003729505	16.05086310672239
Au	16.04057198925766	18.06110004858800	16.04968018099026
Au	18.07678116376355	20.09292124719595	16.08604962097193
Au	20.09291486855690	18.07674714206056	16.08604731664822
Au	20.20402045428931	20.20402012306071	18.20975323230268
Au	22.20432600448880	18.17687359766552	18.19923028344196
Au	22.15199767150010	20.15033096714216	16.14162604180576
Au	24.08358123993194	18.03355770046072	16.04541695238763
Au	7.84962215404516	22.15205805252995	16.14165576901932
Au	9.82311141014050	22.20438997847038	18.19922738034612
Au	9.96643365861626	24.08367789714524	16.04542362990402
Au	11.98540007298984	22.08136160579528	16.03409904659778
Au	11.98653768707918	24.11404015372133	18.04320058768709
Au	14.00000099969101	24.18448497395711	15.99916910173586
Au	16.01467015308114	22.08134817565452	16.03409922764673
Au	16.01350118605512	24.11404306062081	18.04321296812454
Au	18.17687621037333	22.20438119482117	18.19917090715541
Au	18.03360159406032	24.08365854917552	16.04543016415639
Au	20.15034546002505	22.15203111262702	16.14163049377528
Au	14.00001797112673	3.99749794687462	20.01892287705388
Au	9.83969432053867	7.82982508153502	20.17468498104776
Au	11.87213765645805	5.85927420054693	20.14566541831191
Au	11.82590615917667	7.80707263625900	22.20978808252866
Au	14.00001444947284	5.90375234578086	22.11092046985040
Au	14.00003011226520	7.93491394843522	20.09000833938194
Au	16.12790170713483	5.85925653640393	20.14566858734708
Au	16.17411827286574	7.80706407050853	22.20978200523852
Au	18.16032080218429	7.82981203541723	20.17467085607990
Au	5.85926728090048	11.87210021864185	20.14568265095122
Au	7.82983070893301	9.83966802319670	20.17468350025798
Au	7.80708507199103	11.82587106087818	22.20978829185284
Au	9.80653385769793	9.80649684599308	22.22540916557744
Au	9.92575201061644	11.92741159983093	20.12036987150193
Au	11.92745473444926	9.92573132112634	20.12038026987079
Au	14.00005122072277	11.97434929663889	20.06595607914309
Au	16.07259829117023	9.92572551354194	20.12038708628017
Au	18.19348218170412	9.80650987364974	22.22541064574920
Au	18.07427996292138	11.92742401231046	20.12037285335023
Au	20.17014400148786	9.83967615173346	20.17465909796457
Au	20.19288015580129	11.82588311480283	22.20978597049725
Au	22.14064669784642	11.87211062815032	20.14565163478824
Au	3.99751511759608	14.00000044585823	20.01893700447311
Au	5.90378962983788	13.99999668880389	22.11092022764866
Au	5.85926036340451	16.12789202212253	20.14568075524474
Au	7.93492317020379	13.99999787591667	20.09001890239411
Au	7.80708532718230	16.17411590996499	22.20978650292696
Au	11.82588905269925	7.80707590773881	5.79018402599161
Au	9.92574527175302	16.07259191900524	20.12036986443008
Au	11.97441331599786	14.00000445052275	20.06591820106209
Au	14.00005003182827	16.02566155853239	20.06596869770312
Au	16.02568293928003	14.00000368445762	20.06594966425833
Au	18.07428710504332	16.07257705336810	20.12037999183934
Au	20.06505929399374	13.99999686729651	20.08998683448493
Au	20.19288817454812	16.17410483671602	22.20979691468990
Au	22.09615322671920	13.99999208384456	22.11090616728788
Au	22.14065226144790	16.12787054242084	20.14565400450375
Au	24.00237350084525	13.99999339219105	20.01891645030934
Au	7.82981686805334	18.16033322034791	20.17468926775411
Au	9.80652954999146	18.19349528150008	22.22541828238845

Au	9.83967971174273	20.17017500437002	20.17469551859408
Au	11.92744961621798	18.07427501642839	20.12038840786625
Au	11.82590264985918	20.19291568005782	22.20980422792588
Au	14.00002831127077	20.06508499267765	20.09001923620397
Au	16.07260096757339	18.07427594485278	20.12040358535663
Au	16.17412015864994	20.19291837703970	22.20980876208947
Au	18.19348902034009	18.19347935257603	22.22543187552844
Au	18.16033241913263	20.17017759564376	20.17469218521120
Au	20.17015264681994	18.16031143237346	20.17467242266866
Au	11.87212232358900	22.14071918291771	20.14568579119072
Au	14.00001088540895	22.09621981905843	22.11094752782659
Au	14.00001781700603	24.00247293944325	20.01894820602530
Au	16.12790934161789	22.14071959572511	20.14569379792739
Au	14.00001720136517	8.02281229871252	23.98599331558904
Au	9.98466474817179	11.97686120098780	24.07404388237730
Au	11.97688534188378	9.98462733932486	24.07406359780524
Au	14.00003637162376	11.99236680200805	24.24272021304226
Au	16.02316094430909	9.98462220597625	24.07405598123036
Au	18.01535744103620	11.97683897542163	24.07405629801703
Au	8.02278893243611	13.99999409359437	23.98607530018791
Au	9.98465793196931	16.02312957589126	24.07405348733255
Au	11.99240353081179	13.99999605030205	24.24272716977903
Au	14.00003390173370	16.00762732250903	24.24273078516299
Au	16.00765703848496	13.99999606977409	24.24272188086157
Au	18.01536380558945	16.02314757814742	24.07407132307503
Au	19.97714314562088	13.99991229222577	23.98600899831341
Au	11.97687554764406	18.01536175941410	24.07408454854631
Au	14.00001404616310	19.97716868082393	23.98602715498526
Au	16.02316564630244	18.01536247832730	24.07408280568816

$Pt_{105}Au_{300}$LEH-1510.764

Pt	11.97580198187142	7.98416540860943	9.98970895889611
Pt	14.00884300716893	6.05690559785026	10.03180345398100
Pt	14.01348893854707	8.05796578568803	8.06997508116693
Pt	16.04603163584222	7.99142397389769	9.99378242891908
Pt	8.04654688307723	11.95731468779016	10.01801247734842
Pt	10.00774165342127	9.98252789497142	9.99961319557077
Pt	10.04597035456301	11.94133993952042	8.03945830428173
Pt	11.99685885826370	9.97485970438230	8.04185445955009
Pt	12.01590957433076	11.99950329176988	10.01827021615848
Pt	14.00539024351215	10.00532598632950	10.03067209859681
Pt	14.01365977845987	11.97810252660940	8.03730150450438
Pt	16.03966713038587	9.98565360853664	8.05151198406078
Pt	16.00526170279004	11.98598834450327	10.01655367455951
Pt	18.02634477814984	9.97342123220269	9.98925706325644
Pt	18.00873434277384	11.94836993900891	8.04891580177369
Pt	20.01015962695092	11.95439905001996	9.99121300205629
Pt	10.02462719165514	13.99099830304074	10.01874762338065
Pt	12.02749241785028	13.97138365507643	8.03087913956928
Pt	12.00000015367985	15.98924555754955	10.00390077930319
Pt	14.00555795223309	13.99756910518669	9.98891167244944
Pt	14.01252729506052	15.95322339305223	8.02012110538755
Pt	16.00296945416631	13.96463860841640	8.03930862590290
Pt	16.00365478581248	15.98580561522297	10.01629534483792
Pt	17.98667081497569	13.97933513157133	10.03826964771598
Pt	18.00677319667201	15.96322260361150	8.03901317050187
Pt	19.95970531872866	13.94630938087255	8.05743914964591
Pt	19.98492799733894	15.97395728382747	9.99444495406827
Pt	21.97216984790590	13.96650115519938	10.00295658767721
Pt	13.99651609140554	17.97375176088810	10.00509950274176
Pt	18.00609428848432	17.96670316597201	10.00634876316690
Pt	10.02205623013278	8.01935158545827	11.95200699175517

Pt	12.01857984008221	6.02023597334241	11.97381568930190
Pt	12.02018916846163	8.00599801748074	13.97804087320140
Pt	14.00693469650631	5.98953857758183	13.93566297934101
Pt	14.01015652882475	8.01102477792940	11.97604630070621
Pt	15.99888350041241	6.02005716679210	11.97033106716917
Pt	15.99866446165637	8.01346656735226	13.97204397735919
Pt	18.00460398555211	8.02879241331805	11.94571682581214
Pt	8.04956645068582	10.00110439123336	11.96333750531537
Pt	8.03144645524930	11.98557002980506	13.98004964252979
Pt	10.03800619672089	10.00225124517217	13.99310480138471
Pt	10.03317291897288	12.00235376035258	12.01205393134764
Pt	12.02120425603884	9.99613449073609	11.99598073567500
Pt	12.03001098705820	12.00793928392465	13.97662663001189
Pt	14.00469208352260	9.97823046955019	13.97512538790369
Pt	13.99795513045692	11.98336748952781	11.98032568453770
Pt	15.99402046563484	10.00115904543070	11.99377773272228
Pt	15.97966748545915	11.99939547050633	13.97265499354333
Pt	17.98297853048995	10.01581792682894	13.98491869186351
Pt	17.98478537586377	11.99231742547037	11.99428362683294
Pt	19.98161896197248	10.00858174645423	11.94331502024159
Pt	19.98793512213565	11.99818441766371	13.97291010114433
Pt	22.00311960989195	12.00131545322135	11.96617339567235
Pt	8.02186377119985	13.97987679163886	11.99210811853083
Pt	8.01756674499591	15.96161852074202	13.97534814054681
Pt	9.99012389839114	13.99053106818015	13.97545853035130
Pt	10.00135245364717	15.99309349862714	11.99104558726895
Pt	12.02070853409381	13.99313856817042	11.99443128609727
Pt	12.02680395927107	15.97540875707250	13.97141958744923
Pt	14.01373971067269	13.98152315178461	13.95103023400882
Pt	14.00850412239554	15.98830627994960	11.98762406731879
Pt	15.98859923575626	13.96991838882886	11.98722202457338
Pt	15.99196950340292	15.96837343886895	13.96406894397406
Pt	18.00914802054303	13.98758190677334	13.96727110619311
Pt	18.00699963516621	15.98066528414761	12.00050528771808
Pt	20.00747255700240	13.96846817317600	11.98373156186182
Pt	19.98928523824689	15.95522582622054	13.97402378919653
Pt	22.04314175702816	13.95619211676899	13.96127084389707
Pt	10.01754080381592	17.97765055449226	13.98451080042295
Pt	12.01196958799532	17.99769491585866	11.99699016669853
Pt	12.02233037170401	19.98343687231400	13.98693573739204
Pt	14.01611204203218	18.01973457647567	13.96592731888325
Pt	14.01251700561471	19.99368249693314	11.99516176235687
Pt	16.00024532492260	18.00295510414281	11.98841727976778
Pt	15.99862474095800	19.98484127531736	13.97960366270534
Pt	17.99450381733044	17.96224768694341	13.98876226587926
Pt	14.01027489047493	8.01246747970069	15.96334554678963
Pt	10.02221476178610	11.99636101051289	15.97075631146631
Pt	12.01677479753299	10.00551875682927	15.97190553140271
Pt	12.02761707325752	12.00214602798130	17.97863859633383
Pt	14.01223694969305	10.00598428239300	17.96425056659226
Pt	14.00669073457766	12.00698292938404	15.96666163675462
Pt	16.00154922586962	10.00215998892368	15.96824571235507
Pt	15.99114530705031	11.99964007250472	17.97990240072439
Pt	17.99284066548806	12.00085502437941	15.96249724494094
Pt	8.02419763654414	13.97020971927631	15.95682232832295
Pt	10.02729051982195	13.98426838013614	17.96385457033261
Pt	10.02647897558741	15.96673635156040	15.96104740842366
Pt	12.03332305931695	13.99359620855270	15.95950325182763
Pt	12.03412009140906	15.96173800035740	17.96798447681789
Pt	14.00847013786410	13.98166536511596	18.02426802075546
Pt	14.01851541401363	15.96767154388363	15.95642908537100
Pt	15.98059926600520	13.98099870279083	15.95781383027845

Pt	15.98620968801369	15.96071981753332	17.97933519812860
Pt	17.98832963837571	13.97823599274344	17.96509988632084
Pt	17.97786009887476	15.96663681281159	15.97051774255941
Pt	19.98547044881241	13.97432949931098	15.95964679128446
Pt	12.03213203608372	17.96745696287292	15.95675299275257
Pt	14.00796339778832	17.97054177356517	17.96459645888556
Pt	14.01169403601314	19.97851068659283	15.96646520297014
Pt	15.98834232127836	17.97908412431494	15.95835998174186
Pt	14.00821636132950	11.99330433559628	19.98717366485682
Pt	12.01448003281170	13.98386904655056	19.98048221512092
Pt	14.01267247796254	15.97945155994867	19.97705143032289
Pt	15.99740185779585	13.98195222935482	19.98811119266137
Au	11.87843504564870	7.92208402223879	5.88762074207767
Au	13.99915172408875	6.04761473644152	6.00001805119452
Au	14.00053527761686	8.03043717315779	3.96092689344791
Au	16.12731098523140	7.91041032718621	5.88816877964925
Au	7.82294544694474	11.80607712201016	5.80844034165274
Au	9.87428026351381	9.88843382879422	5.87812342825628
Au	9.96493482217787	11.94015871220743	3.86199779080148
Au	11.95694015492918	9.96927996017393	3.86101437667396
Au	11.99778102571541	11.94634815089010	5.94444506787995
Au	14.01631668392117	9.96892094002648	5.99263544708246
Au	14.00912983998240	11.96460890437773	3.73956286714188
Au	16.04944712849825	9.96259700858285	3.86486949690783
Au	16.03957883359870	11.95076455949846	5.95436996231970
Au	18.12872346808684	9.86922135215852	5.86551449244819
Au	18.03937079863324	11.94625722653738	3.86481625502446
Au	20.09767504082451	11.86274267488621	5.87622408774453
Au	5.96101431434016	13.99046620367874	5.95802112811945
Au	8.01848553029234	13.98775324070718	4.00448709976790
Au	7.80495877180398	16.17968187361202	5.80181363197795
Au	10.00012930056103	13.93034763148048	5.97772987135844
Au	9.98038071403577	16.01147195944528	3.92263789964741
Au	12.01334915403982	13.96395976465715	3.72075143864524
Au	12.01213412312055	15.95162159013091	5.91947609272143
Au	14.02355394327163	13.95157813396791	5.89621424418511
Au	14.02812605558249	15.97500169811521	3.73236783947476
Au	16.01849792993480	13.96469645535503	3.72673761180450
Au	16.04432379629119	15.96551944727327	5.94081069574029
Au	18.02725364377226	13.96108386170658	5.99053749648912
Au	18.01856055045561	16.02600439813787	3.87161357920829
Au	19.97217285628525	13.99680517396691	3.96023763342275
Au	20.05738087663169	16.09544910825305	5.92603643304458
Au	21.96378704978030	13.99725964641430	5.99298534639884
Au	9.81561341593367	18.17812886503341	5.80960819129411
Au	11.96454458058507	18.01008999795028	3.91855353100623
Au	11.77364115279180	20.22223891027373	5.76773026620673
Au	14.02663868295894	17.96215294847012	5.96301161157697
Au	14.00209699850224	19.96638906533588	4.03407933090951
Au	16.05817705370414	18.03109993110815	3.89894152675771
Au	16.24976774347321	20.24267642753211	5.75270590432809
Au	18.14889686510677	18.15483185887242	5.84132568020828
Au	14.00690849025389	22.03628936213040	5.95864656797046
Au	11.96929258873458	3.98480208855640	9.99593197684933
Au	14.00037326752866	4.05822025078550	8.06957462874053
Au	16.03072173759737	3.97539005541634	9.99821104684115
Au	7.89034023188682	7.91659464471206	9.92913730392588
Au	9.87115315140255	5.91761987763096	9.90802129109643
Au	9.89022821742957	7.92526714630108	7.90581526573041
Au	11.90763955369816	5.95693687054909	7.91539185084039
Au	16.09656909240682	5.94623813539696	7.91648795225694
Au	18.12965050288646	5.90630768273044	9.90997341532812

Au	18.11864623379813	7.89966098968344	7.89582004427538
Au	20.12661244369999	7.87583865156267	9.91036178491014
Au	3.84573819939123	11.93714310532160	9.95643413862494
Au	5.86303235504033	9.87477051092958	9.87367864695516
Au	5.80684900997317	11.80952058568026	7.82819920718017
Au	7.90543519505545	9.94046449912287	7.91714595633388
Au	20.10793012771600	9.87691588531677	7.88980470387747
Au	22.10030265403963	9.86813652237742	9.89986226601451
Au	22.07501640009540	11.88145635638141	7.89347149071340
Au	24.03988944790425	11.96032774141781	9.97711572328538
Au	4.00098519630746	13.98589757452464	8.00828901387638
Au	3.91029016895386	16.02795841387439	9.95763522934641
Au	5.96811629443732	13.94491603061632	9.97992130201666
Au	5.80014442201817	16.18585245588511	7.79344032779377
Au	7.97950566617066	13.90080493653988	7.96968095950938
Au	7.92504816578939	16.02357651326626	9.92962797737879
Au	9.94766406750366	16.01447613593526	7.92594872002266
Au	22.02770728019757	16.07178376669538	7.95579745977127
Au	23.95787685270028	13.99223510494994	8.05002945712783
Au	24.07668844137291	16.02922803107379	9.97589282596350
Au	5.78842817293265	18.20232496174957	9.79120757351988
Au	7.80697673511855	18.17765358306977	7.80616132944413
Au	7.79917741519066	20.19852851633714	9.80952010728683
Au	9.94653964940956	18.04771483155624	9.93927853993012
Au	9.81705597285864	20.19114850538576	7.80344801310564
Au	11.96849982657518	18.03399795858848	7.92472106328547
Au	11.96679841861153	20.06866072192811	9.93373967848378
Au	14.02639970595652	20.03089258911583	7.93577280824038
Au	16.06910372557212	18.02089013557655	7.95165834098759
Au	16.06648169027268	20.05863038960963	9.93446762804352
Au	18.15425667388229	20.18218583190148	7.83808636557221
Au	20.03453092932855	18.07945321213570	7.95712841515347
Au	20.15804687262670	20.18549872081759	9.84407304564980
Au	22.15540342720255	18.17038603215167	9.85054897531660
Au	9.79719030925708	22.20800007879053	9.79406839870028
Au	11.78234442368659	22.21837147288997	7.77439387331088
Au	11.95520899272227	24.10159612804090	9.95680732934706
Au	14.01724725201957	22.04491422618095	9.98975657679997
Au	14.00675535317259	23.98304386090582	8.01338870741910
Au	16.24333765811592	22.25180672813156	7.75545190356013
Au	16.05597671275723	24.12074320729224	9.95556435754384
Au	18.21115233294653	22.24274675582618	9.78781667957188
Au	8.01573958973706	4.00213833162111	13.98296836435078
Au	9.98327922035120	3.92242496370082	11.94794499394589
Au	12.03198979885552	3.82067347906302	13.93931977511247
Au	14.00119677019737	3.87092971815905	11.96341956924970
Au	15.97414924678277	3.82302989173796	13.93635571323898
Au	18.01955709711751	3.91399073332173	11.95048787295982
Au	19.98796820534836	3.99543156801155	13.98307982578532
Au	3.99994460510226	8.00566737958304	13.99108711200002
Au	5.95189921040068	5.95214075722008	13.99461302391482
Au	5.79450656763702	7.82297866061604	11.80351919478869
Au	7.78542687738529	5.80088953172362	11.79556481110727
Au	7.97046498470713	7.95507597591068	13.91153232581492
Au	9.99954755043024	5.97553905751863	13.93349486641746
Au	18.01481239409635	5.98186082514331	13.92435589757400
Au	20.22146052672154	5.77848801503786	11.79121831767048
Au	20.04181225875728	7.96357088792701	13.90267607219254
Au	22.05163791107914	5.94789779243315	13.98931559401585
Au	22.22143686007893	7.77883650592677	11.78897681636296
Au	23.99956702057931	8.01189111549743	13.98519746065378
Au	3.84493358233652	9.95492616360845	11.93949782768203

Au	3.69372409463810	11.97731371121814	13.97375399294141
Au	5.97346990522837	9.97043804581181	13.94240252051924
Au	5.93208387437979	11.95297728444219	11.95841355272179
Au	22.02097089215798	9.98914757266173	13.93149935831140
Au	24.07885719158240	9.98030677100563	11.95007021580623
Au	24.19625220475972	12.00864179993792	13.95266572824496
Au	3.69208187695079	13.97374776183925	11.98373487754460
Au	3.68092441191323	15.98778178773856	13.98439509971955
Au	5.87547322605517	13.96468654831181	13.96661943839577
Au	5.90388307397269	15.97283934294459	11.98443040444406
Au	22.05993990423549	15.98790793202239	11.97072905532554
Au	24.15975706725279	13.97443232010347	11.96493622741113
Au	24.23362560234008	15.96167595087289	13.95749655440226
Au	3.89963067162364	18.03066496933873	11.94302318396517
Au	4.02011479584816	19.97809068764413	13.99703561525810
Au	5.94746314262428	17.98233072411900	13.98897047965825
Au	5.76065914229439	20.23231940123510	11.76293713611407
Au	7.92717313909078	18.04362760270361	11.95098686837962
Au	7.94077469140227	20.05171502516254	13.99140680537018
Au	9.94791420089905	20.06982187218571	11.95207675978189
Au	18.06200973064686	20.05455182074493	11.93656665157717
Au	20.05885179908019	18.03784362534006	11.93587800674386
Au	20.06418168650676	20.04525308702542	13.97604613094855
Au	22.04782532142690	17.99725841917836	13.97065982026919
Au	22.25130635869634	20.25600788667584	11.75197022950308
Au	24.11171611151901	18.04660352493118	11.93945218061241
Au	23.98547912393263	19.98321233832672	13.99533009243306
Au	5.95617834139667	22.04155457102438	14.00183659431020
Au	7.76724360642267	22.23395240664327	11.76552448849829
Au	8.01633860989195	23.97912545028282	13.99751132530916
Au	10.00599961757139	22.05065952047303	13.99550451929565
Au	9.96393524767959	24.10405268618209	11.94735893113471
Au	12.01086658910076	22.09948306701880	11.99410878093367
Au	12.00011431637814	24.33513505077502	13.99242343552929
Au	14.00792115153244	22.13852709476113	13.98458297020001
Au	14.00485450717992	24.33378306518719	11.99119125835235
Au	16.01254815328481	22.10372589514693	11.98771260997490
Au	16.01144011399242	24.33599881333633	13.99006841660626
Au	18.01305078998310	22.04428119578292	13.97749487954875
Au	18.04313427618276	24.12395085735686	11.94728030279819
Au	20.24153175776410	22.25167845783092	11.75752771063190
Au	19.99003196732463	23.98469107006368	13.99552716553181
Au	22.04717048019273	22.04441878806325	13.99646292683323
Au	9.98930225332496	3.91594805766183	16.00600173305602
Au	11.96399292352620	3.89717842879828	18.01781270980161
Au	14.00761229306606	3.75999943602277	15.95195329712909
Au	16.04473704018132	3.89923649355304	18.02054298094523
Au	18.01584977737720	3.91914658870651	16.00508456899962
Au	5.79730646465055	7.78211965490353	16.19524550593470
Au	7.81981133528423	5.80821163918249	16.16766191294628
Au	7.81163975119381	7.80171580543083	18.17830479916192
Au	9.83667567360562	5.81465521239575	18.16488576463005
Au	9.95487765342544	7.92109858209801	16.01970993426321
Au	12.03133599736471	5.91705614021428	15.96387001557518
Au	11.97503742302132	7.93315114343704	18.03705785650434
Au	14.00584081295580	5.95025965143708	18.00565099306460
Au	15.98269525110850	5.92072150913072	15.96077388039866
Au	16.04119294308461	7.93052994444835	18.03809060872723
Au	18.17624088921255	5.80822997403778	18.17605875126174
Au	18.05760694801739	7.92621899994830	16.01571638955896
Au	20.17638213246158	5.81953233159023	16.15900924421953
Au	20.18911993781773	7.80826294255805	18.17808724345095

Au	22.18260958825586	7.81417025844683	16.16725691336521
Au	3.91499127292078	9.95477535555219	16.02354461206576
Au	3.90719711550233	11.94457288901637	18.02276627676648
Au	5.80083878167882	9.79550714813197	18.18987243105251
Au	5.91245653800804	11.97860930645256	15.96941987398044
Au	7.93812883294509	9.92746295961425	16.01703207133856
Au	7.93862253374784	11.95147056553131	18.03240047181984
Au	9.96155178220589	9.94597311022493	18.03637657345332
Au	18.04917386357261	9.94688014219509	18.03601759732295
Au	20.06826067950951	9.94108471613786	16.01522875501974
Au	20.06648527209462	11.95594250267846	18.04066343403278
Au	22.19024155812816	9.81612430297405	18.18136522299121
Au	22.08398340650473	11.99766376235447	15.98243994093630
Au	24.08664226850904	9.96925779054256	16.01835842160837
Au	24.10395397249254	11.94768456187120	18.03261569032474
Au	3.68072426745322	13.98405882554332	15.98060239907850
Au	3.89804537285127	16.03750422262871	18.01893836111330
Au	5.94883561755155	13.98504318418647	17.97871814415162
Au	5.90924394130377	15.97238057310252	15.97368952144671
Au	7.94150762568540	16.01403360899588	18.02366431298774
Au	20.06283922790051	16.01329534317781	18.02751848237048
Au	22.05251195881412	13.99101895836685	18.00692088357176
Au	22.08557169525994	15.95535158269474	15.95555105911063
Au	24.25004855211335	13.98012069090639	15.96011191337449
Au	24.10056993096462	16.03464549179943	18.01891894583817
Au	3.89766367136612	18.02324518192591	16.04070499456039
Au	5.80494748958763	18.18209457298613	18.17978603772072
Au	5.76904017528999	20.22726285617635	16.22548303186687
Au	7.93891735759083	18.03163978786708	16.01754413436620
Au	7.81598234948542	20.18107379204722	18.16717794236880
Au	9.96626040777362	18.02573757638078	18.02320071376246
Au	9.95753124671116	20.05720500145137	16.02114708793583
Au	11.97442603928366	20.05403368709283	18.02750023258843
Au	16.04108629562417	20.05811110302366	18.03367819098732
Au	18.04280657359366	18.02505229924477	18.02777114833738
Au	18.05778220367019	20.05546234987566	16.01814500271853
Au	20.06876347603148	18.02603769537000	16.01438889152878
Au	20.19043517821593	20.18025856997613	18.17215980986132
Au	22.18288492727181	18.16235487243414	18.16576631101079
Au	22.22489818293029	20.21572269117367	16.21373198021914
Au	24.09369850956686	18.01350978999910	16.02821166905161
Au	7.76414486475758	22.23356936492418	16.23036842723219
Au	9.81003727673094	22.19648023612229	18.18586266687067
Au	9.96626392342438	24.10383347885370	16.04607235252343
Au	12.01234225243295	22.09537394459403	15.98490242666453
Au	11.95584486975392	24.10484713732350	18.02602766895428
Au	14.00319499708375	22.04912831762166	17.98774755526304
Au	14.00392184597988	24.33466153121607	15.98736785108614
Au	16.00249067470306	22.09236149655064	15.97959172767214
Au	16.04901156505642	24.09548266785315	18.02559005296744
Au	18.19944501256489	22.19526616859289	18.18904240640432
Au	18.04168865272840	24.09412146586048	16.03899804856475
Au	20.23463503098313	22.22325985460569	16.22237782752185
Au	13.99954802755814	4.00834045985784	19.98573548013958
Au	9.82050522954136	7.79966611089017	20.19026132435122
Au	11.77410962860748	5.76197464661483	20.22849904160725
Au	11.76653907046470	7.75813846600592	22.23606461303342
Au	14.00199418049084	5.95085144620093	22.04231422783858
Au	14.00667153763890	7.94333200081581	20.05710182107694
Au	16.23361052243868	5.76057782088336	20.23088091173601
Au	16.23890163644353	7.75896449868947	22.24050050547565
Au	18.18919165247751	7.79525503857641	20.19842293519130

Au	5.78001086133738	11.76924649208535	20.22108162226718
Au	7.81320767882327	9.81265382858463	20.18892417901176
Au	7.77125118016441	11.76594467379478	22.23191525750130
Au	9.79937348599036	9.79049568739266	22.20957507997917
Au	9.95246066535320	11.95274781960807	20.06306522536681
Au	11.96846310143611	9.94269416265519	20.06454064916415
Au	12.00393589855830	11.99761808172295	22.10073883128705
Au	14.00445404749489	9.99697867276055	22.05748442673461
Au	16.04649710796830	9.94020855669123	20.06751748628997
Au	16.00533572183349	11.99318199000286	22.10588315531488
Au	18.20502090990919	9.79216112017696	22.20979613507443
Au	18.05724503395944	11.95302749326985	20.06456353421996
Au	20.19827863137802	9.81068551847866	20.19685635663090
Au	20.23488362218971	11.76244582259323	22.24017185249429
Au	22.23184725769244	11.76941430138767	20.23100413564667
Au	4.02124843804892	13.99505207150627	19.97871752072946
Au	5.95644165430183	13.99703381313854	22.04051608758419
Au	5.77844805698758	16.21845178262787	20.21764267122225
Au	7.94610822143732	13.98875427917661	20.04466397595253
Au	7.77088258277143	16.22486527442550	22.22599680038236
Au	10.00271934538004	13.99399471219042	22.05149745980216
Au	9.95806815027642	16.01929664605141	20.05326567354228
Au	12.01139266706583	15.98584349191699	22.09147720128605
Au	14.00378978127787	13.98928204375600	22.12836612618195
Au	15.99740671191703	15.98852020723405	22.09873609075675
Au	18.00422788303087	13.99317589012804	22.05733379754782
Au	18.05133096666388	16.02164071805971	20.05898280069752
Au	20.05874386997737	13.99097800471237	20.05857545686414
Au	20.23814250836314	16.22922482828334	22.23645263950209
Au	22.05011434596457	13.99781063337019	22.04959155220494
Au	22.22490541184434	16.21528736226970	20.22179633051740
Au	23.99040592646121	13.99708926723006	19.99028306878910
Au	7.82382807236961	18.16273895033128	20.17013944658247
Au	9.81140616584136	18.18754951597432	22.18999527064270
Au	9.83197353698704	20.17548291305324	20.17230298991242
Au	11.97341066329106	18.03465718574283	20.05134727548981
Au	11.77227972179057	20.22513775561902	22.22458345517903
Au	14.00270497791811	17.99184761256718	22.04991554088720
Au	14.00384499736029	20.04845787007171	20.04624558585404
Au	16.04202471049662	18.03997962899968	20.06089201405818
Au	16.23041078414828	20.22450986398339	22.22836014302777
Au	18.20320534283783	18.19490579620648	22.20402955157019
Au	18.17916975581925	20.17858977198383	20.18200695909152
Au	20.18801886224215	18.16934218918686	20.18188117653679
Au	11.77467909168559	22.22308890985620	20.22168295068599
Au	13.99979622323751	22.04183319643189	22.04054150868429
Au	14.00259763464216	23.97798383443319	19.98188716744366
Au	16.22705192254531	22.21698277745734	20.22141988820165
Au	14.00214966583029	8.01149597200679	23.98025989408417
Au	9.96313732670812	11.94322788905599	24.10953542463100
Au	11.94904052640025	9.95544339712467	24.10798646645994
Au	14.00330760782135	11.99233527954427	24.32977383290587
Au	16.05899268752942	9.95301552891302	24.11486547763024
Au	18.04517725027371	11.94172102450992	24.11550827349705
Au	8.01814144628889	13.99649431859577	23.98085889051733
Au	9.96631083264284	16.04603983167435	24.10032409322999
Au	12.00023155026276	13.99315245539619	24.33372294981098
Au	14.00150578640489	15.99089688753965	24.33367058742218
Au	16.00751851470985	13.99224303478095	24.33166198394566
Au	18.04071916259852	16.04734343597416	24.10515314388831
Au	19.98935079856450	13.99701115905454	23.98523121993174
Au	11.95344773007843	18.02930197764841	24.10238953714859

Au 14.00111039022448 19.97836817210753 23.97875286299568
Au 16.05259197309847 18.03231647211823 24.10697482495542

$Pt_{43}Au_{158}L1_1$-706.178
Pt 8.53006331236053 12.50000000000000 16.46991168763954
Pt 12.50000000000000 14.49818000409735 14.49820500409729
Pt 10.43489807212588 18.50810961188962 12.44335099160381
Pt 12.50000000000000 10.50181999590265 10.50181999590265
Pt 12.50000000000000 8.53008831236046 8.53008831236046
Pt 14.56510192787412 6.49189038811038 12.55662400839626
Pt 14.59307746110703 10.40694753889291 16.55703896579919
Pt 18.50808461188951 12.44335099160381 14.56510192787412
Pt 8.44852941045566 12.59166680363215 8.44852941045566
Pt 10.40692253889297 16.55706396579912 10.40694753889291
Pt 12.59169180363209 8.44855441045560 16.55144558954440
Pt 8.53006331236053 8.53008831236046 12.50000000000000
Pt 14.59307746110703 8.44293603420088 14.59307746110703
Pt 14.56510192787412 12.44335099160381 18.50810961188962
Pt 10.43489807212588 12.55664900839619 6.49191538811049
Pt 10.45704401397707 14.54293098602299 16.47296285858690
Pt 12.40833319636785 16.55144558954440 8.44852941045566
Pt 8.44293603420088 14.59305246110709 10.40694753889291
Pt 8.52703714141310 10.45706901397701 14.54295598602293
Pt 16.55706396579912 10.40694753889291 14.59307746110703
Pt 12.50000000000000 16.46991168763954 16.46991168763954
Pt 12.55664900839619 6.49189038811038 14.56510192787412
Pt 14.49820500409729 12.50000000000000 10.50181999590265
Pt 18.50808461188951 10.43489807212588 12.55662400839626
Pt 14.54295598602293 16.47296285858690 14.54295598602293
Pt 6.49191538811049 12.55664900839619 10.43489807212588
Pt 10.50179499590271 10.50181999590265 12.50000000000000
Pt 16.46991168763954 12.50000000000000 8.53008831236046
Pt 6.49191538811049 14.56510192787412 12.44335099160381
Pt 10.50179499590271 12.50000000000000 14.49820500409729
Pt 14.49820500409729 14.49818000409735 12.50000000000000
Pt 10.45704401397707 8.52703714141310 10.45704401397707
Pt 16.55147058954434 12.40833319636785 16.55144558954440
Pt 12.44337599160374 18.50810961188962 10.43489807212588
Pt 8.44852941045566 16.55144558954440 12.40830819636791
Pt 16.47296285858690 14.54293098602299 10.45704401397707
Pt 12.44337599160374 14.56510192787412 6.49191538811049
Pt 10.40692253889297 14.59305246110709 8.44293603420088
Pt 12.50000000000000 12.50000000000000 12.50000000000000
Pt 16.46991168763954 16.46991168763954 12.50000000000000
Pt 12.55664900839619 10.43489807212588 18.50810961188962
Pt 16.55147058954434 8.44855441045560 12.59166680363215
Pt 14.54295598602293 10.45706901397701 8.52703714141310
Au 20.50131465524583 8.50000684645327 12.51535404182462
Au 16.50734188113336 12.49337501004195 4.46268100509872
Au 10.47295881556192 4.38241575984737 10.47295881556192
Au 10.43674664308582 10.40961700716299 16.58498871268961
Au 12.51887047551428 12.48115452448584 16.55343423188533
Au 18.69611537196558 16.68474025758453 14.69164907788709
Au 10.32830963190445 18.66250109439438 8.32927723937882
Au 14.54422015957171 12.49333327832183 14.54422015957171
Au 10.45577984042829 14.54419515957177 12.49330827832189
Au 18.62517359583311 14.61076250682982 16.64708663325989
Au 10.45254125615660 6.47911998523600 12.49562650769800
Au 10.40959200716287 16.58501371268954 14.56325335691417
Au 14.67166536809561 6.33749890560562 16.67072276062118
Au 16.50734188113336 20.53731899490128 12.50662498995805
Au 8.23556670184811 8.26031061767481 8.23559170184805

Au	10.39097343373143	6.43168952478774	8.40712530111567
Au	16.70232168824199	8.29767831175801	16.70229668824206
Au	12.46798753900969	18.56575302949430	6.43427197050581
Au	16.55345923188545	12.48115452448584	12.51884547551416
Au	10.30835092211291	18.69614037196552	16.68471525758441
Au	16.64711163325983	14.61076250682982	18.62517359583311
Au	14.60902656626857	8.40712530111567	6.43171452478768
Au	18.49255786830248	12.50000000000000	6.50744213169751
Au	20.53731899490128	12.50662498995805	8.49268311886675
Au	12.51537904182474	4.49868534475417	16.49996815354680
Au	10.39097343373143	16.59287469888433	18.56828547521232
Au	16.76440829815196	16.73968938232520	16.76440829815196
Au	18.56575302949430	6.43424697050570	12.53201246099032
Au	8.35288836674017	18.62519859583305	14.61078750682976
Au	8.35288836674017	10.38923749317018	6.37482640416689
Au	8.31525974241547	10.30837592211302	18.69611537196558
Au	12.52282197964902	4.41256741395612	12.52279697964891
Au	14.56325335691417	16.58501371268954	10.40959200716287
Au	14.53342323129441	10.46657676870559	20.56918914759454
Au	10.48429610037260	20.53479792617203	14.49529528755509
Au	6.37482640416689	16.64711163325983	14.61078750682976
Au	12.48115452448584	12.51884547551416	8.44654076811455
Au	8.41498628731046	14.59038299283701	14.56325335691417
Au	12.51537904182474	8.50000684645327	20.50131465524583
Au	20.56918914759454	10.46657676870559	14.53344823129434
Au	6.37482640416689	10.38923749317018	8.35288836674017
Au	12.50669172167811	10.45580484042823	14.54422015957171
Au	10.45577984042829	12.50666672167817	10.45577984042829
Au	6.33749890560562	14.67166536809561	8.32927723937882
Au	16.58501371268954	10.40961700716299	10.43674664308582
Au	4.46268100509872	8.49268311886675	12.50662498995805
Au	12.52282197964902	12.47720302035109	20.58740758604376
Au	10.50470471244491	4.46520207382797	14.51570389962740
Au	12.50000000000000	6.50744213169751	6.50744213169751
Au	18.62517359583311	8.35288836674017	10.38923749317018
Au	16.58501371268954	14.56322835691424	14.59040799283713
Au	12.49565150769811	14.54743374384346	18.52085501476406
Au	16.68474025758453	14.69162407788698	6.30388462803442
Au	8.50000684645327	20.50131465524583	12.48462095817526
Au	6.33749890560562	16.67072276062118	10.32833463190439
Au	14.53342323129441	4.43081085240547	14.53344823129434
Au	10.47295881556192	14.52704118443808	20.61758424015263
Au	14.54745874384340	18.52088001476400	12.50437349230200
Au	14.49527028755497	14.51567889962746	4.46520207382797
Au	8.40712530111567	14.60902656626857	18.56828547521232
Au	4.46268100509872	12.49337501004195	16.50731688113325
Au	10.38921249317024	8.35288836674017	18.62517359583311
Au	8.32925223937888	14.67166536809561	6.33752390560556
Au	14.52704118443808	10.47295881556192	4.38241575984737
Au	10.38921249317024	6.37480140416695	16.64708663325989
Au	16.59287469888433	18.56831047521226	14.60905156626850
Au	6.47914498523594	12.49562650769800	14.54745874384340
Au	6.47914498523594	10.45256625615654	12.50437349230200
Au	18.49255786830248	18.49255786830248	12.50000000000000
Au	18.52085501476406	12.50437349230200	10.45254125615660
Au	8.44654076811455	12.51884547551416	12.48115452448584
Au	8.49265811886663	12.50662498995805	20.53731899490128
Au	12.49337501004195	16.50731688113325	20.53731899490128
Au	16.67074776062112	6.33749890560562	14.67169036809555
Au	4.49868534475417	12.51535404182462	8.50000684645327
Au	10.40959200716287	10.43674664308582	8.41498628731046
Au	14.69162407788698	16.68474025758453	18.69611537196558

Au	4.38241575984737	10.47295881556192	14.52704118443808
Au	18.56575302949430	12.46798753900969	18.56575302949430
Au	12.50437349230200	18.52088001476400	14.54745874384340
Au	14.61076250682982	16.64711163325983	6.37482640416689
Au	6.43171452478768	8.40712530111567	14.60905156626850
Au	12.48115452448584	16.55343423188533	12.48115452448584
Au	10.30835092211291	8.31525974241547	6.30388462803442
Au	20.61758424015263	14.52704118443808	10.47295881556192
Au	12.50000000000000	18.49255786830248	18.49255786830248
Au	14.54422015957171	10.45580484042823	12.50666672167817
Au	14.51570389962740	4.46520207382797	10.50472971244503
Au	12.48464595817538	20.50131465524583	8.50000684645327
Au	20.53479792617203	14.49527028755497	14.51570389962740
Au	16.68474025758453	18.69614037196552	10.30837592211302
Au	12.49337501004195	4.46268100509872	8.49268311886675
Au	8.50000684645327	12.51535404182462	4.49868534475417
Au	8.49265811886663	4.46268100509872	12.49337501004195
Au	14.49527028755497	20.53479792617203	10.48429610037260
Au	14.69162407788698	6.30385962803448	8.31525974241547
Au	14.61076250682982	18.62519859583305	8.35288836674017
Au	16.59287469888433	10.39097343373143	6.43171452478768
Au	12.50437349230200	10.45256625615654	6.47914498523594
Au	12.49333327832183	14.54419515957177	10.45577984042829
Au	10.43674664308582	8.41498628731046	14.59040799283713
Au	10.45254125615660	12.50437349230200	18.52085501476406
Au	18.66247609439444	8.32927723937882	14.67169036809555
Au	6.30388462803442	8.31525974241547	10.30837592211302
Au	12.53201246099032	6.43424697050570	18.56575302949430
Au	12.50664998995816	20.53731899490128	16.50731688113325
Au	10.46655176870566	14.53342323129441	4.43081085240547
Au	14.59040799283713	14.56322835691424	16.58498871268961
Au	10.32830963190445	16.67072276062118	6.33752390560556
Au	8.31525974241547	6.30385962803448	14.69164907788709
Au	16.76440829815196	8.23559170184805	8.26028561767469
Au	14.52704118443808	20.61758424015263	14.52704118443808
Au	16.49999315354673	4.49868534475417	12.51535404182462
Au	20.58743258604387	12.47720302035109	12.52279697964891
Au	18.66247609439444	10.32833463190439	16.67072276062118
Au	8.41498628731046	10.43674664308582	10.40959200716287
Au	8.32925223937888	18.66250109439438	10.32833463190439
Au	14.60902656626857	18.56831047521226	16.59284969888422
Au	4.46520207382797	14.51567889962746	14.49529528755509
Au	18.56828547521232	16.59287469888433	10.39097343373143
Au	12.49565150769811	6.47911998523600	10.45254125615660
Au	12.47720302035109	20.58743258604387	12.47717802035098
Au	14.54745874384340	12.49562650769800	6.47914498523594
Au	18.52085501476406	14.54743374384346	12.49562650769800
Au	18.69611537196558	10.30837592211302	8.31525974241547
Au	4.49868534475417	16.49999315354673	12.48462095817526
Au	16.67074776062112	10.32833463190439	18.66250109439438
Au	12.51887047551428	8.44656576811467	12.51884547551416
Au	14.51570389962740	14.49527028755497	20.53479792617203
Au	12.47720302035109	12.52279697964891	4.41256741395612
Au	6.43424697050570	12.53201246099032	6.43427197050581
Au	6.50744213169751	6.50744213169751	12.50000000000000
Au	20.53479792617203	10.48432110037254	10.50472971244503
Au	6.43424697050570	18.56575302949430	12.46798753900969
Au	14.67166536809561	8.32927723937882	18.66250109439438
Au	16.64711163325983	6.37480140416695	10.38923749317018
Au	6.30388462803442	14.69162407788698	16.68471525758441
Au	6.50744213169751	12.50000000000000	18.49255786830248
Au	16.73971438232531	16.76440829815196	8.23559170184805

Au	20.53731899490128	16.50731688113325	12.49337501004195
Au	8.26028561767469	8.23559170184805	16.76440829815196
Au	18.56828547521232	14.60902656626857	8.40712530111567
Au	8.23556670184811	16.76440829815196	16.73968938232520
Au	4.43081085240547	14.53342323129441	10.46657676870559
Au	4.41259241395624	12.52279697964891	12.47717802035098
Au	12.50664998995816	8.49268311886675	4.46268100509872
Au	14.56325335691417	14.59038299283701	8.41498628731046
Au	10.50470471244491	10.48432110037254	20.53479792617203
Au	12.48464595817538	16.49999315354673	4.49868534475417
Au	10.46655176870566	20.56918914759454	10.46657676870559
Au	8.40712530111567	6.43168952478774	10.39097343373143
Au	4.46520207382797	10.50472971244503	10.48429610037260
Au	20.50131465524583	12.48464595817538	16.49996815354680
Au	10.48429610037260	10.50472971244503	4.46520207382797
Au	14.59040799283713	8.41498628731046	10.43674664308582
Au	6.43171452478768	10.39097343373143	16.59284969888422
Au	8.29767831175801	16.70232168824199	8.29767831175801
Au	16.49999315354673	12.48464595817538	20.50131465524583

$Pt_{43}Au_{158}$LEH-708.642

Pt	12.53406817850735	16.47150651099471	12.49356782775291
Pt	16.42430567378105	16.46919760653528	12.48915580967391
Pt	10.52326228837338	14.50241168822779	16.49488300323123
Pt	10.53419476174451	10.52021607343741	12.52467724315750
Pt	12.53088101627305	12.50678179949594	16.51541669623249
Pt	12.53698140847237	12.49328356002681	12.51567852194547
Pt	14.47730747647685	14.47603827923730	12.49916952662853
Pt	12.52820053550249	8.56696882871982	12.50150618018719
Pt	14.51328812280858	10.49909353589043	12.52526453462355
Pt	14.50848950242001	10.50513118863552	8.46686708195879
Pt	12.52780180882918	12.52172120836309	8.46875131809350
Pt	16.42810301345357	12.51626332844067	12.49845661441292
Pt	16.43733236294860	12.52221268718231	8.46426316294896
Pt	12.51255521497932	16.46691951440954	16.48201868272297
Pt	10.54966446403748	14.48400871790933	12.45600190997199
Pt	14.51034049943548	14.53146901796929	16.51449328073080
Pt	14.47291955989886	18.46484687606994	12.49513085018889
Pt	10.54471613358723	10.53037183834873	8.51952498232710
Pt	14.49387415876929	14.53104829045481	8.46851620945661
Pt	12.50643701030846	8.56712381872330	8.48603486117618
Pt	8.57651657793145	12.50940980046889	12.49562243790107
Pt	14.52264996967034	12.48116208778809	14.55169130076504
Pt	16.41699297778828	14.53043175529156	10.44386861887194
Pt	12.51964018821645	14.51511782518551	18.51023661835515
Pt	14.47608528174136	12.51991175968226	6.47192633113732
Pt	12.53554469952686	10.53014056550665	14.51758152034496
Pt	12.52753082244582	14.43901978639160	14.49658818345871
Pt	10.55337416972190	16.41970462117636	14.49702269212524
Pt	10.52710046044543	12.47644263311111	14.52001480031899
Pt	14.48473782958795	16.48294314095191	10.46253948492510
Pt	14.50277159919144	8.56209461406101	10.44773464987964
Pt	10.52435108782599	8.58034125781325	10.47784695353288
Pt	12.51579875668710	14.48938885879199	10.48148072356778
Pt	16.43379196952277	14.52431717540944	14.50880543717751
Pt	12.52916812622560	18.45532110458871	14.49655768107602
Pt	14.47950522459105	12.50799928467959	10.48739888464578
Pt	8.55113263777882	14.47166594358163	14.50256855798018
Pt	12.52572330030061	10.53039614960302	6.47707069875881
Pt	12.52527729057609	10.54565305417080	10.52680321677894
Pt	8.54325177714864	10.53799293931011	10.49347078338793
Pt	16.43474308926991	10.54007327028328	10.44620193628200

Pt	14.50868352913046	16.47634825172863	14.52178780195761
Pt	10.54473927222672	12.54957169012932	10.48504420664967
Au	14.51527344710297	14.56330500216564	4.35077619468603
Au	12.51604172993244	6.48473806805052	18.55805677204637
Au	8.29839027003352	10.28889165525044	18.73010747604421
Au	12.50371828120005	4.56209504336915	16.51075834196151
Au	20.47012582027634	12.50334141567620	16.52193550659368
Au	10.50480988407238	14.50572818925825	20.59528262860310
Au	10.42301099418359	18.50603204903295	16.53930342260021
Au	20.61658543267241	14.52559953667128	14.55192065709825
Au	20.51222873080291	10.50151290826001	14.52818762834108
Au	10.48630758738022	14.57132851715709	4.36777429288923
Au	10.27883421484510	8.27972340883606	18.75034878579578
Au	4.37609414767416	14.51255448702656	14.52354756685799
Au	14.76144871464640	6.25194833816547	16.78169309499960
Au	6.29993735941893	10.29911207259693	16.72636217963214
Au	12.49659572681556	6.47936825805171	6.49236595047758
Au	14.60979155026810	16.55355937819662	18.61025019843741
Au	14.49983304232673	4.37618453383072	10.47787357551247
Au	6.30747104425923	8.31460777180366	14.71787468019827
Au	14.61830035006018	6.39729427701003	8.39573229320337
Au	10.49204071210728	20.56503923564917	10.43214420527586
Au	8.36084904581260	6.36667993454130	10.33311452688207
Au	10.31827880335894	16.74160074458498	6.23630936244191
Au	18.69205723829766	8.30043636529409	14.72668757032422
Au	14.50381919667114	20.51368111900566	14.51816056235897
Au	6.30092822215405	14.73841186427359	8.22981032434212
Au	6.47336004989630	6.49199914312366	12.50872021250946
Au	8.35880162265553	18.61667110060273	14.63305954504317
Au	18.51797055276004	6.47647244659563	12.50917564922102
Au	6.48094545331940	12.53115880814365	6.41041690800216
Au	18.60583305492474	16.56537639544513	14.63447892451550
Au	16.75403312820753	8.24584589116412	16.78569299400749
Au	6.34262772862032	10.35971712646245	8.33020263288648
Au	6.30126016556027	16.72553485516714	10.24282427708042
Au	16.62763903298583	16.55800603144291	16.63780390120308
Au	14.60032695251375	18.53276604388550	16.60686626215289
Au	16.64730007065057	6.33034982612635	10.33490648179427
Au	12.50766507089030	4.25825501016230	12.51499689489843
Au	10.51529244088590	20.54020545464781	14.50276416014109
Au	14.54257731814441	4.44275144832127	14.55725165453450
Au	12.52488336819366	12.51946046696189	4.31105699758510
Au	8.31450815871298	18.70549508682335	10.25310610750281
Au	10.39157975968769	6.43243564041963	8.40069234342820
Au	12.52329909783683	20.61621277759823	12.48844045148378
Au	18.67135479730346	8.33749261850387	10.31691229118865
Au	10.42444205687953	8.47826464854691	6.44075568909008
Au	16.72202475957788	6.27987376539472	14.74769794619598
Au	14.50555562426752	10.48782436176325	4.42505510948474
Au	14.59306802088831	8.40913476707061	6.41743247444745
Au	18.48392292529702	18.45131626738194	12.49131113985621
Au	6.34260573694594	16.62032626254253	14.65040130728520
Au	16.58165383032922	18.52943768102267	14.59760713315637
Au	18.57410066708852	14.63411339894540	8.36045903212999
Au	10.46944311737569	10.45986563860860	20.61665613079448
Au	6.35509976172861	8.37851394700683	10.33580112431185
Au	8.26131370692013	8.25864962956514	16.77047071006799
Au	18.57334954298155	16.59348275158264	10.35629011566256
Au	18.55545545605752	12.49449089484735	18.59518776634755
Au	14.51355793359747	14.51316586610661	20.63206469082004
Au	8.35403941415348	10.37898308083782	6.32379554834780
Au	14.55450107819323	10.45135001773672	20.62379233038526

Au	4.48181171733123	14.52706772192665	10.44128020506020
Au	16.52859822043088	18.54609502394286	10.38797725630599
Au	4.38947518966586	10.50125789363765	10.45158503201421
Au	12.49767462283839	18.49356542189965	18.53925741855524
Au	16.57996447546871	10.41150059742177	6.40517992521404
Au	8.32931354063858	14.65354555248111	18.68264136936137
Au	10.40324245367287	16.55076459614270	18.58091076878354
Au	20.59737264683514	10.48774718944975	10.45152062282964
Au	4.24875491448303	12.51200321478283	12.48411192372753
Au	14.67783571616962	18.73091354588271	8.24903359039537
Au	16.53479787927829	14.61366026274326	6.38753557656283
Au	6.49079744104100	18.51023491504647	12.46895196704326
Au	6.44566276217663	12.49003271156860	18.57352134127773
Au	18.70239890211918	10.28937184802974	16.74472508034564
Au	8.28556577563786	6.30126789659128	14.73936372592943
Au	4.45106441831185	10.50422964795216	14.52451065122401
Au	8.42964033013103	8.48375989350006	8.44063136673896
Au	10.50358587309967	4.41737511860807	10.47989068177953
Au	20.66571225218128	12.52723841931454	12.48527777659361
Au	16.55334550737340	16.62896145650292	8.34482189521038
Au	20.56852441289200	14.52581965897143	10.44382686383701
Au	16.60249646349347	8.40172776486796	8.41833133271417
Au	18.48582430008295	12.51149819554140	6.47958630499597
Au	18.59539164982263	10.38179593579469	8.38810129726402
Au	10.47302012114345	4.45349602629753	14.54902526584651
Au	18.71304232352176	14.68667209943721	16.72312112727645
Au	6.33187713679523	14.65101776931218	16.66246023791685
Au	8.28246750660399	16.76235507366197	8.20913393827711
Au	16.71398334962928	10.28517674242678	18.75017053708309
Au	14.51491043576345	20.57735399826303	10.44389913740309
Au	8.30865964231981	14.74065753252238	6.23760618819974
Au	14.67211659325377	16.73078177032303	6.24159698330844
Au	14.75730909360271	8.24780161347805	18.78678980478159
Au	10.26719111060330	6.28498700088783	16.75197959957595
Au	12.51324023023916	12.49741460661082	20.75218287984312
Au	16.69546079287238	14.66610685268644	18.73073633417708
Au	10.51011207182840	10.50510311428012	4.39552019221374
Au	10.32210182581747	18.72387607719642	8.24805678560678
Au	8.41819164099122	16.52600936104952	16.54836630166227
Au	12.50519696469491	18.60754267917267	6.37703580034049
Au	16.50628868842708	12.48857952278067	20.53919376620185
Au	12.49869889262109	8.53039836419121	4.51511646960645
Au	16.43182271914714	12.51583680881688	4.48604839158034
Au	6.49913334625974	10.51907847831042	12.48044139012483
Au	12.50739985681084	8.50563531331481	20.51410004464749
Au	20.50368269869366	16.45222687212026	12.50909299312801
Au	12.50870466433965	20.53658626508006	8.43494026467131
Au	18.46349954248065	14.52814716642836	12.47717890501960
Au	12.50494628143773	16.45880819530565	20.51393436784423
Au	12.50346327277689	4.47104989991078	8.49350876548762
Au	8.47842655400398	12.51909264720543	16.58115622876468
Au	16.43540374681807	20.43979088194503	12.49179553246402
Au	20.49384361377044	12.48953783384238	8.48144368629127
Au	4.49701603262924	12.50854119588938	16.51988526208359
Au	8.53877689141238	20.46203603037667	12.47274976648415
Au	10.52619889553778	6.51707380153183	12.46240726129931
Au	4.53532958705080	16.46865898178342	12.48218539069928
Au	14.50935114714022	6.50392959977043	12.46065030757386
Au	10.51851000739159	18.48485770833285	12.50216989051567
Au	12.51545665369421	8.45658861915051	16.55722009306167
Au	12.50487236891085	16.57261839904992	4.42665452783425
Au	12.50056120297788	20.43544758662435	16.48413675056818

Au	8.51398670760318	12.48556006668644	20.50146918416569
Au	16.55608842790351	12.55713855226307	16.58139092814896
Au	8.49329012521828	12.49999669781137	8.42842798195545
Au	10.45356047176239	10.43603331398871	16.60065900076652
Au	10.45307001086415	14.59770558319451	8.37666270947907
Au	6.50768335411789	14.48915808152285	12.51167117727680
Au	4.53638336483155	12.51134946366125	8.46000874612726
Au	12.53726230906701	16.57281549155361	8.39784568516371
Au	16.47101870535247	4.52416539554806	12.51499324211482
Au	14.59682510332761	10.42140800055122	16.62467670834445
Au	18.48470357133372	10.53528187300943	12.45382555784245
Au	16.53620517185514	8.48729202989966	12.44649326593739
Au	8.53753147116604	4.56240526706531	12.51357783836537
Au	8.50397619948452	16.50887439567725	12.51232861724290
Au	8.48535226047617	8.48189987138642	12.47587689751776
Au	20.44856214344890	8.54620315180392	12.49496905057998
Au	8.52960699070165	12.53353707106147	4.46183584910866
Au	4.52196247578549	8.54762202305932	12.48931357635435
Au	18.46176573210548	12.53091297776307	10.43726005671606
Au	8.44616725457752	10.43876135848246	14.58599796049727
Au	6.50763089196909	12.49678737532729	10.45861222738306
Au	14.51934317472534	12.52921261035427	18.56343202191087
Au	12.51684891525419	6.51679573652894	10.44199288435944
Au	10.46045896924707	16.57389640427127	10.39476650979383
Au	18.49313546619766	12.55773611458281	14.52407835650322
Au	10.52516365545270	12.52425798108554	18.54737633947050
Au	6.49596520471138	12.52028372439422	14.54355400351576
Au	14.59563998857899	8.42654673045586	14.59445320303611
Au	12.53227507063997	18.52694207290823	10.42129015268387
Au	12.53049312502771	14.57509003146174	6.41149399722309
Au	12.52195542910915	10.48060448025535	18.56831180750217
Au	16.58972117484340	10.43149547776043	14.60038054110704
Au	10.45157889893290	8.44472222698032	14.57707109598076
Au	10.50769958185535	12.51026069072098	6.44447604712657
Au	12.51838706621123	6.48571239279798	14.49619419414829
Au	8.44666073651112	14.59129304906552	10.38785088885701

$Pt_{110}Au_{295}L1_1$-1518.215

Pt	12.02145482854823	12.02146740615888	6.00696334485960
Pt	14.02181097516190	10.04499725853099	6.01830439690905
Pt	10.04496984890353	14.02182641728536	6.01829655758491
Pt	16.10142170006305	16.10143473682828	5.96552480392201
Pt	18.08495426986707	14.07320976937060	5.96964421033730
Pt	14.07317073534897	18.08492786290053	5.96965938405808
Pt	11.92542106475028	7.96374932950304	9.99735459702471
Pt	13.97820436375441	6.01850310663542	10.04523071254935
Pt	7.96374261670963	11.92539419088737	9.99730708207783
Pt	9.93790105684972	9.93785653307512	9.98894198252946
Pt	14.02273839016882	11.99355502074473	7.96683092402204
Pt	16.07468171380184	9.99724398967376	7.96378768374784
Pt	16.03251514388093	12.00720214986152	9.95538371213718
Pt	18.06213007635893	9.98892359332715	9.93790535138911
Pt	6.01850733449657	13.97821212859649	10.04521536750305
Pt	9.99724591771077	16.07472491110534	7.96375319664325
Pt	11.99356812358667	14.02270882268141	7.96684751314051
Pt	12.00717226479924	16.03254601789589	9.95541826240795
Pt	14.00832165646680	14.00827959391210	9.92883050849396
Pt	18.10565181781546	16.12244615071721	7.92085242212812
Pt	20.06780679105820	14.10804417093243	7.93218916747251
Pt	20.07909098529649	16.12241879159892	9.89430298954295
Pt	22.03034150801145	14.07315970262685	9.91502549130631
Pt	9.98891566787519	18.06223713633143	9.93788666488459

Pt	14.10786918427196	20.06780160927097	7.93225291560574
Pt	16.12243311206840	18.10558433996087	7.92090190896025
Pt	16.12237256601183	20.07908112919069	9.89439736659468
Pt	18.11938820749598	18.11941269727983	9.88053507891887
Pt	14.07310286613564	22.03035300553297	9.91509576240627
Pt	7.93223880096851	7.93220773212428	14.10811141826286
Pt	9.91508151987154	5.96967823093274	14.07328256990526
Pt	13.97734172624094	7.96668275632637	11.99354434839914
Pt	15.97838617925270	6.00724240299413	12.02165624910730
Pt	16.00655101051429	7.96667746843170	14.02274353881688
Pt	17.95477382039808	6.01853291784668	14.02182254299379
Pt	5.96970986754065	9.91506862262080	14.07324362719831
Pt	9.95535941646496	11.96757348816597	12.00725664158490
Pt	11.96761999080900	9.95531828714776	12.00727205119769
Pt	11.96538190382169	11.96542408870041	14.02049997827539
Pt	13.99176599388058	9.92880073310753	14.00835033054603
Pt	18.04467590382954	12.00721545560669	11.96759139570325
Pt	20.03621791759430	9.99732884217353	11.92542130389293
Pt	20.03323634460857	11.99348838525779	13.97730371059997
Pt	21.98157768806374	10.04508669478336	13.97821164515055
Pt	6.00725146524469	15.97850167847635	12.02159481705416
Pt	7.96670914632864	13.97732840795667	11.99353195266880
Pt	7.96674943887713	16.00661476899012	14.02274778757108
Pt	9.92892440322839	13.99175531554882	14.00833720172036
Pt	14.02042909912781	16.03473901692512	11.96543642215056
Pt	16.03467671307860	14.02041680685824	11.96539086453548
Pt	16.03461636381978	16.03471554129383	13.97961259930260
Pt	18.07117878333173	14.00820844601783	13.99172106319321
Pt	22.03449834640254	16.10137101736070	11.89859386999887
Pt	6.01857383331478	17.95487090499575	14.02184077040621
Pt	9.99730548253089	20.03633665378876	11.92541627730061
Pt	12.00717056092420	18.04471832019986	11.96761143641879
Pt	11.99350088155137	20.03329965280400	13.97733404788823
Pt	14.00822910545939	18.07124770298530	13.99174171786939
Pt	18.10555237099886	20.07908546922875	11.87757240350599
Pt	20.07904993254680	18.10557891437836	11.87753319733886
Pt	20.06779322718006	20.06784217609847	13.89220149916074
Pt	22.03038703877443	18.08489769573959	13.92691361814180
Pt	10.04509229986311	21.98156944796352	13.97822071199412
Pt	16.10130370932631	22.03453897161414	11.89864917761071
Pt	18.08486418661476	22.03040047541666	13.92692904909249
Pt	9.89436443439907	7.92090322564782	16.12252724653111
Pt	11.89854922449578	5.96561595182802	16.10150962172007
Pt	11.87756100315374	7.92093952984883	18.10565367729108
Pt	13.92676031400045	5.96976828966360	18.08491619000472
Pt	18.00258323763557	7.96379575533464	16.07459276537283
Pt	5.96561894148158	11.89858318394649	16.10145814694348
Pt	7.92097418547603	9.89428166191571	16.12252784934269
Pt	7.92100509529360	11.87752464814402	18.10568050639112
Pt	9.88062015868119	9.88057244806459	18.11944627900730
Pt	13.97959805442562	11.96540737033945	16.03469572444941
Pt	15.99280931148356	9.95533196813734	16.03246317796556
Pt	15.99279418601891	11.96753241040468	18.04464590081400
Pt	18.01096710565319	9.93788304683306	18.06207715942751
Pt	21.99287133799792	12.02148638956974	15.97853098213364
Pt	5.96983375892101	13.92680184804001	18.08490594579206
Pt	9.95540902825514	15.99291015222677	16.03250293702292
Pt	11.96540623867756	13.97957948861030	16.03466060993796
Pt	11.96753181130237	15.99288438767131	18.04460444780475
Pt	13.99170927866400	13.99169258572005	18.07108311263785
Pt	18.04461822591492	16.03245269928718	15.99284352045106
Pt	20.03322440204823	14.02264612414355	16.00650559232096

Pt	20.03627822530986	16.07465540822994	18.00273118018599
Pt	21.98159309291289	14.02178720485661	17.95492799773385
Pt	7.96377280210180	18.00271788456891	16.07461980207097
Pt	9.93789458646566	18.01105659974519	18.06211293403237
Pt	14.02264386766343	20.03330803665975	16.00650731606462
Pt	16.03240412669101	18.04469157019024	15.99285366754595
Pt	16.07460829559838	20.03635406160774	18.00274695744930
Pt	18.06215265605037	18.06224072375505	18.01112168148325
Pt	12.02147394502927	21.99288666148589	15.97854641979859
Pt	14.02177666525503	21.98159213723041	17.95492775292396
Pt	13.89190055564242	7.93230123528639	20.06770384202370
Pt	9.89440261052000	11.87754081144723	20.07901738227958
Pt	11.87756663621433	9.89433743725214	20.07903875332807
Pt	11.89858213354491	11.89857075105172	22.03428794415228
Pt	13.92673940355011	9.91508864655436	22.03015432481960
Pt	18.00262760721633	11.92538640806411	20.03624204872541
Pt	7.93228220264419	13.89197011961230	20.06772485916382
Pt	9.91512370304300	13.92677847753696	22.03013038145143
Pt	13.97729283804362	16.00654733786901	20.03326477778487
Pt	16.00650956106743	13.97727990691429	20.03331469488586
Pt	15.97841706715046	15.97846261349970	21.99270414947545
Pt	17.95477990770334	13.97819980295901	21.98146990332779
Pt	11.92537070779408	18.00271679381878	20.03625245360107
Pt	13.97818141449462	17.95484120797921	21.98141202177761
Au	11.76594344885793	7.74437581554838	5.79848986060378
Au	14.00001674785833	5.91209072436596	5.91205058389978
Au	13.98193146750075	8.06519771496160	4.08352319894033
Au	16.13152461663253	7.92548014768315	5.89233256516657
Au	7.74438870438536	11.76594848097443	5.79847072388341
Au	9.71233156461595	9.71232899062544	5.78465044724649
Au	9.98801515496986	11.95625067342490	4.05143554553905
Au	11.95620871569071	9.98804181479325	4.05143870824779
Au	13.99185741940190	11.99673199684037	3.80101173363810
Au	16.03286471056301	9.99199236124224	3.88843779050223
Au	16.06413343250248	11.97042532470616	5.91340495174768
Au	18.23510427342391	9.81493704464687	5.75214474008569
Au	18.05196228865825	11.99931435371173	3.90889261828797
Au	20.17535945520207	11.88989166084609	5.82868465710290
Au	5.91210011157210	13.99998721883751	5.91210277304490
Au	8.06513960141548	13.98198213368150	4.08351482105924
Au	7.92552799040382	16.13152826718234	5.89240627537563
Au	9.99202955409575	16.03286706231913	3.88845253415169
Au	11.99675472025064	13.99183718600699	3.80102167074671
Au	11.97043089715259	16.06410463983262	5.91342538322261
Au	14.01734006559821	14.01732986241225	5.88134204447516
Au	14.04301554682764	16.05351996504622	3.86106747271784
Au	16.05353050951094	14.04298549377012	3.86104622297541
Au	18.05285426401161	16.06614430028006	3.90827388471014
Au	20.00463801994364	14.03181503465966	3.97081588886712
Au	20.18095740786935	16.18074591102040	5.82089896360739
Au	22.06522146786238	14.02810526960223	5.93474264879185
Au	9.81497353558730	18.23507864570559	5.75220923031820
Au	11.99933272854106	18.05195535738134	3.90894843102345
Au	11.88988657684794	20.17532174940940	5.82875696455193
Au	14.03178280285726	20.00463629844627	3.97084234662273
Au	16.06613249847864	18.05283416797724	3.90828368451149
Au	16.18063965214459	20.18086686317192	5.82102064476683
Au	18.20751424555563	18.20751328281464	5.80360897833310
Au	14.02805624363567	22.06520504558560	5.93478680870122
Au	11.96719586085333	3.88853073035994	9.99205243068342
Au	14.01809689481149	4.08364489266866	8.06524387023368
Au	16.04374212991525	4.05159865005345	9.98814256519969

Au	7.72218133850896	7.72215439226628	9.78711952537793
Au	9.76493473281445	5.75216489111138	9.81502802133473
Au	9.82409823455077	7.82852561333251	7.89802232912905
Au	11.86849796981095	5.89238078740982	7.92555159748531
Au	14.00001217920825	7.91998022089631	7.92003593771328
Au	16.23407502995210	5.79851025063355	7.74437791933290
Au	16.09811281317751	7.87860092022760	9.88708994686013
Au	18.28764171377356	5.78467178311252	9.71237355313171
Au	18.17595083678954	7.89799071738646	7.82854753655717
Au	20.17142218809600	7.89804177371626	9.82408115416408
Au	3.88851441873774	11.96714869873500	9.99204862471837
Au	5.75217186224037	9.76490490210822	9.81503113442608
Au	5.89241303371989	11.86847769096972	7.92554907514276
Au	7.82855859435619	9.82408474333461	7.89800807870335
Au	9.88701485770576	11.90189328325272	7.87865372472223
Au	11.90187873984632	9.88699886322796	7.87864746020521
Au	11.95342114558098	11.95342405347431	9.92442352413853
Au	14.00001773954997	9.93518526537416	9.93529421164571
Au	18.12536448962879	11.93045440568919	7.88722778231580
Au	20.27788771709527	9.78702132862245	7.72210530473411
Au	20.11276810799732	11.93044433804675	9.87467405534120
Au	22.24782271359949	9.81498447879356	9.76492185910247
Au	22.17129829301504	11.88987586823597	7.82465389978947
Au	24.09113266027730	11.99931255807865	9.94804392914446
Au	4.08364166858182	14.01809667645720	8.06528315347166
Au	4.05163455057605	16.04376276994576	9.98820163404249
Au	5.79857874529244	16.23405358041484	7.74444297734350
Au	7.91999494955381	14.00000694526969	7.92007447449816
Au	7.87864025088055	16.09813771897333	9.88712707442117
Au	9.93519838430972	14.00006725784322	9.93530252684162
Au	14.01657467663145	16.05763978859599	7.91554435779211
Au	16.05763298457906	14.01656450831251	7.91552687966997
Au	16.05019757875716	16.05023137759049	9.92923102302650
Au	18.07540797510120	14.00685242714472	9.92465056056559
Au	22.17907332512704	16.18073812648488	7.81900088481390
Au	24.02912365965449	14.03178615235280	7.99533271420036
Au	24.09167023472552	16.06609948676284	9.94715371776921
Au	5.78471947574657	18.28766412782048	9.71239229304772
Au	7.89804657470217	18.17594233345124	7.82859495699147
Au	7.89807065262301	20.17143614307749	9.82414633060350
Au	9.78719220578442	20.27772111903970	7.72231419084702
Au	11.93049457384006	18.12536217890447	7.88726681153073
Au	11.93051173972716	20.11279651682318	9.87472461744249
Au	14.00685219996310	18.07545504833035	9.92466431233619
Au	18.21443921068011	20.21824019712377	7.78174814389958
Au	20.21823206544018	18.21446828105532	7.78173690598190
Au	20.21821553719569	20.21825316742482	9.78556293294591
Au	22.19635683990900	18.20748909100438	9.79247343560015
Au	9.81499520532640	22.24776523430197	9.76503075632001
Au	11.88987883674482	22.17123055171084	7.82469207060220
Au	11.99930681176433	24.09104116485305	9.94806038048703
Au	14.03175673145600	24.02912010038936	7.99537965383928
Au	16.18062302735602	22.17896003769828	7.81914651436148
Au	16.06609525999400	24.09167364499075	9.94718769302291
Au	18.20748152740670	22.19638851744982	9.79248434329986
Au	7.99540723393220	3.97091088149329	14.03184433356403
Au	9.94809629232964	3.90887667524262	11.99937171947179
Au	11.94646415060977	3.86113562405805	14.04308032318374
Au	14.00816353001016	3.80113976821414	11.99681196959219
Au	16.00323368861272	3.80117481402689	13.99185965564187
Au	18.01188132692733	4.05159218704967	11.95628894047432
Au	19.93474845502835	4.08366516442578	13.98193379723864

Au	3.97094347425243	7.99539211623013	14.03185260980369
Au	5.93485506221649	5.93482917190313	14.02813313104608
Au	5.82874367892685	7.82467626359448	11.88994140221467
Au	7.82472005719362	5.82870359926048	11.88995581191016
Au	9.87466091256039	7.88722014882185	11.93053554875993
Au	11.93589572692252	5.91342248288803	11.97047056655340
Au	11.94240305595622	7.91553073096070	14.01664051882944
Au	13.98269467476918	5.88141727326738	14.01736318730808
Au	18.11294097544296	7.87862849802604	11.90194573226059
Au	20.25556624025631	5.79856430065123	11.76599747852953
Au	20.07995691912103	7.92002961178500	14.00004093163346
Au	22.08791338960037	5.91213078991298	14.00000811000819
Au	22.10758672781084	7.92554495973976	11.86849956004656
Au	23.91642605242992	8.06523457144624	14.01804172186842
Au	3.90887665416565	9.94808019780599	11.99937803722962
Au	3.86110992506322	11.94648908643376	14.04305332182498
Au	5.91344940604023	11.93588101929034	11.97045577449595
Au	7.88722741243408	9.87462250096304	11.93054170189381
Au	7.91553219057555	11.94236942833760	14.01663782189937
Au	9.92461527092762	9.92459485134638	14.00697689983899
Au	14.00003790672324	11.96784968247269	11.96787660681618
Au	16.04660497491733	9.92432031623293	11.95348081378047
Au	16.03220975686248	11.96783067881037	14.00007158523987
Au	18.06474547974836	9.93521038293260	14.00005913536826
Au	22.08657588926298	11.97038049887885	11.93589630550544
Au	24.11150023937773	9.99201703411226	11.96714593471147
Au	24.19889626606696	11.99668954560129	14.00818739161654
Au	3.80116916540382	14.00812464972314	11.99682360465452
Au	3.80122838328789	16.00320544557475	13.99188230683687
Au	5.88148959113340	13.98270461838110	14.01736179815346
Au	9.92437865228436	16.04669861585337	11.95345617030323
Au	11.96783896362398	14.00008843444658	11.96788375009339
Au	11.96782933361486	16.03227003771665	14.00006385521214
Au	13.99999713231911	14.00006240788407	14.00008863788386
Au	18.07075691182507	16.05017467928828	11.94986563889495
Au	20.08447577657768	14.01651133533461	11.94240919934635
Au	20.08443787291348	16.05755605550979	13.98348195272245
Au	22.11860477426452	14.01724289713048	13.98273522852904
Au	24.13892242329478	14.04294627251002	11.94649006655714
Au	24.13891982860452	16.05344606755613	13.95701753869115
Au	4.05165799766000	18.01191045525018	11.95633167460938
Au	4.08370091580865	19.93478994272569	13.98200098945444
Au	5.79859876418226	20.25558006564870	11.76601053597222
Au	7.87866787900401	18.11298628239273	11.90197353667119
Au	7.92007741887445	20.07997273513260	14.00003217597460
Au	9.93524021636743	18.06482960628058	14.00004957858888
Au	14.01653619641684	20.08455753062931	11.94242126105210
Au	16.05011811088684	18.07082692578879	11.94986694681660
Au	16.05753285423516	20.08453780345289	13.98347663243404
Au	18.07534124502273	18.07540235267651	13.99319461767099
Au	22.17904184219779	20.18091993136400	11.81925668806951
Au	24.09168643436946	18.05277061103434	11.93388632681834
Au	24.02910459766962	20.00458433868974	13.96824288016109
Au	5.91218073732742	22.08791065108402	14.00002840569277
Au	7.92556080624128	22.10757637991339	11.86857230366314
Au	8.06528283112394	23.91641070521172	14.01811668400401
Au	9.99204263652334	24.11148539969836	11.96717864113512
Au	11.97040307981824	22.08658965844780	11.93597053462731
Au	11.99672674265686	24.19885935285070	14.00818269987362
Au	14.01725695271773	22.11866742712620	13.98273821875883
Au	14.04296735205021	24.13886367757076	11.94650058090630
Au	16.05345197166904	24.13888004903552	13.95703244889694

Au	18.05277847678029	24.09169557978378	11.93388523769651
Au	20.18089208403530	22.17903596161298	11.81927780225218
Au	20.00455857295814	24.02912241862067	13.96823572615935
Au	22.06515621449567	22.06515808985188	13.97195009482050
Au	9.94717193542925	3.90837427488244	16.06616183940630
Au	11.93386836372520	3.90836911597312	18.05281363993043
Au	13.95695841637631	3.86114900118494	16.05356240395729
Au	16.00062396220527	3.90894243700027	18.05187669762100
Au	18.00795086064453	3.88857275444217	16.03280202776767
Au	5.82097657856760	7.81902673431078	16.18079506983004
Au	7.81906215016028	5.82094383991848	16.18078464812153
Au	7.78179248926929	7.78176144288262	18.21446215715165
Au	9.79250323085084	5.80366271616190	18.20750708315392
Au	13.98343423952474	7.91555211756949	16.05765031905671
Au	16.02955899031548	5.91344942066903	16.06411678229464
Au	16.06948640582057	7.88725328146948	18.12536115892707
Au	18.18495543874309	5.75222460760137	18.23503084934033
Au	20.07440963904766	5.89243526157114	16.13150604437042
Au	20.10190369521590	7.82857062529394	18.17586406235130
Au	22.20144799926886	7.74443918225857	16.23399644125054
Au	3.90839405507606	9.94716860173399	16.06613895661062
Au	3.90840531574521	11.93385001961579	18.05279636343831
Au	5.80369912487325	9.79247186722199	18.20750913250356
Au	9.92928332179043	11.94984851433989	16.05024086452326
Au	11.94982120866946	9.92922342452512	16.05025079681617
Au	11.94983294724902	11.94984844447388	18.07078795600175
Au	13.99308895140947	9.92462128585964	18.07543861713848
Au	18.07560956638230	11.95341053506522	16.04662601372076
Au	20.12131890434162	9.88702298649799	16.09813060439534
Au	20.12131173892029	11.90188035289853	18.11297534350271
Au	22.21527431307801	9.71234015679280	18.28764081797760
Au	23.94843809282337	9.98804593364422	16.04374946997663
Au	23.94846089441203	11.95620703357899	18.01193593254826
Au	3.86115805809156	13.95696483358317	16.05354714109999
Au	3.90893076729016	16.00071003676463	18.05190459384578
Au	5.91353256609781	16.02955516128955	16.06410221455102
Au	7.91559131558903	13.98343406982536	16.05765740171158
Au	7.88726720501315	16.06948297261691	18.12538258214403
Au	9.92465152463490	13.99313915011197	18.07541546622653
Au	13.99999100857928	16.03224662789813	16.03221798764630
Au	16.03217259428136	14.00003869323301	16.03222794010653
Au	16.04658040041534	16.04664559472209	18.07563931713710
Au	18.06471410591794	14.00002152250068	18.06480368757736
Au	22.08655859385298	16.06405224088791	16.02960539025118
Au	24.19889034561640	13.99175650136879	16.00327468434948
Au	24.11148213725204	16.03281838463326	18.00795399715227
Au	3.88853893725578	18.00787652754391	16.03281158182417
Au	5.75237200609194	18.18489117215163	18.23493834739617
Au	5.89257962640884	20.07441399955090	16.13150163987478
Au	7.82867827468420	20.10191935906874	18.17583543340494
Au	9.88707289823466	20.12133903793824	16.09810692743122
Au	11.95339038633564	18.07567907353742	16.04664278517020
Au	11.90189116919463	20.12134003287935	18.11295437307963
Au	13.99997734104863	18.06480404130566	18.06478809547864
Au	18.12527248610360	20.11278861582601	16.06954743017755
Au	20.11270567453016	18.12531533195332	16.06956218829133
Au	20.27766682336728	20.27770866976402	18.21284001484973
Au	22.24776097044143	18.23500379418940	18.18498599393940
Au	22.17122614535990	20.17525968826266	16.11015134431807
Au	24.09114468430394	18.05189813133709	16.00069582077163
Au	7.74447818841080	22.20143129287098	16.23405405479085
Au	9.71236205297111	22.21529896207788	18.28764846856597

Au	9.98812974407194	23.94843125274316	16.04378464589628
Au	11.95620691380615	23.94844127685070	18.01188872497588
Au	13.99178640792385	24.19885685173823	16.00326670909909
Au	16.06399969772761	22.08657886127665	16.02960617905469
Au	16.03280249092909	24.11149727004071	18.00796224469304
Au	18.23497354268780	22.24779122957376	18.18503740820881
Au	18.05189752577430	24.09106491415938	16.00071415403081
Au	20.17527823058376	22.17123441352349	16.11016458675146
Au	13.96819876691893	3.97094715379546	20.00452093341529
Au	9.78556958473848	7.78177080532595	20.21817975506666
Au	11.81933814828115	5.82107782253155	20.18078597330607
Au	11.81924705028061	7.81908344415033	22.17894849923780
Au	13.97192146503779	5.93489783983636	22.06505700243872
Au	16.11004153237210	5.82878517192155	20.17520439645495
Au	16.11004885842454	7.82474144937819	22.17114342050973
Au	18.21285586956621	7.72220579308425	20.27775693592196
Au	5.82101075623056	11.81920112562691	20.18088839440941
Au	7.78181332885991	9.78554451424077	20.21817238012529
Au	7.81910496983040	11.81921422474707	22.17894907894447
Au	9.79253263564759	9.79250774112692	22.19624705167426
Au	13.98338624040576	11.94235727790892	20.08446669671613
Au	16.06945871866289	9.87462828635845	20.11275182159018
Au	16.02953289964760	11.93586578736915	22.08652265506390
Au	18.18494591565157	9.76493019370349	22.24776615840484
Au	20.10190836626396	9.82410821650005	20.17140436868224
Au	20.07438871755699	11.86848073910564	22.10753371274020
Au	22.20141905230063	11.76596467950677	20.25556169260458
Au	3.97101765593836	13.96817037218307	20.00447104637633
Au	5.93495356826928	13.97191076464023	22.06501117779179
Au	5.82877820983417	16.11014686562844	20.17525308812691
Au	7.82472870349368	16.11015069151504	22.17119955919912
Au	9.87463835739188	16.06946514701589	20.11274747459278
Au	11.94237862863256	13.98339698853905	20.08442601206972
Au	11.93590635406141	16.02951237908037	22.08645465637123
Au	13.98265643098437	13.98265647379582	22.11852322353367
Au	18.11289411528139	16.09810490138332	20.12134258428106
Au	20.07991361562904	13.99998307996957	20.07998855181237
Au	20.25553353811305	16.23402773425025	22.20140131433793
Au	22.08786063095037	13.99997057492422	22.08787694998440
Au	22.10751244229878	16.13148595606914	20.07444468212352
Au	23.91638857158469	13.98195621755620	19.93481938612831
Au	7.72229552363228	18.21284696472931	20.27770142674275
Au	9.76506565202822	18.18489026409934	22.24761891522894
Au	9.82418004971662	20.10191366998421	20.17133684521308
Au	11.86853237016134	20.07439853816661	22.10743864912694
Au	13.99998739375093	20.07996911173517	20.07995155320753
Au	16.09805666955865	18.11296773643086	20.12132651722098
Au	16.23405042199215	20.25559163865731	22.20138607888813
Au	18.28762496838705	18.28765659495269	22.21527543559875
Au	18.17585920841355	20.17143467482385	20.10194758932629
Au	20.17138329506640	18.17590427389521	20.10194514524347
Au	11.76593814973035	22.20141208705693	20.25555066268679
Au	13.99998109899771	22.08789594560587	22.08785239060127
Au	13.98189405750879	23.91638925361182	19.93475558305473
Au	16.13143943962213	22.10756653326541	20.07445617837356
Au	13.96817260198381	7.99545116459998	24.02892977438368
Au	9.94722529243592	11.93386379321248	24.09151458025197
Au	11.93387741262297	9.94719155602534	24.09150678019799
Au	13.95696512706382	11.94644322584051	24.13876013652915
Au	16.00059871525094	9.94806313927116	24.09104698250698
Au	18.00787260971592	11.96713980005110	24.11139013294425
Au	7.99550206743285	13.96817488539880	24.02892336901733

Au	9.94807083931164	16.00068187264397	24.09102108090142
Au	11.94647184409334	13.95694139151119	24.13874551336239
Au	14.00813817616197	16.00317531550721	24.19874798185019
Au	16.00317793786643	14.00811398104399	24.19876429242466
Au	18.01181280824160	16.04374210654319	23.94835595501728
Au	19.93468676348228	14.01806809471656	23.91634088708198
Au	11.96719159315085	18.00780282155064	24.11140856633133
Au	14.01805330177789	19.93472030478534	23.91627674589711
Au	16.04373499276646	18.01184230442126	23.94833141079950

$Pt_{110}Au_{295}$LEH-1523.297

Pt	12.01557247594637	11.97850151069774	6.02382217592768
Pt	13.99208476646364	10.03376553986844	6.07313866797044
Pt	15.97461658281472	11.97109768814916	6.02541543323917
Pt	10.04428786444907	13.95479917586184	6.04424623216977
Pt	12.00319932060258	15.91135913008137	6.02201053333605
Pt	13.98820211254693	13.95259054009933	5.98673001564860
Pt	15.96870204113149	15.90677541344670	6.03284835688435
Pt	17.93317320576516	13.94472223715567	6.05937677544037
Pt	11.96699270010701	7.99283152975979	9.95424405958639
Pt	14.00792608746841	6.07394841433038	10.01350419959053
Pt	14.00825107560098	8.03595003521850	8.02403018488496
Pt	16.05180775162031	7.98193438944742	9.93445171627582
Pt	8.03741575591877	11.95215404386768	9.96178400279261
Pt	10.01669765536706	9.97766315204120	9.95853392751461
Pt	9.98875384013153	11.93445893565264	7.97856835227504
Pt	11.95832098075826	9.96952840633308	7.98997568192958
Pt	12.00901310314483	11.98522458524228	9.97906228608677
Pt	13.99102083911617	9.99481088171838	9.97986475367482
Pt	13.99551323202496	11.96337378233996	8.00368701607497
Pt	16.04171805153919	9.95334531401854	7.98257220527525
Pt	15.97478313579322	11.96764688499639	9.96854236433762
Pt	17.98790335658980	9.94666276828578	9.93812097172599
Pt	18.00008363967168	11.91682242724241	7.97483903431576
Pt	19.95741476405557	11.90708981160176	9.94566350942598
Pt	8.06347188880489	13.96539630094624	8.03040975840790
Pt	8.02909719120698	15.97931226018645	9.95625771430797
Pt	10.02953628588073	13.96871199633651	9.97557316590266
Pt	9.99612142402054	15.99538362730920	7.97908496546387
Pt	11.98714049695359	13.95647839525849	7.99755427708417
Pt	12.01474114404861	15.95182592307835	9.97679077335903
Pt	13.98914776040620	13.94742062641626	9.96023826840826
Pt	13.99060209658713	15.95065560507574	8.01276784581453
Pt	15.98256432061851	13.95429847852201	8.00286787751037
Pt	15.97664709073909	15.94681006119134	9.97428111846889
Pt	17.95770737027669	13.94936202770626	9.97732977529486
Pt	17.98815847442162	15.96768089635104	8.00135215627527
Pt	19.93144674022769	13.92882710253418	8.02671896893268
Pt	19.95767507645023	15.94615337204173	9.97071249510132
Pt	10.00371798738188	17.96574907255239	9.95556720840491
Pt	11.96148023777886	17.94825911967618	8.00409918211193
Pt	13.97798290333707	17.95950108954451	9.98373909973171
Pt	10.01601747808244	8.01491594947074	11.92557115752990
Pt	12.02973062854357	6.01772495444650	11.95377056469427
Pt	12.00770854908020	8.00380619298726	13.95321081971550
Pt	14.01029975455131	6.00435304859995	13.92650714277855
Pt	13.99355761451348	8.01538035655158	11.94513842803874
Pt	15.99436300312184	6.03380643146928	11.94853914439680
Pt	15.98128577407362	8.02142903262913	13.93120910565515
Pt	17.92398613984257	6.06885506981410	13.94184965158913
Pt	18.00406398308194	7.97446998909893	11.90119860770896
Pt	19.91310428192465	8.03789730205756	13.92789717230843

Pt	8.05226061832330	10.00162842115770	11.92327973399659
Pt	8.03566467196168	11.99036632043818	13.93881399755188
Pt	10.02621046805593	9.99079476796045	13.97476370721879
Pt	10.02184396343242	11.97879816409746	11.97041222341744
Pt	12.01071117625424	9.99648602069107	11.96780630769665
Pt	12.00724773501480	11.99303469134297	13.94379597400647
Pt	13.99144410670649	9.97634404330738	13.93858150380417
Pt	13.99246597047923	11.97482005769413	11.95570901767406
Pt	15.97398902221861	9.97514310455945	11.93974657306515
Pt	15.97529730079642	11.97120352680328	13.94487367730956
Pt	17.94312453954691	9.99257457807404	13.92344741142068
Pt	17.96969741579026	11.96229657063389	11.94180986538093
Pt	19.95005374032596	9.95189167950131	11.89950247329599
Pt	19.95083253834094	11.94913324461801	13.92250563972765
Pt	8.03256339643298	13.96661479328853	11.93650635515332
Pt	8.05228700714421	15.93776941027130	13.92139387346688
Pt	10.00257925034245	13.96910108013432	13.96411387694982
Pt	10.02559610807698	15.96574117866514	11.95294402080346
Pt	11.99391786687816	13.97307270698678	11.95439018000071
Pt	12.00657910572473	15.94786640693528	13.94889882984706
Pt	13.98899520325798	13.95755687830563	13.94173024302257
Pt	13.99690675806440	15.94710517764186	11.96194181059996
Pt	15.98757177780724	13.94474063964200	11.96843052296576
Pt	15.96772744488646	15.95940555737932	13.95387574461976
Pt	17.98772368912200	13.94899381154285	13.95896214737633
Pt	17.96014973213604	15.94450990896806	11.95481177002725
Pt	19.95545805004186	13.93110314224690	11.94866282262405
Pt	8.03523203253764	17.93920256214577	11.91422050756944
Pt	10.02917340709967	17.94174155222296	13.93644968629285
Pt	11.99925475286636	17.98928641121190	11.94278510394585
Pt	11.98311999695941	19.99064132563227	13.92947976449697
Pt	13.95257543390924	17.99480909354137	13.92401888652913
Pt	13.97245129229441	19.99530528890741	11.94574423024164
Pt	15.96416924414199	17.96572636254634	11.95402880550431
Pt	13.98703823707591	8.01973443797356	15.94862977336695
Pt	15.96901800710967	6.03402213523178	15.91023953384577
Pt	15.99126683492582	8.03434905868298	17.94485332178203
Pt	17.95692276922215	8.01912484271176	15.96602512038642
Pt	10.01364474913963	11.96445140327846	15.96166136529841
Pt	11.99151300198818	9.98023653106156	15.95272550940480
Pt	11.98801080787685	11.94867203470411	17.99820066212652
Pt	13.95855255837462	9.99367924024574	17.94372802739406
Pt	13.96884519898036	11.99195148340191	15.94466898419360
Pt	15.95384776695112	10.00331231685195	15.93260637322535
Pt	15.95433588858763	11.97375483821697	17.95057592091060
Pt	17.94828982806818	9.98485822698442	17.94177274003779
Pt	17.95582627586373	11.96324932390995	15.94886320554858
Pt	19.93970978841093	9.98126192875030	15.93676548842052
Pt	8.03825697584895	13.96208222992560	15.92874677449789
Pt	10.02107464336168	13.93871317579969	17.96739644096309
Pt	10.02450164082081	15.94467239065106	15.96069893319199
Pt	12.00747888689478	13.95139035384509	15.97025549143558
Pt	11.99520249631969	15.93708063554545	17.96262990198069
Pt	13.97782862259995	13.94337491526486	18.00534053240191
Pt	13.97043122242250	15.95209648051934	15.95507524603643
Pt	15.97109524420713	13.97440055175617	15.95768487197670
Pt	11.98284572759794	17.95262070370017	15.93607194578808
Pt	13.97569267748252	11.95456358693727	19.98629052306565
Pt	11.99140033882272	13.93906933498445	20.00312534831944
Au	11.88826440442437	7.90839663940725	5.92516018979707
Au	13.99637254353938	5.99995561824719	5.99680090409936
Au	13.98933561237979	8.08640202161804	4.04936424888528

Au	16.10778650382307	7.90122795335604	5.90129693745971
Au	7.89294966830221	11.89399394485068	5.93564229476425
Au	9.87032026764663	9.87394281387019	5.90478899907757
Au	10.00402004185196	11.97189343100820	3.97480243713839
Au	11.97083453471102	10.00706416193911	3.97032843656163
Au	13.99458869338491	11.97690722357246	3.86469689702816
Au	16.01258962604119	10.00483232494382	3.96272379834152
Au	18.12612736306142	9.86329379874297	5.87981385988250
Au	17.97560761637958	11.97053413549976	3.97247165140311
Au	20.09567297745663	11.88050686659694	5.92156689001119
Au	5.98273890283503	13.99936856971873	6.03419702510923
Au	8.06268475055005	13.98839083388772	4.05333622320383
Au	7.92748652644199	16.07104567466382	5.95054402879649
Au	10.02741693463904	15.97989014362085	3.98064866618078
Au	12.01798659669813	13.95804511000603	3.86637271983649
Au	14.00055773751846	15.93240125059460	3.85727055009558
Au	15.96758553123391	13.94925515731126	3.86727533288608
Au	17.93943092297989	15.98814602185779	4.01373397122949
Au	19.90778623538690	13.99790958700241	4.07234761087463
Au	20.03778566683674	16.06287383581548	5.99273456355675
Au	22.00233342873208	13.99405547610880	6.04405159050918
Au	9.93484448968588	18.07958553908690	5.93009025856802
Au	11.97993237598897	17.95782773771652	3.94758054614564
Au	11.83269552579508	20.15856036959412	5.84722609437809
Au	13.95714768765992	17.94345360734829	5.99367357855403
Au	13.99564755964010	19.96434687170257	4.03214027043306
Au	16.00078312260515	17.97919913712340	4.00888223236189
Au	16.19319241268294	20.21842245920067	5.80943802016534
Au	18.10428991276495	18.12241723786846	5.93080086423849
Au	13.99039828714147	22.05385545252069	5.95621339663211
Au	11.97299067620417	3.95845642727210	10.00010184981128
Au	13.99681063206607	4.04604419972039	8.07838025273091
Au	16.03172400298694	3.96751878631372	9.98547110087189
Au	7.87557816113274	7.88820941460353	9.92351596601347
Au	9.87436258556817	5.89880543264071	9.91261996498558
Au	9.85869876181594	7.87536757846343	7.89486622852950
Au	11.89458397273269	5.92264127595612	7.91795305235750
Au	16.11629996141955	5.90532022207067	7.88119274445409
Au	18.13859578881717	5.88127721708923	9.85161911487274
Au	18.15821587476060	7.84948685598310	7.84251679726047
Au	20.14642498229037	7.87054141057122	9.85188327081434
Au	3.83133542713393	11.93681905479172	9.94470927147763
Au	5.84371642796981	9.85384730629657	9.87463044223224
Au	5.85908933435176	11.86167504588571	7.89891223763347
Au	7.87073704847077	9.87691790282257	7.91198374721513
Au	20.14255766617996	9.85353999446598	7.86803208796531
Au	22.17165740532160	9.84162294838792	9.84295114908956
Au	22.14569919104096	11.84368860726906	7.88210421645680
Au	24.15605825830693	11.92767862201885	9.94795023987861
Au	3.93160402092879	13.99018194355311	8.00454223833230
Au	3.85087854270133	16.03549677558838	9.94602990517533
Au	5.97174535459381	13.97637431683940	9.92605308922009
Au	5.86656909782737	16.12321836984326	7.89145982069465
Au	22.01218436647865	13.94003589468555	9.94800415990171
Au	22.08066098655895	16.09669090579845	7.95174846801820
Au	24.04724016752223	13.99237118667955	8.02310711310243
Au	24.16371922730305	16.03188449510816	9.95880435060833
Au	5.88755923986098	18.10558224876517	9.88404941701534
Au	7.91347051377511	18.08752338048209	7.92121496604592
Au	7.87951411024775	20.13755478241251	9.86877353712824
Au	9.87138182338533	20.14613393487497	7.86456556125336
Au	11.93516398558881	20.05981771488805	9.89246932392882

Au	13.97146848333477	20.01950999430645	7.92530941140792
Au	16.02922541007217	18.03252002094498	7.94065619341808
Au	16.03109970507900	20.07130064076474	9.92966764870024
Au	18.02945286082448	18.03470784644640	9.94529733356732
Au	18.20746146776977	20.24546468115954	7.80472510648701
Au	20.11212705088376	18.13040236878008	7.92641560657758
Au	20.26750792025639	20.28800672226360	9.75847051318454
Au	22.20806990626383	18.18940419819155	9.81669291208391
Au	9.76340597613962	22.27023438459429	9.74931943391843
Au	11.75810386090001	22.26645088736574	7.75405947942287
Au	11.93865402775091	24.14306308367385	9.94060902091275
Au	13.98263653867124	22.06237611840517	9.95951921242547
Au	13.99678315817102	24.01395162255698	7.99861749780436
Au	16.20899651427828	22.23158032720264	7.79310803950309
Au	16.05103912745886	24.16137172806799	9.93582277143492
Au	18.23737555128416	22.28398856922528	9.76336117139534
Au	8.01674622134513	4.00223412348570	13.97843424773925
Au	9.99074297084675	3.91243540806129	11.95135514006877
Au	12.04893957520252	3.82736152660258	13.93492361823052
Au	14.01348692122921	3.88063434664697	11.95350014517830
Au	15.97736305285347	3.88403671258164	13.94498241211877
Au	17.99117589945167	3.97235852457826	11.96257019404611
Au	19.90755771920682	4.08170748628908	14.00058367188464
Au	3.99312785129552	8.00740906542512	13.98255615920291
Au	5.94635947291296	5.94710027025360	13.98968570819257
Au	5.78194635650943	7.80657459555583	11.79975201975334
Au	7.77901651956635	5.77956062555709	11.79016248719697
Au	7.96289869061626	7.95294530825434	13.88651800764593
Au	9.99996194039539	5.97850800185152	13.90867885536338
Au	20.10121866178959	5.92419519807905	11.88289515882335
Au	21.99774212596997	6.04797976955585	14.00287330010638
Au	22.14087820814931	7.88497712014033	11.85082723491228
Au	24.04276830161257	8.02153011316703	13.99785494290298
Au	3.83305339491404	9.94820740829385	11.92561948245559
Au	3.70806522340847	11.98737402700082	13.94482022028288
Au	5.97319298378008	9.97926127426739	13.90675286808717
Au	5.93551479610639	11.95844462629283	11.91208989907429
Au	22.00350821230728	9.94417373572576	13.94484043309941
Au	22.04626623413533	11.91740689524718	11.91850651488694
Au	24.15503268477325	9.95192112123583	11.94019913749860
Au	24.29989304004734	11.94856622868883	13.96287816279405
Au	3.71792439656002	13.97865810881889	11.93954926113162
Au	3.70927019368806	15.96647177128265	13.95222725811742
Au	5.89278810231226	13.96476102911472	13.91835228090433
Au	5.93203323153639	15.97280911451865	11.92176565832468
Au	20.04787196148705	15.97169957905049	13.92766695970604
Au	22.13601630685756	13.91354011797295	13.91463700917739
Au	22.09213957861877	15.96740884874731	11.89958113791516
Au	24.29441107665958	13.95399910264282	11.94748807408893
Au	24.25114674682368	15.97025453867816	13.95330753652544
Au	3.87518187761822	18.01525338417859	11.93949624360796
Au	4.03721977169900	19.96260002708563	13.98576597885877
Au	5.99271055601048	17.95801244082759	13.91609770539292
Au	5.85704883727282	20.14866133500433	11.81642914031086
Au	7.96134206358531	19.99735381505845	13.92845352680147
Au	9.93799319295267	20.04962306165112	11.89836261034196
Au	16.00385892743448	20.05620383754317	13.92124271771898
Au	18.03932592405357	18.02959668904990	13.91124222481665
Au	18.10408849595321	20.12741018993231	11.86157031756953
Au	20.10401731540531	18.05612672040544	11.85351051669087
Au	20.09476433860687	20.07149215033231	13.94839659168065
Au	22.07075739966281	18.00986310964793	13.95194266231840

Au	22.27114032931663	20.26319177668337	11.75019612260967
Au	24.12127410525467	18.04812202713874	11.93182086206032
Au	24.00040233444019	20.00235752661369	13.99071353773902
Au	5.97450409338216	22.03564847442184	13.98601648937518
Au	7.77487772960134	22.25475147997990	11.75502852115234
Au	8.00815883293265	24.00526554546748	13.98666170706221
Au	9.97795178436797	22.05426265569892	13.95122413442896
Au	9.95411101174780	24.14054146584221	11.92791345674393
Au	11.96274846659371	22.10247759292268	11.93679753548175
Au	11.97517467918260	24.36533740701937	13.96376457440820
Au	13.96003452941857	22.17068081720577	13.92158985456514
Au	13.98259778309849	24.36964738068421	11.95152226031880
Au	16.00134013933786	22.14943677249942	11.93880421676779
Au	15.99782937502073	24.30435883736517	13.97217013915161
Au	18.04795140540243	22.08377355528086	13.96576491306546
Au	18.06168484304270	24.14500162437028	11.93404518363300
Au	20.26690692714838	22.28258348787852	11.75589010532876
Au	20.02392746425515	24.02346925445931	13.99194019212719
Au	22.07465641762034	22.07376966716417	13.99505633559519
Au	10.00365999828747	3.92839908070832	15.98987863728724
Au	11.99343859398950	3.92810661993786	17.99651450956291
Au	14.03272881918107	3.81879807618541	15.93847708695802
Au	16.02866930216632	3.95683140325435	17.98745689779479
Au	17.95276248044979	4.01372881212508	15.99270625349192
Au	5.80215662743182	7.79341792607246	16.17263677380010
Au	7.81350597649268	5.80287999330569	16.16236077965607
Au	7.79741439656838	7.78663814324718	18.18289123691483
Au	9.82577553770277	5.80382694151683	18.16549697002521
Au	9.93310810049054	7.91046922773033	16.00666876978723
Au	12.02546991036787	5.93210867351913	15.94802507586508
Au	11.94529434206195	7.91047368380313	18.03684588549653
Au	14.04654983082700	5.98117236100487	17.97436857226028
Au	18.03347282864198	5.99200328665527	18.07352850518507
Au	20.02767343449637	6.01023031908756	16.06108838740395
Au	20.05293396998379	8.01791641640148	18.07368153795571
Au	22.08472097618116	7.94975970070143	16.10562876120778
Au	3.92003341610184	9.96831731510606	16.00220605653161
Au	3.91893328910590	11.94643665187710	18.00527999806445
Au	5.80173653525347	9.79411490972438	18.18062100131567
Au	5.92368639061348	11.98355946998570	15.93477031745510
Au	7.92679937084397	9.92388590295026	15.99693504163874
Au	7.92941491954892	11.93147792842389	18.02655197336314
Au	9.93321850040297	9.91540085579415	18.03715174915732
Au	20.07295042196516	11.83203845536358	18.04291828999262
Au	22.22770506497530	9.81853791025384	18.19476590979283
Au	22.08625025989084	11.89480316847035	15.96540002471133
Au	24.16584799579476	9.95883380733625	16.03195556795805
Au	24.12380700221087	11.93851652651218	18.04058185102911
Au	3.70075885602064	13.97982716264025	15.95642201861268
Au	3.93958609951737	16.01938584697217	17.99392957344905
Au	5.97150086028004	13.97906874181293	17.94801145918639
Au	5.94746353668798	15.96083620784991	15.92977761199026
Au	7.96943092576324	16.00047959211175	18.01757628991022
Au	15.99799831750394	15.99013562367879	18.02466773330913
Au	18.03634770576705	13.93535417136427	18.02810731446230
Au	18.02771980258671	15.99375041551719	15.98232147402180
Au	20.04774864798731	13.92696173305610	15.97187736523588
Au	20.12051980781075	16.04833071852601	18.08467072975485
Au	22.06105578762006	13.93417449054238	18.01212678278018
Au	22.11302307643566	15.97124910732008	15.97772494291878
Au	24.25621779444132	13.95404404846893	15.97116565795218
Au	24.09317685555783	16.02718579368054	18.02387339479961

Au	3.94870540523651	17.99455259312249	15.99441422132610
Au	5.85179633806903	18.15661607932432	18.15821603465682
Au	5.83092131747172	20.17540658073362	16.17450774562711
Au	7.96945804773746	18.00919550740455	15.99316743760941
Au	7.84897865877642	20.16589276910268	18.16046516455816
Au	9.98282006812707	18.02649085737060	18.03183533703216
Au	9.97039934982436	20.04786803795600	15.99024623827756
Au	11.90574439407795	20.11498149705178	18.07642825003146
Au	13.93718221617194	18.02883662085317	18.03006892329028
Au	13.95237719163543	20.04705277874217	15.97080110858876
Au	15.98665417865057	18.01829922313896	15.96916636781200
Au	16.06765690441437	20.10467934321219	18.07851227874466
Au	18.11097657988369	18.09580148770768	18.09596762187003
Au	18.09451796668978	20.10657905191945	16.04621035520632
Au	20.11875913093904	18.08095996215076	16.04847549904164
Au	20.24722948050703	20.24446771312175	18.22380882114148
Au	22.23724370882069	18.22069030295329	18.22029452856808
Au	22.25588864924317	20.25029750694396	16.22810790631720
Au	24.08737265309833	18.02721322967260	16.03410410704660
Au	7.82036077135336	22.18667039474142	16.18009587166443
Au	9.79489278277076	22.23064228774557	18.20308523818196
Au	9.95323732468859	24.13233189841236	16.02907510861461
Au	11.96888431975370	22.13509971254872	15.95748440111888
Au	11.95154067646670	24.10890070377699	18.03442225751620
Au	13.98925639571180	22.07318919230016	18.01777959332660
Au	13.98875811655446	24.30470678670054	15.98003489005521
Au	16.00374702935298	22.12249862133139	15.97906881772283
Au	16.04888138601947	24.10336431462122	18.03657704693725
Au	18.22756555023709	22.23827477179176	18.22113275257196
Au	18.05273850591189	24.10849493746143	16.03980471108554
Au	20.25144111634058	22.25012308058537	16.22294689609247
Au	14.01322268546190	4.02268962455055	19.98024779843535
Au	9.79851618330222	7.77793256073599	20.20882752827208
Au	11.80814893956415	5.78588182218393	20.19966257916829
Au	11.79223413480888	7.77505555640512	22.21972940307831
Au	14.00772026785189	5.95839972773711	22.05336989503525
Au	14.02967806654002	7.93551941442605	20.02568166086720
Au	16.17927773175532	5.86241086957693	20.18474046228553
Au	16.24476460956004	7.77277152836820	22.27461536520690
Au	18.10559553223747	7.92738077503252	20.13788879307558
Au	5.77901640771288	11.75895151111790	20.22079843174394
Au	7.79076045371429	9.78628487348916	20.20717294563435
Au	7.76901015172523	11.76071608439588	22.24275599420119
Au	9.79195237135420	9.77824313920998	22.22569486948692
Au	9.92341159874947	11.91892710075988	20.07822738893849
Au	11.94404366569179	9.90076210643802	20.07616685426501
Au	11.98565606803840	11.95768560378711	22.11399217581729
Au	14.00817782746218	9.96874940472260	22.06112140198060
Au	16.02587196613850	9.94759500775078	20.04095119333155
Au	16.01845351566478	11.93476532611919	22.14267426590412
Au	18.24025789115717	9.76403765695519	22.29769656854044
Au	18.08912133441116	11.84820598973239	20.09002719064562
Au	20.22045326673811	9.80642329970040	20.24563487577096
Au	20.25444625868460	11.76706900158484	22.27728647912159
Au	22.25759390151809	11.75995173029995	20.25520710219174
Au	4.05281121517090	13.98937974433200	19.96162960080252
Au	5.96696446699580	13.98950116954229	22.04404176608661
Au	5.81256222407421	16.20329515294303	20.20133474457960
Au	7.95012856082967	13.97949910276349	20.04045320773261
Au	7.79610643804994	16.20773291750725	22.22804845447326
Au	9.98965110175542	13.97384162358854	22.07185628395377
Au	9.97813311659870	16.00337843464345	20.06336368644294

Au	11.97887449885231	15.96886062860637	22.15158314700189
Au	13.97532867248971	13.93142402561666	22.17881950397913
Au	13.95731212558220	15.98249714391944	20.05850036805079
Au	16.01144726548159	13.92772048152765	20.06301356230811
Au	16.01123761658720	15.98035752749087	22.13109055526674
Au	18.05837456693970	13.95147516473045	22.07994788764764
Au	18.10008007196841	16.04975769609318	20.11687659751193
Au	20.08833700730888	13.93544194329691	20.06474382009199
Au	20.24538277347645	16.21804630683739	22.24445389014492
Au	22.07167382674599	13.99834733005073	22.07120339831266
Au	22.25448687646584	16.22836836248062	20.24360373870063
Au	23.99933550508126	13.99741646067077	19.99938824146432
Au	7.84468954977906	18.16252035139972	20.17967638096904
Au	9.79087266068041	18.20564042343312	22.24807391783713
Au	9.79089281627643	20.22906533211192	20.23461061060014
Au	11.90503886719899	18.08485544644669	20.12756375833477
Au	11.76277668853323	20.25644061422097	22.26719969908082
Au	13.99338100108250	18.02171587156279	22.08374200320447
Au	13.99347109145949	20.08893820980753	20.09040163516458
Au	16.07189882685266	18.08219948091820	20.11338655336740
Au	16.24137585667476	20.25541478626912	22.25663284027192
Au	18.23359981125470	18.22830435873915	22.24757166285943
Au	18.23632198655022	20.24545575608817	20.24693890312665
Au	20.24922957992399	18.22972342682348	20.24546658284535
Au	11.76174663046440	22.25678629808841	20.26046448464680
Au	13.99728833372720	22.07872819930884	22.08268975764341
Au	14.00070501542923	23.99791018628513	20.00788161274419
Au	16.24086552646842	22.25206322815369	20.25377556445264
Au	14.00766840743896	8.00501176325474	24.01023507328371
Au	9.95569967637130	11.93187051427222	24.12508379855538
Au	11.95231728889190	9.94462467409986	24.13029389756422
Au	14.00185409604453	11.95494732513922	24.37547951507056
Au	16.06292218423897	9.93825401719308	24.17335734396237
Au	18.05876115621198	11.93888245937464	24.16067955638832
Au	8.00794812166666	13.99132990541962	24.00611363190302
Au	9.95339038267532	16.03975136803629	24.14912387758719
Au	11.98791358910214	13.97261611594087	24.37186713688344
Au	13.99931223159121	15.98709673001115	24.32198142940890
Au	16.00670163880149	13.97264610188697	24.31693227980185
Au	18.05453790563780	16.03518841839652	24.10935292244874
Au	20.02614486790663	13.99504000448445	24.03111713058604
Au	11.95007164381605	18.03683251959201	24.13028617654964
Au	13.99877211699964	20.00610406054088	24.00759547216850
Au	16.05359084921448	18.04008625097917	24.10694488619545

Optimized PBE atomic coordinates (in Å) of $Ag_{161}Pt_{40}$ and $Ag_{158}Pt_{43}$ nanoparticles presented in Figure S1

$Ag_{161}Pt_{40}L1_0$-632.307

Ag	14.53194910265009	14.53163903330994	4.26945201897516
Ag	12.49991653022300	6.37590997410565	18.63909512570650
Ag	8.41248255739754	10.44280225967036	18.69181326851403
Ag	12.50017382582750	4.41774994861133	16.55347847563215
Ag	20.58209163949304	12.50008951670340	16.55393289566509
Ag	10.46788002099185	14.53248393489071	20.72942516103550
Ag	10.46766056740267	18.57579101719195	16.57953584257669
Ag	20.62231049813733	14.48012446700583	14.51752626594360
Ag	20.62214009553045	10.52001320961134	14.51797316116372

Ag	10.46859360770162	14.53153174882850	4.26866788940089
Ag	10.44290621421287	8.41271280065413	18.69199832866594
Ag	4.37751468831616	14.47993924146297	14.51700875243941
Ag	14.53194302987791	6.42439261658444	16.58084221710018
Ag	6.42417090968417	10.46802062133916	16.58039677531274
Ag	12.50011317791372	6.37584195931005	6.36166585590047
Ag	14.55743890997547	16.58756204857787	18.69082776182318
Ag	14.47990116751587	4.37728258648144	10.48325150087007
Ag	6.41014632263652	8.44525163371843	14.55361579627702
Ag	14.53201183695389	6.42414977962247	8.42028101740282
Ag	10.51988465607936	20.62250783605650	10.48221337337783
Ag	8.44499825755653	6.40970730845513	10.44653068837584
Ag	10.44288085314689	16.58721565300863	6.30781376874589
Ag	18.58975956450150	8.44532474016720	14.55421291513068
Ag	14.47991938816712	20.62269880680313	14.51715681336884
Ag	6.42418675052339	14.53206836429418	8.41885205663879
Ag	6.41045424398007	6.41008806354968	12.50023188916277
Ag	8.44499984017392	18.59034088539866	14.55321509173960
Ag	18.58952447862927	6.41023204632636	12.50044255262544
Ag	6.37575793563127	12.50003523740095	6.36048679675067
Ag	18.58967762629414	16.55492837671499	14.55353286714344
Ag	16.54605351027175	8.45387221336406	16.58595985149143
Ag	6.42430607179980	10.46791413802611	8.41926945103453
Ag	6.41025833716781	16.55483210056994	10.44603854703294
Ag	16.54631983102596	16.54618043361562	16.58503025913840
Ag	14.53202713189095	18.57588264256198	16.57981649642774
Ag	16.55520016286361	6.40974840433244	10.44676039919183
Ag	12.49997251997099	4.37720361921115	12.50067008659976
Ag	10.52001677023337	20.62280663080784	14.51695577561942
Ag	14.48015986335910	4.37745506958576	14.51781453169303
Ag	12.50033298840011	12.49947362583744	4.15304443472627
Ag	8.44499735893910	18.59025370846922	10.44586422107155
Ag	10.46796344464671	6.42419572094400	8.42010757131362
Ag	12.50008843683973	20.62312870058920	12.49940561404807
Ag	18.58970229850265	8.44512898112056	10.44681924371331
Ag	10.44288080385542	8.41244136849685	6.30892263446595
Ag	16.55515593059205	6.41003084287600	14.55415872278467
Ag	14.53223349088189	10.46767046828728	4.27038656066082
Ag	14.55727143479793	8.41248720691558	6.30923561585183
Ag	18.58948566009601	18.59001933878776	12.49989136958957
Ag	6.41018902818230	16.55480963454281	14.55311778207259
Ag	16.55509234394480	18.59026405286766	14.55356607118802
Ag	18.57582927654950	14.53189750191455	8.41937462946685
Ag	10.46805527440828	10.46831709194665	20.73056635480977
Ag	6.41051677959800	8.44504372351343	10.44644378443253
Ag	8.45373709800537	8.45380486544320	16.58546265615499
Ag	18.58981234229957	16.55471861014404	10.44637418964962
Ag	18.62409983962528	12.50022837436449	18.63977311552268
Ag	14.53149138326122	14.53222370369967	20.73019751257310
Ag	8.41256400547154	10.44271530053513	6.30828482883522
Ag	14.53118373130136	10.46854306487687	20.73151194125875
Ag	4.37785250439494	14.47999469140229	10.48194114748904
Ag	16.55515578507367	18.59013281575815	10.44595641043430
Ag	4.37797668162005	10.51974192209328	10.48208095171638
Ag	12.50000159020355	18.62437345958392	18.63810484585673
Ag	16.58760791417456	10.44264754172280	6.30880847278838
Ag	8.41224266277854	14.55726814238088	18.69091144465309
Ag	10.44246936700957	16.58743426786251	18.69039108429206
Ag	20.62207315832775	10.52004346788632	10.48269341098825
Ag	4.37681377892496	12.50012440921564	12.49937095817588
Ag	14.53202821443531	18.57570184829568	8.41947371631796
Ag	16.58760284061020	14.55704287607481	6.30821926424242

Ag	6.41044625741510	18.59008947010299	12.49939900218962
Ag	6.37538416274877	12.49998171536573	18.63892845593123
Ag	18.57553952293019	10.46809014581684	16.58132208499467
Ag	8.44481634705689	6.40986341079358	14.55398447359407
Ag	4.37756175803851	10.52016352602730	14.51725546723180
Ag	8.45383457905785	8.45373630780531	8.41483516186143
Ag	10.52015131088385	4.37719612514275	10.48324290745781
Ag	20.62303097104822	12.49996032788739	12.50042011353104
Ag	16.54634900188222	16.54615176012473	8.41462102736861
Ag	20.62224000630780	14.47988806259271	10.48268920467055
Ag	16.54623734926203	8.45370065541502	8.41512106786320
Ag	18.62447561564956	12.49984402957371	6.36085436353399
Ag	18.57586890042143	10.46775569906276	8.41958803161316
Ag	10.51977927215142	4.37737392199930	14.51780077978020
Ag	18.57574258508652	14.53221365949483	16.58086009589373
Ag	6.42403244620690	14.53200007874899	16.58009639281105
Ag	8.45376619441804	16.54619132065830	8.41445907067122
Ag	16.58707749505054	10.44309550593435	18.69279608014108
Ag	14.48007246869704	20.62240206712686	10.48207913958571
Ag	8.41274750393755	14.55715308651350	6.30756360935733
Ag	14.55748454237192	16.58712475784210	6.30824514241099
Ag	14.55720082765392	8.41290002330595	18.69240717978827
Ag	10.46787340962145	6.42427963198189	16.58073810525743
Ag	12.49944309737097	12.50096704156075	20.84692397655691
Ag	16.58732141349211	14.55724707581641	18.69191640484575
Ag	10.46808222042229	10.46794757053095	4.26985011982462
Ag	10.46790203214646	18.57571778387832	8.41917811551310
Ag	8.45355313504638	16.54615499054791	16.58448351685739
Ag	12.50016245022540	18.62399717315196	6.36082952560714
Ag	16.54776629283677	12.50046696007366	20.64781288039988
Ag	12.50020133477727	8.45165115483825	4.35404372050254
Ag	16.54821265227238	12.49976786899762	4.35318166591583
Ag	12.49973983192915	8.45191298482014	20.64736771380596
Ag	20.58509678471771	16.54866078165377	12.50010954921102
Ag	12.50003364860458	20.58200630966198	8.44627911324441
Ag	12.49969167816924	16.54847139796756	20.64544512341196
Ag	12.49991068394091	4.41780955133864	8.44728154134378
Ag	16.54818524968871	20.58537944738865	12.49970700162640
Ag	20.58217735951449	12.49984078401992	8.44661092912797
Ag	4.41756546052649	12.50016768086490	16.55330059399385
Ag	8.45151901565956	20.58549982575230	12.49953041461495
Ag	4.41514628091224	16.54868230092680	12.49949752607025
Ag	12.50027734041683	16.54816825068733	4.35298514047735
Ag	12.49999884840437	20.58204166748586	16.55304942744937
Ag	8.45204433666175	12.50030331834606	20.64662754264197
Ag	4.41776942427727	12.49992028634450	8.44614367760635
Ag	16.54830280789388	4.41471835466872	12.50053098836440
Ag	8.45152258330429	4.41453872312039	12.50029826342200
Ag	20.58490570247823	8.45127687276999	12.50044982731546
Ag	8.45194405761646	12.50003244617872	4.35272236731297
Ag	4.41514422300922	8.45133753296214	12.49993853574927
Ag	6.43883871715494	10.50048828220120	12.49980836239778
Ag	12.50015689678141	16.54160038593958	12.49976972016979
Ag	18.56123526875696	14.49965132466678	12.50010247674017
Ag	16.51974813313101	16.51938063288085	12.49977500994983
Ag	10.49504285526100	14.50470132911933	16.66622889180690
Ag	8.47353652229240	12.50003857795904	16.62164003718069
Ag	10.49376798061513	10.49367411715300	12.50021242471302
Ag	12.50002998794447	12.49997655730923	16.67948914735590
Ag	10.49992275305894	6.43871642750338	12.50033322673431
Ag	12.50009900826458	12.49988425584960	12.49990518984248
Ag	14.50644373959686	14.50622949244903	12.50004023835853

Ag	12.49999356320871	8.45827611552037	12.50045162372381
Ag	14.50630884818588	10.49376706282382	12.50003085959383
Ag	14.50497652637027	10.49516223703476	8.33369818074551
Ag	14.50007815343451	6.43874046811406	12.50049309516595
Ag	12.50013148646010	12.50015812027931	8.32054873586261
Ag	10.50002093732919	18.56120846763428	12.49971500195527
Ag	12.49995119240271	8.47385767016803	16.62207392447777
Ag	16.54172142363078	12.50002934800675	12.50018197876675
Ag	16.52648819385472	12.49989152989593	8.37795840932416
Ag	12.49994638821459	16.52615820303271	16.62109637048274
Ag	16.52626215599496	12.50008157681719	16.62243725699420
Ag	10.49363315390793	14.50624738547649	12.49961217356049
Ag	8.47380696255450	12.49999821947248	8.37767967092508
Ag	10.49510422013767	10.49517014859955	16.66677088104268
Ag	10.49528857283277	14.50479150162499	8.33309359553146
Ag	6.43886980132809	14.49967518202075	12.49961267124556
Ag	12.50002220214688	16.52603940282634	8.37793222599780
Ag	14.50502095636450	14.50474377014500	16.66644316451196
Ag	14.50007530194808	18.56112703035922	12.49969849460290
Ag	14.50475766567836	10.49514732561203	16.66706042635284
Ag	18.56106053451984	10.50040798208335	12.50041901614341
Ag	16.51932429145490	8.48075932370246	12.50044509028777
Ag	10.49514643051410	10.49520856573023	8.33340490214266
Ag	8.48041916921920	16.51940827116755	12.49966399690808
Ag	8.48066140937073	8.48061324137415	12.50019126475221
Ag	14.50500647691747	14.50471792536519	8.33329228956940
Ag	12.49998179078537	8.47380211279132	8.37894398449864
Ag	8.45834672824662	12.50013474961117	12.49996149901103
Pt	14.47174566909354	12.50012071444277	14.58627921669224
Pt	16.48532979221644	14.50545454678353	10.42249220980031
Pt	18.54901626152069	12.50000827165925	10.42230276535247
Pt	8.51484318770677	10.49437851273656	14.57757387295411
Pt	6.45097898892124	12.50002192575717	10.42176736458743
Pt	12.49992310573280	14.49468401155758	18.77821805092912
Pt	14.49471362871071	12.50005973306864	6.22127650054588
Pt	14.49440229484402	12.49995369091768	18.77923935029672
Pt	12.49989239565052	10.52849080914878	14.58641739047095
Pt	12.49995262629463	6.45099987430943	10.42279340890671
Pt	12.50025302074627	14.47147066296672	14.58578947648000
Pt	10.49407023502295	16.48503159597340	10.42238192579624
Pt	18.54901077003228	12.50004350280313	14.57834648113474
Pt	10.49418156948894	16.48511456631946	14.57699786509150
Pt	10.52857663646266	12.49976237702253	14.58607024595979
Pt	10.50536313530043	12.49991240150218	18.77853557293107
Pt	14.50597356628148	16.48517486971448	10.42249628932669
Pt	6.45078678909690	12.50005739903492	14.57750345349752
Pt	14.50584252785275	8.51493511267242	14.57773824515673
Pt	12.49994629921827	18.54897286996228	10.42196725931353
Pt	14.50591395874305	8.51490232236950	10.42311139826192
Pt	10.49416351141055	8.51493960184646	10.42294477449552
Pt	12.50003160179196	14.49438561247013	6.22087207492884
Pt	12.49986463676291	14.47144492693256	10.41390121060135
Pt	16.48512030418865	14.50556380299201	14.57754711140201
Pt	12.49991099481945	18.54898359062880	14.57722415941594
Pt	12.50006334297083	10.50567241653731	18.77923328589580
Pt	16.48529859693563	10.49444114846409	14.57795961644489
Pt	14.47149766551244	12.49988088546465	10.41392742342381
Pt	10.49405969897547	8.51506050676967	14.57751440779218
Pt	8.51468335492726	14.50546854665787	14.57720018209861
Pt	12.49993522522063	10.50541379719409	6.22142383913007
Pt	10.50542039499835	12.50007428303707	6.22097311554124
Pt	12.50020430152928	10.52858990167277	10.41420559885688

Pt	12.49991513183552	6.45105287745704	14.57823099661832
Pt	8.51476919142367	10.49446263392061	10.42242612271156
Pt	8.51492678917416	14.50553731370678	10.42225131550659
Pt	16.48517118519241	10.49430934828688	10.42272174668982
Pt	14.50594242391553	16.48504921495693	14.57705444447911
Pt	10.52861137193311	12.50028177791130	10.41386967786588

$Ag_{161}Pt_{40}$LEH-633.801

Ag	20.48964668351552	8.54420556574566	12.51090955148534
Ag	16.43573318035360	12.55444452608004	4.46521956876841
Ag	10.43876839953160	4.43631580818169	10.50472225418965
Ag	18.59478168608337	16.72674031213704	14.62371703678675
Ag	10.30598649281587	18.79529838790009	8.37740959945793
Ag	18.59808171339988	14.63854455382477	16.61040202626096
Ag	14.50045671273806	6.50227724309892	16.55877739515491
Ag	16.50562534740690	20.73377562841173	12.53188492128669
Ag	8.42191350232702	8.59531117816150	8.50934557441481
Ag	10.44460896501010	6.56432665167637	8.49931892102069
Ag	16.44749195721730	8.59104018118591	16.54059170333557
Ag	12.43432703299185	18.69330569936864	6.43450633367026
Ag	10.40922535694532	18.70403119009543	16.56345861285595
Ag	16.50771418090486	14.60061051993947	18.59370795993673
Ag	14.48654111067087	8.51905116732335	6.44028602814645
Ag	18.48980804692971	12.54420407254686	6.45799673750189
Ag	20.48560946085776	12.55112917764572	8.50988363131789
Ag	12.45529005877343	4.49866771843957	16.55623952295364
Ag	10.41766110641414	16.61161939154423	18.60293380599834
Ag	16.50610075803426	16.64997031427401	16.57769855727756
Ag	18.45606004663790	6.51012810793257	12.52506731073091
Ag	8.37229471669705	18.69530616862381	14.58126500231336
Ag	8.37492284596588	10.49971332862509	6.38889265826038
Ag	8.38086106225086	10.51989629805078	18.62373425210067
Ag	12.46611227493336	4.35084935411576	12.51474053994908
Ag	14.48932090070593	10.52739652273650	20.71100864165806
Ag	10.41921279611910	20.83539223083343	14.54528952868637
Ag	6.25278609543618	16.70315886892637	14.63022527234106
Ag	12.46838885425758	8.50986463479645	20.63031224652036
Ag	20.56512914825008	10.54280813897746	14.51750915327980
Ag	6.30700225406602	10.50528045250466	8.44810059827227
Ag	6.29528748565011	14.64937664620339	8.40883303493982
Ag	4.31927185176087	8.49528603787402	12.51232920401239
Ag	12.47103787938023	12.55296539082445	20.83791587122556
Ag	10.43471293758247	4.41664724122246	14.53236134204904
Ag	12.45217906198676	6.55966562221696	6.48501453163905
Ag	18.48863592363337	8.51918577071363	10.49759533651530
Ag	16.48408630293265	14.60941541368859	6.47071050415650
Ag	8.38156178586287	20.70879637115698	12.53755991887691
Ag	6.29596994934619	16.68499845182427	10.43211348659429
Ag	14.48303244911219	4.40963059967108	14.54014667913135
Ag	10.44977627180329	14.54689666058339	20.68338545367741
Ag	14.47350459026031	14.58186720953464	4.44257089482723
Ag	8.39907489655366	14.59953537693732	18.58362823782484
Ag	4.29786020653646	12.54988883834809	16.56844784724614
Ag	10.41975892704577	8.53524191717888	18.61047133360141
Ag	8.33724957195722	14.64586036325075	6.38329345262705
Ag	14.47629388619127	10.52026619659935	4.38290116125738
Ag	10.41969063315721	6.51015338784261	16.55347655759910
Ag	16.60638611551656	18.81541460976674	14.66788539357644
Ag	18.53896016906956	18.70500263738506	12.53216338225880
Ag	8.38287462956068	12.57046732174951	20.61752782189773
Ag	12.46520725831710	16.60422636356360	20.61429173080342
Ag	16.46253528410093	6.53831427923828	14.52908426851610

Ag	4.32010537592230	12.53787101400676	8.46003372808361
Ag	14.49117254322867	16.61897177491289	18.58981693901229
Ag	4.22803821149946	10.52740378066107	14.53260515690239
Ag	18.56643129826175	12.54428990372239	18.62620364765905
Ag	14.55792911220551	16.76296447237426	6.36851164751436
Ag	6.32325468006115	8.51580342909064	14.56509289869398
Ag	10.43685185865578	8.57271803510619	6.45194305816160
Ag	20.49999190361801	14.57455764686931	10.50305187658616
Ag	12.44923898700849	18.68808589529477	18.62933776517179
Ag	14.48355461681590	4.40774138731831	10.49224875874233
Ag	12.44142181169125	20.71126454986887	8.48086574591076
Ag	20.61101951317156	14.59362069557499	14.55336287053238
Ag	16.58952234689765	18.83501396363720	10.40305408280955
Ag	12.45522070212792	4.50054899574140	8.48773602296561
Ag	8.41299185001375	12.54520960982144	4.41386388764583
Ag	8.41387301440880	4.49339491717055	12.52439215883011
Ag	14.49893862540164	20.83234721720084	10.47745281740843
Ag	14.46630211994106	6.54584480119850	8.51092575006606
Ag	14.56948371236664	18.82811245153886	8.38569189917731
Ag	16.48934172217217	10.51341382014755	6.43707800821503
Ag	18.47718932341222	8.56983034071737	14.51994202167614
Ag	12.44752944437716	18.77590716784082	10.51705941487569
Ag	12.45980756409895	6.47456861848387	18.61659396377768
Ag	12.43926274462201	20.70413301726146	16.57063718588842
Ag	10.41654674867846	14.57930301053190	4.36822073195283
Ag	10.34777863583746	16.70116267376369	6.37894138868763
Ag	8.40335635843505	6.50556098844156	14.55580763488304
Ag	16.47723498653452	8.54664834376851	8.48857836758592
Ag	14.49272434144171	20.80047832133411	14.56876814720276
Ag	16.48874810187546	4.43790088018998	12.52527205212713
Ag	20.59694947731135	12.55173611380606	12.49875205616535
Ag	18.56885468809338	10.50534527462654	16.61724559995512
Ag	8.30901835371506	18.75956359558114	10.42634566241588
Ag	14.54891006165264	18.78419463658685	16.63337752284379
Ag	4.24422180507395	14.57381916078760	14.54605456188799
Ag	18.58619818528939	16.76720326827988	10.41684383347253
Ag	12.45709730526077	20.94834387272086	12.52342195074705
Ag	18.51643497760347	10.51212384309965	8.45626684304709
Ag	4.33702465758813	16.59884913263597	12.51362127807474
Ag	16.52234847300776	10.50430898697041	18.64588135900421
Ag	14.48392680013727	14.56675667297146	20.68459995186693
Ag	12.47562741307809	12.54530457406280	4.32116076538092
Ag	6.34109623883743	12.53266799416511	6.41216463543273
Ag	6.34028534065670	6.46623035821301	12.52088621827553
Ag	20.52856917265123	10.53579366078777	10.50911317793000
Ag	6.33463080899733	18.67985492605118	12.53080432503388
Ag	14.50372047138964	8.52115518699225	18.62647109290313
Ag	16.45747881685003	6.52042654807728	10.50305714846054
Ag	6.26963976014665	14.64856130257209	16.61790504314989
Ag	6.32230449081759	12.54792421112903	18.62030760840150
Ag	16.56453841868032	16.78113229298918	8.40007510484227
Ag	20.52568511628351	16.61405968801825	12.52378063261532
Ag	8.43999084832505	8.59552335640962	16.52846047072062
Ag	18.47953009661400	14.60169279110727	8.48244590493866
Ag	8.42709706678157	16.60017232516329	16.53017560024118
Ag	4.27323600809543	14.57954251611637	10.47969065033278
Ag	4.10390460171377	12.55731091734208	12.51496670861093
Ag	12.46217636805898	8.52214508522614	4.38553309668844
Ag	10.44452525558238	10.55849288091712	20.68469140361285
Ag	12.44660102455554	16.60973693765579	4.44227357675971
Ag	10.40262486715100	20.77570584254052	10.50694901219443
Ag	8.41110988792260	6.51542774699563	10.47777673154177

Ag	4.21916081528140	10.51898406550881	10.49639968268442
Ag	20.57094432911882	12.54782844720748	16.56296301563447
Ag	10.42322333185211	10.51824639425448	4.30420031343023
Ag	6.30971988798530	10.51768538887167	16.58702951797570
Ag	8.32612811608739	16.70576619302643	8.41069980578034
Ag	16.52156501385695	12.55647553456761	20.62629773704844
Ag	8.38819517089153	12.57850055490767	16.55428798034541
Ag	10.42265065175619	18.74424380863656	12.53987798002880
Ag	10.43918605640757	6.52188472100871	12.51827607254652
Ag	14.45907379946611	6.51182159724499	12.51734293687193
Ag	18.55438764146089	12.56335744361617	14.54900723283360
Ag	8.37828563634723	12.57677490021487	8.45869478984261
Ag	10.41140883057768	16.69575954696468	10.48617918767265
Ag	12.46286779713872	8.55358263838044	16.55388629711306
Ag	14.47071433751254	16.71967684127656	10.49744410008869
Ag	8.38528483009271	8.55748327669296	12.51180874500762
Ag	8.37512367141251	14.59932727542102	14.51308949223329
Ag	14.46759090088775	12.55427210839883	18.62195547947091
Ag	10.41892828593844	12.54814432017474	6.41129828304804
Ag	16.50199422544981	14.62600046327882	14.52844942600165
Ag	12.45947775324156	14.55815145004596	18.61659687767590
Ag	12.44782395542665	16.70000848114020	8.44989151704585
Ag	14.47461554702906	18.78480226246220	12.52390918383896
Ag	8.37435233702423	14.60829281598177	10.50831599461307
Ag	6.29470709076277	12.55360042138192	14.53063885736709
Ag	6.31122289709225	10.55530775569760	12.51404351274962
Ag	12.46170804198339	16.65584022398607	16.56024505373748
Ag	12.45874329305346	6.51519549897320	14.51760111762045
Ag	14.47470642357342	16.68359976698316	14.53006910629367
Ag	6.29986038305028	12.55232670051434	10.50398293039464
Ag	12.44783323037978	18.75562756078583	14.51641385879312
Ag	6.29536583896584	14.57002713057067	12.52187323779725
Ag	12.45620585290602	10.56736490562082	6.42405155217948
Ag	16.50073551382071	12.57544001594752	16.56438430281395
Ag	10.44122487564483	12.56905059547481	18.60893955571098
Ag	6.32316643209423	8.51280659025008	10.46828386500157
Ag	8.37994580543662	16.65852528856286	12.52463443919209
Ag	16.49515365850420	14.64877716237567	10.50334878476232
Ag	12.45551992520314	6.52810173907054	10.52512980591523
Ag	12.44886801577810	14.57820416484839	6.41714284990459
Ag	18.53997255283347	14.60993131651852	12.53016830895224
Ag	10.42569205659737	14.62077033006695	8.46228672655341
Ag	16.51241198988466	16.70567721236746	12.51542810268945
Ag	12.46264406128651	10.57222200050834	18.61916546372467
Ag	14.45984511302228	14.64740959293839	8.47643236543832
Pt	10.45801336581706	10.60685166250550	16.53311379355454
Pt	12.45537855012849	12.56928700993665	16.47114613352531
Pt	12.43521253041777	14.62171036366233	14.49594199760809
Pt	14.45290560460064	12.61414291815853	14.48706401197136
Pt	10.46129019083331	14.60814106164751	12.51821495257817
Pt	10.47528195947327	16.62743138728958	14.50298230918362
Pt	12.42279171247468	10.60634415197430	10.51423571603002
Pt	12.47170753502671	8.63438009009922	8.53558924822520
Pt	16.46394792505034	12.59191747244141	12.51284231014790
Pt	14.44699926789023	10.61479839970237	16.51057699495094
Pt	12.45297571602704	12.58767300273182	8.52259726293081
Pt	14.44655327423357	8.64087212268953	14.48800673168603
Pt	12.43094732275816	10.58709073824131	14.52173598837442
Pt	10.46137500611215	12.58443133210789	10.52274348139406
Pt	10.47232766580067	14.57706644723918	16.51091410403166
Pt	16.47645826521204	10.59546708744325	10.51363086703280
Pt	8.39686479485496	10.59808370447470	14.53189538692541

Pt	16.47959213704413	10.60280392174228	14.51711425393304
Pt	18.48120111093651	12.54473538546004	10.51374966258652
Pt	8.42027534233791	12.56048329083322	12.51185300406522
Pt	14.43755051468523	12.58855159516547	10.52433914006948
Pt	18.49189240992374	10.59032447828989	12.49759537521440
Pt	10.44266841835903	10.61140639021432	8.51533683796856
Pt	12.44201708935360	16.63355696693178	12.51084870459235
Pt	10.40107796478944	10.61038733749213	12.54349576917020
Pt	16.45836996559638	12.54923845798759	8.50855874586131
Pt	14.44918350916746	10.62159316486086	12.54450111536591
Pt	10.44282617624749	12.57642567726269	14.48518800563407
Pt	14.45141626711076	14.62790705573133	12.50024794403758
Pt	10.46306930015192	8.64696287847722	10.52091447035354
Pt	12.43778193470330	14.64756380321246	10.50559665017262
Pt	10.44370984724315	8.61804607657356	14.50853615584634
Pt	14.42940972319213	14.57858266751057	16.52784407621601
Pt	8.40670873396667	10.61080465803305	10.51147852951128
Pt	14.45872579587655	12.54954354769453	6.47756742571316
Pt	12.43941105236605	8.61303763349768	12.52914984247164
Pt	12.46213534597399	12.62143931768964	12.48264829584479
Pt	16.42912377895005	8.62493374284524	12.50241345243115
Pt	14.43640508331964	8.66964490035535	10.54574262213402
Pt	14.46442690961426	10.60299307180019	8.51365663715359

$Ag_{158}Pt_{43}L1_1$-645.077

Ag	20.62306374032496	8.45077398767555	12.52406638551157
Ag	16.53550846631271	12.49281244347260	4.36095030643278
Ag	10.47751733632905	4.28424587107382	10.47751733632905
Ag	18.61851619182606	16.59344487913657	14.54719557978727
Ag	10.39600437934740	18.66331423164135	8.36549735091817
Ag	18.57861155184207	14.49529311848059	16.56772070471313
Ag	14.60397062065267	6.33668576835866	16.63450264908183
Ag	16.53550846631271	20.63904969356722	12.50718755652740
Ag	8.41063930357533	8.40916795018774	8.41066430357527
Ag	10.49772537538191	6.44247967613867	8.49012598713885
Ag	16.63404618847453	8.36595381152547	16.63402118847459
Ag	12.46642702119083	18.60955797171303	6.39046702828708
Ag	10.45280442021273	18.61854119182600	16.59341987913645
Ag	16.56774570471307	14.49529311848059	18.57861155184207
Ag	14.50227462461809	8.49012598713885	6.44250467613861
Ag	18.54351045333435	12.50000000000000	6.45648954666565
Ag	20.63904969356722	12.50718755652740	8.46451653368740
Ag	12.52409138551168	4.37693625967505	16.54920101232451
Ag	10.49772537538191	16.50987401286115	18.55749532386139
Ag	16.58933569642473	16.59083204981226	16.58933569642473
Ag	18.60955797171303	6.39044202828697	12.53357297880917
Ag	8.43225429528693	18.57863655184201	14.49531811848052
Ag	8.43225429528693	10.50470688151941	6.42138844815792
Ag	8.40655512086344	10.45282942021284	18.61851619182606
Ag	12.53979875051816	4.38697340837479	12.53977375051805
Ag	14.54355583553392	10.45644416446607	20.66274769618725
Ag	10.47505272343466	20.59767328663794	14.48524179049953
Ag	6.42138844815792	16.56774570471307	14.49531811848052
Ag	12.52409138551168	8.45077398767555	20.62306374032496
Ag	20.66274769618725	10.45644416446607	14.54358083553386
Ag	6.42138844815792	10.50470688151941	8.43225429528693
Ag	6.33668576835866	14.60397062065267	8.36549735091817
Ag	4.36095030643278	8.46451653368740	12.50718755652740
Ag	12.53979875051816	12.46022624948195	20.61300159162509
Ag	10.51475820950046	4.40232671336206	14.52494727656534
Ag	12.50000000000000	6.45648954666565	6.45648954666565
Ag	18.57861155184207	8.43225429528693	10.50470688151941

Ag	16.59344487913657	14.54717057978715	6.38148380817394
Ag	8.45077398767555	20.62306374032496	12.47590861448832
Ag	6.33668576835866	16.63450264908183	10.39602937934733
Ag	14.54355583553392	4.33725230381275	14.54358083553386
Ag	10.47751733632905	14.52248266367095	20.71575412892618
Ag	14.48521679049942	14.52492227656541	4.40232671336206
Ag	8.49012598713885	14.50227462461809	18.55749532386139
Ag	4.36095030643278	12.49281244347260	16.53548346631260
Ag	10.50468188151947	8.43225429528693	18.57861155184207
Ag	8.36547235091823	14.60397062065267	6.33671076835859
Ag	14.52248266367095	10.47751733632905	4.28424587107382
Ag	10.50468188151947	6.42136344815799	16.56772070471313
Ag	6.39072357205721	10.48123165105712	12.49307888758714
Ag	18.54351045333435	18.54351045333435	12.50000000000000
Ag	8.46449153368728	12.50718755652740	20.63904969356722
Ag	12.49281244347260	16.53548346631260	20.63904969356722
Ag	16.63452764908177	6.33668576835866	14.60399562065260
Ag	4.37693625967505	12.52406638551157	8.45077398767555
Ag	14.54717057978715	16.59344487913657	18.61851619182606
Ag	4.28424587107382	10.47751733632905	14.52248266367095
Ag	18.60955797171303	12.46642702119083	18.60955797171303
Ag	14.49529311848059	16.56774570471307	6.42138844815792
Ag	6.44250467613861	8.49012598713885	14.50229962461803
Ag	10.45280442021273	8.40655512086344	6.38148380817394
Ag	20.71575412892618	14.52248266367095	10.47751733632905
Ag	12.50000000000000	18.54351045333435	18.54351045333435
Ag	14.52494727656534	4.40232671336206	10.51478320950058
Ag	12.47593361448843	20.62306374032496	8.45077398767555
Ag	20.59767328663794	14.48521679049942	14.52494727656534
Ag	16.59344487913657	18.61854119182600	10.45282942021284
Ag	12.49281244347260	4.36095030643278	8.46451653368740
Ag	8.45077398767555	12.52406638551157	4.37693625967505
Ag	8.46449153368728	4.36095030643278	12.49281244347260
Ag	14.48521679049942	20.59767328663794	10.47505272343466
Ag	14.54717057978715	6.38145880817401	8.40655512086344
Ag	14.49529311848059	18.57863655184201	8.43225429528693
Ag	16.50987401286115	10.49772537538191	6.44250467613861
Ag	18.66328923164141	8.36549735091817	14.60399562065260
Ag	6.38148380817394	8.40655512086344	10.45282942021284
Ag	12.53357297880917	6.39044202828697	18.60955797171303
Ag	12.50721255652751	20.63904969356722	16.53548346631260
Ag	10.45641916446614	14.54355583553392	4.33725230381275
Ag	10.39600437934740	16.63450264908183	6.33671076835859
Ag	8.40655512086344	6.38145880817401	14.54719557978727
Ag	16.58933569642473	8.41066430357527	8.40914295018763
Ag	14.52248266367095	20.71575412892618	14.52248266367095
Ag	16.54922601232444	4.37693625967505	12.52406638551157
Ag	20.61302659162520	12.46022624948195	12.53977375051805
Ag	18.66328923164141	10.39602937934733	16.63450264908183
Ag	8.36547235091823	18.66331423164135	10.39602937934733
Ag	14.50227462461809	18.55752032386133	16.50984901286104
Ag	4.40232671336206	14.52492227656541	14.48524179049953
Ag	18.55749532386139	16.50987401286115	10.49772537538191
Ag	12.46022624948195	20.61302659162520	12.46020124948183
Ag	18.61851619182606	10.45282942021284	8.40655512086344
Ag	4.37693625967505	16.54922601232444	12.47590861448832
Ag	16.63452764908177	10.39602937934733	18.66331423164135
Ag	14.52494727656534	14.48521679049942	20.59767328663794
Ag	12.46022624948195	12.53977375051805	4.38697340837479
Ag	6.39044202828697	12.53357297880917	6.39046702828708
Ag	6.45648954666565	6.45648954666565	12.50000000000000
Ag	20.59767328663794	10.47507772343459	10.51478320950058

Ag	6.39044202828697	18.60955797171303	12.46642702119083
Ag	14.60397062065267	8.36549735091817	18.66331423164135
Ag	16.56774570471307	6.42136344815799	10.50470688151941
Ag	6.38148380817394	14.54717057978715	16.59341987913645
Ag	6.45648954666565	12.50000000000000	18.54351045333435
Ag	16.59085704981238	16.58933569642473	8.41066430357527
Ag	20.63904969356722	16.53548346631260	12.49281244347260
Ag	8.40914295018763	8.41066430357527	16.58933569642473
Ag	18.55749532386139	14.50227462461809	8.49012598713885
Ag	8.41063930357533	16.58933569642473	16.59083204981226
Ag	4.33725230381275	14.54355583553392	10.45644416446607
Ag	4.38699840837491	12.53977375051805	12.46020124948183
Ag	12.50721255652751	8.46451653368740	4.36095030643278
Ag	10.51475820950046	10.47507772343459	20.59767328663794
Ag	12.47593361448843	16.54922601232444	4.37693625967505
Ag	10.45641916446614	20.66274769618725	10.45644416446607
Ag	8.49012598713885	6.44247967613867	10.49772537538191
Ag	4.40232671336206	10.51478320950058	10.47505272343466
Ag	20.62306374032496	12.47593361448843	16.54920101232451
Ag	10.47505272343466	10.51478320950058	4.40232671336206
Ag	6.44250467613861	10.49772537538191	16.50984901286104
Ag	8.36595381152547	16.63404618847453	8.36595381152547
Ag	16.54922601232444	12.47593361448843	20.62306374032496
Ag	10.49662321518709	10.45296654283357	16.58130723140990
Ag	12.53143349820919	12.46859150179093	16.59946370452671
Ag	14.55036078461845	12.46508135124663	14.55036078461845
Ag	10.44963921538155	14.55033578461851	12.46505635124670
Ag	10.48120665105718	6.39069857205727	12.50692111241287
Ag	10.45294154283345	16.58133223140984	14.50337678481291
Ag	16.59948870452682	12.46859150179093	12.53140849820908
Ag	14.50337678481291	16.58133223140984	10.45294154283345
Ag	12.46859150179093	12.53140849820908	8.40051129547318
Ag	8.41866776859017	14.54703345716643	14.50337678481291
Ag	12.53494364875331	10.44966421538149	14.55036078461845
Ag	10.44963921538155	12.53491864875337	10.44963921538155
Ag	16.58133223140984	10.45296654283357	10.49662321518709
Ag	16.58133223140984	14.50335178481297	14.54705845716655
Ag	12.50694611241298	14.51876834894288	18.60927642794279
Ag	14.51879334894282	18.60930142794273	12.49307888758714
Ag	16.50987401286115	18.55752032386133	14.50229962461803
Ag	6.39072357205721	12.50692111241287	14.51879334894282
Ag	18.60927642794279	12.49307888758714	10.48120665105718
Ag	8.40051129547318	12.53140849820908	12.46859150179093
Ag	10.45294154283345	10.49662321518709	8.41866776859017
Ag	12.49307888758714	18.60930142794273	14.51879334894282
Ag	12.46859150179093	16.59946370452671	12.46859150179093
Ag	14.55036078461845	10.44966421538149	12.53491864875337
Ag	12.49307888758714	10.48123165105712	6.39072357205721
Ag	12.46508135124663	14.55033578461851	10.44963921538155
Ag	10.49662321518709	8.41866776859017	14.54705845716655
Ag	10.48120665105718	12.49307888758714	18.60927642794279
Ag	14.54705845716655	14.50335178481297	16.58130723140990
Ag	8.41866776859017	10.49662321518709	10.45294154283345
Ag	12.50694611241298	6.39069857205727	10.48120665105718
Ag	14.51879334894282	12.50692111241287	6.39072357205721
Ag	18.60927642794279	14.51876834894288	12.50692111241287
Ag	12.53143349820919	8.40053629547329	12.53140849820908
Ag	14.50337678481291	14.54703345716643	8.41866776859017
Ag	14.54705845716655	8.41866776859017	10.49662321518709
Pt	8.52968733034395	12.50000000000000	16.47028766965611
Pt	12.50000000000000	14.48790895258759	14.48793395258753
Pt	10.40423655431750	18.58549184583676	12.40743312985041

Pt	12.50000000000000	10.51209104741241	10.51209104741241
Pt	12.50000000000000	8.52971233034388	8.52971233034388
Pt	14.59576344568250	6.41450815416324	12.59254187014964
Pt	14.58765007162387	10.41237492837606	16.58257197692708
Pt	18.58546684583665	12.40743312985041	14.59576344568250
Pt	8.43377906548960	12.60174163834615	8.43377906548960
Pt	10.41234992837612	16.58259697692702	10.41237492837606
Pt	12.60176663834609	8.43380406548954	16.56619593451046
Pt	8.52968733034395	8.52971233034388	12.50000000000000
Pt	14.58765007162387	8.41740302307298	14.58765007162387
Pt	14.59576344568250	12.40743312985041	18.58549184583676
Pt	10.40423655431750	12.59256687014958	6.41453315416335
Pt	10.50401939655908	14.49595560344099	16.46994630491979
Pt	12.39825836165385	16.56619593451046	8.43377906548960
Pt	8.41740302307298	14.58762507162394	10.41237492837606
Pt	8.53005369508022	10.50404439655902	14.49598060344093
Pt	16.58259697692702	10.41237492837606	14.58765007162387
Pt	12.50000000000000	16.47028766965611	16.47028766965611
Pt	12.59256687014958	6.41450815416324	14.59576344568250
Pt	14.48793395258753	12.50000000000000	10.51209104741241
Pt	18.58546684583665	10.40423655431750	12.59254187014964
Pt	14.49598060344093	16.46994630491979	14.49598060344093
Pt	6.41453315416335	12.59256687014958	10.40423655431750
Pt	10.51206604741247	10.51209104741241	12.50000000000000
Pt	16.47028766965611	12.50000000000000	8.52971233034388
Pt	6.41453315416335	14.59576344568250	12.40743312985041
Pt	10.51206604741247	12.50000000000000	14.48793395258753
Pt	14.48793395258753	14.48790895258759	12.50000000000000
Pt	10.50401939655908	8.53005369508022	10.50401939655908
Pt	16.56622093451040	12.39825836165385	16.56619593451046
Pt	12.40745812985035	18.58549184583676	10.40423655431750
Pt	8.43377906548960	16.56619593451046	12.39823336165391
Pt	16.46994630491979	14.49595560344099	10.50401939655908
Pt	12.40745812985035	14.59576344568250	6.41453315416335
Pt	10.41234992837612	14.58762507162394	8.41740302307298
Pt	12.50000000000000	12.50000000000000	12.50000000000000
Pt	16.47028766965611	16.47028766965611	12.50000000000000
Pt	12.59256687014958	10.40423655431750	18.58549184583676
Pt	16.56622093451040	8.43380406548954	12.60174163834615
Pt	14.49598060344093	10.50404439655902	8.53005369508022

$Ag_{158}Pt_{43}$LEH-643.158

Ag	20.58764744391275	8.55246668122098	12.44401054320540
Ag	16.55656880678965	12.56942483862210	4.27910037727890
Ag	10.49461936605925	4.44863472086443	10.39411841970069
Ag	18.67968763743974	16.75923306876269	14.57430097055317
Ag	10.44794203479764	18.69130237204742	8.37223826998108
Ag	10.48409593352481	18.69702663219499	12.43159351562613
Ag	18.56582331620912	14.57936249248125	16.48554472828827
Ag	10.47263913260777	6.48865888470489	12.42182646940562
Ag	10.49238716909000	16.65340549965776	14.46146257245299
Ag	14.50866661760766	6.55436315581300	16.44929224093534
Ag	16.54561762205646	20.68127701647773	12.44272186099998
Ag	8.39806575024595	8.46818621032936	8.28685822922461
Ag	12.52802509794762	8.55496390052030	8.35054937017310
Ag	10.41493740968293	6.43788999017672	8.27006225601112
Ag	16.56503234590713	8.51523647259356	16.49704206929357
Ag	12.48827767676080	18.66693695462386	6.32483288055164
Ag	10.40378971410648	18.78244982796658	16.56443189659122
Ag	8.46759534629988	12.58026173952925	8.36532720478562
Ag	16.56446830154495	14.58985298854849	18.52008802095088
Ag	14.62589379359179	8.46337426241207	6.17498497148062

Ag	18.60056976438452	12.56136246490751	6.31233372935051
Ag	20.59661723929439	12.55122661589428	8.38140882463119
Ag	12.50504724012958	4.53511749639314	16.38866723678268
Ag	10.40173774464551	16.70495401654441	18.58892395443729
Ag	16.62069557289343	16.74278914581028	16.56477695522814
Ag	18.57085669423732	6.52385848755776	12.42619178078998
Ag	8.44760345324495	18.71256508716001	14.47996068667156
Ag	8.38839980862428	10.49608655349145	6.25033381550263
Ag	8.43096509263816	10.51001675587258	18.45986081617935
Ag	12.52350039544943	4.44390539128095	12.42838851379040
Ag	14.54491184980644	16.64949925085157	10.40898662684424
Ag	14.49078653974479	10.52749425454516	20.54563950589269
Ag	10.47625224352778	20.79546233224082	14.47090512565778
Ag	6.48359706889794	16.63628485544988	14.45822500686473
Ag	8.43416837762597	8.50221003785560	12.42607672148717
Ag	12.48499988236953	8.54166639801575	20.44687686995173
Ag	20.61309770433443	10.52581089809595	14.45175943360122
Ag	14.52758400153337	12.54734091937328	18.50151223156963
Ag	6.36738511780464	10.49316059362772	8.31329029492222
Ag	10.50441803982106	12.58890875949638	6.27479904115634
Ag	6.42204119925849	14.62815836750880	8.35039139078734
Ag	10.49780715940904	14.61197814110729	16.45995347744058
Ag	4.38193484831966	8.48240957366814	12.44701919119579
Ag	12.49212941802929	12.53462438620496	20.59326286091360
Ag	10.50621802612511	4.45630680918913	14.41938234200972
Ag	12.51551725319430	6.51269348610106	6.30594490527986
Ag	18.55936201016662	8.57464824822692	10.41753800126449
Ag	16.58741791396265	14.63499763289373	14.45576752066068
Ag	12.50067269015855	14.58585762120639	18.53473622403166
Ag	16.62076527391569	14.66165917388362	6.25296312912193
Ag	8.48737930975377	20.73242599239894	12.43164834865594
Ag	12.49127907148958	16.62759097691497	8.37948224490475
Ag	6.46368984546699	16.59783472733971	10.40208975252194
Ag	14.50763734431799	4.53132944028212	14.41995089146762
Ag	10.45516465326434	14.57983002240357	20.58817764684014
Ag	14.51043514642583	18.74288926589978	12.44663494411157
Ag	14.52143854456595	14.59548580064219	4.22252608587807
Ag	8.44997860567907	14.59614812437277	18.51580997354148
Ag	4.44024283374093	12.55982862830106	16.39480514938985
Ag	10.48136904988020	8.54843132447808	18.41477194349847
Ag	8.46876366682256	14.61477913658164	6.30013726046972
Ag	14.55082164231754	10.53134455045022	4.15962766590214
Ag	10.50640432828189	6.56130377147540	16.37967442934427
Ag	16.64657869632247	18.80320973330464	14.57208843674306
Ag	6.40003030092982	10.52098529007194	12.44463979863348
Ag	12.51050044421742	16.66589283757635	16.49703417946153
Ag	18.60017302432660	18.67537766973651	12.44533984721552
Ag	18.61391987581635	12.54816628221348	10.40654384250106
Ag	8.46422441332392	12.53479059977026	20.50423005131904
Ag	12.49300908405391	16.58237265555127	20.51805141380554
Ag	16.55471442357462	6.54338650415047	14.45870560955988
Ag	4.41872807363394	12.57610291731118	8.39500037612241
Ag	10.49348926631120	10.55415268070417	8.32909889247314
Ag	14.60132690710018	16.68865539417422	18.58759203052480
Ag	4.36199659205876	10.53087727875154	14.43729264993077
Ag	14.53983330321217	16.68381374520524	14.45896475480276
Ag	6.42274930991043	12.57804555720889	10.41818490738272
Ag	18.54853831674976	12.53204769151447	18.51294873317826
Ag	12.50071504347149	18.75650517874595	14.45618869719879
Ag	14.57963466537396	16.67158451249253	6.27760054814247
Ag	6.35916236883688	8.44652148179622	14.49795694854341
Ag	10.41438743550827	8.46937213049989	6.22544533342329

Ag	16.57672856102893	12.56246015396619	8.35119138618917
Ag	20.67357251521219	14.57234487703712	10.41784363392961
Ag	12.50613922392016	18.67629526607413	18.53815296533796
Ag	14.52304533710616	4.51507677382267	10.42908407542129
Ag	12.49613377238846	20.68591230492957	8.38331151904035
Ag	20.59988352150663	14.57344596921038	14.47527747916162
Ag	16.62977155206001	18.74481631938848	10.32358134457445
Ag	12.52331778618909	4.51958375452057	8.37135809768871
Ag	8.45007596483520	12.58031699960868	4.29426190233986
Ag	8.46289573088410	4.46360000348326	12.42310868675377
Ag	14.51665768019886	20.74568993844991	10.40908358946101
Ag	14.56943794828926	6.54007352014902	8.33277961199794
Ag	10.47038822419027	8.51204770334822	10.36634052739463
Ag	14.57753021955371	18.70740733774561	8.32507032371640
Ag	16.62997909883295	10.46398209638959	6.18805379283203
Ag	12.52339110404315	10.57624425171982	6.23574171734685
Ag	10.48297590722751	12.55514170833881	18.47880487649208
Ag	18.57446125735578	8.51587321187255	14.46772353244539
Ag	12.47991359469125	18.70569532370025	10.42460376474561
Ag	6.36247665565229	8.45332042921113	10.35236149721038
Ag	12.48588300226812	6.53202333917709	18.42730099040003
Ag	12.51352204091629	20.71739453705458	16.49458540031625
Ag	10.49881371763484	14.56998037650511	4.15595521816462
Ag	14.53954924172807	14.61710517263191	16.48982468696820
Ag	10.46048320933442	16.61816576186291	6.30728592414749
Ag	8.40643375300712	6.43712514420879	14.48535694370699
Ag	16.52558877124330	8.58045833909637	8.35807300260719
Ag	14.54755191760109	20.80877050669030	14.48878924282661
Ag	16.52044786396009	4.55991658723628	12.43072234789122
Ag	20.68226406090963	12.54175920057247	12.46187676638958
Ag	18.59367113609625	10.50536134860654	16.50820581229203
Ag	8.43566041950544	10.53864173264222	10.37833971876388
Ag	8.48151366336450	18.66515817510870	10.39774692124355
Ag	14.61429419925683	18.79037142166089	16.57280593895673
Ag	4.42950392827857	14.59529951803159	14.43319090851463
Ag	16.59199823800832	14.60875741495475	10.40808537274092
Ag	18.66054024336620	16.68636191149248	10.34133480117098
Ag	12.52800742280536	6.52069998176798	10.39102276388900
Ag	12.49613930223041	20.90729197615318	12.44749324017106
Ag	14.52502088466299	12.56833081142395	6.24292761489240
Ag	12.49468585275831	14.57983631996983	6.27978205190434
Ag	18.62834478530978	14.59029579908877	12.44356634496690
Ag	18.60582489952930	10.50042549713199	8.31854205358223
Ag	4.47994994465720	16.61110475028303	12.44391627840037
Ag	16.56489442361911	10.50550183815435	18.52990381561612
Ag	14.52775214886839	14.56757288688130	20.58837047059688
Ag	12.51890456075940	12.56105305601461	4.03880039320392
Ag	6.40049508909526	12.56826653630883	6.30679101767516
Ag	6.39186909049015	6.44013247972578	12.44180765392302
Ag	20.63111622880784	10.54582844170791	10.43674716098331
Ag	6.46931216287049	18.67157995234652	12.42666563764601
Ag	14.50751089233598	8.55282708200036	18.45413118927953
Ag	16.51722221755157	6.58436567292830	10.41523516170433
Ag	6.47637250026536	14.58586710690687	16.45251340546326
Ag	6.45616560825682	12.54030226374007	18.44477395724676
Ag	16.62530342441258	16.68788611335520	8.30380531769294
Ag	20.60233527043530	16.60391164023589	12.45395072458794
Ag	8.45786847264885	8.52812058580621	16.40273483066389
Ag	18.66261393574488	14.66064893521282	8.30573654215877
Ag	8.44964099340553	16.66573728963565	16.51262659064667
Ag	16.58773472483559	16.67540478948019	12.44205193358515
Ag	4.40730845796481	14.57988638109377	10.45500318277539

Ag	4.34583067111111	12.57088742989459	12.45000352203749
Ag	12.50501427957751	8.54053676804012	4.27315308228949
Ag	14.54256165369143	14.59623725466264	8.34822607666837
Ag	10.48734602623957	10.52595630830142	20.49361352021583
Ag	12.48844362066127	16.61367262439108	4.29337461535683
Ag	10.49359859745367	20.81999126080718	10.41832956792332
Ag	8.39012904304421	6.43008456902671	10.32950131813852
Ag	4.36238247557137	10.53881477656304	10.42392184796862
Ag	20.57342051971952	12.53879649885659	16.44491531901740
Ag	10.47376895604238	10.56051336103779	4.22508089236318
Ag	14.51412986461657	10.59260406904088	8.34597045747927
Ag	6.44084794394663	10.53523532886680	16.39621477891638
Ag	8.51890013728310	16.57461772568415	8.40873121022203
Ag	16.52454633159446	12.52925487148581	20.56321088223651
Pt	10.48639645417251	10.59563711259045	16.39466638671144
Pt	12.51273873684831	12.56576082811812	16.40713960392388
Pt	8.48129260147818	12.55025488899624	16.38849041148625
Pt	12.54915161789915	14.59259863543226	14.42199162222488
Pt	14.54753636613002	12.59632027701142	14.43923290416120
Pt	10.52496938164230	14.61324376698633	12.45072756052837
Pt	12.47721570961130	10.60540257350870	10.43520424243576
Pt	14.51065197905480	6.57798083551443	12.44307836111938
Pt	16.55232798783322	12.56612786538026	12.44080902677515
Pt	14.53010019991079	10.57581146475352	16.40439670682003
Pt	18.58264129210093	12.52322488824426	14.43396606973170
Pt	10.52135406277753	16.56120032587537	10.46692369316816
Pt	12.50956783010076	8.64910069910055	16.35583539678236
Pt	12.51522563250272	12.58725097811039	8.40310424065263
Pt	14.53420497248962	8.60332622149208	14.41499034415897
Pt	8.47922479181471	14.57159405393417	14.40144316976230
Pt	12.50404129266809	10.62005360281938	14.38592834821291
Pt	10.52204017526953	12.56881748170678	10.43464981606203
Pt	16.51681258332691	10.59208425009165	10.42644569089163
Pt	8.53203744898986	14.55570892255338	10.45023759298695
Pt	6.44117956610916	12.57780766184962	14.39448154345185
Pt	8.52112074425317	10.58526150766923	14.38886975224097
Pt	16.53992465652716	10.56439879325235	14.45990826690230
Pt	12.51756274110580	6.58654477406561	14.37951291388922
Pt	8.49037156977906	12.57013383593682	12.43714155681577
Pt	14.53264692580395	12.60198116320658	10.43637864348260
Pt	18.56169652878323	10.56914932439753	12.45849275352520
Pt	12.53023649966668	16.60708156885947	12.43324929141347
Pt	10.47274426835536	10.57590709715987	12.38931147631721
Pt	14.53108624956222	10.61723398664598	12.41231667607164
Pt	6.46151541139855	14.58023905711890	12.44686184275189
Pt	10.51065867426252	12.61634831426868	14.41863632487041
Pt	14.55141581062857	14.59420490036767	12.43526504981872
Pt	16.54906802869321	12.52467272620855	16.43008934485990
Pt	12.52252080048789	14.57293175665606	10.44833728534040
Pt	10.50385980874530	8.60909587935167	14.37467709917403
Pt	8.47097914723775	16.59013817396594	12.40276469869464
Pt	12.50131180429510	8.59815978042572	12.41111177144203
Pt	10.52641809980018	14.54993860483489	8.42282439232854
Pt	12.53479728982014	12.59700342739945	12.45946287994044
Pt	12.50333564616208	10.57209822430579	18.43715862504534
Pt	16.50895644266809	8.60427886683490	12.43101890743833
Pt	14.49404796624503	8.63703807216556	10.44765050132505

****** END OF DATA *******
